# Bioinformatics characterization of BcsA-like orphan proteins suggest they form a novel family of pseudomonad cyclic-β-glucan synthases

**DOI:** 10.1371/journal.pone.0286540

**Published:** 2023-06-02

**Authors:** Andrew J. Spiers, Helge C. Dorfmueller, Robyn Jerdan, Jessica McGregor, Abbie Nicoll, Kenzie Steel, Scott Cameron

**Affiliations:** 1 School of Applied Sciences, Abertay University, Dundee, United Kingdom; 2 Division of Molecular Microbiology, School of Life Sciences, University of Dundee, Dundee, United Kingdom; 3 Nuffield Research Placement Students, School of Applied Sciences, Abertay University, Dundee, United Kingdom; Weizmann Institute of Science, ISRAEL

## Abstract

Bacteria produce a variety of polysaccharides with functional roles in cell surface coating, surface and host interactions, and biofilms. We have identified an ‘Orphan’ bacterial cellulose synthase catalytic subunit (BcsA)-like protein found in four model pseudomonads, *P*. *aeruginosa* PA01, *P*. *fluorescens* SBW25, *P*. *putida* KT2440 and *P*. *syringae* pv. tomato DC3000. Pairwise alignments indicated that the Orphan and BcsA proteins shared less than 41% sequence identity suggesting they may not have the same structural folds or function. We identified 112 Orphans among soil and plant-associated pseudomonads as well as in phytopathogenic and human opportunistic pathogenic strains. The wide distribution of these highly conserved proteins suggest they form a novel family of synthases producing a different polysaccharide. *In silico* analysis, including sequence comparisons, secondary structure and topology predictions, and protein structural modelling, revealed a two-domain transmembrane ovoid-like structure for the Orphan protein with a periplasmic glycosyl hydrolase family GH17 domain linked via a transmembrane region to a cytoplasmic glycosyltransferase family GT2 domain. We suggest the GT2 domain synthesises β-(1,3)-glucan that is transferred to the GH17 domain where it is cleaved and cyclised to produce cyclic-β-(1,3)-glucan (CβG). Our structural models are consistent with enzymatic characterisation and recent molecular simulations of the *Pa*PA01 and *Pp*KT2440 GH17 domains. It also provides a functional explanation linking *Pa*PAK and *Pa*PA14 Orphan (also known as NdvB) transposon mutants with CβG production and biofilm-associated antibiotic resistance. Importantly, cyclic glucans are also involved in osmoregulation, plant infection and induced systemic suppression, and our findings suggest this novel family of CβG synthases may provide similar range of adaptive responses for pseudomonads.

## Introduction

Advances in sequencing technology have added a new dimension to microbial molecular ecology allowing biochemical potential to be inferred from protein coding sequences [1, 2]. However, investigation of protein function remains difficult, and most new gene sequences are annotated based on homologies and should be viewed with scepticism [3]. Very few proteins have experimentally verified functions, misannotations are frequent, and many protein families remain annotated as hypothetical or uncharacterized [4–7]. Critically, homologous regions identified between pairs of proteins may not be responsible for conserved function [8] and the assumption that proteins with very similar sequences and structures should have similar functions may not always be reliable [9]. Domain recognition and modelling provide complementary means of recognising and comparing structural and functional (protein) folds and overall, functional predictions based on homologous sequences and structures have been improving [10–12]. However, ‘twilight’ proteins with less than 20–40% amino acid sequence homology remain difficult to annotate, as they are unlikely to share the same protein fold and therefore function [13–16]. Furthermore, functional homology may not be retained within diverse families, as subtle differences in sequences, folds, and active sites may result in altered activity [17].

We have encountered this problem in functional annotation through our attempts to understand the nature of a novel family of proteins initially annotated as bacterial cellulose synthase catalytic subunits (BcsA) or more generally as glycosyltransferases. Glycosyltransferase (GT) family enzymes [17, 18] catalyse the transfer of sugar groups from activated donor molecules to specific acceptor molecules (Leloir type GTs) and the Carbohydrate-Active enZYmes Database (CAZy) [19] (www.cazy.org) classifies these into distinct sequence-based families where the same three-dimensional structural and functional fold [20] is expected to occur within each family. However, in the GT family, enzymes with different donor and/or acceptors (poly-specificity) are common, making functional predictions problematic with 115 GT families assigned to 144 Enzyme Commission (EC) activities (CAZy, August 2022). This is not surprising, as over 75% of the GT families belong to one of three superfamilies (GT-A, B & C) [21]. Poly-specificity is also seen within the GT2 family enzymes [17, 22], which have an α/β/α sandwich Rossmann-like fold [18] that binds nucleotide sugars and are members of the nucleoside-diphosphosugar transferase (GT-A) superfamily [21]. Hypervariable loops in the shared core component of the GT-A fold contribute to diversity within this superfamily in which inverting and retaining catalytic mechanisms have emerged multiple times [23]. Poly-specificity also confuses homolog / ortholog / paralog distinctions, and a lack of clear evolutionary relationships between proteins limits both homology and non-homology-based functional prediction methods [24].

GT2 synthases transfer sugars to a growing acceptor molecule in a processive manner to produce long-chain cellulose, chitin, curdlan, hyaluronan and mixed-linkage glucans [25–31], as well as a range of other linear, branched, and cyclic polysaccharides (CAZy lists 18 GT2 family EC activities and over 20 ‘characterized’ bacterial synthases producing different polymers; August 2022). High-resolution X-ray structures [32, 33] are available for the *Rhodobacter sphaeroides* (now *Cereibacter sphaeroides*) 2.4.1 bacterial cellulose synthase catalytic subunit A protein (*Rs*BcsA), showing the GT2 domain which includes the conserved DD, DxD, ED, and Q(Q/R)xRW motifs in the active site cavity, the Rossmann-like fold, a transmembrane (TM) region that forms a channel through which the cellulose chain translocates through to the periplasm, and the cyclic-*di*-GMP–binding PilZ regulatory domain [25, 30–31, 34]. The *Rs*BcsA structure has been used to model related GT2 synthases through template-based modelling (TBM) and predictive modelling that uses Artificial Intelligence (AI) / Deep Learning methods incorporating free modelling (FM) approaches [35–37]. However, despite the relative ease of modelling novel GT2 sequences today, the question of how diverse homologous proteins can become before a significant change in function occurs is still relevant, despite the retention of a conserved protein fold and conserved motifs and residues.

We identified a *bcs*A-like gene in several model pseudomonads including the plant pathogen *P*. *syringae* pathovar tomato DC3000, and the plant and soil-associated *P*. *fluorescens* SBW25 and *P*. *putida* KT2440 [38–40]. Each of these strains requires BcsA and other cellulose synthase subunits encoded by the *bcs* operon to produce cellulose [41–46]. As the *bcsA*-like gene was not associated with these operons, we referred to it as an ‘Orphan’ and expected the protein to add to the pool of BcsA subunits to modify cellulose synthesis in different environmental conditions. However, as we begun to characterise the Orphan protein *in silico*, we realised that the link to cellulose synthesis through the conserved GT2 domain and associated transmembrane helices was misleading, and the identification of a second but smaller GH17 glycosyl hydrolase family domain suggested that the Orphan was more likely to be a transmembrane cyclic β-(1,3)-glucan synthase.

In this work we describe our identification and characterisation of the Orphan proteins in model pseudomonads and show that they in fact belong to a highly conserved group found across the genus and in other related bacteria. We use secondary structure predictions to identify the functional domains, and protein structural modelling to provide a consensus structure. We produce a functional model, in agreement with earlier enzymatic investigations and molecular dynamics simulations [47–49], which suggest that the Orphan proteins form a novel family of cyclic β-(1,3)-glucan (CβG) synthases and explains the production of CβG by the opportunistic human pathogens *P*. *aeruginosa* PAK and *Pa*PA14 that is associated with biofilm antibiotic resistance and tolerance [50, 51]. We suggest that the Orphan synthases may have a broader role in a range of environments, as other cyclic glucans are also involved in bacterial osmoregulation and adaptation, membrane structure, plant infection and induced systemic suppression [52–59].

## Methods

### Accessing genomes and protein sequences and other information

*Pseudomonas fluorescens* SBW25 [40], *P*. *putida* KT2440 [38], and *P*. *syringae* pv. *tomato* DC3000 [39] genomes were accessed through the *Pseudomonas* Community Annotation Project (PseudoCAP) [60] (www.pseudomonas.com) or downloaded as GBK files and viewed using Artemis [61] (www.sanger.ac.uk). Operon predictions [62, 63] (www.microbesonline.org) was used to assess whether orphan and *dapE* genes were likely to be part of the same operon. Additional information about proteins were obtained from Universal Protein Knowledge Database (UniProtKB) (www.UniProt.org) [64] entries. Gene synteny was also checked using EnsemblBacteria [65] (bacteria.ensembl.org) and NCBI Nucleotide [66] (www.ncbi.nlm.nih.gov/nucleotide). Unpublished draft genomes generated in other projects (A. Koza, K. Kabir & A. Spiers) from pseudomonad strains isolated in earlier work, including the soil-associated DBG-1, DBG-3, DBG-6, DBG-15, DBG-16, and DBG-23 strains [67] and the mushroom pathogen NZ092 [68], were investigated using Artemis and protein sequences provided (see **S1 File** for protein sequences analysed in this work). Locus tags and UniProtKB accessions are provided for key proteins where appropriate.

### Identification of other Orphans and other proteins

Orphan proteins were identified in PseudoCAP by searching complete or draft genomes using the ‘Pseudomonas Ortholog Group’ generated for *Pf*SBW25 DapE (locus tag PFLU1259; UniProtKB C3K5D5) [69]. For each genome sampled, the gene immediately upstream of *dapE* was examined and recorded if the protein had a functional annotation similar to the *Pf*SBW25, *Pp*KT2440 or *Ps*DC3000 Orphan proteins (i.e., *orphan–dapE* gene synteny was used for selection, but no alternative genetic arrangements involving Orphan homologs were seen in those genomes sampled from PseudoCAP). Further Orphan orthologs were identified by NCBI BLAST+ [70] (www.ebi.ac.uk) searches of UniProtKB using *Pf*SBW25, *Pp*KT2440 and *Ps*DC3000 Orphan proteins, and by sampling proteins listed by InterPro [71] (www.ebi.ac.uk) as having the same Pfam PF00332 –PF13641 domain architecture. Duplications were removed as well as metagenome or whole genome shotgun entries which were labelled as preliminary data in UniProtKB, and all searches were completed by the end of October 2021 (see **S1 File** for the final list of Orphan, BcsA and related proteins analysed in this work). PseudoCAP BLASTP search was also used to identify *Escherichia coli* MdoB (UniProtKB P39401) homologs in *Pa*PA01, *Pf*SBW25, *Pp*KT2440 and *Ps*DC3000.

### Pairwise and multiple sequence alignments, phylogenetic trees, and cladograms

Similarities between sequences were investigated using the Water pairwise sequence alignment tool [70] (www.ebi.ac.uk) and presented as heatmaps using JMP statistical software (SAS Institute, UK). Clustal Omega and MView [70] were used to produce and view multiple sequence alignments coded according to conserved physiochemical amino acid classes [72]. Sequence conservation was assessed by Shannon entropy using the Protein Residue Conservation Prediction tool [73] (compbio.cs.princeton.edu). Phylogenetic trees were produced from Clustal Omega multiple sequence alignments using Simple Phylogeny [70] and the computationally fast unweighted pair group method with arithmetic mean (UPGMA). Trees were constructed with distance correction on, as recommended for divergent sequences, and with a constant-rate assumption the distance from root to each tip is the same. We constructed UPGMA trees in an iterative approach which maximised the number of orthologs that could be processed and selected representative sequences for clades that could be removed without affecting the tree topology. Hierarchical Cluster Analysis (HCA) was used to cluster proteins using amino acid profiles obtained by Protein Stats [74] (www.bioinformatics.org) and JMP 12 using the Ward method with an equal weighting of variables.

### Functional domains, predicted secondary structure and topology, and modelling

Functional domains were identified using HMMSCAN [75] (www.ebi.ac.uk) and secondary structures and topologies predicted using HMMSCAN, LipoP [76] (services.healthtech.dtu.dk), Phobius [77] (www.ebi.ac.uk), PRED-TAT [78] (www.compgen.org), Proetus2 [79] (www.proteus2.ca), Protter [80] (wlab.ethz.ch), and SignalP [81] (services.healthtech.dtu.dk). Predicted structures (models) were produced using AlphaFold Colab Notebook [82, 83] (www.alphafold.ebi.ac.uk), IntFOLD6 [84] (www.reading.ac.uk/bioinf/IntFOLD), Phyre^2^ [85] (www.sbg.bio.ic.ac.uk/~phyre2), RoseTTAFold implemented through Robetta [86] (robetta.bakerlab.org), SWISS-MODEL [87, 88] (www.swissmodel.expasy.org), and TrRosetta [89–91] (yanglab.nankai.edu.cn/trRosetta), following the suggested approaches and default options and using the full-length protein sequences (i.e., including putative signal sequences). All modelling was completed by the end of June 2022 and Protein Data Base (PDB) files are available (see **S2 File** for a list of all models and DOI for downloads). Models were visualised using Mol* 3D Viewer [92] (www.rcsb.org/3d-view) using representation and residue property settings, and membranes visualised using the ANVIL algorithm [93] implemented by Mol*. Models were visually compared by identifying secondary structure features characteristic of the reference structures and quantitatively using Pairwise Structure Alignment [94] (www.rcsb.org/alignment) with FATCAT-rigid body alignments suited to the identification of structural equivalences between closely related proteins with similar shapes [95,96].

## Results and discussion

### Orphan genes are not duplications of the *bscA* cellulose synthase subunit gene

**Fig 1:**
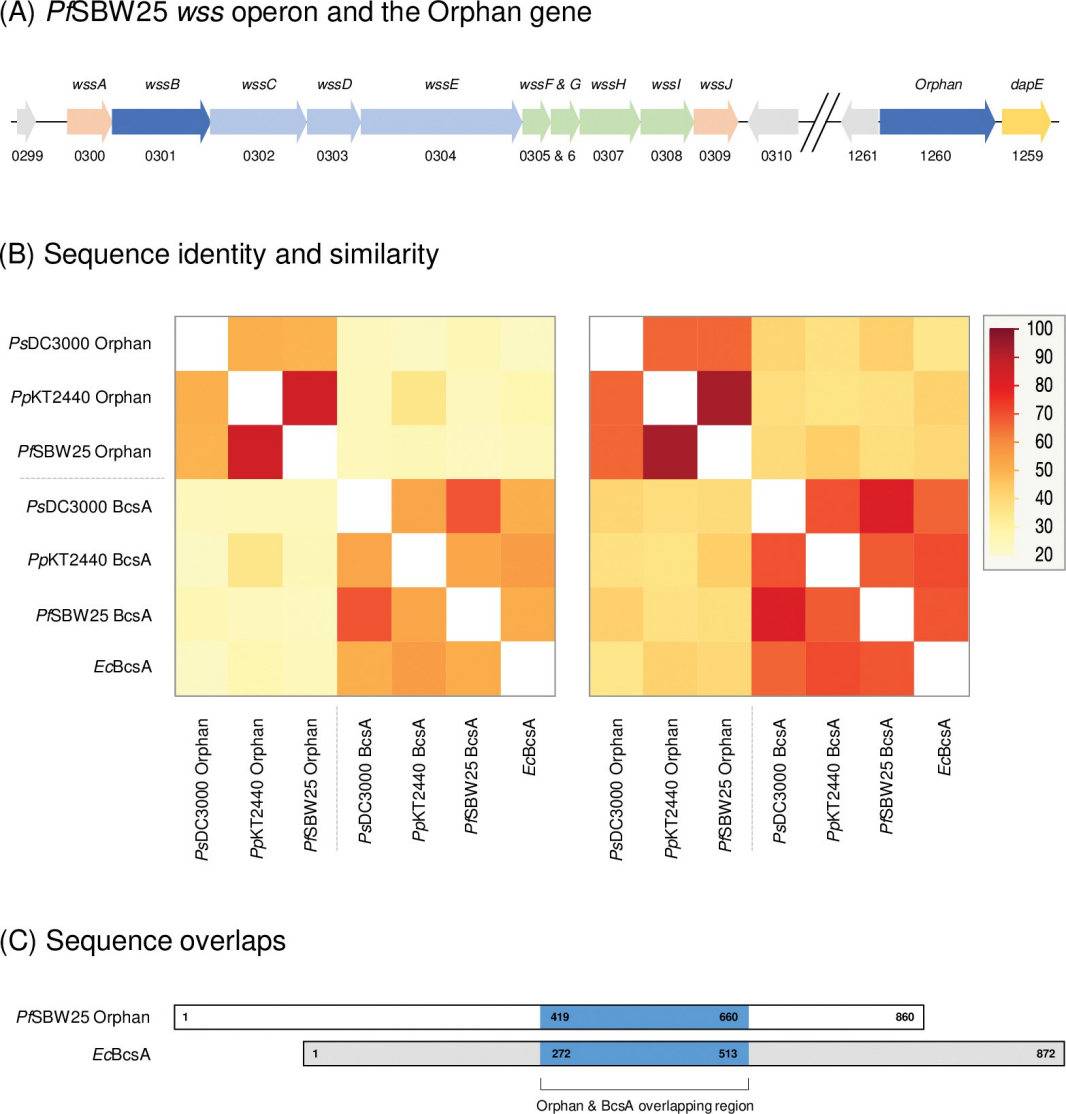
Orphan proteins are not recent duplications of BcsA proteins. Shown here is the bacterial cellulose synthase (*bcs*) operon found in *Pseudomonas fluorescens* SBW25 known as the Wrinkly Spreader Structural (*wss*) operon [41] **(A)**. WssB/BcsA (dark blue) and WssC–E (light blue) are the core cellulose synthase subunits, WssF–I (green) are involved in the partial acetylation of the cellulose polymer [41, 42], and Wss A and WssJ (rose) are likely to be involved in the positioning of the cellulose synthase and are required for cellulose production. A second BcsA homolog known as the Orphan is located upstream of *dapE* (yellow) in a different region of the chromosome. DapE and other genes (grey) indicated here are not involved in cellulose production. *P*. *fluorescens* SBW25 locus tag (PFLU) numbers are shown below the genes. Heatmaps of the amino acid sequence identity (left panel) and similarity (right panel) **(B)** determined from Water pairwise comparisons [70] of amino acid sequences of BcsA and Orphan proteins from *P*. *fluorescens* SBW25, *P*. *putida* KT2440 and *P*. *syringae* DC3000, as well as with the *Escherichia coli* MG1665 BcsA (*Ec*BcsA) reference protein, suggest that the Orphan genes are not recent duplications of the *bcsA* genes in these model pseudomonads (see **S1 Table** for protein locus tags & UniProtKB accessions and **S1 File** for protein sequences). These comparisons also show that the *Pf*SBW25, *Pp*KT2440 and *Ps*DC3000 Orphan proteins and *Ec*BcsA share a central overlapping region **(C)** of ~240 residues in the multiple sequence alignment (this region is indicated in blue with the residue numbers provided for *Pf*SBW25 Orphan and *Ec*BcsA proteins).

*Pseudomonas fluorescens* SBW25 [40–42, 97], *P*. *putida* KT2440 [38, 43, 45, 97], and *P*. *syringae* pv. tomato DC3000 [39, 44, 46, 97] each contain a bacterial cellulose synthase (*bcs*) operon encoding BcsA and other subunits required for cellulose production [98]. These pseudomonads also contain a second *bcsA*-like ‘orphan’ gene located in a different region of the genome, immediately upstream of *dapE* [69] and not associated with other cellulose synthesis-related genes (**Fig 1A**).

The Orphans are currently annotated as β-(1,3)-glucosyl transferases or glycosyl transferase family proteins in PseudoCAP [60], and we confirmed by pairwise sequence alignment that each has limited amino acid sequence identity (24.7–26.5%) with the functionally-active BcsA protein (UniProtKB P37653) from *Escherichia coli* MG1665 (*Ec*BcsA) which was chosen as a non-pseudomonad reference protein for this work [99] (see **S1 Table** for percentage identity & similarities). The Orphan genes are not recent duplications of the *bcsA* gene in each of the three pseudomonads, as pairwise alignments revealed limited DNA sequence identity (47.9–48.8%) and amino acid sequence identity (21.6–24.5%) between gene and protein pairs. Homologous proteins sharing more than 30% sequence identity are likely to share similar structure and function [16], but the poor level of amino acid identity seen between BcsA and Orphan proteins suggests that a BcsA or BcsA-like functional annotation for the Orphan proteins may not be justified.

We attach no significance to the positioning of the Orphan gene immediately upstream of *dapE*, as DapE has never been associated with cellulose production in bacteria, and Operon predictions [62] suggest that in *Pf*SBW25, *Pp*DC3000 and *Ps*DC3000 the orphan and *dapE* genes are unlikely to be in the same operon (Estimated probability that the pair is in the same operon, pOp values of 0.017, 0.038 & 0.207, respectively). The beginning of the Orphan genes is marked by a region of low GC content, and in *Ps*DC3000, the GC content of the orphan gene is clearly lower than that of *dapE* indicating recent recombination or horizontal/lateral gene transfer (see **S1 Fig** for GC content traces). Recombination is part of the dynamic nature of pseudomonad genomes including *P*. *syringae* [100], and gene amelioration is expected to reduce the difference in GC content of genes acquired by LGT over time [101]. This suggests that in *Ps*DC3000 the acquisition or repositioning of the Orphan gene might be more recent than it was in *Pf*SBW25 or *Pp*KT2440.

### Orphan proteins are more like one another than they are to BcsA proteins

The *Pf*SBW25 and *Pp*KT2440 Orphans could be aligned with the *Ps*DC3000 Orphan protein with 47.3–47.9% amino acid identity, with only five insertion-deletions (INDELs) of 3–9 amino acids required in the *Ps*DC3000 Orphan to align with the *Pf*SBW25 and *Pp*KT2440 proteins. Heatmaps based on Water pairwise alignments demonstrates that the Orphan proteins are more like one another than they are to their cognate BcsA proteins or to the *Ec*BcsA reference sequence (**Fig 1B**). Furthermore, the C-terminal region of these proteins overlap with the N-terminal region of *Ec*BcsA with a central core of ~240 residues with an over-all 21% amino acid sequence identity (**Fig 1C**). Our analysis of conserved domains, motifs, and residues described in a later section shows that the central core includes most of the active site found in BcsA proteins [25], but the C-terminal cyclic-*di*-GMP-associated regulatory PilZ domain is missing in the Orphans.

**Fig 2:**
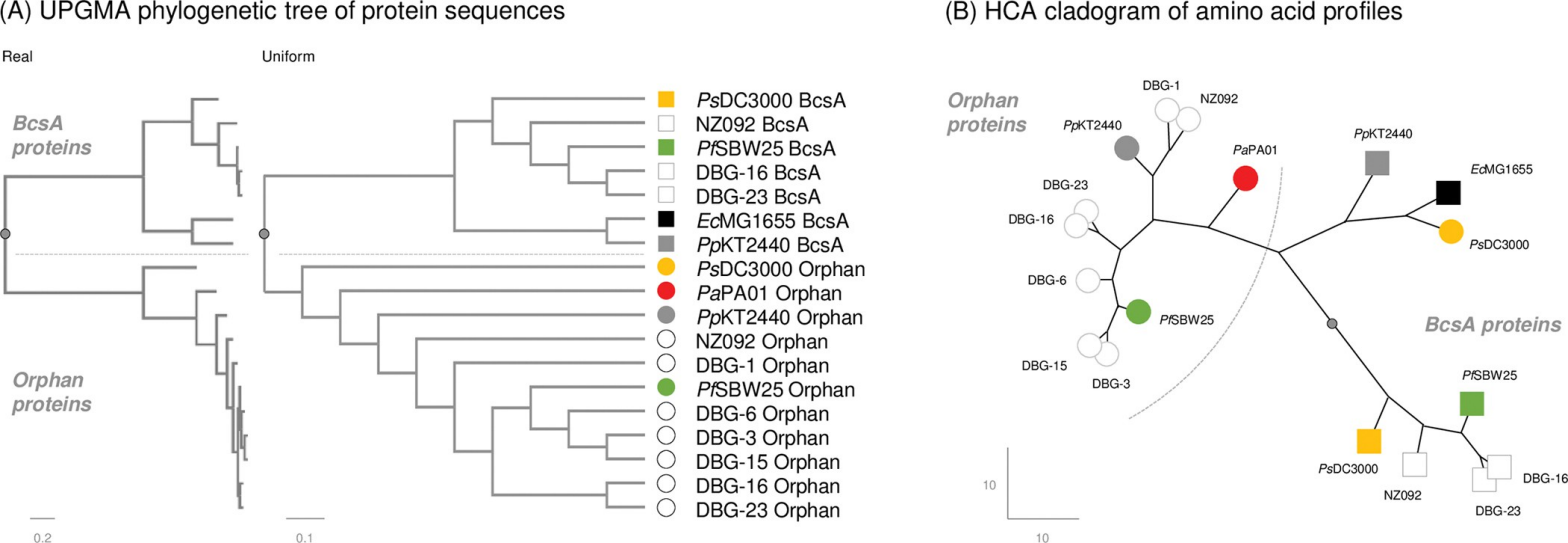
*Pseudomonas* spp. Orphan and BcsA proteins originate from two different but related groups of proteins. Shown here is a simple un-rooted UPGMA phylogenetic tree produced by Clustal Omega Simple Phylogeny [70] (**A**) of BcsA and Orphan proteins from eleven pseudomonads plus the *Escherichia coli* MG1665 BcsA reference protein (see **S1 File** for protein sequences). The tree is drawn with the real (relative) genetic distances and as a cladogram with a uniform distance between the root, indicated by the small grey circle, and the terminal nodes shown as large circles for the Orphans and squares for the BcsA proteins. The dashed lines separate the two main clades of the tree with the Orphan and BcsA proteins in different branches. The real and uniform distance (horizontal) scales are shown at the bottom-left of each cladogram. The Orphan and BcsA proteins can also be differentiated by hierarchical cluster analysis (HCA) based on amino acid profiles (**B**). The same symbols and colours are used to indicate the arbitrary root which is located at the mid-point of the longest branch, and Orphan and BcsA proteins. The dashed arc indicates the branch containing most of the Orphans from the rest of the cladogram which includes the BcsA proteins and the remaining Orphan. The x-y scale is indicated at the bottom-left of the cladogram.

The difference between Orphan and BcsA proteins was maintained when we expanded our comparison to include Orphans identified in draft genomes of pseudomonads we and others had isolated earlier [67, 68], as well as Orphan proteins from *P*. *aeruginosa* PAK, *Pa*PA01 and *Pa*PA14, which are referred to as NdvB in PseudoCAP and elsewhere (*Pa*PA01 locus tag and UniProtKB: PA1163 / Q9I4H4). We had originally ignored *P*. *aeruginosa* strains because this species has not been reported to produce cellulose and we did not expect them to have *bcsA*-like duplications (the *P*. *aeruginosa* Orphans share 46.2–47.9% normalised amino acid identity with the *Pf*SBW25, *Pp*KT2440 and *Ps*DC3000 Orphan proteins). A phylogenetic analysis of this larger set of proteins placed the Orphan and BcsA proteins in separate clades rooted by a common ancestral sequence in the UPGMA [70] tree (**Fig 2A**). This relationship was confirmed by HCA which does not presume common ancestral sequences but uses the amino acid profiles to group proteins instead, and in this, the *Ps*DC3000 Orphan was found to group with *Ec*BcsA and *Pp*BcsA (**Fig 2B**).

Further analysis of Orphan homologs indicates that they are widely distributed amongst the pseudomonads with 84 Orphans identified in 41 pseudomonad species with a further 28 in strains not classified to species level, and 42 additional homologs from other genera (see **S2 Table** for a list of *Pseudomonas* spp. Orphans; note that our selection of Orphan proteins for this larger set of proteins was by sampling and was not exhaustive). We undertook a UPGMA phylogenetic analysis of these proteins using the fungal *Schizosaccharomyces pombe* 972 α-(1,3)-glucan synthase Ags1 (UniProtKB Q9USK8) and the bacterial *Rhizobium meliloti* 1021 cyclic β-(1,2)-glucan synthase NdvB (UniProtKB P20471) as outlier sequences. It should be noted that while the *Pa*PA14 Orphan protein was given the same name as *Rm*NdvB because of (limited) sequence homology [50], *Rm*NdvB is a significantly larger protein with only 12.2–13.6% sequence identity with the *Pa*PAK, *Pa*PA01, *Pa*PA14, *Pf*SBW25, *Pp*KT2440, and *Ps*DC3000 Orphan proteins (*Rm*NdvB was named because of its role in nodule development [101–103]).

**Fig 3:**
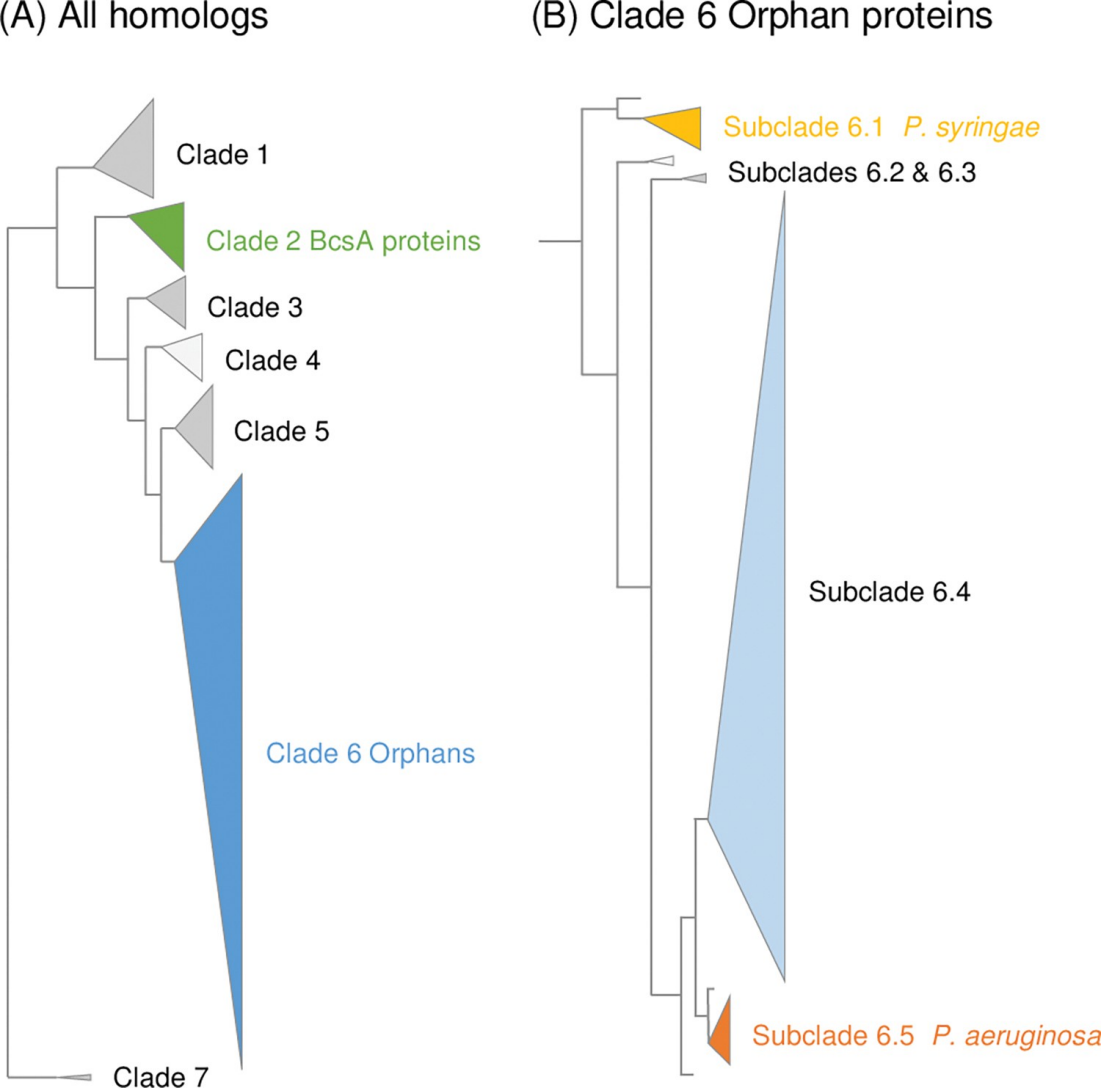
Orphan proteins are predominantly found within the *Pseudomonas* genus. Shown here are two schematics of an un-rooted UPGMA phylogenetic tree produced by Clustal Omega Simple Phylogeny [70] showing the seven main clades (**A**) and five subclades within Clade 6 that containing all *Pseudomonas* spp. Orphan proteins (**B**). These are simplifications of the original UPGMA phylogenetic tree of 190 Orphan protein homologs (see **S2 Fig** for the full tree; see **S1 File** for protein sequences) and are drawn with real (relative) genetic distances. *Clades and Subclades*: **Clade 1,** This clade includes the fungal *Rhizomucor miehei* CAU432 β-(1,3)-Glucanosyltransferase, *Rm* Bgt17A and contains a total of 20 proteins from fungi, plants, and Gammaproteobacteria with glucosidase, glucanosyltransferase, glycogen synthase, and mannosyltransferase annotations. **Clade 2,** This clade includes the *Escherichia coli* MG1655 and *Rhodobacter sphaeroides* 2.4.1 BcsA reference proteins and contains a total of 14 BcsA cellulose synthase proteins from the Alphaproteobacteria and Gammaproteobacteria. **Clade 3,** This clade contains 11 proteins from the Alphaproteobacteria and Epsilonproteobacteria with glucosyl/glycosyl transferase and glucanase annotations. **Clade 4,** This clade contains 10 proteins from the Betaproteobacteria and Gammaproteobacteria with benzoate transporter, glucanase, glucosyl transferase, and glycosyl hydrolase family annotations. **Clade 5,** This clade contains 17 proteins from the Alphaproteobacteria, Deltaproteobacteria, and Gammaproteobacteria, with glucanase, glycosyltransferase, and cellulose synthase annotations. **Clade 6,** This clade contains 112 representative *Pseudomonas* spp. Orphan proteins and four non-pseudomonad homologs in the following five subclades. **Subclade 6.1,** This subclade contains six *P*. *syringae* strain Orphan proteins including *Ps*DC3000, with glucosyl/glycosyl transferase annotations. **Subclade 6.2,** This subclade contains two non-*Pseudomonas* spp. Orphan proteins from the Betaproteobacteria and Gammaproteobacteria with gluco/glycosyl transferase annotations. **Subclade 6.3,** This subclade contains two non-*Pseudomonas* spp. Orphan proteins from the Deltaproteobacteria and Gammaproteobacteria with glucanase annotations. **Subclade 6.4,** This subclade contains a total of 94 *Pseudomonas* spp. Orphan proteins, including *P*. *fluorescens* SBW25 and *P*. *putida* KT2440 and excluding all *P*. *aeruginosa* and *P*. *syringae* Orphans, with glucanase, glucan biosynthesis protein, glucosyl/glycosyl transferase, Glyco trans 2-like domain-containing protein, and cellulose synthase annotations. **Subclade 6.5,** This subclade contains eight *P*. *aeruginosa* strain Orphan proteins including *Pa*PA01 and one additional *Pseudomonas* spp. Orphan, with glucanase, glycosyl transferase, and synthases of periplasmic glucan annotations. The unmarked nodes in (**B**) represent single *Pseudomonas* spp. Orphan proteins are not included in the subclades. **Clade 7,** This clade includes the Alphaproteobacteria bacterium *Rhizobium meliloti* 1021 NdvB and the fungal *Schizosaccharomyces pombe* 972 Ags1 proteins chosen as outliers for this tree.

The UPGMA tree we constructed consisted of seven clades (**Fig 3A**), placing plant and fungal homologs in Clade 1, BcsA proteins in Clade 2, and the Orphan proteins in Clades 3–6, with *Sp*Ags1 and *Rm*NdvB in Clade 7 (**Fig 3A;** see **S2 Fig** for the full tree listing all proteins and **Table 1A** for clade characteristics). We note that in the case of the Clade 3–5 representatives, *Arcobacter butzleri* ED-1, *Azoarcus* strain DN11 and *Methylomonas methanica* MC09, the *Orphan–dapE* gene synteny seen in our earlier analysis of key pseudomonads was not retained, perhaps because of ancient genome rearrangements and further supporting the lack of functional linkage between DapE and the Orphan protein. In contrast, the gene synteny was also retained by the Clade 6 representative, *Pseudomonas viridiflava* LMCA8, and by the other pseudomonad members of the Clade as this was part of the criteria used to select Orphans from PseudoCAP. However, Clade 6 also includes two small subclades which include four Orphans from other genera (Subclades 6.2 and 6.3), and in each case the gene synteny is not conserved. This suggests that the *Orphan–dapE* gene synteny is restricted to the pseudomonads and may reflect a relatively more recent genome rearrangement that brought the two genes together early in the development of the genus.

**Table 1:** Clade and subclade characteristics.

		*UPGMA Tree*	*Clustal Omega multiple sequence alignment*			
(A) Clades^a^		*Genetic distance* ^*b*^	*Coverage* ^*c*^	*Identity* ^*d*^	*Length* ^*e*^	*INDELS* ^*f*^
1	Plant and fungal glucanosyltransferases (*Oryza sativa*) (20)	0–0.47	43.3–90.9	5.2–52.6	1097	0.046
2	Cellulose synthase BcsA proteins (*Rhodobacter sphaeroides* 2.4.1) (14)	0.0007–0.74	86.9–92.3	13.9–28.2	1820	0.016
3	Non-*Pseudomonas* spp. Orphans (*Arcobacter butzleri* ED-1) (11)	0.0006–0.32	99.4–99.5	37.2–98.6	954	0.018
4	Non-*Pseudomonas* spp. Orphans (*Azoarcus* strain DN11) (10)	0.003–0.26	88.9–92.4	39.1–77.0	988	0.130
5	Non-*Pseudomonas* spp. Orphans (*Methylomonas methanica* MC09) (17)	0.0005–0.39	90.4–98.5	37.2–79.2	1031	0.014
6	*Pseudomonas* spp. Orphans (*Pseudomonas viridiflava* LMCA8) (116)^g^	0–0.33	97.4–99.8	39.4–59.2	923	0.016
7	Outliers (*Rhizobium meliloti* 1021) (2)^h^	1.24	72.1	12.9	3199	0.048
**(B) Clade 6 Subclades** ^**a**^						
6.1	*P*. *syringae* Orphans (*Pseudomonas syringae* ICMP 9617) (6)	0.018–0.30	99.3–100	59.0–63.2	842	0.004
6.2	Non-*Pseudomonas* spp. Orphans (*Limnobacter* strain 130) (2)	0.31	99.2	57.1	871	0.002
6.3	Non-*Pseudomonas* spp. Orphans (*Desulfofustis glycolicus* DSM 9705) (2)	0.33	99.4	55.5	880	0.003
6.4	*Pseudomonas* spp. Orphans (*Pseudomonas balearica* DSM 6083) (94)	0–0.17	97.4–100	74.4–76.9	892	0.008
6.5	*P*. *aeruginosa* Orphans (*Pseudomonas aeruginosa* PA14) (9)^i^	0–0.01	100	99.2–99.8	869	0.000

Orphan proteins and homologs were placed into seven clades (1–7) in the UPGMA tree shown in **Fig 3** and **S2 Fig**. Clustal Omega multiple sequence alignments were used to further characterise each clade or subclade.

^**a**^ The reference protein used in each multiple sequence alignment is provided in parentheses followed by the number of proteins in each clade or subclade.

^**b**^ Minimum–maximum genetic distances between proteins in the same clade.

^**c**^ Coverage of individual Orphan sequences in the multiple sequence alignment.

^**d**^ Sequence identities (%) in the multiple sequence alignment.

^**e**^ Length of the multiple sequence alignment.

^**f**^ Number of INDELS normalised for multiple sequence alignment length.

^**g**^ This clade contains four non-*Pseudomonas* spp. Orphans. Three *Pseudomonas* Orphans listed in this clade are not included in subclades 6.1–6.5.

^**h**^ Two reference proteins (*R*. *meliloti* 1021 NdvB and *Schizosaccharomyces pombe* 972 Ags1) that had very limited homology with the Orphan proteins were used as outliers for the UPGMA tree.

^**i**^ This subclade contains one non-*P*. *aeruginosa* strain Orphan.

All *Pseudomonas* spp. Orphan proteins were located in Clade 6 which we suggest is the true Orphan family (we recognise that this is oxymoronic) and these proteins are distributed across five subclades (**Fig 3B** and **Table 1B**). *P*. *aeruginosa* and *P*. *syringae* Orphan proteins formed separate subclades (Subclades 6.5 and 6.1, respectively), but most pseudomonad Orphan proteins were in a large and unstructured subclade (Subclade 6.4) and included proteins from *P*. *fluorescens* and *P*. *putida*, as well as some other plant pathogens. This suggests that these proteins may have a relatively recent ancestor or that environmental / host adaptation may not be particularly strong, except for *P*. *aeruginosa* and *P*. *syringae* where host adaptation might be selecting for divergent sequences. A multiple sequence alignment of *P*. *aeruginosa*, *P*. *fluorescens*, *P*. *putida* and *P*. *syringae* Orphan proteins grouped sequences by species, with the *P*. *syringae* proteins the most divergent and including a large 14 residue insertion not seen in the other proteins (see **S3 File** for an annotated multiple sequence alignment of 26 *Pseudomonas* spp. Orphan proteins showing domains, conserved motifs, and residues). Further analysis involving more Orphans may confirm the pseudomonad family as the dominant clade, but this may change as genome sequences become available for under-represented sister and more distant genera.

Our larger comparison of Orphan homologs confirms their distant relationship to BcsA proteins and other orthologs. The poor level of amino acid sequence conservation in the central core region of the Orphan proteins suggests that they are unlikely to function as additional or alternate BcsA subunits in the cellulose synthase holoenzyme and are more likely to be involved in the synthesis or modification of some other polysaccharide. This is supported by the identification of more general glycosyltransferase (GT) annotations provided for Orphan proteins in PseudoCAP, though this is not particularly informative as GT proteins are highly diverse with 114 families currently listed by the Carbohydrate Active Enzymes Database (crazy.org); for example, the GT2 family is also highly diverse and includes sixteen distinct enzymatic activities including cellulose synthase [25]. The sequence divergence within the Orphan clade also suggests that this family of proteins may provide an adaptative advantage in different environments with continued adaptation and altered enzymatic activities.

### Conserved domains and structural comparison by homology modelling suggest a two-domain structure and function for the Orphan proteins

**Fig 4:**
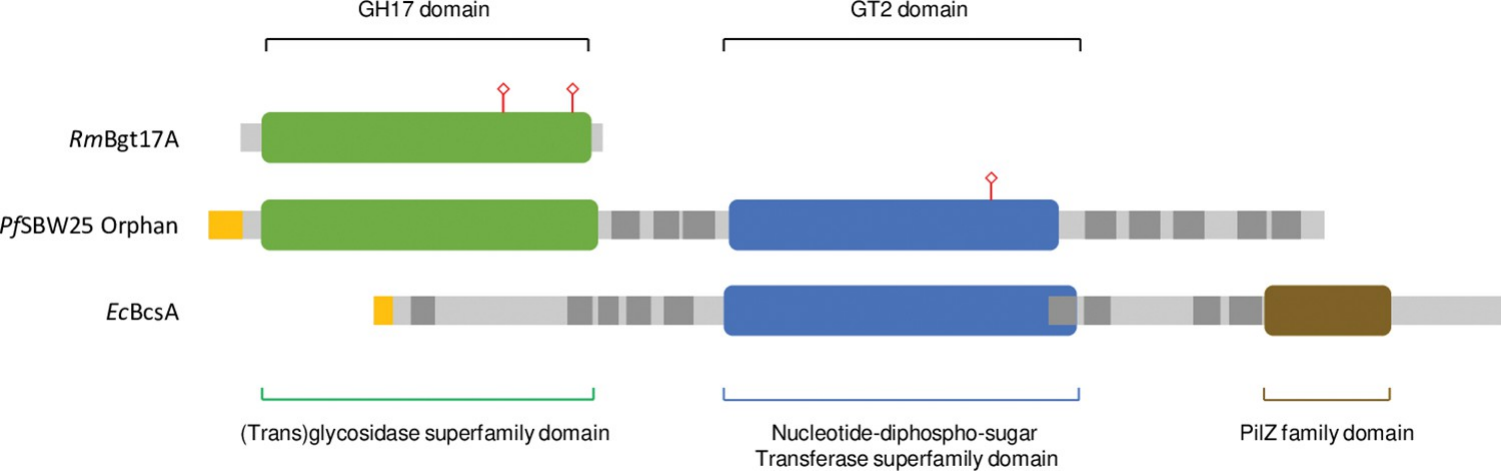
Functional domains identified in the Orphan protein sequence by HMMER. Shown here are schematics of the fungal *Rhizomucor miehei* CAU432 β-(1,3)-Glucanosyltransferase, the *Pseudomonas fluorescens* SBW25 Orphan protein, and the *Escherichia coli* MG1665 cellulose synthase catalytic BcsA subunit, aligned to show the positioning of the (first) GH17 (trans)glycosidase and (second) GT2 nucleotide-diphospho-sugar transferase domains identified by HMMSCAN [75]. The *Pf*SBW25 Orphan protein includes a peptide signal sequence (yellow) and a series of transmembrane helices (dark grey), but not the regulatory PilZ domain (Pfam 07238) present in BcsA. HMMSCAN also identified catalytic residues (red marker) but not in both homologous domains. The first domain of the *Pf*SBW25, *P*. *putida* KT2440 and *P*. *syringae* DC3000 Orphan proteins share significant homology with the (Trans)glycosidase superfamily / β-Glucanases family (Superfam 51445 and 51487; Conditional E-values of 1.3e-45–5.4e-57) and Glycosyl hydrolase family 17 (Pfam 00332; 2.0e-07–2.7e-8). The second domain shared significant homology with the NDP-sugar-transferase superfamily (Superfam 53448; 2.4e-50–1.2e-52) and Glycosyltransferase-like family 2 (Pfam PF13641; 1.1e-27–1.0e-34).

We used HMMSCAN to identify conserved domains in the *Pf*SBW25 Orphan protein using profile Hidden Markov Models [75] (**Fig 4**). This suggested a two-domain model corresponding to the upstream and over-lap regions seen in our earlier pairwise and multiple sequence alignments. Similar HMMSCAN results were obtained for *P*. *aeruginosa* PA01, *P*. *putida* KT2440, and *P*. *syringae* DC3000 Orphan proteins, and these plus the high degree of amino acid sequence identity seen between all Orphan proteins examined (e.g., see **S3 File** for the multiple sequence alignment of 25 Pseudomonas spp. Orphan proteins), also provides evidence for an Orphan ‘protein’ family which complements our earlier presentation of the Orphan ‘phylogenetic’ family.

HMMSCAN, Proteus2 [79], and Protter [80] identified a signal peptide sequence (residues 1–25) in the *Pf*SBW25 Orphan protein sequence, and the Protter protein topology prediction suggested a N-terminal periplasmic domain (residues 1–311) and a central cytoplasmic domain (residues 392–680) linked by two transmembrane (TM) regions (residues 312–391 with three TM helices, and residues 681–847 with five TM helices).

The first *Pf*SBW25 Orphan domain (residues 26–311) included matches to transglycosidase / glycosyl hydrolase (GH) domains (Superfamily 51445, Family 51521 & 51487, and Protein family (Pfam) 00332), and we suggest that this domain is involved in the sequential hydrolysis and rearrangement of a β-glucans producing elongated, branched, or cyclised structures, in agreement with earlier enzymatic characterisation studies and recent molecular simulations [48, 49]. To avoid confusion, we now refer to this region of the Orphan protein as the GH17 domain.

The second *Pf*SBW25 Orphan domain (residues 312–847) included matches to nucleotide-diphospho-sugar transferase / glycosyltransferase domains (Superfamily 53448; Pfam 13641) and we therefore propose that is a GT2 family glycosyltransferase involved in the processive addition of glycosyl subunits from UDP-hexose to an elongating glucan polymer. This part of the Orphan protein also included the two TM regions (residues 312–391 and 681–847) which are commonly found either side of the GT2 domain in BcsA-like synthases [25, 30, 31]. We therefore refer to these regions of the Orphan protein as the TM region / GT2 domain.

We used Phyre^2^ [85] and SWISS-MODEL [87, 88] to produce template-based models of the GH17 domain and the TM region and GT2 domain of the *Pf*SBW25 Orphan protein (all models are available as PDB files, see **S2 File**). Both Phyre^2^ and SWISS-MODEL identified the fungal *Rhizomucor miehei* CAU432 β-(1,3)-glucanosyltransferase Bgt17A (*Rm*Bgt17A) structure [104] (UniProtKB: A0A0M3KKZ6; Protein Databank (PDB) 4WTP) as the best template for the GH17 domain (Phyre^2^, mean ProQ2 score of 0.23 for residues; SWISS–MODEL, QMEANDisCo score of 0.50 for the model). However, pairwise alignment of the *Pf*SBW25 Orphan GH17 domain (residues 37–306) and *Rm*Bgt17A showed that they shared only 26.9% sequence identity and 53.1% similarity, respectively.

Template-based modelling also identified the *Rhodobacter sphaeroides* 2.4.1 BcsA (*Rs*BcsA) structure [32, 33] (UniProtKB: A0A3G6W9S6) as the best template to model the TM region / GT2 domain (Phyre^2^, PDP 4HG6 template [32], mean ProQ2 score of 0.39 for residues; SWISS–MODEL, PDP 4P00 and 4P02 [33] templates, QMEANDisCo scores of 0.49 and 0.51 for each model). No corresponding structure was available for our reference protein, *Ec*BcsA, but *Rs*BcsA (residues 13–740) and the *Pf*SBW25 Orphan TM region / GT2 domain (residues 314–860) shared 24.4% sequence identity and 38.4% similarity, respectively.

We used Pairwise Structure Alignments [94] and the FATCAT-rigid algorithm [95, 96] to compare single-domain models and the template structures. Despite the low pairwise sequence identities between the *Pf*SBW25 Orphan protein and the template sequences, the RMSD (0.55–2.15) and TM-scores (0.57–0.94) indicate that these models and structures were very similar (**Table 2A**). To put this into context, most single-domain proteins can be folded onto the best homologous template with an overall average RMSD of 2.3 and high-quality models can be produced with TM-scores above 0.8 when there is better than 40% amino acid sequence identity with the template [105] (notwithstanding the fact that model quality is dependent on the quality of the template). However, differences between models were evident at the local scale when comparing the positioning of the start and ends of secondary structure features along the primary amino acid sequence. Although each model used the same protein sequence and structural templates, there were only four starts and three ends conserved in eight reference α-helices compared in the first domain, and seven starts and five ends conserved in 14 TM helices, α-helices, and β-sheets compared in the second domain (see **S3 Table** for our comparison of secondary structures), perhaps reflecting the problems caused by adjusting to five INDELS needed to align with *Rm*Bgt17A and over 20 INDELS needed to align with *Rs*BcsA.

**Table 2:** Pairwise comparisons of protein structures and models.

			FATCAT-Rigid Body Comparison				
			Coverage (%) ^a^	RMSD ^b^	TM-Score^c^	FATCAT Score ^d^	Length ^e^
(A) Comparisons between *Rm*Bgt17A and *Rs*BcsA protein structures and *Pf*SBW25 Orphan single-domain models ^f^^aa6^							
*Rm*Bgt17A		Phyre^2^ GH17 (37–306)	97 / 95	0.80	0.94	658.66	257
*Rm*Bgt17A		SWISS-MODEL GH17 (36–305)	96 / 95	0.55	0.94	638.62	256
*Rs*BcsA (13–740)		Phyre^2^ TM & GT2 (314–856)	72 / 96	1.36	0.70	1315.47	521
*Rs*BcsA (13–740)		SWISS-MODEL TM & GT2 (416–855)	57 / 95	0.95	0.57	1101.61	417
Phyre^2^ GH17 (37–306)		SWISS-MODEL GH17 (36–305)	99 / 99	1.59	0.94	660.94	265
Phyre^2^ TM & GT2 (314–864)		SWISS-MODEL TM & GT2 (416–855)	98 / 98	2.15	0.93	1014.50	430
**(B) Comparisons between the AlphaFold *Pf*SBW25 Orphan model, *Rm*Bgt17A and *Rs*BcsA protein structures, and *Pf*SBW25 Orphan single-domain models** ^f^							
AlphaFold		*Rm*Bgt17A	26 / 97	1.99	0.25	540.84	225
AlphaFold		*Rs*BcsA (13–740)	55 / 65	3.10	0.51	1092.45	476
AlphaFold (37–306)		Phyre^2^ GH17 (37–306)	100 / 100	3.09	0.88	632.07	270
AlphaFold (316–858)		Phyre^2^ TM & GT2 (317–859)	86 / 86	3.17	0.77	1053.84	468
AlphaFold (36–305)		SWISS-MODEL GH17 (36–305)	100 / 100	2.63	0.90	666.81	270
AlphaFold (416–855)		SWISS-MODEL TM & GT2 (416–855)	95 / 95	3.05	0.84	929.83	417
**(C) Comparisons between different *Pf*SBW25 Orphan models**							
AlphaFold		InterFOLD	83 / 83	16.41	0.33	1675.9	716
AlphaFold		RoseTTAFOLD	98 / 98	17.31	0.37	2316.53	845
AlphaFold		TrRosetta	94 / 94	9.65	0.62	2119.71	808
InterFOLD		RoseTTAFOLD	86 / 86	14.78	0.41	1737.10	738
InterFOLD		TrRosetta	85 / 85	14.59	0.37	1651.51	731
TrRosetta		RoseTTAFOLD	99 / 99	18.05	0.38	2185.13	853
(**D) Comparisons between the AlphaFold *Pf*SBW25 Orphan model and AlphaFold models of other Orphan proteins** ^g^							
*Pf* SBW25		*Arcobacter butzleri* ED-1 (Clade 3*)	96 / 99	2.05	0.92	2146.08	826
*Pf* SBW25		*Azoarcus strain* DN11 (Clade 4*)	99 / 90	2.62	0.85	2297.55	855
*Pf* SBW25		*Methylomonas methanica* MC09 (Clade 5*)	100 / 90	1.86	0.88	2359.44	856
*Pf* SBW25		*Pseudomonas aeruginosa* PA01 (Clade 6.5)	100 / 99	0.92	0.98	2523.12	860
*Pf* SBW25		*Pseudomonas putida* KT2440 (Clade 6.4)	99 / 98	0.59	0.98	2519.35	850
*Pf* SBW25		*Pseudomonas syringae* DC3000 (Clade 6.1)	80 / 78	19.43	0.27	2200.68	671
*Pf* SBW25		*Pseudomonas viridiflava* LMCA8 (Clade 6*)	78 / 80	19.58	0.28	2214.96	667

Protein structures, single-domain and prediction models were quantitatively compared using Pairwise Structure Alignment and FATCAT-rigid body comparisons. Note that Phyre^2^ and SWISS-MODEL homology models and some comparisons used restricted sections of the *Pf*SBW25 Orphan protein.

^**a**^ Fraction of residues matched by the superposition relative to the number of aligned modelled residues.

^**b**^ RMSD (root mean square deviation) is calculated between aligned pairs of the backbone Cα atoms in the superposition of the two structures (smaller the better) but it is sensitive to local structure differences.

^**c**^ Template modelling score (TM-Score) is a measure of the topological similarity between structures (0 [no match]– 1 [perfect match]) and is less sensitive to local differences. A score of 0.2 or below suggest that the two structures are unrelated; 0.5 or above suggest that they have the same fold.

^**d**^ FATCAT score is a measure of structural similarity specific to this algorithm depending on the length of aligned fragment pairs and distance cut-offs (lower the better).

^**e**^ Number of residue pairs that are structurally equivalent in the alignment of the structures (higher the better, maximum of 860 for the *Pf*SBW25 Orphan protein).

^**f**^ Single-domain *Pf*SBW25 Orphan models are of GH17 domain and the TM region and GT2 domain (TM & GT2); residue numbers in parentheses indicate those included in each model.

^**g**^ UPGMA tree clades and subclades shown in **Fig 3** and **S2 Fig** are indicated in parenthesises with Clade representatives (**Table 2**) indicated by the asterisk (*Pf*SBW25 Orphan is in Clade 6.4).

#### The first Orphan domain adopts a TIM-barrel–like structure and active cleft seen in fungal β-glucosyltransferases

The 278-residue *Rm*Bgt17A protein produces branched glucans from linear β-(1,3)-glucan during fungal cell-wall assembly and rearrangement and is a single domain (GH17) protein with a classical (α/β) TIM-barrel fold [104]. In *Rm*Bgt17A and related fungal glucosyltransferases, two sub-domains (SD1 & 2) and two catalytic glutamic acids in the conserved motifs (VGxEV and ExGWPx) have been identified previously, though *Rm*Bgt17A has a shorter catalytic cleft located on the rim of the TIM-barrel compared to other proteins [104]. *Rm*Bgt17A has β-(1,3)-glucanase and β-(1,3)-glucanosyltransferase activities producing elongated and branched β-glucans [106]. Residues 305–269 of the *Rm*Bgt17A protein sequence aligned to the *Pf*SBW25 Orphan GH17 domain (positions 60–299) with a 50.6% sequence similarity and there was a matching pattern of α-helices and β-sheets as predicted by Proteus2. We confirmed the similarity with *Rm*Bgt17A by identifying corresponding TIM-barrel α-helices in the Phyre^2^ and SWISS-MODEL single-domain models with those in the *Rm*Bgt17A template (**S3 Table**) and by FATCAT comparisons (**Table 2A**). However, although the *Rm*Bgt17A sub-domain SD1 α-helix–β-sheet and SD2 β-sheet–loop sequences were not found in the *Pa*PA01, *Pf*SBW25, *Pp*KT2440 and *Ps*DC3000 Orphan proteins (**Table 3**), the corresponding regions had similar predicted secondary structures. The corresponding SD1 sequence was more highly conserved than the SD2 sequence in an alignment of a larger set of Orphan homologs (**S3 File**), suggesting a structural importance for these sub-domains.

**Table 3:** Conserved motifs and residues identified in the Orphan proteins.

**(A) Motifs associated with *Rm*Bgt17A**		*SBW25 Orphan*
VG(S/N)EV	Conserved motif seen in a multiple sequence alignment of *Rm*Bgt17A and related proteins including the proton donor catalytic residue (E).	VGNEL
E(T/S)GWP(T/S)	Conserved motif seen in a multiple sequence alignment of *Rm*Bgt17A and related proteins including the nucleophile catalytic residue (E).	EVGWPS
KELVAPHGI	Subdomain (SD1) recognised in other proteins related to *Rm*Bgt17A but significantly truncated in *Rm*Bgt17A.	RAAVKVPVT^a^
MMNAFPYEGV	Subdomain (SD2) recognised in other proteins related to *Rm*Bgt17A but significantly truncated in *Rm*Bgt17A.	AAHILPFWEYI^a^
**(B) Motifs associated with *Rs*BcsA**		*SBW25 Orphan*
DD, DxD, ED, Q(Q/R)xRW	Conserved motif common in processive β-glycosyl transferases including BcsA. These residues combine to form a GT-A fold and mediate substrate and acceptor-binding.	
	The first D in the conserved DD (or DDG) motif is involved in UDP-Glucose coordination with FxVTxK and HxKAG.	DNN
	The first D in the conserved DxD motif is the metal ion binding site and involved in UDP-Glucose coordination.	DSD
	D in the conserved ED (or TED) motif is the catalytic base located on the side of the substrate-binding pocket opposite HxKAG.	CED
	Q(Q/R)xRW (or QxxRW) forms the substrate and acceptor binding site with FFCGS. W coordinates the positioning of the terminal glucose unit of the cellulose polymer and forms part of the entrance to the transmembrane channel.	QRFRW
FFCGS	Forms the binding site for the terminal glucose of the cellulose polymer with Q(Q/R)xRW.	IQHGT
FxVTxK	Part of the gating loop that moves to allow UDP-Glucose access to the active site. Involved in UDP-Glucose coordination with DDG and HxKAG.	FFRTPK
HxKAG	Involved in UDP-Glucose coordination with DDG and FxVTxK [x].	GFKGG
QTPH	Involved in positioning the reducing end (acceptor) of the cellulose close to the catalytic base.	QSPQ
RxxxR	Involved in binding c-di-GMP and part of the PilZ regulatory domain.	(Not present)
DxSxxG	Involved in binding c-di-GMP and part of the PilZ regulatory domain.	(Not present)

For motifs associated with *Rm*Bgt17A see [104] and *Rs*BcsA see [25, 30–32, 34]. There is some minor variation in the reported sequences of conserved motifs associated with BcsA proteins (e.g., [30–32]).

^**a**^ Position mapped onto the Orphan sequence using an alignment with *Rm*BgtA17 but conservation is limited.

**Fig 5:**
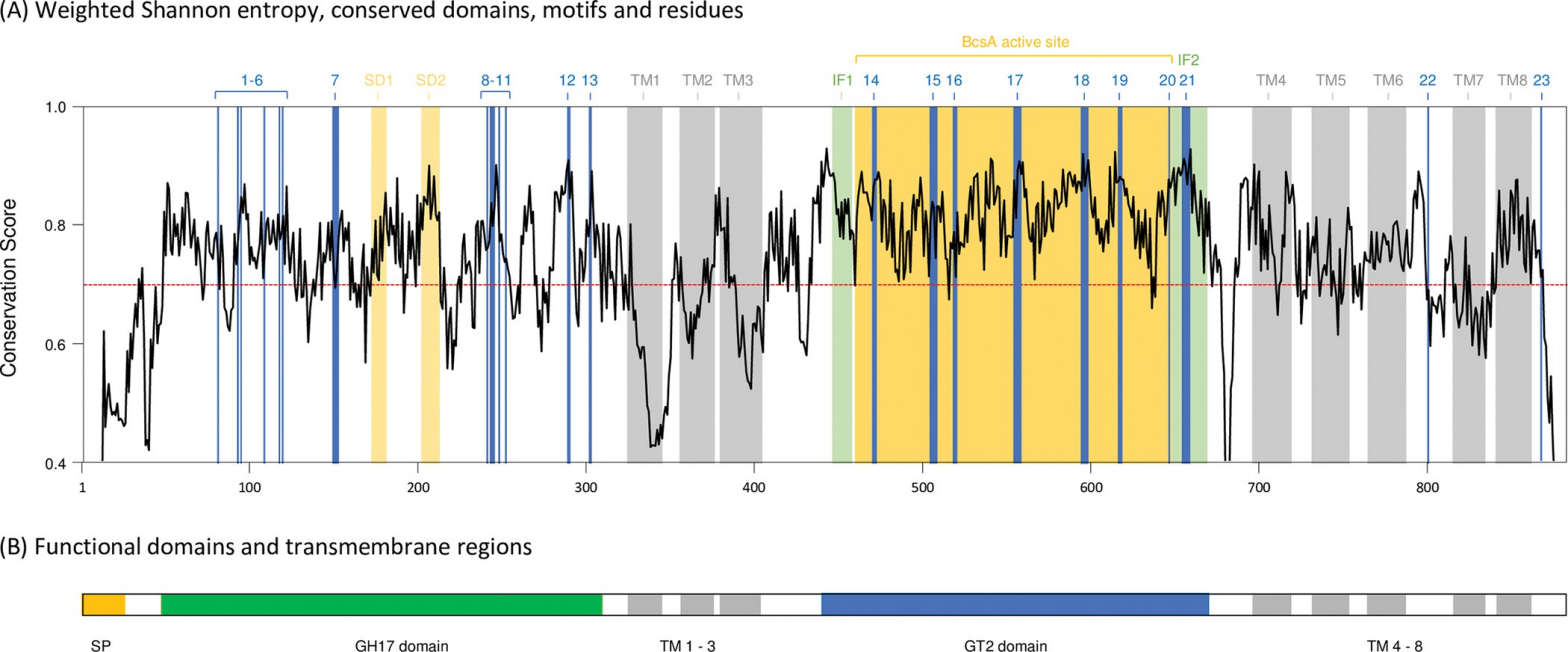
The Orphan proteins share conserved features found in GH17 glucanosyltransferases and GT2 cellulose synthases. Shown here is a map of amino acid conservation scores (**A**) determined by Shannon entropy [73] from a Clustal Omega multiple sequence alignment [70] of 26 Orphan proteins found in *Pseudomonas aeruginosa* AZPAE12140, BL14, PAK, PA01, PA14, LESB58, 19BR and 3573, *P*. *fluorescens* ICMP 11288, ICMP 3512, KF1, LMG 5329, SBW25, SS101, WH6 and WS 5037, *P*. *putida* KT2440, S610, W619 and YKD221, and *P*. *syringae* B728a, DC3000, ICMP 9617, NCPPB 4273, UMAF0158 and 41a strains (see **S1 File** for protein sequences and **S3 File** for an annotated multiple sequence alignment of these proteins). A value of 1 indicates complete conservation while lower values indicate less conservation of that residue. Overlaid onto this map are conserved domains, motifs, and residues, found in fungal GH17 β-(1,3)-glucanosyltransferases and *Rhizomucor miehei* CAU432 Bgt17A, as well as bacterial GT2 synthases such as *Rhodobacter sphaeroides* 2.4.1 BcsA, that are indicated by vertical blue lines and coloured rectangles. Below this is a simplified schematic of the Orphan protein (**B**) in which the proposed functional GH17 and GT2 domains are indicated along with the signal peptide sequence (yellow) (not identified in *P*. *syringae* strain Orphans) and transmembrane domains (grey). Note that the x-axis and Orphan schematic shown here are longer than individual Orphan proteins and the additional length is a result of the INDELS (mainly insertions) introduced by the multiple sequence alignment. *Fungal GH17 / RmBgt17A conserved domains*, *motifs*, *and residues*: **1,** Conserved D. **2,** Conserved R. **3,** Conserved Y. **4,** Conserved E. **5,** Conserved G. **6,** Conserved W. **7,** VGNE motif. **SD1 & SD2,** Sub-domains. **8,** Putative catalytic E. **9,** GWP catalytic site. **10,** Conserved G. **11,** Conserved G. **12,** Conserved WK. **13,** Conserved WG. Bacterial GT2 **/** *RsBcsA conserved domains*, *motifs*, *and residues*: **TM1 –TM3,** Transmembrane helices. **IF1,** Amphipathic interface helix 1. **BcsA Active Site,** Active site of GT2 synthases. **14,** DDG motif (but only D). **15,** HAKAG motif. **16,** DAD motif. **17,** QTPH motif. **18,** FFCGS motif (but only G). **19,** TED motif (but only ED). **20,** Conserved E not seen in the Orphans. **IF2,** Amphipathic interface helix 2. 21, QRxRW motif. **TM4 –TM6,** Transmembrane helices. **22,** Conserved T not seen in the Orphan proteins. **TM7 & TM8,** Transmembrane helices. **23,** RxxxR motif associated with c-di-GMP binding not seen in the Orphan proteins. Note some conserved motifs / residues not identified in the Orphan proteins are also indicated for reference.

The VGxEV and ExGWP motifs and conserved glutamic acid residues were recognised in the *Pf*SBW25 Orphan sequence, and in both Phyre^2^ and SWISS-MODEL single-domain models they were localised in an exposed cleft running across the bottom of the TIM-barrel like *Rm*Bgt17A [104]. Further comparison using Shannon entropy analysis [73] indicate that GH17 sub-domains, motifs, and conserved residues, are in regions of high conservation across Orphan proteins (**Fig 5**) and therefore may retain some GH17 / *Rm*Bgt17A-like functionality with the first major INDEL seen among Orphan homologs occurring after the end of the GH17 domain (**S3 File**).

We note that the GH17 domains of the *Pa*PA01 and *Pp*KT2440 Orphan proteins have been enzymatically characterised as fusion or tagged proteins (referred to as Glt1 and Glt3, respectively) that showed non-Leloir trans-β-glucosylation activity on linear β-(1,3)-glucan, cleaving short β-(1,3)-oligosaccharides and retaining the non-reducing end which is transferred to another acceptor glucan with a β-(1,3) linkage in an elongation reaction [48]. Glt1 and Glt3 template-based models were also created using the *Rm*Bgt17A template and the two catalytic glutamic acids within the active site cleft confirmed by superimposition of *Rm*Bgt17A [49]. Molecular dynamics simulations using these models demonstrated that Glt1 and Glt3 could bind β-(1,3)-glucan with the major cleavage site occurring at the third or fourth β-(1,3) linkage.

#### The second Orphan domain includes a Rossmann-like fold and a BscA-like active site positioned at the base of twisted column of transmembrane helices

The *Rs*BcsA catalytic cellulose synthase sub-unit contains a transmembrane (TM) region formed by a twisted cylinder of eight transmembrane helices that cross the inner membrane in a truncated cone of twisted cylinders, with a large intracellular Leloir (sugar-nucleotide-dependent) GT2 domain linked to a six-stranded β-barrel PilZ domain by a curved α-helical region [25, 30, 31, 34]. The domain contains a α/β/α sandwich Rossmann-like fold that includes seven β-sheets arranged in a 3214657 topology in which β-sheet 6 is antiparallel to the others [18]. The domain also contains the Q(Q/R)xRW motif which forms part of the active site and is on amphipathic interface helix IF2 located perpendicular to the base of the transmembrane helices on the cytoplasmic surface of the inner membrane. The first aspartate in each of the conserved DD and DxD motifs are involved in the coordination of the UDP-Glucose substrate while the aspartate in the ED motif is the catalytic base [32]. The DD, DxD, ED and Q(Q/R)xRW motifs form the active site where they mediate substrate (UDP-Glucose) and acceptor (non-reducing end of the glucan chain) binding. This is positioned just below a narrow transmembrane channel formed by six transmembrane helices (TM3–8) [32] and an additional TM helix is provided by the accessory membrane-anchored *Rs*BcsB protein which is required for BcsA activity [107]. The regulatory PilZ domain includes the RxxxR and DxSxxG c-*di*-GMP-associated motifs [34]. When bound there is a conformational change in a gating loop allowing UDP-Glucose to bind to the active site (the gating loop also contains the conserved FxVTxK motif) [33, 108]. The glucosyl unit is transferred to the non-reducing end of the cellulose polymer with a β-(1,4) linkage and the elongating polymer is translocated up through the transmembrane channel to the periplasm and the *Rs*BcsC porin located in the outer membrane [25, 30, 31, 109].

Residues 15–566 of the *Rs*BcsA protein aligned to the *Pf*SBW25 Orphan protein TM region / GT2 domain (residues 305–813) with a 37.7% normalised similarity and there was a matching pattern of TM helices, α-helices, and β-sheets, in *Rs*BcsA and the *Pf*SBW25 Orphan as predicted by Proteus2. We confirmed the similarity with *Rs*BcsA by identifying corresponding TM helices, Rossmann-like fold β-sheets, and IF2 / Q(Q/R)xRW in the Phyre^2^ and SWISS-MODEL models (**S3 Table**) and by FATCAT comparisons (**Table 2A**).

However, the Phyre^2^ and SWISS-MODEL coverage of the *Rs*BcsA structure differed, with Phyre^2^ modelling residues 314–856 that including the TM helices on either side of the GT2 domain, and SWISS-MODEL a shorter sequence covering residues 416–855 that missed out the TM helices encoded before the GT2 domain (**S3 Table**). Differences were seen in the positioning of the start and ends of TM helices, α-helices, and β-sheets along the primary amino acid sequence with SWISS-MODEL predicting a significantly shorter TM5, and both models presenting TM6 predicted by both HMMSCAN and Proteus2 as a normal α-helix located perpendicular to the base of the TM region. Despite these differences, Shannon entropy analysis indicate that GT2 motifs and conserved residues in the active site are in regions of high conservation across Orphan proteins (**Fig 5**), suggesting that this domain may retain some GT2 / BcsA-like functionality. Poor levels of structural conservation with *Rs*BcsA were seen in the transmembrane helices TM1–3, just before TM4, and in TM7, though the only major INDEL among Orphan homologs in the TM region and GT2 domain in fact occurs just after IF2 / Q(Q/R)xRW outside the BcsA-like active site. It is noteworthy that in a larger alignment of GT-A Rossmann-like folds, the Q(Q/R)xxRW motif is located on a hypervariable region (HV3) rather than on a conserved α-helix (IF2 in BcsA proteins), β-sheet, or loop structure, despite the enzymatic importance of this sequence [23].

### Structural predictions of the Orphan protein suggest a transmembrane ovoid-like protein with a periplasmic GH17 domain and a cytoplasmic GT2 domain

While template-based homology modelling of the *Pf*SBW25 Orphan protein by Phyre^2^ and SWISS-MODEL and comparisons of these with the *Rm*Bgt17A and *Rs*BcsA proteins have provided some insight into the likely function of the Orphan protein. However, these models are biased by the need to align the *Pf*SBW25 sequence to the X-ray structures, and neither Phyre^2^ or SWISS-MODEL were able to confirm the Protter protein topology prediction by producing a combined structure linking the GH17 and TM region / GT2 domains. We decided to explore different predicted structures produced using AI / Deep Learning-based free modelling that allow structures to be predicted beyond the limits of available templates [35–37]. The limited sequence homology seen between *Rm*Bgt17A and the GH17 domain (26.1% sequence identity) and between *Rs*BcsA and the TM region / GT2 domain (23.8%) suggest that the *Pf*SBW25 Orphan protein is in the ‘twilight’ modelling category [16] and it is possible that free modelling might produce significantly different domain structures as well as a combined GH17 / TM region / GT2 domain structure stabilised by tertiary interactions. However, we would expect to see consistency across predictions allowing a consensus structure to be produced in agreement with our earlier secondary structure and protein topology predictions.

We submitted the *Pf*SBW25 Orphan sequence initially to AlphaFold Colab Notebook [82, 83], and then to InterFOLD6 [84], RoseTTAFold [86] and transform-restrained Rosetta (TrRosetta) [89–91] for comparison and discuss here the top-ranked predicted models they produced (**S2 File**). In this modelling, we chose to retain the signal peptide identified in the *Pf*SBW25 Orphan protein, as in other Orphans it was not clear whether a signal sequence and cleavage site was present or not (see our test of the significance of the signal sequence on the AlphaFold *Pf*SBW25 predicted structure which is described later). To assess these models, we visually compared structures to determine the relative positioning and orientation of the G17 domain, TM region and GT2 domain and compared the start/stop positions of the reference TM helices, α-helices, and β-sheets we used to assess our single-domain models. We complemented this using Pairwise Structure Alignments and compared FATCAT-rigid RMSD and TM-score values to assess global differences in the positioning of the Cα backbone and similarities in protein topologies (due to the number of possible pairwise combinations we focussed on comparisons with the AlphaFold predicted structure).

#### The AlphaFold predicted PfSBW25 structure reveals the association between the GH17 domain, transmembrane region, and GT2 domain

**Fig 6:**
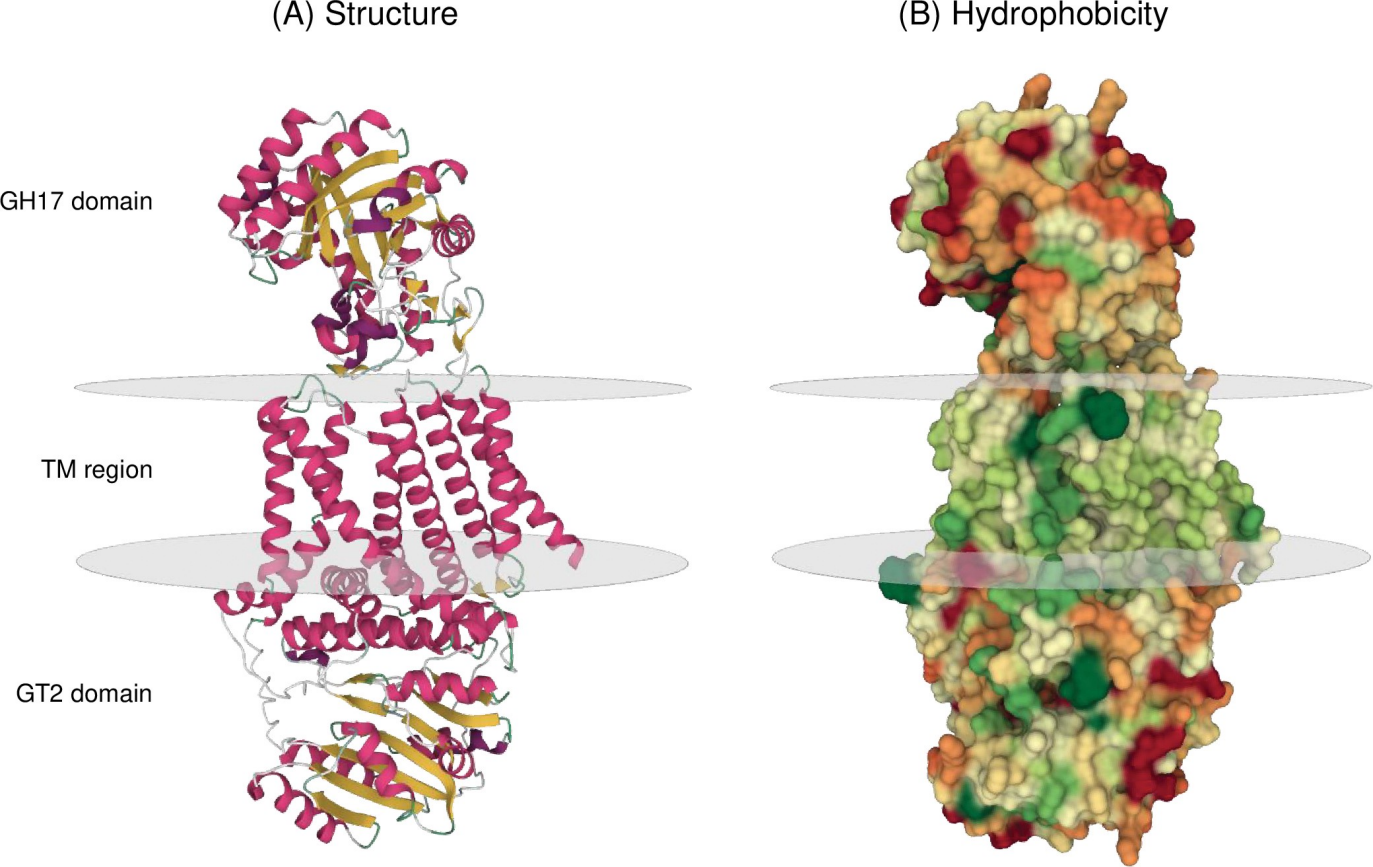
Two-domain structural prediction of the *Pf*SBW25 Orphan protein. Shown here is the AlphaFold predicted structure of the *Pseudomonas fluorescens* SBW25 Orphan protein showing the relative positioning of the GH17 domain, transmembrane (TM) region and GT2 domain. The transmembrane ovoid-like structure of the protein (**A**) is dominated by α-helices (magenta) in the TM region with some β-sheets (gold) found in the GH17 TIM-barrel and GT2 Rossmann-like fold. Loops (green) (sections with poor certainty are in light green and white) are also indicated. Surface hydrophobicity (**B**) is represented by cold colours with hydrophilic surfaces indicated by warmer colours. The model was produced by AlphaFold Colab Notebook [82, 83] and the PDB file is available (see **S2 File**). The model was visualised with Mol* 3D Viewer [92] using molecular surface and membrane orientation representations and colouring residues according to secondary structure or hydrophobicity.

The *Pf*SBW25 Orphan model is a transmembrane ovoid-like structure with the GH17 domain in the periplasm and the GT2 domain in the cytoplasm (**Fig 6**) (it should be noted that Mol* was used to visualize the membrane using the ANVIL algorithm [93] rather than it being modelled by AlphaFold; ANVIL positions membranes based on amino acid hydrophobicity and the likelihood that these residues are embedded in membranes). The GH17 domain is dominated by the TIM-barrel with the GH17 active site cleft running horizontally across the bottom of the structure and immediately above the top of the TM region formed by a twisted cylinder of transmembrane helices and the α-helix signal peptide (see **S3 Fig** for a schematic identifying each of the TM helices). The GT2 domain appears to be compressed against the bottom of the TM region, with several α-helices including IF2 lying perpendicular to the transmembrane helices and the seven β-sheets of the Rossmann-like fold located further away from the membrane surface. In addition to the TIM-barrel and the Rossmann-like fold, we were able to identify the α-helices and transmembrane helices we had seen in *Rm*Bgt17A and *Rs*BcsA, and in the Orphan single-domain models (**S3 Table**), with the AlphaFold model having similar variation in secondary structure start and stop positions as SWISS-MODEL compared to the Pyre^2^ reference models.

Surface representations of the model suggested substantive interactions between the GH17 domain and TM region though they were only linked by one unstructured sequence from the end of the last α-helix identified in the TIM-barrel to the start of TM1 (residues 283–311). We identified six non-covalent interactions between the GH17 domain, unstructured sequence and the TM region that might help stabilise the positioning of the GH17 domain against the periplasmic face of the TM region (see **S4 Table** for a list of non-covalent interactions). The signal peptide, shown as a long α-helix and associated with the TM region, is unlikely to have any impact on the prediction, as AlphaFold produced an almost identical structure for a truncated *Pf*SBW25 Orphan protein lacking this sequence (FATCAT-rigid RMSD 0.69, TM-score 0.96, and Score 2468.03) (**S2 File**).

Pairwise Structure Alignments with the *Rm*Bgt17A and *Rs*BcsA structures and single-domain models produced low RMSD values of 1.99–3.17 (**Table 2B**) reflecting the relative ease of predicting single-fold structures and confirming the AlphaFold predictions of the GH17 and GT2 domains (AlphaFold was trained to produce predicted structures most likely to appear in protein database structures [82]). We obtained a low TM-Score in the comparison with the *Rm*Bgt17A template but suspect this was the result of difficulties in the sequential alignment of α-helices, as in a restricted comparison omitting the AlphaFold α-helix signal peptide (residues 36–305), a better TM-Score of 0.87 was obtained in line with our other comparisons. A smaller improvement in TM-Score of 0.64 was also obtained by comparing the AlphaFold TM region / GT2 domain (residues 416–855) with the corresponding region of the BcsA protein (residues 140–700) reflecting problems associated in the prediction of TM α-helices either side of the GT2 domain and subsequent alignment. However, the alignment between the *Rs*BcsA template and the AlphaFold model was sufficiently good to allow us to superpose structures including the cellulose polymer which passes up through the *Rs*BcsA TM channel and turns sharply to follow the membrane-proximal surface of *Rs*BcsB the periplasm [30]. In the AlphaFold model, the superposed cellulose non-reducing end is positioned near IF2 which includes the Q(Q/R)xRW motif, whilst the reducing end projects out from the base of the GH17 domain (see **S4 Fig** for images of the superposed model). The cellulose polysaccharide binds in the *Rs*BcsA TM domain with a significant bend, which is determined by the overall structural arrangement in complex with *Rs*BcsB; however, the *Rs*BcsA-cellulose complex structure lacks a GH17 domain and therefore we suggest that in Orphan proteins the cellulose polysaccharide reducing end would extend into the GH17 active site cleft.

#### Structural prediction programmes reveal common features and a consensus model

**Fig 7:**
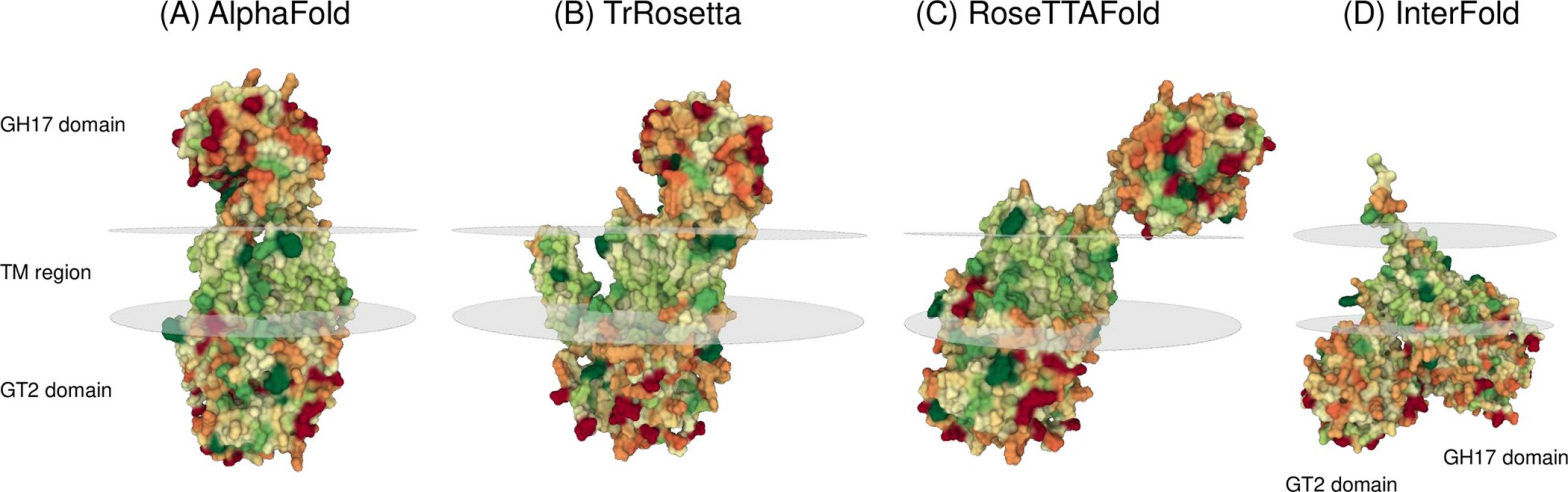
Common features are seen in *Pf*SBW25 Orphan protein structural predictions produced by different servers. Shown here are predicted structures of the *Pseudomonas fluorescens* SBW25 Orphan protein produced by AlphaFold (**A**), TrRosetta (**B**), RoseTTAFold (**C**), and IntFOLD6 (**D**). The relative positioning of the GH17 domain, transmembrane (TM) region and GT2 domain are indicated along with the position of a predicted lipid bilayer (grey ovals). Note that relative sizes vary from image to image and volumes may be hard to assess. Surface hydrophobicity is represented by cold colours with hydrophilic surfaces indicated by warmer colours. The models were produced by AlphaFold Colab Notebook [82, 83], IntFOLD6 [84], RoseTTAFold [86], and TrRosetta [89–91] and PDB files are available (see **S2 File**). Models were visualised with Mol* 3D Viewer [92] using molecular surface and membrane orientation representations and colouring residues according to hydrophobicity.

A visual comparison of the InterFOLD6, RoseTTAFold and TrRosetta predicted structures for the *Pf*SBW25 Orphan protein (**Fig 7**) suggests that the TrRosetta prediction is very similar to AlphaFold, except for the projection of two TM helices (TM7 & TM8) apart from the main TM structure. The way TrRosetta has broken up the TM region looks unusual, and it would be interesting to simulate the dispersion of lipids around this structure to see whether the projection breaks the upper lipid layer or not. In both the AlphaFold and TrRosetta predictions, surface representations also suggest substantive tertiary interactions stabilizing the GH17 domain next to the TM region. In contrast, RoseTTAFold suggests a different configuration that places the GH17 domain out on an extended unstructured linking sequence away from the TM region and the lipid bilayer (**Fig 7**). InterFOLD6 suggests a third configuration with the GH17 domain perpendicular to a more cone-shaped TM region and below the membrane visualised by Mol* 3D Viewer. The linking sequence lengths differ slightly between models, with AlphaFold and RoseTTAFold predicting a short α-helix (residues 284–288) in this region, and all but RoseTTAFold looping the sequence around rather than showing it as an extended, linear structure. As for AlphaFold, non-covalent interactions linking the GH17 domain, unstructured sequence and the exposed surface of the TM region were seen in the other models (**S4 Table**) with the most identified in the InterFOLD6 model where the periplasmic face of TM region was larger and allowing greater contact with the GH17 domain. The differences in positioning the GH17 domain in these models illustrates problems in positioning long unstructured regions linking discrete domains and assessing interactions which might stabilise tertiary structures. We note that the relative positioning of the GH17 and GT2 domains with respect to the bacterial inner membrane could be confirmed experimentally using Alkaline phosphatase (PhoA) fusions [110] while protease-sensitivity assays could be used to investigate the separation between the top of the TM region and the base of the GH17 domain. Transmembrane and membrane-associated proteins are often found in complexes with other proteins, and the interactions between these might have a significant impact on the positioning of flexible structures such as the TrRosetta TM projection and the RoseTTAFold GH17 domain.

As expected, the Pairwise Structure Alignments of predicted structures resulted in higher RMSD values (9.65–18.05) and lower TM-Scores (0.33–0.62) (**Table 2C**) than seen in our earlier comparisons of single-domain models and structural templates. Differences between homologous structures are generally the result of changes in the positioning of α-helices and β-sheets packed within domains as well as local changes especially in α-helices [13], and AI / Deep learning methods are successful in producing higher-level representations of predicted structures but may not be so consistent with smaller-scale details [35]. Although we found variation between models in the positioning of structures along the primary amino acid sequence (**S3 Table**), we were able to recognise similar organisation of TM helices, α-helices, and β-sheets across the predicted structures. The AlphaFold and TrRosetta models adopted similar organisation with the RoseTTAFold model having an extended unstructured sequence linking the GH17 domain and TM region. The InterFOLD6 model differs substantially from these structures but retains the same relationship between the TM region and GT2 domain. Molecular surface representations of the AlphaFold and TrRosetta models suggest that the GH17 domain and the exposed surface of the TM region are closely fitted, though no significant difference in non-covalent interactions that might stabilise the positioning of the GH17 domain were found between the four models.

On this basis we propose a consensus predicted structure for the *Pf*SBW25 Orphan protein based on the AlphaFold transmembrane ovoid-like model which places the GH17 domain, TM region and GT2 domain along a central axis with the GH17 domain in the periplasm and the GT2 domain in the cytoplasm, in agreement with our HMMSCAN, Proetus2, and Protter predictions and earlier speculative schematic structures [47, 48]. It should be noted that we have not determined that the AlphaFold predicted structure is somehow better than the other others (this would require a physical structure for comparison), rather it shows the most common positioning of the GH17 domain (with TrRosetta), TM region structure (with RoseTTAFold), and positioning of the GT2 domain (with TrRosetta and RoseTTAFold) (**Fig 7**). As for all predicted structures, it is unclear whether our consensus represents the actual structure adopted by the real protein [35] and how much this would be distorted by substrate and product binding and interaction with other proteins in the inner membrane and periplasm of the bacterial cell, and these possibilities would need to be investigated experimentally.

#### Predicted structures for other Orphan proteins

**Fig 8:**
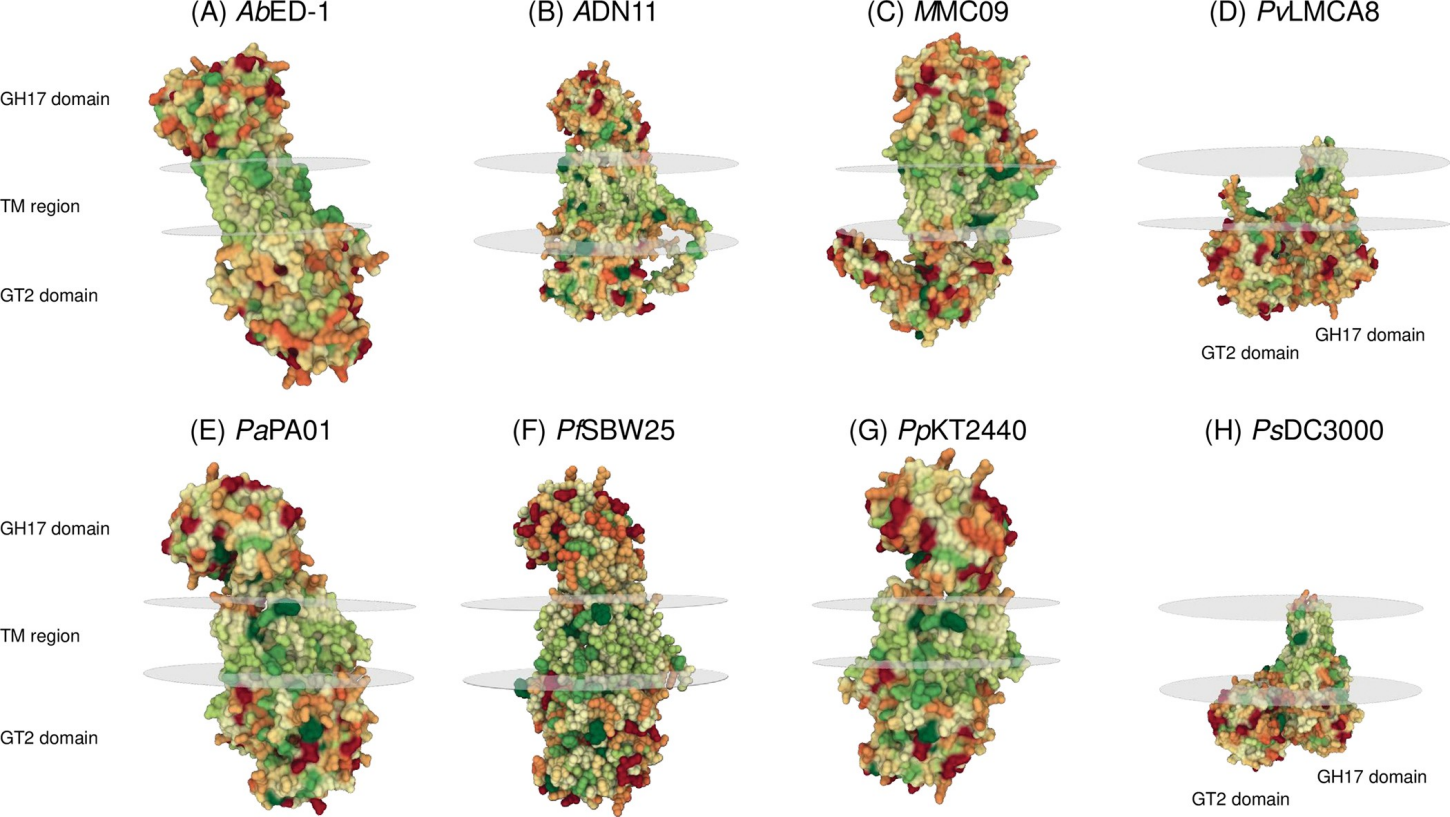
The Orphan protein two-domain structure is conserved across clades. Shown here are the AlphaFold predicted structures of Orphan proteins from other model *Pseudomonas* spp. and representatives of sister clades identified in the UPGMA analysis of homologs. Clade 3 representative *Arcobacter butzleri* ED-1 (**A**); Clade 4 representative *Azoarcus* strain DN11 (**B**); Clade 5 representative *Methylomonas methanica* MC09 (**C**); Clade 6 representative *P*. *viridiflava* LMCA8 **(D**); Clade 6 member *P*. *aeruginosa* PA01 (**E)**; Clade 6 member *P*. *fluorescens* SBW25 (**F**); Clade 6 member *P*. *putida* KT2440 (**G**); Clade 6 member *P*. *syringae* DC3000 (**H**). The relative positioning of the GH17 domain, transmembrane (TM) region and GT2 domain are indicated along with the position of a predicted lipid bilayer (grey ovals). Note that relative sizes vary from image to image and volumes may be hard to assess. Surface hydrophobicity is represented by cold colours with hydrophilic surfaces indicated by warmer colours. The models were produced by AlphaFold Colab Notebook [82, 83] and the PDB files are available (see **S2 File**). Models were visualised with Mol* 3D Viewer [92] using molecular surface and membrane orientation representations and colouring residues according to hydrophobicity.

We also determined AlphaFold predicted protein structures for the *Pa*PA01, *Pp*KT2440 and *Ps*DC3000 Orphan protein sequences (**S2 File**). Both *Pa*PA01 and *Pp*KT2440 predictions were very similar to the AlphaFold *Pf*SBW25 Orphan model (**Fig 8** & **Table 2**), confirming our consensus structure for these highly homologous proteins. In the recent AlphaFold Protein Structure Database update (v27 January 2022) [83], we noted the release of the *Pa*PA01 Orphan structure (AlphaFold DB AF_Q914H4-F1) created by the AlphaFold Monomer v2.0 pipeline. This prediction is almost identical to our AlphaFold Colab Notebook structure (FATCAT RMSD 0.73, TM-score 1, Score 2587.7) and demonstrates the reliability of the more accessible but simplified Colab Notebook server. The small RSMD value differentiating the two AlphaFold models serves as a reminder that as AI/Deep learning methods improve and databases expand, predicted structures will become outdated and should not be used as fixed references or gold standards.

In contrast to the *Pa*PA01 and *Pp*KT2440 Orphan models, the *Pf*DC3000 prediction positioned the GH17 domain perpendicular to the TM region and on the same side of the membrane as the GT2 domain as for the InterFOLD6 *Pf*SBW25 Orphan model. The *Pf*DC3000 Orphan structure and Protter protein topology prediction agree, with residues 1–43 shown in the model as an extended and largely unstructured region leading right up to the edge of the TIM-barrel. HMMSCAN and other transmembrane topology and signal peptide predictions including LipoP [76], Phobius [77], PRED-TAT [78], and SignalP [81] failed to identify a signal peptide in the *Pf*DC3000 Orphan. Protter also failed to identify signal peptides and predicted the GH17 domain to be cytoplasmic for five other *P*. *syringae* Orphans we analysed (B728a, ICMP 9617, NCPPB 4273, UMAF0158, and 41a) and they do not share any significant sequence conservation until residue 35 of the *Ps*DC3000 Orphan protein suggesting that the *P*. *syringae* Orphan subclade has lost the signal peptide found in other Orphan proteins. However, as our investigation of the *Pf*SBW25 Orphan protein has shown the signal sequence had no significant impact on the predicted structure, it is unlikely that residues 1–43 have a great impact on the structure of the *Ps*DC3000 Orphan protein. Further modelling of hybrid *Pf*SBW25/*Ps*DC3000 proteins might reveal which sequences are responsible for the different orientation of the G12 domain in these AlphaFold models, and PhoA fusions [110] used to confirm the positioning of the *Ps*DC3000 Orphan protein in the bacterial inner membrane. The different structure predicted for *Ps*DC3000 suggests that the protein is non-functional despite the high level of sequence conservation of these proteins within the *P*. *syringae* subclade.

#### Predicted consensus structure is conserved in more distant homologs

More distant homologs of the *Pf*SBW25 Orphan protein, such as *Arcobacter butzleri* ED-1 which was identified as having the same Pfam PF00332 –PF13641 domain architecture as other Orphan proteins rather than by BLAST+ analysis or through PseudoCAP, and shares only 35.1% amino acid sequence identity (54.1% similarity) to the *Pf*SBW25 Orphan, may not therefor be expected to share the same protein folds [13–16]. However, AlphaFold predicted structures for representative Orphans from the sister clades identified in our earlier UPGMA comparison of homologs (Clade 3, *Ab*ED-1; Clade 4, *Azoarcus* strain DN11; and Clade 5, *Methylomonas methanica* MC09, respectively, **Table 1**) were visually similar to the *Pf*SBW25 Orphan consensus structure (**Fig 8**) (like *Ab*ED-1, *A*DN11 and *Mm*MCO9 were identified by domain architecture though their annotations vary: *Ab*ED-1: glycosyltransferase, *A*DN11: Uncharacterised protein, and *Mm*MCO9: Glycosyl transferase family 2).

Pairwise Structure Alignments confirmed structural similarities, with FATCAT RMSD values of 1.86–2.62 and TM-Scores of 0.85–0.92 (**Table 2D**). Unsurprisingly, the predicted structure of the Clade 6 representative, *Pseudomonas viridiflava* LMCA8, which is also a member of the *P*. *syringae* Subclade 6.1, was more similar to the *Ps*DC3000 protein (**Fig 8**). These comparisons suggests that the consensus Orphan structure is highly conserved within Clade 6 containing the *Pseudomonas* Orphans (excepting the *P*. *syringae* proteins as discussed), and across into sister clades where sequence conservation tis progressively reduced.

### Orphans are likely to be cyclic-β-glucan (CβG) synthases

Our interpretation of the structure and function of the Orphan protein was initially driven by homology to BcsA cellulose synthase subunit. However, significant sequence and structural variation is found among GT2 / GT-A homologs which have a range of synthase activity with predictive structures of cellulose, chitin and curdlan synthases, as well as β-(1–3,1–4)-glucan synthase, all produced using the *Rs*BcsA structure [27, 28, 111, 112]. This means that the GT2 domain may not synthesize cellulose or a related β-(1,4) glucan such as chitin, or even a β-(1,3) glucan such as curdlan, and we are not aware of any definitive sequence or structural feature in the Orphan protein which suggests it produces a particular β-glucan. Despite the differences in width and stacking of the glycosyl units in different glucans, it is possible to position the curdlan chain in the transmembrane channel of the predicted structure of a curdlan synthase based on *Rs*BcsA template [28], and single amino acid changes in TM6 of the plant β-(1–3,1–4)-glucan synthase alters the proportion of the two linkages in the glucan product [111]. Nonetheless, we are able to suggest a model for function of the Orphan synthase based on earlier work linking the Orphan protein (NvdB) in *Pa*PAK and *Pa*PA14 to the production of a partially glycerol-phosphorylated cyclic-β-glucan (CβG) [48, 50, 51], enzymatic characterization and molecular dynamics simulations of Glt1 and Glt3 fusion proteins [48, 49], and our understanding of the relationship of the GH17 domain, TM region and GT2 domain through the consensus structure obtained in this work (the distorted predicted structure obtained for *Ps*DC3000 suggests that this Orphan would be non-functional and we note that no periplasmic cyclic glucans were identified in *Ps*R32 [113]).

We suggest that the Orphan GT2 domain acts as a Leloir-type synthase to produce a β-(1–3)-glucan chain which passes through the transmembrane channel to the GH17 active site cleft where transglycosylation hydrolysis, elongation, and cyclization reactions produce the 12–16 glucosyl backbone [51] (we accept that the GH17 and GT2 functions might be provided by separate proteins, but the identification of double-domain containing Orphan proteins within the *Pseudomonas* and in other genera suggests that the two domains are functionally connected in the same transmembrane protein). The major cleavage site of GLT1 is at the fourth linkage from the non-reducing end of the glucan chain [48, 49], but 3–4 oligosaccharide fragments would need to be cleaved and re-joined through the elongation reaction, retaining the original β-(1–3) linkage, before cyclization to produce the CβG. However, the same product might be more efficiently produced by looping the β-(1–3)-glucan chain through the active site with only one hydrolysis reaction between the 12–16 linkages followed by cyclization. Clearly this process needs to be confirmed by experimentation and comparison of enzymatic behaviour of Orphan mutant proteins produced by site-directed or random scanning mutagenesis might identify the critical residues and motifs used by the GH17 domain to modify the glucan chain produced by the GT2 domain.

We suggest that a second, membrane-associated enzyme is then responsible for the partial substitution of glucosyl groups in the CβG. Phosphoglycerol transferase (MdoB; UniProtKB P39401) transfers phosphoglycerol residues from phosphatidylglycerol to membrane-derived oligosaccharides in the periplasm of *E*. *coli* [114] and we have identified possible but distant homologs of this protein in *Pa*PA01, *Pf*SBW25, *Pp*KT2440 and *Ps*DC300 with 22–27% sequence identity which might perform this function (see **S5 Table** for a list of MdoB homologs) (we note that in *Desulfofustis glycolicus* DSM 9705 the orphan gene is located immediately upstream of a MdoB homolog, but this is the only example we are aware of). The involvement of a MdoB homolog in the partial substitution of glucosyl groups would need to be confirmed experimentally by chemical analyses and genetic approaches. It would be interesting to model the interactions between the Orphan protein and *Ec*MdoB or one of the MdoB homologs to determine if CβG synthesis and substitution are linked, which is perhaps supported by the high level of substitution observed [51], though the two processes may happen independently if the MdoB homolog had a high affinity for CβG. We note that of all the *Orphan–dapE* gene syntenies we have checked, one has the orphan gene immediately upstream of a MdoB homolog (*Desulfofustis glycolicus* DSM 9705), and this system in particular might be worthy of future investigation.

## Conclusion

Bacteria produce a wide range of structurally diverse polysaccharides with numerous functional roles, yet it appears that our understanding of the genes and enzymes involved in producing these remain incomplete even in well-studied model strains. The rapid development of sequence and structure-based annotation allows the rapid identification of genes [1, 2], but it remains problematic that sequence, structure, and function are not always robustly linked [3, 8, 9, 17] and that some sequences may be misannotated. Our investigation of the Orphan proteins, highly conserved within the *Pseudomonas*, is a good example of this problem, as sequence homology to the cellulose synthase catalytic subunit BcsA suggested a role in cellulose production, whereas further investigation of conserved domains and predicted structures have allowed us to suggest they represent a novel family of cyclic-β-glucan (CβG) synthases, in agreement with earlier characterization of the GH17 glycosyl hydrolase family domain and transposon mutants [47–49, 51]. Our comparison of predicted structural models has allowed us to identify a consensus transmembrane ovoid-like structure which positions the GH17 domain in the periplasm and the GT2 glycosyltransferase family domain in the cytoplasm. These findings have given us sufficient insights to plan further *in silico* and biochemical analysis to confirm Orphan function and investigate the functional role of CβG produced by pseudomonads in a wide range of environments including soil and plant surfaces as well as during plant and human pathogenesis.

Our use of predictive modelling also highlights variation in the structures produced by different AI / Deep Learning-based free modelling approaches. In the absence of highly homologous template structures, we advise the use two or three approaches followed by pairwise structural comparison to evaluate models and propose a consensus structure. Furthermore, models made available in various databases need to be regularly revised as algorithms become more sophisticated and template databases expand.

## Supporting information

S1 Fig GC content of the *orphan-dapE* regions of *Pseudomonas fluorescens* SBW25, *P*. *putida* KT2440 and *P*. *syringae* DC300. Shown here are GC content plots covering the *orphan* (blue) and *dapE* (gold) genes and adjacent genes (grey) not involved in DapE activity or cellulose production. Each of the plots covers approximately 6,000 bp and the locus tags (from left to right) are *Pf*. SBW25: PFLU1258, PFLU1259, PFLU1260 and PFLU1261; *Pp*. KT2440: PP1524, PP1525, PP1526 and PP1527; and *Ps*. DC3000: PSPTO1522, PSPTO1523, PSPTO1524 and PSPTO1525. The horizontal dashed line indicates the mean GC content for each genome. Genomes were obtained from PseudoCAP [60] as GBK files and were viewed using Artemis [61]. The GC plots are copies of the Artemis graphs.
(PPTX)

S2 Fig Phylogenetic tree of Orphan protein homologs. Shown here is composite figure of an un-rooted UPGMA phylogenetic tree produced by Clustal Omega Simple Phylogeny [70] of 190 Orphan protein homologs and drawn with real and cladogram (uniform) scales. Species and protein annotation (in parentheses) and genetic distances provided for each protein (see **S1 File** for protein sequences). The UPGMA tree is divided into seven clades **(A)** with Clade 6 containing all *Pseudomonas* spp. Orphan proteins and further subdivided into five subclades **(B)** (inset figures are from **Fig 3**). *Rhizomucor miehei* CUA432 Bgt17A is indicated by the white circle (Clade 1). *Escherichia coli* MG1655 and *Rhodobacter sphaeroides* 2.4.1 BcsA reference proteins are indicated by the black circles (Clade 2). Orphan proteins from *Pseudomonas aeruginosa* PA01 (Clade 6 Subclade 5), *P*. *fluorescens* SBW25 (Clade 6, Subclade 4), *P*. *putida* KT2440 (Clade 6, Subclade 4), and *P*. *syringae* DC3000 (Clade 6, Subclade 1) are indicated by coloured squares. *Rhizobium meliloti* 1021 NdvB and *Schizosaccharomyces pombe* 972 Ags1 were chosen as outliers for this tree (Clade 7). The real and cladogram trees and text are copied from the Simple Phylogeny output.
(PPTX)

S3 Fig Identification of the transmembrane helices in the AlphaFold predicted structure of the *Pf* SBW25 Orphan protein. Shown here is a view of the AlphaFold model of the *Pseudomonas fluorescens* SBW25 Orphan protein with the cartoon representation colour-coded according to secondary structure **(A)**. These include α-helices (magenta), β-sheets (gold), and loops (green) (sections with poor certainty are in light green and white). The GH17 domain, transmembrane (TM) region and GT2 domain are indicated along with the position of a predicted lipid bilayer (grey ovals). The TM region includes seven transmembrane helices (TM1–7) which were also identified by Proteus2 [79] and Protter [80] **(B)**. The signal peptide (SP), identified by HMMSCAN [75], Proteus2 and Protter, is shown aligned with the other TM helices. The model was produced by AlphaFold [82, 83] and the PDB file is available (see **S2 File**). The model was visualised with Mol* 3D Viewer [92] using cartoon and membrane orientation representations and colouring residues according to secondary structure.
(PPTX)

S4 Fig The elongating cellulose chain seen in the *Rs*BcsAB crystal structure can be superposed on the AlphaFold predicted structure of the *Pf* SBW25 Orphan protein. Shown here are views of the AlphaFold model of the *Pseudomonas fluorescens* SBW25 Orphan protein superimposed with the cellulose polymer as visualised in the homologous *Rhodobacter sphaeroides* 2.4.1 BcsAB (*Rs*BcsAB) X-ray crystal structure [32, 33]. The Orphan protein **(A)** is shown as a cartoon representation colour-coded according to secondary structure with α-helices (magenta), β-sheets (gold), and loops (green) (sections with poor certainty are in light green and white). The GH17 domain, transmembrane (TM) region and GT2 domain are indicated along with the superimposed position of a short cellulose chain (linked purple beads) with the reducing end projecting away from the base of the GH17 domain and the non-reducing (elongating) end buried at the base of the TM region. In the *Rs*BcsAB crystal structure, the cellulose chain passes up through a transmembrane channel where it is then threaded into the *Rs*BcsC porin in the outer membrane. A similar transmembrane channel appears to be present in the AlphaFold Orphan model, but the cellulose chain is likely to continue to project towards the GH17 Orphan domain rather than adopting an acute turn as seen in the *Rs*BcsAB crystal structure. A second view of the Orphan protein is given looking down into the centre of the TIM-barrel like structure of the GH17 domain **(B)**. Although it seems as if the cellulose chain could project up into the TIM-barrel, it is more likely that it will come into contact with the GH17 cleft and active site residues. The AlphaFold model (**S2 File**) [82, 83] was superposed with the *Rs*BcsAB crystal structure which included the cellulose chain with Pairwise Structure Alignment [94]. The superposed model was then visualised with Mol* 3D Viewer [92] with only the cellulose chain and Orphan protein visible and using cartoon representation and colouring residues according to secondary structure.
(PPTX)

S1 File FASTA file of proteins investigated in this work. Proteins are listed by species and strain and were include as Reference proteins or Orphan or BcsA homologues identified in PseudoCAP [60], unpublished genomes, by BLAST or InterPro IPR000490-PF13641. UniProt Accessions are provided plus protein function if known.
(PDF)

S2 File Single-domain homology models and predicted structure models. This is a list of single-domain homology models and predicted structure models (PDB files) generated in this work and available from DOI: XXX (to be added after acceptance).
(PDF)

S3 File Multiple sequence alignment of 26 Orphan proteins from representative *P*. *aeruginosa*, *P*. *fluorescens*, *P*. *putida* and *P*. *syringae* strains. Clustal Omega [70] was used to produce a multiple sequence alignment of 26 Orphan proteins identified in *Pseudomonas aeruginosa* AZPAE12140, BL14, PAK, PA01, PA14, LESB58, 19BR and 3573, *P*. *fluorescens* ICMP 11288, ICMP 3512, KF1, LMG 5329, SBW25, SS101, WH6 and WS 5037, *P*. *putida* KT2440, S610, W619 and YKD221, and *P*. *syringae* B728a, DC3000, ICMP 9617, NCPPB 4273, UMAF0158 and 41a strains, using the *Pa*PA14 Orphan (NdvB) as the reference sequence (see **S1 File** for protein sequences). Orphan proteins show 95.3–100.0% coverage and 46.9–99.2% sequence identity normalised by aligned length. The Mview [70] file was copied and annotated to show the signal peptide sequence predicted by Proteus2 [79], the first (GH17) and second (GT2) Orphan domains, and conserved domains, motifs, and residues found in homologous fungal proteins and BcsA proteins.
(PDF)

S1 Table BcsA and Orphan proteins from *Pseudomonas fluorescens* SBW25, *P*. *putida* KT2440 and *P*. *syringae* DC3000. This lists locus tags and UniProtKB accessions, PseudoCAP annotations, number of residues, genome coordinates, and amino acid identity and similarity to the *Ec*BcsA reference protein.
(PDF)

S2 Table List of *Pseudomonas* species containing Orphan proteins. This lists *Pseudomonas* species and strains containing Orphan genes identified in our sampling of PseudoCAP entries, by BLAST searches, and inspection of unpublished draft genome sequences. See **S1 File** for all protein sequences.
(PDF)

S3 Table Comparison of secondary structures in Orphan homology models and predicted structures. This lists reference transmembrane helices, α-helices and β-sheets identified in the *Rhizomucor miehei* CAU432 and *Rhodobacter sphaeroides* 2.4.1 BcsA X-ray crystal structures and seen in the single-domain models and predicted structures of the *Pseudomonas fluorescens* SBW25 Orphan protein. The start and stop residues for each structure is recorded showing the variation from that shown in the Phyre^2^ homology models for the GH17 domain and the TM region and GT2 domain.
(PDF)

S4 Table Interactions between GH17 domain and TM region in Orphan predicted structures. This lists the non-covalent bonds identified in the AlphaFold, InterFOLD6, RoseTTAFold and TrRosetta predicted structures of the *Pseudomonas fluorescens* SBW25 Orphan protein connecting residues located in the GH17 domain, linking (unstructured) sequence, and the exposed surface of the TM region.
(PDF)

S5 Table Homologs of the *Escherichia coli* Phosphoglycerol transferase MdoB. This lists MdoB homologs identified in *Pseudomonas aeruginosa* PA01, *P*. *fluorescens* SBW25, *P*. *putida* KT2440, and *P*. *syringae* DC3000.
(PDF)
