# Supplementary material for: Bioinformatics characterization of BcsA-like orphan proteins suggest they form a novel family of pseudomonad cyclic-β-glucan synthases
Source: PLoS One. 2023 Jun 2;18(6):e0286540. doi: 10.1371/journal.pone.0286540 (PMC10237404; doi:10.1371/journal.pone.0286540)
Supplement: S1 Fig — Shown here are GC content plots covering the orphan (blue) and dapE (gold) genes and adjacent genes (grey) not involved in DapE activity or cellulose production. Each of the plots covers approximately 6,000 bp and the locus tags (from left to right) are Pf. SBW25: PFLU1258, PFLU1259, PFLU1260 and PFLU1261; Pp. KT2440: PP1524, PP1525, PP1526 and PP1527; and Ps. DC3000: PSPTO1522, PSPTO1523, PSPTO1524 and PSPTO1525. The horizontal dashed line indicates the mean GC content for each genome. Genomes were obtained from PseudoCAP [60] as GBK files and were viewed using Artemis [61]. The GC plots are copies of the Artemis graphs. (PPTX) [file pone.0286540.s001.pptx]

## Slide 1
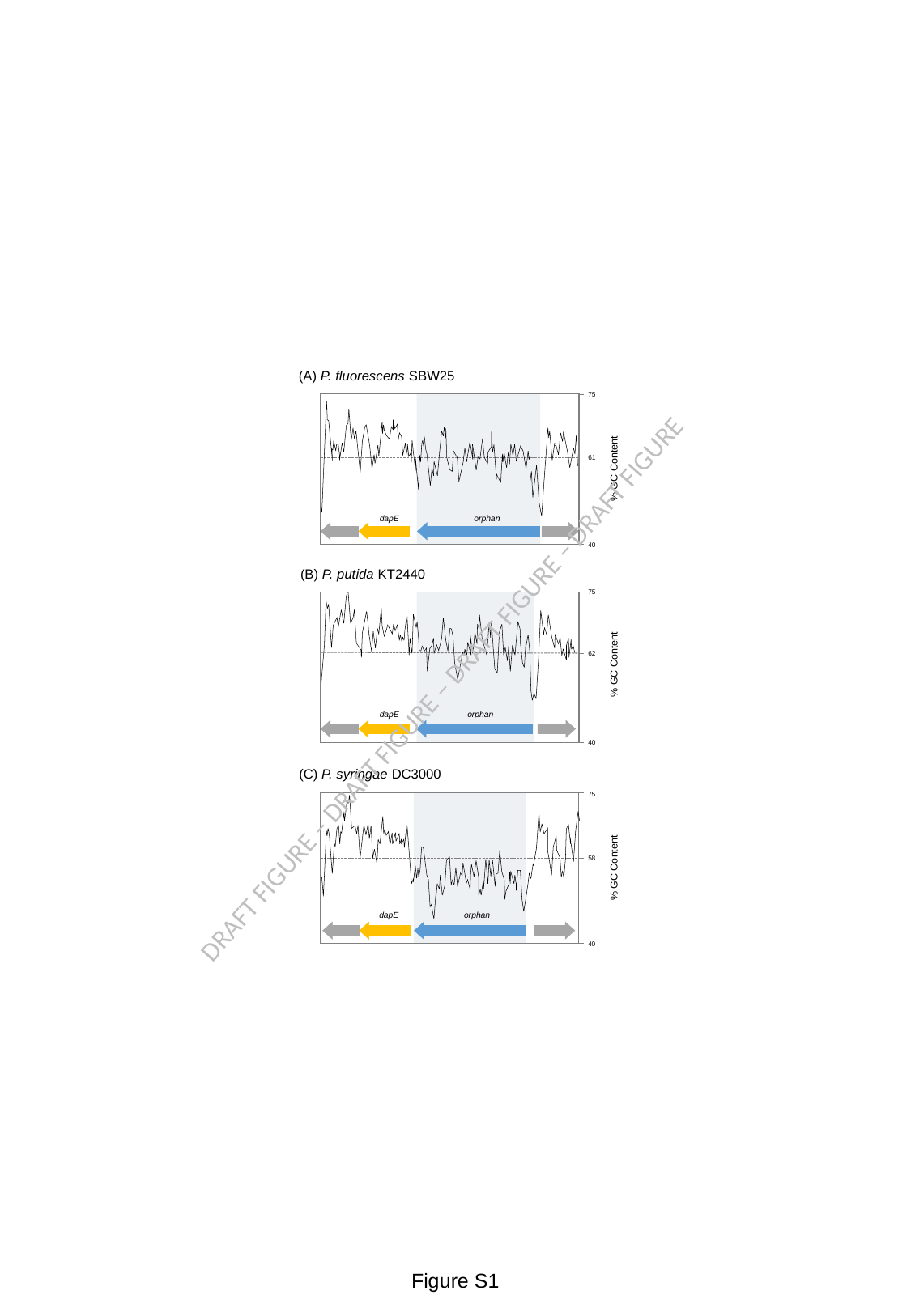

(A) P. fluorescens SBW25
75
61
dapE
orphan
40
% GC Content
(B) P. putida KT2440
75
62
dapE
orphan
40
% GC Content
(C) P. syringae DC3000
75
58
40
dapE
orphan
% GC Content
DRAFT FIGURE – DRAFT FIGURE – DRAFT FIGURE – DRAFT FIGURE
Figure S1
