# Supplementary material for: Bioinformatics characterization of BcsA-like orphan proteins suggest they form a novel family of pseudomonad cyclic-β-glucan synthases
Source: PLoS One. 2023 Jun 2;18(6):e0286540. doi: 10.1371/journal.pone.0286540 (PMC10237404; doi:10.1371/journal.pone.0286540)
Supplement: S2 Fig — Shown here is composite figure of an un-rooted UPGMA phylogenetic tree produced by Clustal Omega Simple Phylogeny [70] of 190 Orphan protein homologs and drawn with real and cladogram (uniform) scales. Species and protein annotation (in parentheses) and genetic distances provided for each protein (see S1 File for protein sequences). The UPGMA tree is divided into seven clades (A) with Clade 6 containing all Pseudomonas spp. Orphan proteins and further subdivided into five subclades (B) (inset figures are from Fig 3). Rhizomucor miehei CUA432 Bgt17A is indicated by the white circle (Clade 1). Escherichia coli MG1655 and Rhodobacter sphaeroides 2.4.1 BcsA reference proteins are indicated by the black circles (Clade 2). Orphan proteins from Pseudomonas aeruginosa PA01 (Clade 6 Subclade 5), P. fluorescens SBW25 (Clade 6, Subclade 4), P. putida KT2440 (Clade 6, Subclade 4), and P. syringae DC3000 (Clade 6, Subclade 1) are indicated by coloured squares. Rhizobium meliloti 1021 NdvB and Schizosaccharomyces pombe 972 Ags1 were chosen as outliers for this tree (Clade 7). The real and cladogram trees and text are copied from the Simple Phylogeny output. (PPTX) [file pone.0286540.s002.pptx]

## Slide 1
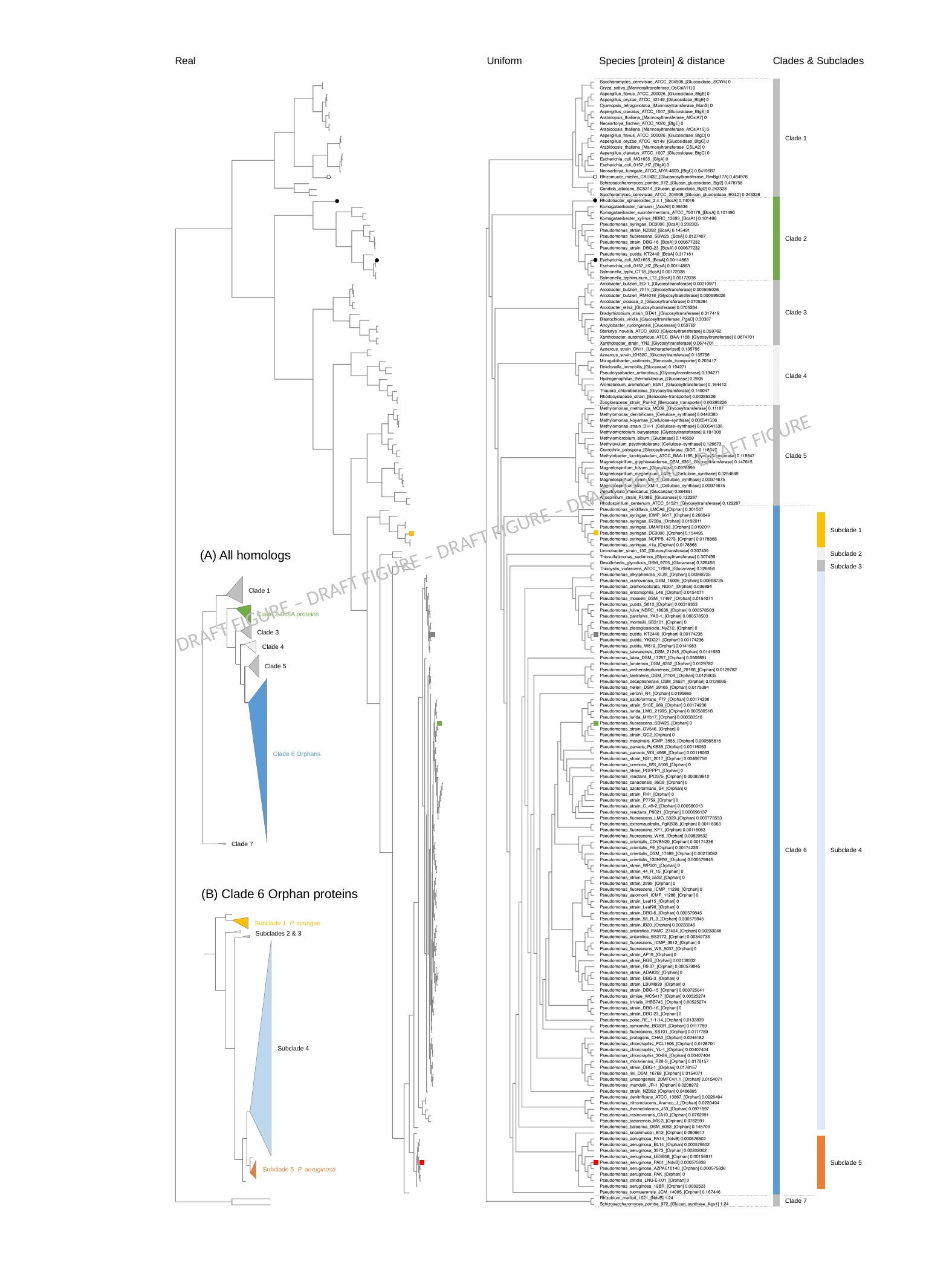

Real
Uniform
Species [protein] & distance
Clades & Subclades
Clade 1
Clade 2
Clade 3
Clade 4
Clade 5
Subclade 1
(A) All homologs
Clade 1
Clade 2 BcsA proteins
Clade 3
Clade 4
Clade 5
Clade 6 Orphans
Clade 7
Subclade 2
Subclade 3
Clade 6
Subclade 4
(B) Clade 6 Orphan proteins
Subclade 1 P. syringae
Subclades 2 & 3
Subclade 4
Subclade 5 P. aeruginosa
Subclade 5
Clade 7
DRAFT FIGURE – DRAFT FIGURE – DRAFT FIGURE – DRAFT FIGURE – DRAFT FIGURE
