# Supplementary material for: Bioinformatics characterization of BcsA-like orphan proteins suggest they form a novel family of pseudomonad cyclic-β-glucan synthases
Source: PLoS One. 2023 Jun 2;18(6):e0286540. doi: 10.1371/journal.pone.0286540 (PMC10237404; doi:10.1371/journal.pone.0286540)
Supplement: S3 Fig — Shown here is a view of the AlphaFold model of the Pseudomonas fluorescens SBW25 Orphan protein with the cartoon representation colour-coded according to secondary structure (A). These include α-helices (magenta), β-sheets (gold), and loops (green) (sections with poor certainty are in light green and white). The GH17 domain, transmembrane (TM) region and GT2 domain are indicated along with the position of a predicted lipid bilayer (grey ovals). The TM region includes seven transmembrane helices (TM1–7) which were also identified by Proteus2 [79] and Protter [80] (B). The signal peptide (SP), identified by HMMSCAN [75], Proteus2 and Protter, is shown aligned with the other TM helices. The model was produced by AlphaFold [82, 83] and the PDB file is available (see S2 File). The model was visualised with Mol* 3D Viewer [92] using cartoon and membrane orientation representations and colouring residues according to secondary structure. (PPTX) [file pone.0286540.s003.pptx]

## Slide 1
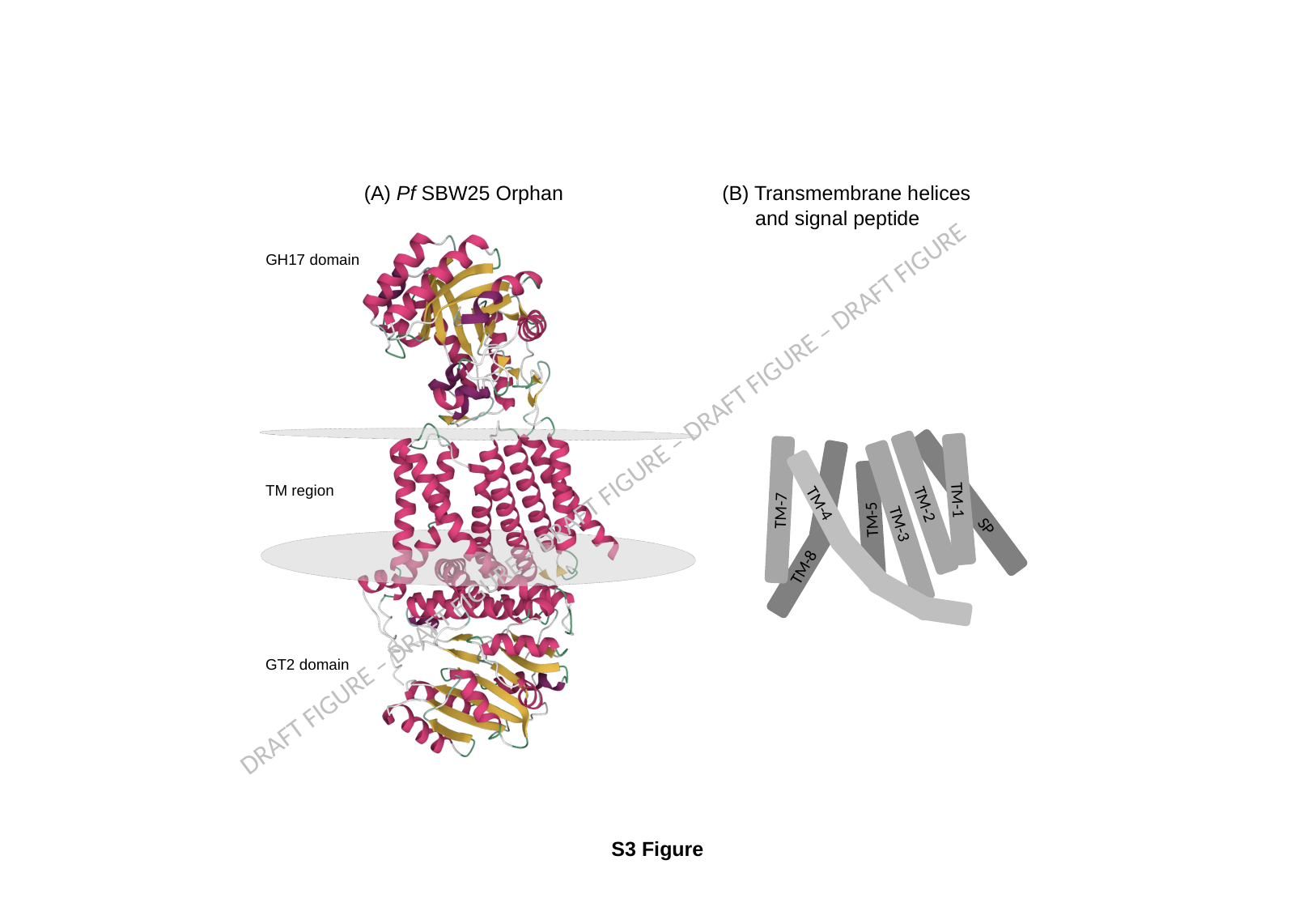

(A) Pf SBW25 Orphan
(B) Transmembrane helices
and signal peptide
GH17 domain
TM-7
TM-4
TM-5
TM-2
TM-1
TM-3
TM-8
SP
DRAFT FIGURE – DRAFT FIGURE – DRAFT FIGURE – DRAFT FIGURE – DRAFT FIGURE
TM region
GT2 domain
S3 Figure
