# Supplementary material for: Bioinformatics characterization of BcsA-like orphan proteins suggest they form a novel family of pseudomonad cyclic-β-glucan synthases
Source: PLoS One. 2023 Jun 2;18(6):e0286540. doi: 10.1371/journal.pone.0286540 (PMC10237404; doi:10.1371/journal.pone.0286540)
Supplement: S4 Fig — Shown here are views of the AlphaFold model of the Pseudomonas fluorescens SBW25 Orphan protein superimposed with the cellulose polymer as visualised in the homologous Rhodobacter sphaeroides 2.4.1 BcsAB (RsBcsAB) X-ray crystal structure [32, 33]. The Orphan protein (A) is shown as a cartoon representation colour-coded according to secondary structure with α-helices (magenta), β-sheets (gold), and loops (green) (sections with poor certainty are in light green and white). The GH17 domain, transmembrane (TM) region and GT2 domain are indicated along with the superimposed position of a short cellulose chain (linked purple beads) with the reducing end projecting away from the base of the GH17 domain and the non-reducing (elongating) end buried at the base of the TM region. In the RsBcsAB crystal structure, the cellulose chain passes up through a transmembrane channel where it is then threaded into the RsBcsC porin in the outer membrane. A similar transmembrane channel appears to be present in the AlphaFold Orphan model, but the cellulose chain is likely to continue to project towards the GH17 Orphan domain rather than adopting an acute turn as seen in the RsBcsAB crystal structure. A second view of the Orphan protein is given looking down into the centre of the TIM-barrel like structure of the GH17 domain (B). Although it seems as if the cellulose chain could project up into the TIM-barrel, it is more likely that it will come into contact with the GH17 cleft and active site residues. The AlphaFold model (S2 File) [82, 83] was superposed with the RsBcsAB crystal structure which included the cellulose chain with Pairwise Structure Alignment [94]. The superposed model was then visualised with Mol* 3D Viewer [92] with only the cellulose chain and Orphan protein visible and using cartoon representation and colouring residues according to secondary structure. (PPTX) [file pone.0286540.s004.pptx]

## Slide 1
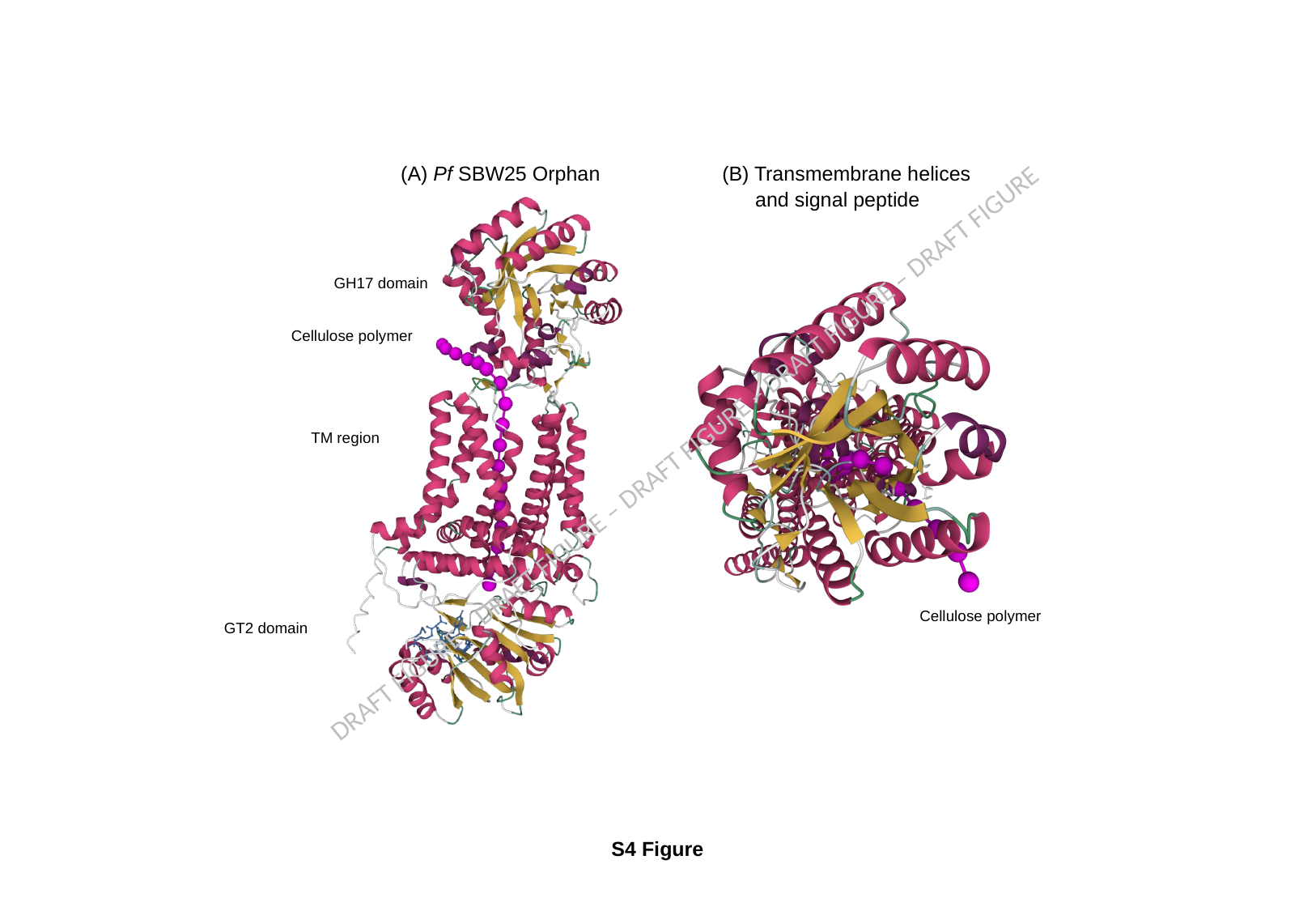

(A) Pf SBW25 Orphan
(B) Transmembrane helices
GH17 domain
Cellulose polymer
TM region
Cellulose polymer
GT2 domain
and signal peptide
DRAFT FIGURE – DRAFT FIGURE – DRAFT FIGURE – DRAFT FIGURE – DRAFT FIGURE
S4 Figure
