# Supplementary material for: Bioinformatics characterization of BcsA-like orphan proteins suggest they form a novel family of pseudomonad cyclic-β-glucan synthases
Source: PLoS One. 2023 Jun 2;18(6):e0286540. doi: 10.1371/journal.pone.0286540 (PMC10237404; doi:10.1371/journal.pone.0286540)
Supplement: S1 File — Proteins are listed by species and strain and were include as Reference proteins or Orphan or BcsA homologues identified in PseudoCAP [60], unpublished genomes, by BLAST or InterPro IPR000490-PF13641. UniProt Accessions are provided plus protein function if known. (PDF) [file pone.0286540.s005.pdf]

Supporting File S1. **FASTA files of proteins investigated in this work.**

```
>Ancylobacter_rudongensis_[Glucanase] InterPro PF00332-PF13641: A0A1G4QCQ5 Exo-
beta-1,3-glucanase, GH17 family
MRPAIRVAAAAVAVVTCVHVFVWLALRQEVSAPAAPDRFQSMSFAPYGRDANPDKGEPTTEAQIRADVEAVAPYTRAVRT
YASTGNLDLVARLAGEKGLKTTVGAWLDEDEARNEREITNAIDVAKKNSNVVGIVVGNESILRAERTPEQLIETIRRVKR
ETNVPVTTGETWDVWLDHPELVSAVDYIAAHMLPYWEGVSPEQVVDHTIAIYDRLRATYPGKRIVIAEFGWPSQGYNRDN
AVPGELTQAQIIRSFAARADALGIEYNLIEAFDAPWKSFEGSVGQYWGILDADRVAKFPLAGPITPSTYNATAAIALLLG
IAFSLPVFRFARLTLTQALVMVGAANLVGAWIATVTDHWLTHYVVGGNMVTMVVCLVLLVPLVVVMLYRVEELATIAFGR
TPSRLLRRDTVTAPSRTPKVSIHIPAYKEPPEMLKQTLDSVARLNWPNFECLVIINNTPDPAFWEPIEAHCRTLGERFKF
INLPKVAGFKAGALRAAMLQTAPDAEIIGIIDADYVVDANWLMDLVPTFEDPTVGIVQAPQDHRDADRSLLHEAMNTEYA
GFFDIGMVQRNEHDAIVVHGTMCLMRRAAMVEAGDWSSDTICEDTDLGLAIVERGWKSHYTSTRYGWGLLPDDFASFKKQ
RHRWAYGGMQIIKKHWRRMLPNAGTRLTPAQRREFSIGWVSWLGSESVGALVAILSLLWVPFVLGLGIAVPQRILTIPIV
FCFGIYLLHFIALYRLRVATTPVRMLGAAFAAMAVQFTVAKAVYDSFRYKDLAFARTAKGSWLADAARAFPALPEAIIGA
GLMLSAVGLRLTNWHAVVEVDLFAIALAIQSLPFLAAAGIGWLEGSTLNSFATWSALRGRALALLPGRTAQPQAIPEKIG

>Arabidopsis_thaliana_[Mannosyltransferase_AtCslA7] BLAST: Q9ZQN8 Glucomannan 4-
beta-mannosyltransferase 7, AtCslA7
MSPLPIFHRLPHATFSSFLLSLSQAGSSKTSVAFLNAFKSEDIIARIGLWWQLIRAVVVVPVFKFLVLLCLVMSVMFFVE
VMYMGIVVLYVKLFKRKPEKFYKWEAMEDDVECGSASYPMVLVQIPMYNEKEVCEQSIAAACKISWPSNRIIIQVLDDST
DPASKELVKKECDRWSKEGVNITFEIRDNRNGYKAGALREGMRHSYVKQCDYVAIFDADFQPDPDFLHRTVPFLIHNPKL
ALVQGRWEFVNAGQCMMTRLQEMSLSYHFTIEQQVGSSTFAFFGFNGTAGVWRISALNESGGWNDQTTVEDMDLAVRATL
RGWKFLYIDDLKVKSELPCSFKALRSQQHRWTCGPANLLRKMAGQIIRSENVSLWKKWYMLYSFFFMRKIVAHILTFCFY
CVILPATVLFPEVTVPKWAAFYLPSLITLLIAIGRLRSIHLLAFWVLFENAMSLLRAKALVMGLFETGRVQEWVVTEKLG
DTLKTKLIPQVPNVRFRERVHLLELLVGAYLLFCGIYDIVYGKNTLYVYLLFQSVAFFVVGFGFVGKYVPASSYLA

>Arabidopsis_thaliana_[Mannosyltransferase_AtCslA15] BLAST: Q9T0L2 Probable
glucomannan 4-beta-mannosyltransferase 15, AtCslA15
MFLLLKPLLSLHDLSLNLLSVMFHGETLKASVDGVGINMSTMWRETRNVFIVPLFKCIVVMCLIISLLVFVESVYMNLVV
LYVKLFNRKPEKVYKWEAMQEDMELGHQNYPMVLVQIPMYNEREVFELSIGAACRLTWPSDRLIVQVLDDSTDPAIMELV
SMECTKWASKDININYERRENRNGYKAGALKHGMRHSYVKQCQYLAIFDADFQPEPDYLQRAIPFLIHNPEVALVQARWR
FVNANTCLMTRMQEMSLNYHFMAEQQSGSTRHAFFGFNGTAGVWRMVAMEEAGGWKDRTTVEDMDLAVRVGLLGWKFIFV
NDLEVKSELPSQFKAFRFQQHRWSCGPANLIRKMTMEIIHNKRVKIWKKFYVIYSFFFLRKIVVHFFTYFFYCVILPTSV
FLPEVNIPNWSTIYVPSVITLLSAIATPRSFYLVIFWVLFENVMAMHRTKGTLIGLFEGGRVNEWVVTEKLGDTLNTKLL
PQNGRLPKRVNLKEMMMGIYILCCACYDFAFGNAFLYLYLFMQATAFLISGVGFVGT

>Arabidopsis_thaliana_[Mannosyltransferase_CSLA2] BLAST: Q9FNI7 Glucomannan 4-
beta-mannosyltransferase 2, CSLA2
MDGVSPKFVLPETFDGVRMEITGQLGMIWELVKAPVIVPLLQLAVYICLLMSVMLLCERVYMGIVIVLVKLFWKKPDKRY
KFEPIHDDEELGSSNFPVVLVQIPMFNEREVYKLSIGAACGLSWPSDRLVIQVLDDSTDPTVKQMVEVECQRWASKGINI
RYQIRENRVGYKAGALKEGLKRSYVKHCEYVVIFDADFQPEPDFLRRSIPFLMHNPNIALVQARWRFVNSDECLLTRMQE
MSLDYHFTVEQEVGSSTHAFFGFNGTAGIWRIAAINEAGGWKDRTTVEDMDLAVRASLRGWKFLYLGDLQVKSELPSTFR
AFRFQQHRWSCGPANLFRKMVMEIVRNKKVRFWKKVYVIYSFFFVRKIIAHWVTFCFYCVVLPLTILVPEVKVPIWGSVY
IPSIITILNSVGTPRSIHLLFYWILFENVMSLHRTKATLIGLFEAGRANEWVVTAKLGSGQSAKGNTKGIKRFPRIFKLP
DRLNTLELGFAAFLFVCGCYDFVHGKNNYFIYLFLQTMSFFISGLGWIGTYVPS

>Arcobacter_butzleri_ED-1_[Glycosyltransferase] InterPro PF00332-PF13641:
A0A0M1UPH9 Glycosyltransferase
MAGLFQVFFWLTQDNLITLKEDSFEKIESLSYSPYEGYNKNLLSKQQIYDDVNMLENFTNKLRTYATLEANAVLEATPDS
NMPIDLGLWISGDHEQNHLEIKRAIKLLEKYPNRINSVIVGNEVLLRADLDEVELFAYVDFMRQFTKKPITVAETWDVWE
RVPDLAKHVDFLTIHILPYWEKIPIDRFNEFIIEKYSLVEKIHPNRKIVIGETGWPSHGYNNRSAIPSLKNQAMAIRGFV
NLANEHGWHYNIIEAFDQQWKGYDEGNVGQYWGIFTSDRALKFQLNGDIELNQYWLYQMIAATIIGAIITLLGLRNQRVN
ISHALAYAVTAQGMAFGIVMAAIYPFINYMNFGMWVMWGMGTFLMIPLVIITLAKANELFRSSIGVPPSRLVPLDLKSQN
APLVSIHVPAYKEEPHVLEETLRSLSQLKYPNYEVLVIINNTPEEYYWKPIEKLCEELGDKFVFMNITCTGFKAGALNAA
LERTSKDAEIVAVIDADYKVESPWLVDLVPLFDDPKVAIVQAPQDHRDGKESIMKAAMNAEYAGFFDIGMVDRNEENAIV
VHGTMVMVRLSAMMEVGGWGTDTIVEDSELGLRLFEAGYIAHYTNRRYGYGLLPDTLEAFKTQRHRWAYGAIQILKKHWR
EFKPSANRLTPRQKNKFVTGWFFWLSDAMGPIMAVMNIIWVPVIIFVGVTIPTIPLTIPIITAFLVNILHTFILYRTKVK
ATFKEILLSSIASMSLQLIIFKAVFDGFIKDGLPFKRTQKGGKAKKSANPIKYESILAVLLLIAFFALIFTNKTGITEIY
VFAVTIFIQSIPYLSAIIMRMLEVYSIKHQKS
```

>Arcobacter_butzleri_7h1h_[Glycosyltransferase] InterPro PF00332-PF13641: S5P613 Glycosyltransferase, family 2
MRYIVLGLIMAGLFQVFFWLTQDNLITLKEDSFEKIESLSYSPYEGYNKNLLSKQQIYDDVTMLENFTNKLRTYATLEAN
AVLEATPDSNMPIDLGLWISGDHEQNHLEIKRAIKLLEKYPNRINSVIVGNEVLLRADLDEVELFAYVDFMRQFTKKPIT
VAETWDVWERVPDLAKHVDFLTIHILPYWEKIPIDRFNEFIIEKYSLVEKIHPNRKIVIGETGWPSHGYNNRSAIPSLKN
QAMAIRGFVNLANEHGWHYNIIEAFDQQWKGYDEGNVGQYWGIFTSDRALKFQLNGDIELNQYWLYQMIVATILGAIITL
LGLRNQRVNISHALAYAVTAQGMAFGIVMAAIYPFINYMNFGMWVMWGMGTFLMIPLVIITLAKANELFRSSIGVPPSRL
VPLDLKSQNAPLVSIHVPAYKEEPHVLEETLRSLSQLKYPNYEVLVIINNTPEEYYWKPIEKLCEELGDKFVFMNITCTG
FKAGALNAALERTSKDAEIVAVIDADYKVESPWLVDLVPLFDDPKVAIVQAPQDHRDGKESIMKAAMNAEYAGFFDIGMV
DRNEENAIVVHGTMVMVRLSAMMEVGGWGTDTIVEDSELGLRLFEAGYIAHYTNRRYGYGLLPDTLEAFKTQRHRWAYGA
IQILKKHWREFKPSANRLTPRQKNKFVTGWFFWLSDAMGPIMAVMNIIWVPVIIFVGVTIPTIPLTIPIITAFLVNILHT
FILYRTKVKATFKEILLSSIASMSLQLIIFKAVFDGFIKDGLPFKRTQKGGKAKKSANPIKYESILAVLLLIAFFALIFT
NKTGITEIYVFAVTIFIQSIPYLSAIIMRMLEVYSIKHQKS

>Arcobacter_butzleri_RM4018_[Glycosyltransferase] InterPro PF00332-PF13641: A8EUT2 Glycosyltransferase
MRYIVLGLIMAGLFQVFFWLTQDNLITLKEDSFEKIESLSYSPYEGYNKNLLSKQQIYDDVTMLENFTNKLRTYATLEAN
AVLEATPDSNMPIDLGLWISGDHEQNHLEIKRAIKLLEKYPNRINSVIVGNEVLLRADLDEVELFAYVDFMRQFTKKPIT
VAETWDVWERVPDLAKHVDFLTIHILPYWEKIPIDRFNEFIIEKYSLVEKIHPNRKIVIGETGWPSHGYNNRSAVPSLKN
QAMAIRGFVNLANEHGWHYNIIEAFDQQWKGYDEGNVGQYWGIFTSDRALKFQLNGDIELNQYWLYQMIVATILGAIITL
LGLRNQRVNISHALAYAVTAQGMAFGIVMAAIYPFINYMNFGMWVMWGMGTFLMIPLVIITLAKANELFRSSIGVPPSRL
VPLDLKSQNAPLVSIHVPAYKEEPHVLEETLRSLSQLKYPNYEVLVIINNTPEEYYWKPIEKLCEELGDKFVFMNITCTG
FKAGALNAALERTSKDAEIVAVIDADYKVESPWLVDLVPLFDDPKVAIVQAPQDHRDGKESIMKAAMNAEYAGFFDIGMV
DRNEENAIVVHGTMVMVRLSAMMEVGGWGTDTIVEDSELGLRLFEAGYIAHYTNRRYGYGLLPDTLEAFKTQRHRWAYGA
IQILKKHWREFKPSANRLTPRQKNKFVTGWFFWLSDAMGPIMAVMNIIWVPVIIFVGVTIPTIPLTIPIITAFLVNILHT
FILYRTKVKATFKEILLSSIASMSLQLIIFKAVFDGFIKDGLPFKRTQKGGKAKKSANPIKYESILAVLLLIAFFALIFT
NKTGITEIYVFAVTIFIQSIPYLSAIIMRMLEVYSIKHQKS

>Arcobacter_cloacae(2)[Glucosyltransferase] InterPro PF00332-PF13641: A0A6M8N7U2 Putative glucosyltransferase
MRYIILGLIMAGLFQVFFWITKDNRVSLVETLSEKIESLSYSPYKGYNKQVLSPREIEDDVNMLSHITNKVRTYSTMDAK
VILEEASKINLPVDLGLWISGDHKENHLEIERAIKLLEKYPNNIANVIVGNEVLLRADINEVELFAYIDFMREFTDKPIT
SAETWDVWERVPQLANHVDFLTIHILPYWEKVPIEQFNSFVIEKYNTVKKIHPDKKITIGETGWPSHGYNNNSAVPSLKN
QATAIRSFINLAQENDWSYNIIEAFDQQWKGYDEGNVGQYWGIFTSDRTLKFYLSGDIEINENWLYQMIAAIIIGALITL
YGLKNQRLNISHAFAYAIAAQGMAFGIVMAVIYPFTNYMNFGMWVMWGMGTFLMIPLIIITLAKANELFRNSIGIAPQRL
VPLDLKSDNIPFVSIHVPAYKEQPHVLEETLRALSRLKYPNYEVLVIINNTPEEFYWKPIEKLCEELGDKFVFMNITCTG
FKAGALNAALEQTNKEAEIIAVIDADYVVESSWLVDLVPLFDDPKVAIVQAPQDHRDGNESIIKAAMNAEYAGFFDIGMI
DRNEENAIVVHGTMVMVRLSSMMEVGGWGTDTIVEDSELGLRLFEAGYIAHYTNRRYGYGLLPDTVEAFKTQRHRWAYGA
IQILKKHWKEFKPSANKLTPRQKNKFVAGWFFWLSDAMGPVMAVMNIIWVPVIIFVGVTIPTIPLTIPIITAFLVNILHT
FILYRMKVKATLKDTLLSSIASMSLQLIIFKAVFDGFVKDGLPFKRTQKGGKAKKTNDNPIKHETILAVLLLTSFFALIF
TNHSGIVEIYVFAATIFIQSIPYISAIIMRRLELYSIKNQKS

>Arcobacter_ellisii_[Glucosyltransferase] InterPro PF00332-PF13641: A0A347U9G5 Putative glucosyltransferase
MRYIILGLIMAGLFQVFFWITQDNRVSLSDDSFDKIESLSYSPYEGYDKKMLSYQQIENDVNMLVHFTNKVRTYAASEAE
AILEVTSKTDMPIDLGLWLSGDLKENFKEVERAIKLLEKYPDNIANVIVGNETLLRADINEVELMAYIDFMREYTDKPIT
TAETWDVWERVPEIANHVDFLTIHILPYWEKIPIELFNSFVIEKYNVVKSIHPNKKIAIGETGWPSHGYNNRGAVPSLKN
QAMAIRGFINLAKENGWTYNIIEAFDQQWKGYDEGNVGQYWGIFTSGRELKFSLNGDIELNQYWLYQMIAAIIIGALITL
YGLRNQRLNLNHAIAYAIAAQGMAFGIVMAVIYPFTNYMNFGMWIMWGMGTFLMIPLVIITLAKANELFKCSIGTPPTRL
VPLDLRSENAPFVSIHVPAYKEQPHVLEETLRALSRLKYPNYEVLVIINNTPEEFYWKPIEKLCEELGEKFVFMNITCSG
FKAGALNAALEQTDKRAEIIAVIDADYVVESPWLVDLVPLFDDPKVAIVQAPQDHRDGDESIIKAAMNAEYAGFFDIGMI
DRNEENAIVVHGTMVMVRLSAMMEVGGWGTDTIVEDSELGLRLFEAGYIAHYTNRRYGYGLLPDTVEAFKTQRHRWAYGA
IQILKKHWKEFKPSSTKLTPNQKKKFVAGWFFWLSDAMGPVMAIMNIIWVPVIIFVGVTIPTIPLTIPIITAFLVNILHT
FILYRMKVKTSIKNTILSSIASMSLQLIIFKAVYDGFVKDGLPFKRTQKGGKTAKKSANPIKHETILGVLLLASFFALIF
TNKTGITEIYVFAATIFIQSIPYISAIIMRFLEVYSIKNNQKS

>Aromatoleum_aromaticum_EbN1_[Glucosyltransferase] InterPro PF00332-PF13641: Q5NZ95 Putative beta-(1-3)-glucosyl transferase
MKYAASLIYRLIVALILAGLVAGAQYLFAERWNRGTEFIGAGNSIRGYAYSPFQRDQSPLKGTYPNEAEIAADLDLLAQT
GERIRTYGSTEVPAIVRLAGERRLTVTAGAWLSPDPEANDREIDALIESAREMRHIERVIVGNEVLLRGDLSVAELSTYL
DKVRKALRRPKVPVSTAEPWHVWLKHPELAKSVDFITVHLLPYHEGVPVESAVEYVLMRYDELAKAFPKKKIVIGEVGWP

SRGPVMNSFGAEETTSVPSVENEARFIREFLAHPRSPTLDYFIMEAIDQPWKIQLEGWAGAYWGMFNAERQAKFPLEGLV
VQDIRWHEKARMAALIALVPMFLICFLLRDWSILGRLWLSALIQACAVTLVIGANVPADYYLTQRDLIGLAMLIGATVLT
IAVLLSHGFEFGEVLFKRRWKRRFLPLTPLAAEREPFVSIHLACCNEPPEMVIATIDSLAAMNYGNFEVLVLDNNTKDEA
LWKPLEARCAELGSRFRFFHLENWPGFKAGALNFGLKQTDPRAEVVGVVDADYVVSPDWLSCLIPHFDAADVAVVQAPQA
HRDWETQPFRRMCNWEFEGFFRIGMHHRNERNALIQHGTMTLVRRLALEEVGGWSEWCICEDTELGLRLIEKGYDTRYVD
HILGRGLTPSDFAAIKSQRFRWAFGAMQILKHHLPAMIGPSRLNIAQRYHFLTGWFAWLGDALQLVFVFASLAWTIGMLY
MPQEFGLPVSALALPILVFMAFKGGLGPILYRRTMDAPWKDILGASILSVGMAHAIARGVFAGLVKKRGEFVRTPKGWKD
KGTLAFFSPIREEIGLLLALVLGSVVLVWLRGAQDLEAQLWVGILALQCIPYLAAIACQAASYMPERPATTVEPAPETRP
SASVTPIEEASVTPIVEVEAMRAGG

>Aspergillus_clavatus_ATCC_1007_[Glucosidase_BtgC] BLAST: A1CAI0 Probable glucan endo-1,3-beta-glucosidase, BtgC
MSGPHRTFSFEQRGDGEAHSSFMDHAMHPQYDDVSPISNMSSSPGHMNETHHGLASVPEDNHQGWGQARGPSPSNRTGFT
ATPEMDNLGPASVGGGISGIALGVANSHDRLSGVEARRGTDGQEANIPAERGYNTTGSDNPYIPAPPDMGGYGSSETLHP
RQSYGSNVALGAAAGPAGQLTPGHSATHLGTSNSSQRNLYDAPYQSAGGLSAGPYQRHSAYSSNDLPLDINPEEIADDGD
DGFAPAGNSRSSARRSQAVPAAAGGAAAGGVLGGIGGLFNNRNPAETSYDPVPGAGLEAGEKSQWVKPKPSTGSRKRGWI
IGAILAVIIIGAIVGGAVGGTIGHKDSGDSASGSSASTQSASGDTDTNGDLDKNSAEIKALMNNKDLHKVFPGMDYTPWG
VQYPLCLKYPPSQNNVTRDMAVLAQLTNNVRLYGTDCNQTEMVLHAIDKLDLKDMKVWLGVWIDTNETTSRRQIDQLYKI
VDDAKDISIFNGAIVGNEALFRAGDNKITAQATLTKYMQEVRDHFKKHDIKMPIATSDLGDNWNAELVQIADVVMSNVHP
FFGGIPVDQAAAWTWRFWQDHDVILTQGTDKRQVISEVGWPSGGGNDCGKGANCPDDTSGAVAGIDELNKFMEDWVCQAL
DNGTDYFWFEAFDEPWKIEFNTKNENWEDKWGLMDPARKLKSGLKIPDCGGKTAA

>Aspergillus_clavatus_ATCC_1007_[Glucosidase_BtgE] BLAST: A1C499 Probable beta-glucosidase, BtgE
MRGAFLATAAAIAGTAMADIAHMRRHGHDSFHQRRAVEQPAPEADATCGCTTEVVTSWGPPTLIPIATSSPSSTVTSEVV
TTLHSTSYSTVTLVVTPSGASPNRESAPATPAVTLPTPGVTSFSTTGTYTIPATTLTVTHSTTVCGATTTELPSGTHTYG
GVTTVVDRHTTVVCPYATVEPSGSTVTSVIRTTTYVCPSAGTYTIAPTTTYVPTSTVIVYPTPATITPGTYTQPAQTITV
TRDNYIYVCPFTGQQLPTTAPVAPATTAVPATTTAVPATTTAVPATSSVAPSSSPSKPAAPSGAVSGQMGMTYSPYTNEG
GCKDKASIISEVALLKSKGFTHVRVYSTDCGSLEFIGEAARTSGLRMIIGVFIKQSGVAGAQDQVTAISKWAQWDLVSLI
VVGNESIQNHFCDASTLAGFIVSAKQSFKAAGYSGQVTTTEPINVWQANGDALCGAVDIIGANIHPFFNADVSAAEAGKF
VAQEFKTLKGICPGKDVINLETGWPHSGEANGKAIPSREEQAIAIKAIADEVGSMSVFFSYFDDLWKQPGAFGVERYWGC
IENF

>Aspergillus_flavus_ATCC_200026_[Glucosidase_BtgC] BLAST: B8NTP7 Probable glucan endo-1,3-beta-glucosidase, BtgC
MSGPHRSFSFNQGDDGAGDAGDVSPIRSQEGHFMNSPPRHNDVSPVSARSQAMGSSPSSGFLSAHEHGDRGWGQNSGHTQ
AMRTNSTTPGMDNLGPAAVGGGISGIALGVANSHNRQSGIDAFRDTDGRNLPAERGYNTTGSDNPYVPTPPGGGSHGSAE
NLRPRDSYGSNVALGAAAAPAGQLTPGGSNPSQRSLFDSPYQGVGAMDAGPYQRQSAYSAAGDYPLVINPDEIADDGDDG
FTPVPNGKSASSNARAIPAAAAGGAAGGGLFGLFKSKKADNPSYGPVPGAGLEAGEKSRWVKPTPGGGSRKRGWIVGLAL
AFIVVGAIVGGAVGGTLGNRENEAPDTTKSASSDTESNGDLNKDSSEIKDLMNNPDLHKVFPGMDYTPWGVQYPLCLKYP
PSQNNVTRDVAVLSQLTNTVRLYGTDCNQTEMVLHAIDRLELKDMKVWLGVWIDSNDTTNDRQIKQLYKVLDDTKDISIF
KGAIVGNEALYRAGNDIASAKKKLISYMDDVRNHFKEKNYDLPIATSDLGDNWKEDLVTATDLVMSNVHPFFAGVTAKEA
AGWTWNFWNQNDVPLTKGTNKKQVISEVGWPSGGGNDCGSNNKCTDDTSGSVAGIDEMNQFMSDWICQALENGTDYFWFE
AFDEPWKVQYNTKDENWEDKWGLMDAARKLKPGLKIPDCGGKTAA

>Aspergillus_flavus_ATCC_200026_[Glucosidase_BtgE] BLAST: B8MXP5 Probable beta-glucosidase, BtgE
MRGAFLAAAAAVAGTAMADVAHMRRHGHDSFHHNRAYQPEVPAEGDENCECTTKVITITGPPTLVPINTPAPEPSSSSSS
EVPSVPSSESSVVTSEAVTTLHSTSTATVTVVTTPGVDATGAQTPTGGVPGTPEASSPAGTPEASTPAVPATSESPLPTP
GVTSFSSTGIYTIPATTVTVRDTTTVCGATTTELPSGTHTFGGVTTVVSTATTVTCPVATVEPSGSTVTSKIYTTTYVCP
SAGTYTIAPTTTYVPTSTVVVYPTPATITPGTYTQDEQTVTVTRTDFTYVCPFTGNDQPTSAPVASTSAVPVTTTAAPST
TSAVASSSASASSTATAVPTGVSGQQMGMTYSPYTNEGGCQSKDQVLKDVALIKQKGFTHVRVYSTDCNGLEYIGEAARE
NGLKMIIGVFISSTGISGAQEQVTAITKWAQWDLVTLVVVGNEAIQNGYTDASSLAGFISSCKSSFQASGYSGQVTTTEP
INVWQQSGSALCGAVDILGANLHPFFNADVTPDQAGSFVRAQIKDLEAVCNKDVINLETGWPSAGNANGKAVPGTAQQAA
AIKALVEEVGSQSVFFSYSNDLWKDAGEFDVERYWGCIDQFK

>Aspergillus_oryzae_ATCC_42149_[Glucosidase_BtgC] BLAST: Q2U492 Probable glucan endo-1,3-beta-glucosidase, BtgC
MSGPHRSFSFNQGDDGAGDAGDVSPIRSQEGHFMNSPPRHNDVSPVSARSQAMGSSPSSGFLSAHEHGDRGWGQNSGHTQ
AMRTNSTTPGMDNLGPAAVGGGISGIALGVANSHNRQSGIDAFRDTDGRNLPAERGYNTTGSDNPYVPTPPGGGSHGSAE
NLRPRDSYGSNVALGAAAAPAGQLTPGGSNPSQRSLFDSPYQGVGAMDAGPYQRQSAYSAAGDYPLVINPDEIADDGDDG

FTPVPNGKSASSNARAIPAAAAGGAAGGGLFGLFKSKKADNPSYGPVPGAGLEAGEKSRWVKPTPGGGSRKRGWIVGLAL
AFIVVGAIVGGAVGGTLGNRENEAPDTTKSASSDTESNGDLNKDSSEIKDLMNNPDLHKVFPGMDYTPWGVQYPLCLKYP
PSQNNVTRDVAVLSQLTNTVRLYGTDCNQTEMVLHAIDRLELKDMKVWLGVWIDSNDTTNDRQIKQLYKVLDDTKDISIF
KGAIVGNEALYRAGNDIASAKKKLISYMDDVRNHFKEKNYDLPIATSDLGDNWKEDLVTATDLVMSNVHPFFAGVTAKEA
AGWTWNFWNQNDVPLTKGTNKKQVISEVGWPSGGGNDCGSNNKCTDDTSGSVAGIDEMNQFMSDWICQALENGTDYFWFE
AFDEPWKVQYNTKDENWEDKWGLMDAARKLKPGLKIPDCGGKTAA

>Aspergillus_oryzae_ATCC_42149_[Glucosidase_BtgE] BLAST: Q2US39 Probable beta-glucosidase, BtgE
MRGAFLAAAAAVAGTAMADVAHMRRHGHDSFHHNRAYQPEVPAEGDENCECTTKVITITGPPTLVPINTPAPEPSSSSSS
EVPSVPSSESSVVTSEAVTTLHSTSTATVTVVTTPGVDATGAQTPTGGVPGTPEASSPAGTPEASTPAVPATSESPLPTP
GVTSFSSTGIYTIPATTVTVRDTTTVCGATTTELPSGTHTFGGVTTVVSTATTVTCPVATVEPSGSTVTSKIYTTTYVCP
SAGTYTIAPTTTYVPTSTVVVYPTPATITPGTYTQDEQTVTVTRTDFTYVCPFTGNDQPTSAPVASTSAVPVTTTAAPST
TSAVASSSASASSTATAVPTGVSGQQMGMTYSPYTNEGGCQSKDQVLKDVALIKQKGFTHVRVYSTDCNGLEYIGEAARE
NGLKMIIGVFISSTGISGAQEQVTAITKWAQWDLVTLVVVGNEAIQNGYTDASSLAGFISSCKSSFQASGYSGQVTTTEP
INVWQQSGSALCGAVDILGANLHPFFNADVTPDQAGSFVRAQIKDLEAVCNKDVINLETGWPSAGNANGKAVPGTAQQAA
AIKALVEEVGSQSVFFSYSNDLWKDAGEFDVERYWGCIDQFK

>Azoarcus_strain_DN11_[Uncharacterized] InterPro PF00332-PF13641: A0A3S7UEI7 Uncharacterized protein
MAAGAVLSCMRVINPASEWITPFALSLSKGPVQTGSARTVSGCRVNNAVMYPSHSDGSSPFAAGPHLRSPLAFLAALGRL
FVDLGLALSACAIVLVAWSALERPITPPDWDGRVPGVTYSGYRPGQGPAQQRYPGAEEVAQDMALLAPHTRRVRTYSSTE
GPDVPEIAARHGLDVMAGAWLGKDRAKNERELQGLMRQASEHRNVSRLIVGNEVMLREDLAEAELIAALDRVRRSTRKPV
STADTWHMWLQHPKLAAHVDFIAIHILPYWEGVKIEEAAAYTLERLKRVKAAYPNKPVVITETGWPSHGDVQWDAIPTPQ
AQARYLREFIARARSLGIDYYVIEAFDGQWKRDEEGRPGPYWGLFDAFRQWKIPTEGPLWVHQGWQDRVVPALGCVLALG
VLLSWRFRRWHWWARLSAVLAVAAGTSFVLWRGMAGVGSYAPVQSTLIDAALAGLLLVSLLVFAVQLIEALDVLGTRRWR
RAFAATPWPDERPVPMVSLHVAISNEPPDMVIATLESLRRLDWPALEVLVIDNNTANEALWRPVQAWIAQHPERFRFWTL
PACEGFKAGALNFALAQTDPHAEVIGIVDADYQVEPFWLREVMGHFASPEVAVVQAPQAHRDYESDLLARSANWEFEGFF
RAGMHHRNERNAIIQHGTMCLVRTSALRDAGGWGQWTLCEDTELGLRLLTRGWELRYVDRVYGRGLTPENFAALRSQRRR
WALGAMQILKGHAGALCGRSQLTLAQRYHFVAGWLPWLQEALQVAVMLTSVCWTLGMLLWPRYIEPPIPGTLALMLAVVL
GRTAIGAAVYATKVRCTWRESLEAAIASMALNYAVACGVWAGLLGRHARFIVTAKAGASARRGRRRLPPEAKWALALLVA
AIATLIQNGVDAPEPLCWAVALVVMALPHCAALWLSFAPRGGRPGPHPVGASQGKSLPAATDCALPSSTGQ

>Azoarcus_strain_KH32C_[Glucosyltransferase] InterPro PF00332-PF13641: H0PW10 Putative glucosyltransferase family protein
MRTGTGFIAFERLLSAFVLALVACAAVWGAWHVLERPFTPPEWDGQLSGLTYSGYRPGQGPAQHRYPSPDEIAQDMALLA
PHTHRIRTYTATEGPDVPALAARYGLDVMAGAWLGKDKAANEREVKALIRQARENANVSRLIVGNEVMLREELTEAQMIA
YLDRVRRQTRQPVSTADPWGIWLEHPKLAAHVDFIAIHVLPYWEGVAIEEAVAYTLAQLQRVQAAYPNKPIVITETGWPS
RGNVQSEARPTPQAQARYLREFIPRARALGIDYYVIEAFDGQWKRDEEGRPGPYWGLFDAFRDWKIPLEGTLWVDQGWQD
RVVPALVVALLLGTLWNWVFAHWRWWARLLAVLALAAGASFVVWRSLADAGSYAPIQSPFIELVLNGLLGVSLLVFTVQL
VEALDVLGTHRWRRAFGASPWPAEKPLPLVSLHVAICNEPPEMVIATLESLERLDWPALEVLVVDNNTRDDRLWRPVAAW
IAERPERFRFWSLPVCEGFKAGALNFALSQTDPRAEVLGVIDADYRVEPDWLRDLMGHFTAPQVAVVQAPQAHRDFEADL
LARSANWEFEGFFRAGMHHRNERNAIIQHGTMCLVRAAALREAGGWAQWTICEDAELGLRLMTRGCELRYVDRVYGRGLT
PENFAALRSQRRRWALGAMQILKGHVGALFGRSPLSWGQRYHFIAGWLPWLQEALQVGVVLMCVAWTAGMLIAPRYVEPP
IPGTLALMLAIVLARAAIGAAVYATRVRCTWRESFEAAIAAMALNYAVASGVWAGLLGRHARFIVTAKAGTRTGRQPLPP
ETKWAVALLAAAVATLIQNGFGGPEPLCWATALVVMALPHCAALWLSRASRPGRPSMQPAPAAGVDSVPGLTSPA

>Azospirillum_strain_RU38E_[Glucanase] InterPro PF00332-PF13641: A0A239AN08 Exo-beta-1,3-glucanase, GH17 family
MLCSSVKDHDCAFHKNNKKRLQIVEPPPFLAGNVPRGLIGKMGKILKMRGSAWLVLALLVLGNIGAWALSNRPTTPDRLW
AGTIAGVAFSPYQENQNPQDGRHPSRADMERDLEVVAPYVRSIRTYTATDGLEVIPELAAKHDLDVTAGAWISGDLGKNE
QEINSLVRLTRANPNVKRVMVGNEVMYRGDLEVGALIDYVQRVKKQLDVPVSTAEPPYVWIKHPELAEAVDYITIHLLPY
WEKVPIEQAMEQVRRGYDEVRAKYPDKHILVGEAGWPSDGPYRGGAQASLVNEATFIRNFLNLAGENRWDYYIMEAFDQP
WKREIGGEAEANWGIWDTHRQMKFPMEGGVFEVPGWPVLCAIATALAFLPMVWFVLQRDDLKPAGQLFYGALIQGIASGL
VWTATEAMTTGMNTMSFIAFLVLIFAQILLLVVVLIDGLELTEVVWTDRWKRKFAPVKANPNNYYPKVSIHVPCYNEPAH
MVIETLNALAKMDYPNFEVLVLDNNTKDEAVWKPLEEHCAKLGPRFRFFHLAKWPGYKAGALNFGLAVTAPDAEVVGVID
SDYQVTPDWLSSTTPYFENPKVAFVQSPQDYRDWHIHPFHRMINWEYQGFFHIGMVQRNERNAIIQHGTMTLIRTSILKQ
VGWWAEWCICEDAELGLRLFEEGLESVYMPDSFGKGLVPDSFAGYKTQRFRWAYGAVQIIKRHWREFLPSGKKLTFGQKY
HFVTGWLPWFADAAHMGFVIGGVFWSLGLLLAPRYFEFPPTVFIMPTLGVFFFKVACGLWLYAARVKCGFLDKVNAAVAG
MALTHTVGRAVWTGLFTQGRPFVRTPKCEDQPALIQGFLHAKEEVMLMVALWITAFAVGWREGGDNRDAYIWSAMLVVQS
LPYIAALAVATINGLPMPKQDEPQPAPAAE

>Blastochloris_viridis_[Glucosyltransferase_PgaC] InterPro PF00332-PF13641: A0A0H5BJI8 Beta-(1-3)-glucosyl transferase, PgaC
MLPSSVKAEYKGLADNENRRRFSKSTFALVLAVMAMVATVHGALWMWAQETMSAPAAINKFPSLSFAPYDPKSDPESGGV
TNEAQIRSDMKVVAPYTRAIRTYSSTGGLEYVPSVAAEHGIKVSMGAWVDKSDDRNERELRAVVDLARKNSNINAIVVGN
ETILRAEQTVDELIKKIQRVKRETSGIPVTTGEIWNIWLEHPELVSAVDFIAVHILPYWEGHPETSAVDQAIIIYNKLRA
AYPGKRIVIAEFGWPSGGYNLHRAQPGPMIQAQVIRDFVARAEAYGIDYNIVEAFDQPWKTNEGSVGCYWGLFDGDRNLK
FNLAGDITNTRANTNTIIALVIGALLSLTALKMVRPTFGHVAMRVAAANAVGAWLAVVFDYWVTHYFVTGAMFSMALGFI
LLIPLVFVAMARIDELAAIVFGRAPRRLLAKPAHDPVRAPKVSIHIPAYREQPEMLKATLDAVARLDYPNFECVVIINNT
PDPACWQPIEAHCKLLGERFKFLNEPKVQGFKAGALRIALAHTDPEAEVIGVIDADYVVHPDWLKDLVPAFEDPRVGIVQ
APQDHRDGKRSVMHEMMNAEYAGFFDIGMVQRNEHDAIVVHGTMVLIRRTALKAGGDWSSDTICEDTDLGLCLLEQGWSV
HYTSRRYGWGMLPDTYEAFRKQRHRWAYGGVQIVKKHWRQMWQPEATRLTGDQRREFLMGWFNWLGAESVGVLMAILNIL
WVPVVAFGGIAVPERILTLPIVATFVIYLVHFMWLYRQRVGIGPVRSIGAAIAAMGMQFTVAKAVADGVVKDNLPFNVTA
KGGGKAKKGSDFAAFWETVIAGLLLLGAAVLHMTNYHQVNEINIFKWVLVVQSLPFLAAAGMAALERTPLNDFALWRRLV
ERIHAALPRRTADVGVPPKVSAD

>Bradyrhizobium_strain_BTAi1_[Glucosyltransferase] InterPro PF00332-PF13641: A5EJ90 Putative beta-(1-3)-glucosyl transferase, NdvB-like protein
MRVIAAVILFVAAAHAALWGLLQQKQSAPDFTGMLPSVSYAPFEGTDHPDVDNIPNAERIRSDLKKLATITRAIRLYSST
GGVELVPPIAAEVGLKVTLGVWIDKNEDRNKREIAAAIQLARRNSNVIGVVVGNETLYRGELKPDELIGYIKMVKKSVTV
PVTTGEIWNLWRDYPQLASNVDFIAAHVLPYWEFFNHTQAVDQAVDRYQLLREKFPGKRIVIAEFGWPSEGYNRGIADPG
PFQQAWVLRNFVTRAEAIGMEYNIVEGIDQPWKFFEGGVGPYWGVLNAAREVKFAWTGPIVNPDYWKLAAIALLVGVLLS
LPLVRLRQPTVLQALVLAITAHGVGAWVSNVFAYWNVHYFVWGSAFALTLGLTLLVPLILIAMARIEEIAAIAFGRAPRR
LLTRDKAARARAAAPDYCPKVSIHVPAYFEPVEMMKQTLDALARLDYPNYEVVVIINNTPDPAFWQPIQDHCRMLGERFK
FINAEKVKGFKAGALRIAMERTAVDAEIIGIIDADYVVTPDWLKDLVPAFADPAVGLVQAPQEHRDEDLSLMHYIMNGEY
AGFFDIGMVQRNEENAIIVHGTMCLIRRAAMDMAGGWSSDTICEDTDLGLAIQELGWQTHYTATRYGAGLLPDTYEAFKK
QRHRWAYGGFQIVKKHWRRFLPGRSRLTADQKREFSLGWLNWLGAESLGVVVAILNLIWVPIVAFAGIAIPDKILTIPII
AAFVVSLAHFLILYRLRVAVKPWQMLGAMIAAMSVQWTVSRAVAQGLITEHLAFARTSKGGLSRMSIEFQAFWEAVIGAL
LLIGATILIMTNYLAITELYIFAAVLILESLPFLSAVAIAVLEMSRINSFEFWRHATVRTAELIGLRPVWVPELAGPGMQ
PAALSAAPTKAVEKV

>Candida_albicans_SC5314_[Glucan_glucosidase_Bgl2] BLAST: Q5AMT2 Glucan 1,3-beta-glucosidase, Bgl2
MQIKFLTTLATVLTSVAAMGDLAFNLGVKNDDGTCKDVSTFEGDLDFLKSHSKIIKTYAVSDCNTLQNLGPAAEAEGFQI
QLGIWPNDDAHFEAEKEALQNYLPKISVSTIKIFLVGSEALYREDLTASELASKINEIKDLVKGIKDKNGKSYSSVPVGT
VDSWNVLVDGASKPAIDAADVVYSNSFSYWQKNSQANASYSLFDDVMQALQTLQTAKGSTDIEFWVGETGWPTDGSSYGD
SVPSVENAADQWQKGICALRAWGINVAVYEAFDEAWKPDTSGTSSVEKHWGVWQSDKTLKYSIDCKFN

>Crenothrix_polyspora_[Glycosyltransferase_GlGT) InterPro PF00332-PF13641: A0A1R4H746 Glycosyl transferase family 2, GlGT
MITKIATLLTLVLLVVINFSIWSYINYPLQFQPWTKTTMGVTFDPMRKDDTQKTKIFHDPVEIEADLALLANKVHAVRTY
SVLKGLDKVPELAAKHGLNITVGAWIGGDLEKNRQEIETLIKISREDNPKIVRVMVGNEVLLRNEITEDLLIDYIREVKK
RTWRPVSTSETWDKWLIHPELVKEVDFIGVHILPYWEGIAANEAVDYVFDRYNTLKQAYPNKPIIITEVGWPSDGQPFKH
ATASRANQARFLREFLNRAAKEQIAYYVIEAFDQPWKMSDEGSAGAYWGIFDAERKAKYSMDGVVFSLPAWEHWAVGAAL
FSVVLMGLFLFTRTNLKLPGLLFFGLIANLAASVIFWTLSIGAQQYQTPVTIVFWVLLVLMQMLAAVILLIESLEIAEVV
WHRKTARTFTPLEPTSEFKYPKVSLHLPIHNEPPMMVRKTLEALDRVDYPNLEVLVMDNNTKDPAIWEPVREDCQRLGAK
FRFFHLENWPGFKAGAINHALEQTASDAEIIAVIDSDYMLSPDWLKCMVPYFDNDNVGFVQSPQDYRDRHLNTFKSFCYW
EYAGFFNIGMVQRNEYNAIIQHGTMTMIRKSALQEVGKWAEWCICEDSELGLRLYEAGYDSVYCKESFGQGVMPDTMSGY
MTQRFRWVYGAMQIIKGHWRSFLPTKKSPLTSAQRYYFVAGWLPWFSDALALLFTTTSLILTAVLLYDPLHSELPVNAFL
LPTIGLFSFKIIRSLWLYQARVPCSIWQSLGASLSGLSLTHTVARGTIQGLFTNGKPFMRTPKFEKQGALVAGLLTIRQE
LLLLTLLGTAIWFMYHLEHFDNLSGRLWIAVLSVQSVPYLAALITLLVSIAPAYGLGGKALAEEDDV

>Cyamopsis_tetragonoloba_[Mannosyltransferase_ManS] BLAST: Q6UDF0 Glucomannan 4-beta-mannosyltransferase 1, ManS
MRNLIFEEPEGIPGNSSSSLRYAWQSIRAPVIIPLLKLAVIVCSVMSIMLFVERVAMAAVILIVKVLRKKRYTKYNLEAM
KQKLERSKKYPMVLIQIPMYNEKEVYKLSIGAVCGLSWPADRFIVQVLDDSTNPVLRELVEMECQKWIQKGVNVKYENRR
NRNGYKAGALKEGLEKQYVEDCEFVAIFDADFQPDADFLWNTIPYLLENPKLGLVQARWKFVNSEECMMTRLQEMSLDYH
FSVEQEVGSSTYSFFGFNGTAGVWRIQAIKDAGGWKDRTTVEDMDLAVRASLHGWEFVFVGDVKVKNELPSTFKAYRFQQ
HRWSCGPANLFKKMTKEIICCKRVPLLKRLHLIYAFFFVRKIVAHWVTFFFYCIVIPACVIVPEVNLKKQIAIYIPATIT
ILNAVSTPRSMHLLVLWILFENVMSLHRTKAAIIGLLEANRVNEWVVTEKLGNAMKQRNNARPSRASRFRIIERIHPLEI
IVGMYMLHCATYDLLFGHDHFFVYLLLQAGAFFTMGFGLVGTIVPT

>Desulfofustis_glycolicus_DSM_9705_[Glucanase] InterPro PF00332-PF13641: A0A1M5V603 Exo-beta-1,3-glucanase (GH17 family)
MTRINTFICVTVALVSLCCWALLNRPEIEPPWPDRIQGFSFSPLRIGHNPVKQEAPSIAEIDSDLQLLAGQTNAIRTYSV
DGILQHVPSLAQKYGLNVTLGAWISDDLEENERQLETVIYLARQHYHNVIRVIVGNEALLRGDVSTARLHGYLDRVREAL
DIPVSTAEPWHIWQQQPDLADHVDFIAVHLLPYWEGIHLDRAVDHVIDRHDMLKLTFPEKEIVITEVGWPSNGRIRQDAV
ASTANQAAFLRRFLDRAERLDLVYYVLEAFDQPWKRTSEGTVGAYWGVYDADRRPKFPFTTPIVNIPKWHILAAISVALS
LLVSTLLLIDSRSLRKRGCGFLAAVAVLCASGAVWIVYSYWRQYMTPGALTIGALMLIGLTGVLVVLLAEAHEWAESLWG
GVGRRFSPPITVTDDLLPKVSIHVPAYNEPPQMLIETLNALSELDYPDFEVIVMDNNTKDPSVWQPVREHCLTLGERFRF
FHEDRLTGFKAGALNYALARTAADTSVVAVIDSDYVVKRNWLRDLAPHFLKPQVAIVQAPQDYRDDRDNLFKTMCYAEYR
GFFCIGMITRNERNAIIQHGTMTMVRKAVLTEIEGWAPWCITEDAELGLHIFEKGYEAIYVPTSYGKGLMPDTFIDFKKQ
RYRWAYGAVQIMRQHARTLLGSSDSRLTRGQRYHFIAGWLPWLADGINLLFTLAALAWSAAMIQAPLRFEPPLLMISLIP
LSLFFFKLAKMIYLYRRRVNASAVQTIASALAGLSLTHTIAKAVLFGLVSSNLPFFRTPKRAAAGTIRYALQAVREEGLL
ATALLAAIYGIIVRQGVESPDLLLWVMVLLVQTIPYLAAVLVSFIAAGTPAPTSLIATLAKPISVTGNRQEGSTYAPLHE
R

>Desulfovibrio_mexicanus_[Glucanase] InterPro PF00332-PF13641: A0A238ZUM5 Exo-beta-1,3-glucanase, GH17 family
MRKNALIVLIVFAVNLGLWAWFNRPAEQQLDFTGQVKAVSFSPYRADQDPLVNKFPTAQQIEEDIVFLKGKAGAIRTYSS
MDGMEQIPALADKHGMPVIAGAWLDKRMKRNETEMAGLIKNLHEHKNVTRAIVGNESILRGDFKPAALAEYIRRVKEAAP
HVQVSTAEPWHVWINNPELADSVDFIAIHILPYWERVPEEKAIEWSMHRYNQVVKRFPNKHVLVAEIGWPSNGERWGKAK
ASIANEAKFLRQFMNLAVQQNIDYCVMEAFDQPWKRPLEGVVGAHWGIWTVDRENKIPLVGPILEDSLWPVQWGLSLLSL
IPIGFFLYKRKDQPFQGRLFFCLLMQTVVSSMVWIVFTPITVEFLPAETAAWAILLPMQIGLLAVVLINGFEMSEMLWPE
GLKRRFFPLRHDADEGLRKVSIHLACCKEPPAMVVETLNSLAALDYPDFEVLVVDNNTHDPALWKPLEEHCAKLGPRFRF
FHLEEWPGYKAGALNFALKNTAPDARIIAVVDSDYQVKPGWLRSLTPYFDKPDVAIVQSPQDHRAWEGEPYKTVCAWEYD
GFFRIGMVHRNERNAIIQHGTMTMIRKAVMDEVGGWGEWCICEDAELGLRVFERGYQAVYVPESFGQGLTPDTYAGFKSQ
RFRWAYGAVQILKRHWRQLMPWNKTTGLDNGQKYHFLAGWLPWFADATQIAVLVVSLVWSLGMLALPRYFGTPLSIFLLP
PLGAFLFKLFHFMWLYKTRVKCGLWERIGAAVAGLALTHTIAKAIFQGLTTKNKPFLRTPKCEERSAVVRGLMMAREELF
VLAALWVAAGGVIARYGTENPDIVTWAVILVVQSAPYLAALCLSLLSVMPNLIPAIARKAAPQCGCELAPAAAQAAGLAS
IAKDRVKN

>Dokdonella_immobilis_[Glucanase] InterPro PF00332-PF13641: A0A1I4WTB8 Exo-beta-1,3-glucanase, GH17 family
MLPRCFALRASHHGFPLINTRSEVVEKSFWRAVLFAVTIAALNFGLWAFLNRPMQVPDWSGRVEGMAFSAFQRYQDPTKD
LFPSETELASDIRLLSQHTKRLRTYSSVESPQIPRLAAFYDMEVMAGAWIDKRMRNNEAELEALVALSRKHPNITRAMVG
NETILRGDVSVEQLINYIDRARAQLKIPVSTAEPFFVWERNPQLADHVDFISVHLLPYWEKIPRKDAINFTLGQYNRLRE
LFPGKPVVIGEIGWPSNGDRKEYAQPSIENEAQFLREWFNVAEREHIDYYVMEAIDQPWKEDIGGRAEAYWGMFNAAREP
KFSLTGKVTEDPTWPIKAICASLLALLPMFWFARHFMRFYVSGILFFLGLIQLSASVVVWSVSVPMAFYLSPFDWTMFLL
LVPAQLAIILVLLINGFEFTEVLWRPRWLRHYGLLEPSPPAAQPFVSIHLACCNEPPEMVILTLDSLAALDYENYEVLVI
DNNTKREEVWKPVEEYCARLGARFRFFHLNPWPGFKAGALNFGLEQTDPRAEVVAVVDADYVVRRDWLSALTGHFGDPKV
AVVQCPQAHREFEDNAFRRMTAWEYDGFFRIGMHHRNERNAIIQHGTMTMVRKDLLANTGCWSEWTICEDAELGLRLMHA
GHELVYVDELMGKGLTPADFTAYKSQRYRWAFGAMQIMKARFGWMTRKDSPLSRGQKFHFLTGWFSWFADALHLVFTMMA
IIWTIGMVGWPEKFTLPMELFLIPIIGFIISKAVFGIVLYRKRVPCSWYDTIMASIASMGLSHAIARGIFLGLWKKKGEF
VRTAKSRRLSSKPSAFSSVREELLMFIALMGCVVGMVTSNGITYTEGKLWVAILAAQAIPYASALIGAWVAHRSNDRAE

>Escherichia_coli_MG1655_[BcsA] Reference protein: P37653 Cellulose synthase catalytic subunit [UDP-forming]
MSILTRWLLIPPVNARLIGRYRDYRRHGASAFSATLGCFWMILAWIFIPLEHPRWQRIRAEHKNLYPHINASRPRPLDPV
RYLIQTCWLLIGASRKETPKPRRRAFSGLQNIRGRYHQWMNELPERVSHKTQHLDEKKELGHLSAGARRLILGIIVTFSL
ILALICVTQPFNPLAQFIFLMLLWGVALIVRRMPGRFSALMLIVLSLTVSCRYIWWRYTSTLNWDDPVSLVCGLILLFAE
TYAWIVLVLGYFQVVWPLNRQPVPLPKDMSLWPSVDIFVPTYNEDLNVVKNTIYASLGIDWPKDKLNIWILDDGGREEFR
QFAQNVGVKYIARTTHEHAKAGNINNALKYAKGEFVSIFDCDHVPTRSFLQMTMGWFLKEKQLAMMQTPHHFFSPDPFER
NLGRFRKTPNEGTLFYGLVQDGNDMWDATFFCGSCAVIRRKPLDEIGGIAVETVTEDAHTSLRLHRRGYTSAYMRIPQAA
GLATESLSAHIGQRIRWARGMVQIFRLDNPLTGKGLKFAQRLCYVNAMFHFLSGIPRLIFLTAPLAFLLLHAYIIYAPAL
MIALFVLPHMIHASLTNSKIQGKYRHSFWSEIYETVLAWYIAPPTLVALINPHKGKFNVTAKGGLVEEEYVDWVISRPYI
FLVLLNLVGVAVGIWRYFYGPPTEMLTVVVSMVWVFYNLIVLGGAVAVSVESKQVRRSHRVEMTMPAAIAREDGHLFSCT
VQDFSDGGLGIKINGQAQILEGQKVNLLLKRGQQEYVFPTQVARVMGNEVGLKLMPLTTQQHIDFVQCTFARADTWALWQ
DSYPEDKPLESLLDILKLGFRGYRHLAEFAPSSVKGIFRVLTSLVSWVVSFIPRRPERSETAQPSDQALAQQ

>Escherichia_coli_MG1655_[GlgA] Reference protein: P0A6U8 Glycogen synthase

MQVLHVCSEMFPLLKTGGLADVIGALPAAQIADGVDARVLLPAFPDIRRGVTDAQVVSRRDTFAGHITLLFGHYNGVGIY
LIDAPHLYDRPGSPYHDTNLFAYTDNVLRFALLGWVGAEMASGLDPFWRPDVVHAHDWHAGLAPAYLAARGRPAKSVFTV
HNLAYQGMFYAHHMNDIQLPWSFFNIHGLEFNGQISFLKAGLYYADHITAVSPTYAREITEPQFAYGMEGLLQQRHREGR
LSGVLNGVDEKIWSPETDLLLASRYTRDTLEDKAENKRQLQIAMGLKVDDKVPLFAVVSRLTSQKGLDLVLEALPGLLEQ
GGQLALLGAGDPVLQEGFLAAAAEYPGQVGVQIGYHEAFSHRIMGGADVILVPSRFEPCGLTQLYGLKYGTLPLVRRTGG
LADTVSDCSLENLADGVASGFVFEDSNAWSLLRAIRRAFVLWSRPSLWRFVQRQAMAMDFSWQVAAKSYRELYYRLK

>Escherichia_coli_0157:H7_[BcsA] BLAST: Q8X5L7 Cellulose synthase catalytic subunit [UDP-forming]
MSILTRWLLIPPVNARLIGRYRDYRRHGASAFSATLGCFWMILAWIFIPLEHPRWQRIRAEHKNLYPHINASRPRPLDPV
RYLIQTCWLLIGASRKETPKPRRRAFSGLQNIRGRYHQWMNELPERVSHKTQHLDEKKELGHLSAGARRLILGIIVTFSL
ILALICVTQPFNPLAQFIFLMLLWGGALIVRRMPGRFSALMLIVLSLTVSCRYIWWRYTSTLNWDDPVSLVCGLILLFAE
TYAWIVLVLGYFQVVWPLNRQPVPLPKDMSLWPSVDIFVPTYNEDLNVVKNTIYASLGIDWPKDKLNIWILDDGGREEFR
QFAQNVGVKYIARTTHEHAKAGNINNALKYAKGEFVSIFDCDHVPTRSFLQMTVGWFLKEKQLAMMQTPHHFFSPDPFER
NLGRFRKTPNEGTLFYGLVQDGNDMWDATFFCGSCAVIRRKPLDEIGGIAVETVTEDAHTSLRLHRRGYTSAYMRIPQAA
GLATESLSAHIGQRIRWARGMVQIFRLDNPLTGKGLKFAQRLCYVNAMFHFLSGIPRLIFLTAPLAFLLLHAYIIYAPAL
MIALFVLPHMIHASLTNSKIQGKYRHSFWSEIYETVLAWYIAPPTLVALINPHKGKFNVTAKGGLVEEEYVDWVISRPYI
FLVLLNLVGVAVGIWRYFYGPPTEMLTVVVSMVWVFYNLIVLGGAVAVSVESKQVRRSHRVEMTMPAAIAREDGHLFSCT
VQDFSDGGLGIKINGQAQILEGQKVNLLLKRGQQEYVFPTQVARVMGNEVGLKLMPLTTQQHIDFVQCTFARADTWALWQ
DSYPEDKPLESLLDILKLGFRGYRHLAEFAPSSVKGIFRVLTSLVSWVVSFIPRRPERSETAQPSDQALAQQ

>Escherichia_coli_0157:H7_[GlgA] Reference protein: P0A6V0 Glycogen synthase
MQVLHVCSEMFPLLKTGGLADVIGALPAAQIADGVDARVLLPAFPDIRRGVTDAQVVSRRDTFAGHITLLFGHYNGVGIY
LIDAPHLYDRPGSPYHDTNLFAYTDNVLRFALLGWVGAEMASGLDPFWRPDVVHAHDWHAGLAPAYLAARGRPAKSVFTV
HNLAYQGMFYAHHMNDIQLPWSFFNIHGLEFNGQISFLKAGLYYADHITAVSPTYAREITEPQFAYGMEGLLQQRHREGR
LSGVLNGVDEKIWSPETDLLLASRYTRDTLEDKAENKRQLQIAMGLKVDDKVPLFAVVSRLTSQKGLDLVLEALPGLLEQ
GGQLALLGAGDPVLQEGFLAAAAEYPGQVGVQIGYHEAFSHRIMGGADVILVPSRFEPCGLTQLYGLKYGTLPLVRRTGG
LADTVSDCSLENLADGVASGFVFEDSNAWSLLRAIRRAFVLWSRPSLWRFVQRQAMAMDFSWQVAAKSYRELYYRLK

>Hydrogenophilus_thermoluteolus_[Glucanase] InterPro PF00332-PF13641: A0A2Z6DYI1 Exo-beta-1,3-glucanase
MLRSVTPLLPLLRRTLTALLLTVLVLTAFWQAWAWHVRLGTEPGVAGEINGFAYTPFRRDQSPLEKRYPSPEQIAQDLDL
LALYTKRIRTYGVTDAPAIYPLAHQRKFEVAMGLWVSADQALSEREIAAGLEMAERYDNITRIVVGNEALLRKEMTVAEM
AAYLDRVRDELRRRFPDKNRRPLVSTAEPWHVWLKNPELADHVDFVMIHLLPYHEGIDVARALDYAFGRLEEVQKRFPKL
PVVIGEIGWPSRGEVMPNLLGGDERAEASIENAAHFVRGFLSDPRSLFLDYYIMEAFDQPWKVAVEGWAGAHWGVFDADR
QPKYSLDGLIVRDVRWQEKAKLATFIGAPLLFALAFALASWNLLGRLWLMGLTQLCVIVLLIGVYLPKDYYLSRGDLVGL
IFLILATLMTAAVLLSHGFEFGEVLFKRQWRRRFTPLPPVPPEKAPFVSVHLACYNEPPEMVIATIDSLVAMDYPHFEVI
VVDNNTTDEALWKPVEAHCAQLGDRVKFFHLPKWPGFKAGALNFALRQTDPRAEVVGVVDADYVVERDWLARLIPHFLES
PLVAVVQAPQAHREYEHSFFQRMCNWEFEGFFRIGMHHRNERNALIQHGTMTLIRRPMLEAVGGWSEWCICEDTELGLRL
LERGYEIRYVDHIFGRGLTPSDFAAIKSQRFRWAFGAMQILKAHMPYLLGKKPSQLTFAQRYHFLTGWFAWFGDALHLIF
AMGSLFWTLGMIYAPKTFTLPVATLAAPVLGFMAFKSALGPILYRRTMQTKWIDILGASILSVGLSHTIARGVLAGLTHK
KGTFVRTPKGWRAKGTFAFFGPIREELGMLIAITASGALLVAQRGWLSLEIQLWVAILALQTIPYLAAVLCQIFAYLPDN
TPPETAASTPSPAPHS

>Komagataeibacter_hansenii_[AcsAII] BLAST: Q59167 Cellulose synthase catalytic subunit [UDP-forming])
MIYRAILKRLRLEQLARVPAVSAASPFVMMAVGVFLMLMAGGVTISTTSQAFVTCGTVGLFLLLKGRKGRGVTCFLMMLS
LLVSLRYMVWRLTTTLELHSPLQAALSLLLVAAELYALLTLCLSYFQMSWPLDRKPLPLPADTTDWPVVDVYVPSYNEEL
SLVRSTVLGALAIDWPADKLNVYILDDGRRKSFHAFAMEAGAGYIIRDQNNHAKAGNLNHALRVTEGEYVVIFDCDHIPT
RGFLKKTIGWMMADPKLALLQTPHHFYSPDPFQRNLATGQNVPPEGNMFYGLVQDGNDFWDATFFCGSCAAIRRSAVLGI
GGFATETVTEDAHTALKMQREGWHTAYLRQPLAAGLSTERLMLHIGQRVRWARGMLQIMRLDNPLLGSGLRWQQRLCYLS
AMSHFLFAIPRLVFLASPLAFLFLGQNIIAASPFAILVYAFPHVFHSIGTLSRVEGRWRYSFWSEIYETTLALFLVRVTI
MTLLNPRKGEFNVTDKGGLLQSEYFDLNAVYPNVILAVILALALVRGIGGMMWEYHDRLALQSFALNTLWVAVSLIIVLA
SIAVGRETRQIRHKPRVRATLPITLIDEHGQHYHAHTSDISLGGIAARLSTEHALPTQTRVTMLYHNEKDGIDVRIPAVI
LFSKPGQLHLQWSVDDLDVERQIVEFMFGRNDAWSNWGDFQPDRPVRSFLMVLRSIGGLFRRGQRLFRWQAPQEAPLAES
EHVEEEKLEKKSLVLKPVRRSARHGATASLIVLLGLPAAIAPSLAQAPSRATPVATEQGATPVEPPPVNAPPPPSLPQPP
GTLPTPPQIAPASAGELLPAATAVSLPTGPATQQMRERLSERTGVSPASPFGDTNTGALPADPSAPPIDPADAARVADGE
ITRTSTFRDLGLATGPLTLRGFSPLQGLDVIVPANRVVTRARITLSGALSPSLLPEASAVSVTLNEQYVGTIRVDPEHPR
FGPITFDIDPLYFTGDNKLNFHFAGEYRRDCNDLYNEVLWARISDFSTVTLTTTRIAPDRKLSYLPAPFYDPNLRTPLRV
PVVMPNPDAHGMLKASALVASWFGKLADFRKVSFPVSTTIPASGNAIAIGENLPIDARGTRPTGPTLSEVENPNDRLGTI
LVLTGRNAQEVEVAARVLAFSSDTLGAVGTKVVNDVTLQPRHPYDAPAFVPTDRPVRFGELVAASDLQGGGFAPPVMALP

FHLPPDLYSWRNRPYPIDLWVRTPGGPVVDLETSRLDVHLNNNYLDSFTLKPPSLWAAWSERLVNQHAGAVEHAAALPPW
LLFGQNQLKFSFDARPIDRGVCRRTPDDIHMSVDSDSWLDFRRGYHFARLPNLSYFAEAAFPFSRMADLSETTVVVPHHI
DAGTAGTFMDLMGFFGATTWYPASGVQVADINDLSEHPPQGDILILATAGDAPKFEELLTRAPYELTDGHIRVGQHMGLQ
GIWYLFQDHDHAGLQDGVQANLNAPIAGAGVLLGAQSPYRSDRSVVALMGDTPSRMHDLVMGLRSKEDVPRIQGDLVLRN
GDRLTSYRTAPTFTMGSLPWWMWLDWYLGTRPLTLYVLGLVGAGLVAAAAVRLLRRRAQHRLEEAARVKDTTDASH

>Komagataeibacter_sucrofermentans_ATCC_700178_[BcsA] BLAST: O82859 Cellulose
synthase catalytic subunit [UDP-forming]
MSEVQSPVPTESRLGRISNKILSLRGASYIVGALGLCALIAATTVTLNNNEQLIVAAVCVVIFFVVGRGKSRRTQIFLEV
LSALVSLRYLTWRLTETLDFNTWIQGILGVILLMAELYALYMLFLSYFQTIQPLHRAPLPLPDNVDDWPTVDIFIPTYDE
QLSIVRLTVLGALGIDWPPDKVNVYILDDGVRPEFEQFAKDCGALYIGRVDVDSAHAKAGNLNHAIKRTSGDYILILDCD
HIPTRAFLQIAMGWMVADRKIALMQTPHHFYSPDPFQRNLAVGYRTPPEGNLFYGVIQDGNDFWDATFFCGSCAILRREA
IESIGGFAVETVTEDAHTALRMQRRGWSTAYLRIPVASGLATERLTTHIGQRMRWARGMIQIFRVDNPMLGRGLKLGQRL
CYLSAMTSFFFAIPRVIFLASPLAFLFAGQNIIAAAPLAVAAYALPHMFHSIATAAKVNKGWRYSFWSEVYETTMALFLV
RVTIVTLLFPSKGKFNVTEKGGVLEEEEFDLGATYPNIIFATIMMGGLLIGLFELIVRFNQLDVIARNAYLLNCAWALIS
LIILFAAIAVGRETKQVRYNHRVEAHIPVTVYDAPAEGQPHTYYNATHGMTQDVSMGGVAVHIPLPDVTTGPVKKRIHAV
LDGEEIDIPATMLRCTNGKAVFTWDNNDLDTERDIVRFVFGRADAWLQWNNYEDDRPLRSLWSLLLSIKALFRKKGKIMA
NSRPKKKPLALPVERREPTTIHSGQTQEGKISRAAS

>Komagataeibacter_xylinus_NBRC_13693_[BcsA1] Blast: Q9WX61 Cellulose synthase 1
catalytic subunit [UDP-forming]
MSEVQSSAPAESWFGRFSNKILSLRGASYVVGALGLCALLAATMVTLSLNEQMIVALVCVAVFFIVGRRKSRRTQVFLEV
LSALVSLRYLTWRLTETLDFDTWTQGILGVTLLLAELYALYMLFLSYFQTISPLHRAPLPLPANPDEWPTVDIFIPTYDE
ALSIVRLTVLGALGIDWPPDKVNVYILDDGRREEFARFAEACGARYIARPDNAHAKAGNLNYAIKHTTGDHILILDCDHI
PTRAFLQISMGWMVSDSNIALLQTPHHFYSPDPFQRNLAVGYRTPPEGNLFYGVIQDGNDFWDATFFCGSCAILRRKAIE
EIGGFATETVTEDAHTALRMQRKGWSTAYLRIPLASGLATERLITHIGQRMRWARGMIQIFRVDNPMLGSGLKLGQRLCY
LSAMTSFFFAIPRVIFLASPLAFLFFSQNIIAASPLAVGVYAIPHMFHSIATAAKVNKGWRYSFWSEVYETVMALFLVRV
TIVTMLFPSKGKFNVTEKGGVLEREEFDLTATYPNIIFAIIMALGLLRGLYALIFQHLDIISERAYALNCIWSVISLIIL
MAVISVGRETKQLRQSHRIEAQIPVTVYDYDGNSSHGITEDVSMGGVAIHLPWREVTPDHPVQVVIHAVLDGEEMNLPAT
MIRSAQGKAVFTWSISNIQVEAAVVRFVFGRADAWLQWNNYEDDRPLRSLWSLILSIKALFRRKGQMIAHSRPKKKPIAL
PVERREPTTSQGGQKQEGKISRAAS

>Limnobacter_strain_130_[Glucosyltransferase] InterPro PF00332-PF13641:
A0A653I611 Beta-(1-3)-glucosyl transferase
MPSVFALFAFVLLVVGGYGYINQPEQVPPWNQVIPGFAFSPYQAGQSPIDNIEPKVEDINRDLSLLAGKTLAIRTYTVAG
IFGEIPALAKTHNINVALGAWLSPDLQANEKELDRLLSVAHSPPYNVVRLIAGNEALLREDLNLTQMIAYLDKVRSATDI
PVSTAEPWHVWLKNPELAEHVDYLAVHLLPFWEGIALDNAVDFSINTYQRLQEAFPGKPIVVTEVGWPSNGRSIKQADAS
QANQAKFLRRFIPRAEQEHMVFYVMEAFDQPWKSDIEGSVGGHWGVFDSFREPKFEPDRPIIAIPQWKLLAAASIVVALM
MTFLLLIDSRTLRDSGRTFLIFNAFVISSLIIYVIYDFTLQYHTWVSVAISVFLLLGVVGVFVIVMSEAHEWAEAMWYTS
RRRLLTLPTRTDSHSSVVTKYRPFVSVHVPAYEEPPEMLIDTLNALTRLDYPNFEVIVVDNNTKDDDTWRPVQAHCQLLG
ERFRFFHVKPLSGYKAGALNYALERTCPHAEVVAVIDADYKVQPHWLSDLAPQFEKPEIAIVQAPQDYRDGADSAFKSLC
YAEYKGFFHLGMVTRNERNAIIQHGTMTMVRRSVLEKVGCWGVSTITEDTELGLRIFEQGHEAVYIDQSYGHGLIPDTFT
DYKKQRHRWAYGAMQILREHAGQLLGFRRSSLTAGQRYHFVSGWLPWVADGLNLVFTLLAIVWSGLMLFDPLTFNAPPLL
ISVVPIMFFLFKITKLFTLYLVQVKASFRTAFAATLAGLSLSYAIGRATLSGLFVGRKIPFIRTPKMANRMAVLYAMNAA
RDESVLAFVLLCAVGVIYFQLGFDSKENLAWCLVLVSQSLPFVAAFVVSLLSAAPTRAAAHNEVGVKQS

>Magnetospirillum_fulvum_[Glucanase] InterPro PF00332-PF13641: A0A1H6GNJ8 Exo-
beta-1,3-glucanase, GH17 family
MRLSGIVVLLLIVLGNLGFWAAINRPQTGLPWSGTIKSVSFSPSRADDDPTLVRKLPYLDETRLATREEMDEDLALLAGK
VEQVRTYSTLEGLDQVPELAAHHGLKALPGAWLDERLARNEVELNNIIRIARDNPNVERVIIGNENLTLHRLSVDQMIRY
LRRVRAALPDRVKISTAEAWSIWLDYPELAREVDFITIHTLPFWERVDISGALDFTKRMVREVKNAYPDKPLFIGEVGWP
SAGRSYGDSEPSLVNQALFLRHFLNWAHQEKLDYNIVEAFDQPWKVNLDNNASEKHWGIWTVDRQPKFNWIGPVIEFEEW
PFQAAAATFIAFLPVAWFLFKWRNLRLPGKIFFGVLVQFAATLVIWTMSTPVLKDVAPGTGLMLGLLLPAQLLLLFVVLI
AGIEVTELTWASRFKRRFTALPSAECKRFPKVSLHLPCYNEPPDMVKLTIDSLMALDYPNFEILVLDNNTKDPNVWGPVR
DYCDTLGEKVKFFHLAPWPGAKAGALNFALTVTDPEAEIIGVVDSDYIVSPNWLKGLVPYFENPKVGHVQAPQDHREWEN
DLFKEMINWEYAGFFDIGMVYRNEANAIIQHGTMTLIRKEALEKAGRWGEWCIVEDAELGLRFLKDGYESVYVQDRIGHG
LVPDSFMAYKKQRFRWAYGAVQILKAHWRSLMPFTKSGLTAGQKYHFVSGWLPWFADAFYLLFTIASLIWSLGMVLAPRY
FPTPLAVFTLPTVGVFVAKIVHHFFLYTTRVKCTLRQRSLAAIAGMGLTYSIARAMWQGMFTKSTPFMRTPKMANKAAFT
QGFLMASEESALMLAQWIAAIVVLLPKNNFYDPDVRLWSLVLVVQAMPFLAALITSLISVMPSKVSTDAPPAPTADPAKV
QPAA

>Magnetospirillum_gryphiswaldense_DSM_6361_Glycosyltransferase] InterPro PF00332-PF13641: V6F4I5 Glycosyl transferase, family 2
MRFSGFLVLLVIMLANLGFWSALNQPKTGLPWSGTIQSVSFSPSRADDDPTIARLMPHMEDTLLATREEMDEDLAMLSGT
VQMVRTYSTLEGLDQVPELAGKHGLKALPGAWLDERLGRNEREIANIIRIARDNPNVERIIVGNENLTLHRLTPEQMIRY
IRRVRAEVPARVKISTAEAWSIWLEHPELAQEVDYITIHTLPFWERVNIDEALPFTQRMVHEVKAAYPDKPLFIGEVGWP
SAGRSYGRAEPSLVNQALFLRRFLNWANEEGLDYNVVEAFDQPWKVNLDGTASEKHWGIFSVERTPKFDWIGPVIEFQEW
PFQAAAATLIAFLPVVWFLMRWKDLRLAGKVFFGMLVQFAATLVIWTMSTPVVRDLAPATELMIFVLLPAQLLLLVVVLI
NGIEVTELTWSGRLKRGFKPHPRDVIKRYPKVSLHLPCYNEPPEMVKLTLDSLLALDYPNFEVIVLDNNTKKEEVWKPVE
AYCAQFPDKVKFYHLAPWPGAKAGALNFGLSVTDPEAEIIGVVDSDYQVRRDWLSSLVPYFEDPKVGHVQAPQDHRDWER
DLFKEMINWEYAGFFDIGMVFRNEANAIIQHGTMTLVRKKAMDDAGKWAEWCIVEDAELGLRMMKLGYESVYIQERMGHG
LVPDSFMAYKKQRFRWAYGAVQILKAHWKSLIPFKETGLTAGQKYHFVSGWLPWFADGFYLMFCLTSLFWTAGMVLAPRY
FDTPLAFFILPTVGVFIAKIIHHIFLYTTRVKCGWKQRLLSAVAGMGLTYAIAWAMWQGIFTKHTPFMRTPKMAEKVGLK
DAVKMTAEETTLMLAHWAAAVAVLIPGPNAQDPEIRLWAVVLVVQSMPFLAALVSSVISTLPSRPAPVEKAPDAPTPAAA
E

>Magnetospirillum_magneticum_AMB-1_[Cellulose_synthase] InterPro PF00332-PF13641: Q2W2R8 Cellulose synthase catalytic subunit
MRLSGFVVLLLIVLGNLGFWAVMNRPQNAALPWSGTLNSVSFSAGRADDDPTIVRKLPYMDEWLLPTRAEMDEDLAMLAG
KVHQVRTYSTLEGLDQVPELAAKYGLKALPGAWLDERLGRNEVEIANIIRIARDNPNVDRVIIGNENLTLHRLSPEQMIR
YLRRVRAALPDRVKISTAEAWAIWLDYPELTREVDFITIHTLPFWEPGGVHIDNALDFTKRMVRDVKAAYPDKPIFIGEV
GWPSAGRNYGVSEPSLVNQAMFLRNFVNWAHEEKLDYNIVEAFDQPWKVNLDNTASEKHWGIYTVERQPKFSWIGPVLEF
EEWPTQAITATLIALLPVVWFLGKWKTLRLPGKIFFALLVQFASTLLIWTMSTPVIRDVSPGTGLMLGLMLPAQLLLLIV
VLIAGIEVTELTWASKFKRRFTALPPDQCKRFPKVSIHLPCYNEPPAMVKLTLDSLMALDYPNFEIIVLDNNTKKEEVWR
PVEEYCKTLGDKVKFFHLAPWPGAKAGALNFGLTVTDPEAEIIGVVDSDYMVDKNWLKGLVPYFENPKVGHVQAPQDHRE
WEHDLFKEMINWEYAGFFDIGMVFRNEADAIIQHGTMTLVRKKTLEDAGRWGEWCIVEDAELGLRMMKAGYQSVYVQDRL
GHGLVPDSFMAYKKQRFRWAYGAVQILKAHWRSLIPFKQTGLTTGQKYHFVAGWLPWFADAFYLLFCVAALAWSLGMIVA
PRYFSTPLPFFTLPTVGVFVAKIFHHFFLYTTRVNCGLKRRSLAAIAGMGLTYSIAWAMWQGIFTKSTPFMRTPKMANKA
AFTQGFLMASEEATLALLHYVAAIAVLIPRNNFTDPDVRIWSLTLVVQAMPFLAALVASLISVMPSDGPAQPEHSHSNAK
PEAAE

>Magnetospirillum_strain_ME-1_[Cellulose_synthase] InterPro PF00332-PF13641: A0A1W6CTZ4 Cellulose synthase
MRLSGFVVLLLIVLGNLGFWAMMNRPQSGLPWSGTLNSVSFSPGRADDDPTIVRKLPYMDEWLLPTRAEMDEDLAMLAGK
VHQVRTYSTLEGLDQVPELAAKYGLKALPGAWLDERLGRNEVELANIVRIARDNPNVDRVIIGNENLTLHRLSPDQMIRY
LRRARALLPDRVKISTAEAWAIWLDYPELAREVDFITIHTLPFWEPGGVHIENALDFTKRMVRDVKAQYPDKPIFIGEVG
WPSAGRNYGVSEPSLINQAMFLRNFVNWAHEEKLDYNIVEAFDQPWKVNLDNTASEKHWGIYTVERKPKFSWINPVLEFE
EWPVQAITATLIALLPVVWFLGKWKTLRLPGKIFFALLVQFASTLLIWTMSTPVIRDVSPGTGLMLGLMLPAQLLLLIVV
LIAGIEVTELTWASKFKRRFTALPPDHIKRFPKVSIHLPCYNEPPAMVKLTIDSLMALDYPNFEIIVLDNNTKKEEVWRP
VEEYCKSLGEKVKFYHLAPWPGAKAGALNFGLTVTDPEAEIIGVVDSDYIVDKNWLRGLVPYFEDPKVGHVQAPQDHREW
EHDLFKEMINWEYAGFFDIGMVFRNEADAIIQHGTMTLVRKKTLEDAGRWGEWCIVEDAELGLRMMKAGYSSVYVQDRLG
HGLVPDSFMAYKKQRFRWAYGAVQILKAHWRSLIPFKDTGLTTGQKYHFVAGWLPWFADAFYLLFCVASLAWSLGMILSP
RYFSTPLPFFTLPTVGVFVAKIFHHFFLYSNRVDCGFKRRGLAAIAGMGLTYSIAWAMWQGIFTKSTPFMRTPKMANKAA
FTQGFLMASSEAILALLHYVAAIAVLIPRNNFTDPDVRIWSLTLVVQAMPFLAALVASLISVMPSNGPEHPAHSLSDAKP
EAAS

>Magnetospirillum_strain_XM-1_[Cellulose_synthase] InterPro PF00332-PF13641: A0A0U5MHP5 Putative Cellulose synthase catalytic subunit
MRLSGFVVLLLIVLGNLGFWAMMNRPQSGLPWSGTLNSVSFSPGRADDDPTIVRKLPYMDEWLLPTRAEMDEDLAMLAGK
VHQVRTYSTLEGLDQVPELAAKYGLKALPGAWLDERLGRNEVELANIVRIARDNPNVDRVIIGNENLTLHRLTPDQMIRY
LRRARALLPDRVKISTAEAWAIWLDYPELAREVDFITIHTLPFWEPGGVRIENALDFTKRMVRDVKAQYPDKPIFIGEVG
WPSAGRNYGVSEPSLINQAMFLRNFVNWAHEEKLDYNIVEAFDQPWKVNLDNTASEKHWGIYTVDRKPKFSWINPVLEFE
EWPVQAITATLIALLPVVWFLGKWKSLRLPGKIFFALLVQFASTLLIWTMSTPVIRDVSPGTGLMLGLMLPAQLLLLIVV
LIAGIEVTELTWASKFKRRFTALPPDHIKRFPKVSIHLPCYNEPPAMVKLTIDSLMALDYPNFEIIVLDNNTKKEEVWRP
VEEYCKSLGEKVKFYHLAPWPGAKAGALNFGLTVTDPEAEIIGVVDSDYIVDKNWLRGLVPYFEDPKVGHVQAPQDHREW
EHDLFKEMINWEYAGFFDIGMVFRNEADAIIQHGTMTLVRKKTLEDAGRWGEWCIVEDAELGLRMMKAGYQSVYVQDRLG
HGLVPDSFMAYKKQRFRWAYGAVQILKAHWRSLIPFKKTGLTTGQKYHFVAGWLPWFADAFYLLFAAASLAWSLGMIVAP
RYFSTPLPFFTLPTVGVFVAKIFHHFFLYSTRVNCGFKRRGLAAIAGMGLTYSIAWAMWQGIFTKSTPFMRTPKMANKAA
FTQGFLMASSEAILALLHYVAAVAVLIPRNNFTDPDVRIWSLTLVVQAMPFLAALVASLISVMPSDGPEQPAHSHSDAKP
EAAQ

>Methylobacter_tundripaludum_ATCC_BAA-1195_[Glycosyltransferase] InterPro PF00332-PF13641: G3IW31 Glycosyl transferase family 2
MKTKIVTVIALALIVFINFSIWSYVNNPLQLQPWTKTTMGVTFDPMRKQDTQKSDTFPSEADIDHDLGLLENKVHAVRTY
SVLKGLDKIPELAAKHNLNTTVGAWIDSNLEKNRQEIETLINVSRQNNPNIVRVMVGNEVLLRGDMTAEQLIEYIREVKR
NTWRPVSTSETWDIWIKHPELVAEVDFIAVHILPYWEGIAAEDAVDYVFDRYHDVQKAYPNKPIVITEVGWPSDGQPFKH
ATASVSNQAKFLREFLNRADAEKVTYYIVEAFDQPWKMSLEGSAGAYWGIFNADRQPKYPMDSDVIAMPNWEHWATGAAV
FSVVLMGLFLFTRSSMKLPGLLFFGLIANLAASTIFWSVSIGAQQYQTNLSMVFWGILILMQVMAAVILLIETLEIAEVI
WHRKTARTFQPLKPSPEFKYPKVSLHLPIHNEPPDMVRMTLEALDRVDYPNLEVLVMDNNTKDPAVWEPVKVDCERLGPK
FRFFHLDNWPGFKAGAINHALEQTAPDAEIIAVIDSDYILSPDWLNAMVPYFDNENVGFIQSPQDYRDRDQSAFKSFCYW
EYAGFFNIGMVQRNEYNAIIQHGTMTMIRKSALLEVGKWSEWCICEDSELGLRLYEAGYDSVYVKDSFGKGVMPDTMSGY
MTQRYRWVYGAMQIIKAHWRSFLPSKNPVLTPAQKYYFIAGWLPWFSDALALLFTITSLILTAVILYDPIHSELPANAFL
LPTVGIFSFKIIRGLWLYQARVPCSIWQSLGASLSGLALTHTVAKGTVQGLFTSGKPFMRTPKFEQHSALFVGLVTIRQE
LLLLILLSTAIGMMASLEHFDNFSGKLWIAILSVQAVPYVAALLTLLISIAPDYKSYNPELVVEETAANSKSKKK

>Methylomicrobium_album_[Glucanase] InterPro PF00332-PF13641: H8GHK0 Exo-beta-1,3-glucanase
MKRTLTILLTLTVLVLVNFSIWSYINNPLQLRSWTKTTMGVTFDPRRKEYTQKNGQFPTEAEIDADLTVLENKVHAVRTY
SVLKGLHKVPELAAKHGLNTTIGAWIDGDLEKNRQEIETLLQVSNQNNNKIVRVMVGNEVLFRNDIPVQQLIDYIREVKK
RTWRPVSTSETFDIWLKHPELVAEVDFIGAHILPYWNGIAVEDAVDFVFEKYEELKKAYPDKPIVITEVGWPSDGQPSRH
ATASRTNQAFFLREFLNRAAEQKIPYYIVEAFDQPWKMEIEGSAGAYWGIFNADRQPKYPMDGAVLALPSWRDWATGAAA
ASAVLMGLFLFTRRTMALPGIVFFGVITNLAASFIFWTVSIGAQQYQTELTIVFWVLLIVMQLLALTILLIETMEIVEVV
WHRRTTRTFQPLAPSPDFRYPKVSIHLPIHNEPPMMVRKTLEALAKLDYPHYEVMVMDNNTKDPAVWEPVRDDCERLGEK
FRFFHLENWPGFKAGAINHALEQTAPDAEIIAVIDSDYILSPDWLKRMVPYFDNENIGFVQSPQDYRDGHVNLFKRFCYW
EYAGFFNIGMVQRNEYNAIIQHGTMTMIRKSALFEVGKWSEWCICEDSELGLRLYEAGYDSVYVKDSFGRGLMPDTMSGY
MTQRFRWVYGAMQIIKAHWKSFKPTKKSPLTSAQRYYFVAGWLPWFSDALALVFTVASLVLTGVIFLDPIHTELPVNAFL
LPTIGLFTFKILRSLWLYQAKVPCRLWESVGASLSGLALTHTVARGVWQGLFVPGKPFMRTPKYEKQGPLFAGLMVIRQE
LFLLILLMAGVFGVNSLEHFDNLSGKLWMGVLSVQAVPYLATTITLLMSILPYYRKGSALTIDEIDDEIVASSGKSE

>Methylomicrobium_buryatense_[Glycosyltransferase] InterPro PF00332-PF13641: A0A4P9UIM6 Glycosyltransferase
MKTKILTLLILPLLVWVNLSIWNYLNYPLKMQSWDKVMKGITYSPMRRDFDPGNSSPPTQVQIEDDLKLLAGKVHAVRTY
SALEGLEFVPELAEKYDLNVTMGAWIDADLDKNRREIDSLIELSNQNSPTIVRLLVGNEVLLRKDIAPDQLIAYIREVKA
RTWRPVSTSETWDMWLAHPELAEEVDFIALHILPYWEGLSIDAAVDYVFYRFNAMREAFPNKPIIITEVGWPSDGQPFKN
ATASLANQAQFLRQFLNRATEQKITYYIIEAFDQPWKVELEGSAGAYWGIFNADRELKFPMKGDVTPMPDWQAWATGAAV
LSIFLMALFLFSRHRRLKLPGKIFFGIVANLAASVILWSAAVAAQQYQTGVSLVFWTLLLLMQAMAVVILLTESMEIAEV
LWHRKGKRTFKPLQPPAGFTFPKVSVHLPIHNEPPEMVRETLEALARVDYPNLEVLVLDNNTKDPAVWEPVQKDCERLGG
VFKFFHLENWPGFKAGAINFGLEQTASDADIIAVIDSDYIISPDWLKSMVPYFEDEKVGFVQSPQDYRDRGLSTFKSMCY
WEYAGFFNIGMVQRNEYNAIIQHGTMTMIRKSALLEVGRWGEWCICEDSELGLRLYEAGYDSVYCKDSFGQGLMPDTFSG
YMTQRFRWVYGAMQIIKHHWRQFLPGKQSTLTTAQRYYFIAGWLPWFSDALALLLTIASLIMTTLLVADPLRSELPVNAL
LLPTIGLFCFKIFRTLWLYKARVNCSTLQSLGAALSGLSLTHTVAKGTLQGLFTSGKPFMRTPKLEKQGPFIAGLATIWQ
ELLLLTLLGLAIYSMSSIDHFDNLSGRLWVAVLSVQTVPYIATFLTLLFSIAPNYYPGSETQAIKGPQDKELL

>Methylomonas_denitrificans_[Cellulose_synthase] InterPro PF00332-PF13641: A0A126T3H2 Cellulose synthase
MKAKIATFFTLVLLVVINLGIWSYVNNPLKLPSWSDTMMGVTFNPKGRDFNPADGVFPTREQIQSDLELLSGKAHSIRTY
TALEGMEVVPELTAKNAINLAMGCWVDLVDDDSADDDPQKKLENTQKRLDKNQREVESLINLTNQYPKTIVRTLVGNESL
LRYRNKISGPIAQKVRQEFSGSMSEQDLQLKIEADVKAEVAGKANELIEYIREVKKRTWKPVSTAETWDIWVANPALAAE
VDYIAVHILPYWEGVPVDLPQGAEGDNAVEYVFKRYYELQALYPNKTIVITEVGWPSDGPPQKAASASLANQAKFLREFL
NRATAENVIYYVVEAFDQPWKIKLEGTAGAYWGLFNADRQPKFPMEGDVLANPTWRNWASGAAVLSIILMAAFLFTRKSL
KLPGKFFFGIVANLAASVLFWSASIAAAQYQTSFSVVFWAILLMMQAMAILVLLTESLEISEVIWHRKGRRTFTPLTPSA
DFRYPKVSIHLPIHNEPPEMVRKTLNALAKVDYPNFEVLVMDNNTKDPTVWQPVRDDCDRLGARFRFFHLDNWPGYKAGA
INYALTNTAEDAEIIAVIDSDYILNPDWLKAMVPYFDQENVGFVQSPQDYRDANQGAFKDICYWEYAGFFNIGMVQRNEF
NAIIQHGTMTMVRKSAFDKVGPWGEWCICEDSELGLRLYEAGYDSVYCKESFGRGLMPDTFSGYMTQRFRWVYGAMQIIK
KHWRHFLPNKKSSLTSAQRYYFVAGWLPWFSDALALLFTGTSLVLTALIVSDPIHSELPVNAFLLPTIGLFAFKILRGLW
LYKARVACSMIHALGAALAGLSLTHTVARGTLQGLFTSGKPFMRTPKYEQQGPLVAGLLIIWQELLLLALLVAGILAMRS
IEHFDNLSGRLWMAVLAVQSVPYVATLLTILISVAPNYFPGKKLSADELDADE

>Methylomonas_koyamae_[Cellulose-synthase] InterPro PF00332-PF13641: A0A177MWM5 Cellulose synthase
MKAKIATFFTLVLLVLINLGIWSYINNPLKLPSWSDTMMGVTFNPKQRDFNPQDSIFPTREQIQADVELLSGKAHSIRTY
TALEGMEVVPELTAKSALNLAMGCWVDLVEDEESEENRQKRLDKNQREVESLINLANQYPKTIIRTLVGNESLLRVRNKV

SEPVAKRVRDEFAGQMSEQELQDKIKAEINLQVTNKANELIEYIREVKKRTWKPVSTAETWDIWVDHPELAAEVDYIAVH
ILPYWEGIPVELPQGAAGDTAVEYVFKRYYELQKLYPDKTIVITEVGWPSDGPPQKAATASLANQAKFLREFLNRATAEN
VIYYVVEAFDQPWKIKLEGTAGAYWGLFNADRQPKFPMEGDVLANPTWRNWASGAAILSIILMAAFLFTRKSLKLPGKFF
FGIVANLAASVLFWSASIAAAQYQTGFSVVFWAILLMMQAMAILVLLTESLEIAEVIWHRKGRRTFQPLTPSADFRYPKV
SIHLPIHNEPPEMVRKTLNALAKVDYPNYEVLVMDNNTKDPAVWQPVRDDCDRLGEKFRFFHLDNWPGYKAGAINYALEN
TAEDAEIIAVIDSDYILSPDWLKAMVPYFDQENVGFVQSPQDYRDAHQGSFKNMCYWEYAGFFNIGMVQRNEYNAIIQHG
TMTMVRKSAFEKVGPWGEWCICEDSELGLRLYEAGYDSVYCKESFGRGLMPDTFSGYMTQRFRWVYGAMQIIKKHWRHFL
PNKRSSLTSAQRYYFVAGWLPWFSDALALLFTGTSLILTALIVADPKHSELPVNAFLLPTIGLFAFKIVRGLWLYKARVA
CSMLQALGAALAGLSLTHTVARGTLQGLFTSGKPFMRTPKYEQQGPLVAGLLIIWQELLLLTLLVAGIVAMRSIEYFDNL
SGRLWVAVLAVQSVPYIATFVTILISVAPNYFPGKKLSADELDAE

>Methylomonas_methanica_MC09_[Glycosyltransferase] InterPro PF00332-PF13641:
G0A4Z3 Glycosyl transferase family 2
MKAKIATLFTLIVLVVVNFAIWTYINNPLQLPSWSSTMMGVTYNGTRRDFDPAKRNYPTPEQVQEDLTLLADKVHAIRTY
TSLEGMEAVPEAAAKNNINLAMGAWVNLFENTIVVPDDISEEEREKILDKNRSIEAQNQKIEERNRRDIESLIKLSNDYP
KTIVRTMVGNEALLRARNNLAGPIAKRIREEYRGQGLSEREINDKIDLAVTQAVAEKANELISYIKEVKKRTWKPVSTSE
TWDIWAKNPALVDAVDYIGVHILPYWEGIPVDLPEGAKGEAAVDYVFKRYYELQQLYPNKPIVITETGWPSDGPPQKSAT
ASLINEAKFLRAFLNRATAENVIYYIIEAFDQPWKIKLEGTAGAYWGLFNVDRQPKFPMEGEVVGNPTWRNFATGAAVLS
FILMAAFLFTRKRLKLAGKFLFGIMANLAASVLFWSASIAASQYQTGFSVVFWSILLMMQAMAILVLLTESLELAEVLWH
RKGRRTFSPLAPHADFKYPKVSIHLPIHNEPPEMVRKTLNALAKVDYPNYEVLVMDNNTKDPAVWQPVQADCDRLGDKFR
FFHLDNWPGFKAGAINYALENTAEDAEIIAVIDSDYILSPDWLKSMVPYFDQENVGFVQSPQDYRDAHQSTFKDMCYWEY
AGFFNIGMVQRNEYNAIIQHGTMTMVRKSAFDKVGAWGEWCICEDSELGLRLYEAGYDSVYCKESFGRGLMPDTFSGYMT
QRFRWVYGAMQIIKKHWRHFLPNKKSSLTSAQRYYFVAGWLPWFSDALALLFTGASLILTTMLVLNPLHSELPINAFLLP
TIGLFVFKVGRGLWLYKARVACSLLQALGAALAGLSLTHTVARGTLQGLFTSGKPFMRTPKYEAQGPLIAGLLVIWQEIM
LLSLLIAGIVAMRMEPQFDNTSGRLWIAVLAVQAVPYLATFLTILISVAPNYMPGSKVAAEELDVDS

>Methylomonas_strain_DH-1_[Cellulose-synthase] InterPro PF00332-PF13641:
A0A172UCV7 Cellulose synthase
MKAKIATFFTLVLLVLINLGIWSYINNPLKLPSWSDTMMGVTFNPKQRDFNPQDSIFPTREQIQADVELLSGKAHSIRTY
TALEGMEVVPELTAKSALNLAMGCWVDLVEDEESEENRQKRLDKNQREVESLINLANQYPKTIIRTLVGNESLLRVRNKS
EPVAKRVRDEFAGQMSEQELQDKIKAEINLEVTNKANELIEYIREVKKRTWKPVSTAETWDIWVDHPELAAEVDYIAVHI
LPYWEGIPVELPQGAAGDTAVEYVFKRYYELQKLYPDKTIVITEVGWPSDGPPQKAATASLANQAKFLREFLNRATAENV
IYYVVEAFDQPWKIKLEGTAGAYWGLFNADRQPKFPMEGDVLANPTWRNWASGAAILSIILMAAFLFTRKSLKLPGKFFF
GIVANLAASVLFWSASIAAAQYQTGFSVVFWAILLMMQAMAILVLLTESLEIAEVIWHRKGRRTFQPLTPSADFRYPKVS
IHLPIHNEPPEMVRKTLNALAKVDYPNYEVLVMDNNTKDPAVWQPVRDDCDRLGEKFRFFHLDNWPGYKAGAINYALENT
AEDAEIIAVIDSDYILSPDWLKAMVPYFDQENVGFVQSPQDYRDAHQGSFKNMCYWEYAGFFNIGMVQRNEYNAIIQHGT
MTMVRKSAFEKVGPWGEWCICEDSELGLRLYEAGYDSVYCKESFGRGLMPDTFSGYMTQRFRWVYGAMQIIKKHWRHFLP
NKRSSLTSAQRYYFVAGWLPWFSDALALLFTGTSLILTALIVADPKHSELPVNAFLLPTIGLFAFKIVRGLWLYKARVAC
SMLQALGAALAGLSLTHTVARGTLQGLFTSGKPFMRTPKYEQQGPLVAGLLIIWQELLLLTLLVAGIVAMRSIEYFDNLS
GRLWVAVLAVQSVPYIATFVTILISVAPNYFPGKKLSADELDAE

>Methylovulum_psychrotolerans_[Cellulose-synthase] InterPro PF00332-PF13641:
A0A1Z4BUA8 Cellulose synthase
MKTKIATVLALTLLVMINFSIWSFVNNPLQLQPWTKTTMGLTYDPTRKTDDPRDNIFVSEEDMDKDLTLLENKVHAIRTY
SMLRGQNKIPDIAAKHNLNVTMGAWIDGNLEKNRQELEALINVSRQDNPKIIRVMVGNEVLLRNEIPEDALIEYIREVKK
RNWRPVSTSETWAEWLKHPKVAAEVDFIAVHILPYWEGIAAEDAVDYIFDRYHQVQQAYPDKPVIITELGWPSDGQPAKH
ATASVSNQAKILREFLNRADQEKITYYIVEAFDQPWKQSIEGSAGAYWGIFNADRQAKYPMDGDVLTLPSWSHWATGAAV
LSVVLMALFLFTRRSLKLPGMVFFGLITNLAVSTIFWSLSIGAQQYQTPLTIIFWVVLILMQVLAAIILLIESLEIAEVI
WHRKTARTFQPLTPSPDFKYPKVSLHLPIHNEPPLMVRKTLEALAKVDYPNMEVLVMDNNTKDAEIWEPVRDDCERLGPM
FRFFHLENWPGFKAGAINHALEQTAADAEIIAVIDSDYILSPDWLNCMVPYFDNENVGFVQSPQDYRDRHQSTFKDICYW
EYAGFFNIGMVQRNEYNAIIQHGTMTMVRKSALLEVGKWSEWCICEDSELGLRLYEAGYDSVYVKDSFGRGLMPDTFSGY
MTQRFRWVYGAMQIIKAHWRSFLPTQKSPLTSAQRYYFVAGWLPWFSDALALVFTITSLLLTAIIVYDPIHSELPINAFL
LPTIGLFTFKVLRGLWLYQARVPCSFWQSLGAALSGLSLTHTVARGTIQGMFTSGKPFMRTPKYEKQGPLFAGLMIIRQE
VILMLLLAAAMIAMASLEHFDNLSGRLWISILAVQAVPYIATVATLLISIAPHYGKNAKLAEELDEV

>Mizugakiibacter_sediminis_[Benzoate_transporter] InterPro PF00332-PF13641:
A0A0K8QQI8 Benzoate transporter
MNPPSPPSRRTLVAAFLMATLVAALNFGLWWWANRPTAVVDWDGPVGGFAFSAFQRYQSPFTDSYPNAAELASDIALLSK
YTHRLRTYSTLDNPEIPRLARDAGLQLLAGAWLDRRWEHNERELAALIAASRRYPNITRVMVGNEVLLRNDMTPAQLMAY
LDRARAAIRQPVSTAEPWHIWMKYPELADHVDYITVHLLPYWEGVPRKDAVGQALMRYQQIKDLFPGKHVVIGEIGWPSN
GDRYQYAQPSVANEGIFLRQWMNVARERGIDYYLMEAFDQPWKENLGEGRVGAYWGMFGADRKPKFPFSGPVIEDVDWPW

KALASAALAFLPMLWFARRYGRFKPAGRLFFLVLIQLAAGLIVWSVTVPFQFYLGVIDWAMLVILFPAQLAILAVLLING
FEFTEVLWRRKWLRHAGPLEPEPGAPQPFVSIHLACYNEPPEMVILTLDSLAALDYENYEVLVIDNNTRDEAIWKPVEAH
CAKLGPKFRFFHLAPWPGFKAGALNYGLKVTDPRADVVAVVDADYVVRHDWLRALTGYFHDPKVAVVQCPQAHRDFERNP
FRRMTAWEYDGFFRIGMHHRNERNAIIQHGTMTMVRRSALEGTGGWSEWTICEDAELGLRLMHSGYELVYVDELMGKGLT
PADFKAYKSQRTRWAFGAMQILKGRWGWLVRKGPLTAGQRFHFLTGWFSWFADALHLVFTLMALYWTAGMIGLPQVFSLP
MQLFLVPVIGFFFAKAAFGIVLYRARVPCGWRDTLMASLASMGLSHAIARGILQGLVKKKGEFVVTAKSRRIGGGAFGAF
APVHEELLMATALALGVIGMLHAYGGTYLEGKLWVGILAAQSIPYLSALAGAWIAHRAGSEIG

>Neosartorya_fischeri_ATCC_1020_[BtgE] BLAST: A1DBG6 Probable beta-glucosidase
MRGAILATAAALAGTAMADVAHMRRHGHDSFHQRRAAVAEADATCGCTTEVVTVWGPPTLIPVATPTPSTVTSEAVTTLH
STSTSTVTIVASASTPATSSSPATPKVPLPTPAITNFPSTGVYTIPATTVTVFDTTTVCGATTTELPAGTHTYGGVTTVV
ETATTVVCPYATVEPSGTTVTSVIKTTTYVCPTPGTYTIAPTTTTVPTSTVVVYPTPAVITPGTYTQPEQTVTVTRTDYT
YVCPFTGQNEPTSAPAAPSTTAVPATTTAAVPSTSSAAPSSSSTAPASTGAVGGQMGMTYTPYTKGGDCKDKSSVLSEVA
NLKSKGFTHVRVYSTDCNSLEYIGEAARTSGLQMIIGVFISSTGVSGAQDQVTAISKWAQWDLVSLIVVGNEAIQNGYCD
ASTLAGFISSAKSAFQSAGYTGKVTTTEPINVWQAYGSTLCGVCDIIGANIHPFFNADVSADQAGKFVAQEIKVLEGICP
GKDVLNLETGWPHAGNANGKAVPGASEQAIAIKSIAQEVGSKSVFFSYFDDLWKEPGQFDVERYWGCIDTFN

>Neosartorya_fumigate_ATCC_MYA-4609_[BtgC] BLAST: Q4WUK5 Probable glucan endo-1,3-beta-glucosidase
MSGPNRTYSFGEGDDSLAHPSSRTHAMHSQYDDVSPISDGARMNPMNGQGMDHGLASVLEDGRQGWGRSPEPSPSLLTGS
SATPGMDNLGPGAVGGGISGIALSVANSHDRLSGVEALMGTDGQEANIPAERGLSTTGSDNPYVPEPPEHRYSYGSNIAL
GAAAAPAGQLTPGQSVSHLSSTNPSQRNLYDIPYQDVGGLNAGPYQRHSAYSSNDLPVDINPDEIVDDGDDGFVPAPNSG
SGARKSQAIPAAAGGAAAGGVLGNLGGLFGGKSAADTSYGPVPGAGLEAGEKGRWVKPKPGGGNKKRGWIVGAILAFIII
GAIVGGAVGGTIGHRGNEEPSSASSASSSSTQTATEDTSVNGDLDKNSAEIKALMNNKNLHKVFPGIDYTPWGVQYPLCL
KYPPSQNNVTRDMAVLTQLTNNVRLYGTDCNQTEMVLHAIDKLEIKDMKIWLGVWIDSNETTSRRQIDQLYKIIDDAKDI
SIFNGAIVGNEALYRAGSDKTSAQTTLINYMQEVKDHFKKKNIDLPVATSDLGDNWDATLVQAADVVMANVHPFFGGIPV
DQAAAWTWRFWQDHNVALTKGTNKKQIISEVGWPSGGGNDCGQGANCPNDTAGAVAGVDELNKFMEDWVCQALDNGTDYF
WFEAFDEPWKIVYNTGKENWEDKWGLMDSARNLKPGLKIPDCGGKTAT

>Oryza_sativa_[Mannosyltransferase_OsCslA11] BLAST: Q6YWK8 Probable glucomannan 4-beta-mannosyltransferase 11, OsCslA11
MSSSGGGGVAEEVARLWGELPVRVVWAAVAAQWAAAAAAARAAVVVPAVRALVAVSLAMTVMILAEKLFVAAVCLAVRAF
RLRPDRRYKWLPIGAAAAAASSEDDEESGLVAAAAAFPMVLVQIPMFNEREVYKLSIGAACSLDWPSDRVVIQVLDDSTD
LVVKDLVEKECQKWQGKGVNIKYEVRGNRKGYKAGALKEGLKHDYVKECEYIAMFDADFQPESDFLLRTVPFLVHNSEIA
LVQTRWKFVNANECLLTRFQEMSLDYHFKYEQEAGSSVYSFFGFNGTAGVWRIAAIDDAGGWKDRTTVEDMDLAVRATLQ
GWKFVYVGDVKVKSELPSTFKAYRFQQHRWSCGPANLFKKMMVEILENKKVSFWNKIHLWYDFFFVGKIAAHTVTFIYYC
FVIPVSVWLPEIEIPLWGVVYVPTVITLCKAVGTPSSFHLVILWVLFENVMSLHRIKAAVTGILEAGRVNEWVVTEKLGD
ANKTKPDTNGSDAVKVIDVELTTPLIPKLKKRRTRFWDKYHYSEIFVGICIILSGFYDVLYAKKGYYIFLFIQGLAFLIV
GFDYIGVCPP

>Pseudolysobacter_antarcticus_[Glycosyltransferase] InterPro PF00332-PF13641: A0A411HQE7 Glycosyltransferase
MWRALLLSLLVAGLNFALWAVVNRPVQVPDWTGKIKGMAVNWFQRYQSPIKHIYADEADIESDIKLLSQYTSHLRTYTST
EAPSVPRLAAKYGMTVLAGAWLDRRAENSELELQAMIDSARQNKNIDRVIVGNETLLRQDLSPNELIGYLDRARAALKQP
VSTAEPVGIWLRNPKLVKHVDFITVHLLPYWNGVIRRDALGLSVLKDYNDLRARYPDKHILIGEIGWPSNGDRHEYAQPS
IADEAQFLREWFYTADLYNLDYFIEEAFDQPWKEAGEGRVGAYWGMFNSDRQPKFPLTGTVVEDPQWPWKALIGSLLGLG
PMFWFARHFMRFKPAGILFYLILLQLSASLLVWSAMVPYAFYLSPFDWTMLIVLFPAQIAIVMILLINGFEFTEVLWRPR
WMRQFGLLNPLPGDPQPFVSIHLACCNEPPEMVILTLDSLAALEYDNFEVLVLDNNTKEESVWRPVEEHCAKLGAKFRFF
HLSPWPGFKAGALNFGLTETDPRAEAVAVIDADYVVRPDWLRALTGYFANPKVALVQCPQAHRDWEHNAFRRMTNWEYDG
FFRIGMHHRNERNAIIMHGTMTMVRRSALQDTGGWSEWTICEDAELGLRLMHAGYDTLYVDEIMGRGLTPADFTAYKSQR
YRWAFGAMQVLKARWNWMTTKGPLDAGQRFHFLTGWFSWFADALHLVFTMLALVWTAGMIGLPQVFSLPLDLFLIPLLGF
FAFKAAFGVILYRVRVPCSWRDTLSASIASMALSHAIARGIFLGLWKKHGEFVRTAKSRRLHGRPNPFTAVREELLMFVA
IGLGIFGMWHAVGINYIEGKLWIAILAAQAIPYASAMIGAVVAGRSGEKSG

>Pseudomonas_aeruginosa_AZPAE12140_[Orphan] PseudoCAP: Beta-(1-3)-glucosyl transferase
MSSRKIGLNLVVIVALAALFTGIWALYNRPVSVPDWPERISGFSFSPFRLNQNPQSGRYPSAEQMRTDLELVARHTHSIR
TYSVQGALGDIPALAEAFGLRVSLGIWLGPDLASNEAEIARAIRIANESPSVVRVIVGNEALFRREVTAEQLIAYLDRVR
AAVKVPVTTAEQWHVYREHPELAQHVDLIAAHVLPYWEATPVADAVDFVLERARELKAAFPRKPLLLAEVGWPSNGRMRG
SAEATPADQAIYLRRLTNALNGEGYSYFVIEAFDQPWKVSAEGSVGAYWGVYNADRKAKFNFTGPVVPIPKWRALAIASA
VLAVLAFTLLLIDSSSLRQRGRTFLAVVSFACASVLVWIAYDYSQQYSTWFSLTVGALLGVGALGVVIVLFTEAHELAEA

VWTRKRRRPFLPITAAQAYRPKVSIHVPCYNEPPELLKQTLDALARLDYPDYEVLVIDNNTRDPAVWQPVEAHCARLGER
FRFFHVAPLEGFKAGALNFALGHVAADVEVVAVIDADYCVDPDWLRHMVPHFGDPRIAVVQSPQDYRDQHESAFKRLCYA
EYKGFFHIGMVTRNDRDAIIEHGTMTMIRRSVLDELRWPEWCITEDAELGLRVFEKGLSAAYFERSYGKGVMPDTFIDFK
KQRFRWAYGAIQIMKRHTDALLRGRGPDGSRLTRGQRYHFVAGWLPWIADGLNIFFTLGALLWSAAMIIVPKRVDPPLLI
FAILPLALFVFKVGKILFLYRRTVGVDLRDSFFAALAGLSLSHTIAKAVLYGFVTRGIPFFRTPKMRSSHGLLVALAEAR
EEVFVMLLLWGAAAGIVAVQGVPSRDLLIWVAMLLVQSLPYLAALVMALLSSLPKPREELAGGAEQIGG

>Pseudomonas_aeruginosa_BL14_[Orphan] PseudoCAP: Beta-(1-3)-glucosyl transferase
MSSRKIGLNLVVIVALAALFTGIWALYNRPVSVPDWPERISGFSFSPFRLNQNPQSGRYPSAEQMRADLELVARHTHSIR
TYSVQGALGDIPALAEAFGLRVSLGIWLGPDLAGNEAEIARAIRIANESPSVVRVIVGNEALFRREVTAEQLIAYLDRVR
AAVKVPVTTAEQWHVYREHPELAQHVDLIAAHVLPYWEATPVADAVDFVLERARELKAAFPRKPLLLAEVGWPSNGRMRG
SAEATPADQAIYLRRLTNALNGEGYSYFVIEAFDQPWKVSAEGSVGAYWGVYNADRKAKFNFTGPVVPIPKWRALAIVSA
VLAVLAFTLLLIDSSSLRQRGRTFLAVVSFACASVLVWIAYDYSQQYSTWFSLTVGALLGVGALGVVIVLFTEAHELAEA
VWTRKRRRPFLPITAAQAYRPKVSIHVPCYNEPPELLKQTLDALARLDYPDYEVLVIDNNTRDPAVWQPVEAHCARLGER
FRFFHVAPLEGFKAGALNFALGHVAADVEVVAVIDADYCVDPDWLRHMVPHFGDPRIAVVQSPQDYRDQHESAFKRLCYA
EYKGFFHIGMVTRNDRDAIIEHGTMTMIRRSVLDELRWPEWCITEDAELGLRVFEKGLSAAYFERSYGKGVMPDTFIDFK
KQRFRWAYGAIQIMKRHTDALLRGRGPDGSRLTRGQRYHFVAGWLPWIADGLNIFFTLGALLWSAAMIIVPKRVDPPLLI
FAILPLALFAFKVGKILFLYRRTVGVDLRDSFFAALAGLSLSHTIAKAVLYGFVTRGIPFFRTPKMRSSHGLLVALAEAR
EEVFVMLLLWGAAAGIVAVQGVPSRDLLIWVAMLLVQSLPYLAALVMALLSSLPKPREELAGGAEQIGG

>Pseudomonas_aeruginosa_LESB58_[Orphan] PseudoCAP: Putative glucosyl transferase
MSSRKIGLNLVVIVALAALFTGIWALYNRPVSVPDWPERISGLSFSPFRLNQNPQSGRYPSAEQMRTDLELVARHTHSIR
TYSVQGALGDIPALAEAFGLRVSLGIWLGPDLASNEAEIARAIRIANESPSVVRVIVGNEALFRREVTAEQLIAYLDRVR
AAVKVPVTTAEQWHVYREYPELAQHVDLIAAHVLPYWEATPVADAVDFVLERARELKAAFPRKPLLLAEVGWPSNGRMRG
SAEATPADQAIYLRRLTNALNGEGYSYFVIEAFDQPWKVSAEGSVGAYWGVYNADRKAKFNFTGPVVPIPKWRALAIASA
VLAVLAFTLLLIDSSSLRQRGRTFLAVVSFACASVLVWIAYDYSQQYSTWFSLTVGALLGVGALGVVIVLFTEAHELAEA
VWTRKRRRPFLPITAAQAYRPKVSIHVPCYNEPPELLKQTLDALARLDYPDYEVLVIDNNTRDPAVWQPVEAHCARLGER
FRFFHVAPLEGFKAGALNFALGHVAADVEVVAVIDADYCVDPDWLRHMVPHFGDPRIAVVQSPQDYRDQHESAFKRLCYA
EYKGFFHIGMVTRNDRDAIIEHGTMTMIRRSVLDELRWPEWCITEDAELGLRVFEKGLSAAYFERSYGKGVMPDTFIDFK
KQRFRWAYGAIQIMKRHTDALLRGRGPDGSRLTRGQRYHFVAGWLPWIADGLNIFFTLGALLWSAAMIIVPKRVDPPLLI
FAILPLALFVFKVGKILFLYRRTVGVDLRDSFFAALAGLSLSHTIAKAVLYGFVTRGIPFFRTPKMRSSHGLLVALAEAR
EEVFVMLLLWGAAAGIVAVQGVPSRDLLIWVAMLLVQSLPYLAALVMALLSSLPKPREELAGGAERIGG

>Pseudomonas_aeruginosa_PAK_[Orphan] PseudoCAP: Glycosyltransferase
MSSRKIGLNLVVIVALAALFTGIWALYNRPVSVPDWPERISGFSFSPFRLNQNPQSGRYPSAEQMRTDLELVARHTHSIR
TYSVQGALGDIPALAEAFGLRVSLGIWLGPDLASNEAEIARAIRIANESPSVVRVIVGNEALFRREVTAEQLIAYLDRVR
AAVKVPVTTAEQWHVYREHPELAQHVDLIAAHVLPYWEATPVADAVDFVLERARELKAAFPRKPLLLAEVGWPSNGRMRG
SAEATPADQAIYLRRLTNALNGEGYSYFVIEAFDQPWKVSAEGSVGAYWGVYNADRKAKFNFTGPVVPIPKWRALAIASA
VLAVLAFTLLLIDSSSLRQRGRTFLAVVSFACASVLVWIAYDYSQQYSTWFSLTVGALLGVGALGVVIVLFTEAHELAEA
VWTRKRRRPFLPITAAQAYRPKVSIHVPCYNEPPELLKQTLDALARLDYPDYEVLVIDNNTRDPAVWQPVEAHCARLGER
FRFFHVAPLEGFKAGALNFALGHVAADVEVVAVIDADYCVDPDWLRHMVPHFGDPRIAVVQSPQDYRDQHESAFKRLCYA
EYKGFFHIGMVTRNDRDAIIEHGTMTMIRRSVLDELRWPEWCITEDAELGLRVFEKGLSAAYFERSYGKGVMPDTFIDFK
KQRFRWAYGAIQIMKRHTDALLRGRGPDGSRLTRGQRYHFVAGWLPWIADGLNIFFTLGALLWSAAMIIVPKRVDPPLLI
FAILPLALFVFKVGKILFLYRRTVGVDLRDSFFAALAGLSLSHTIAKAVLYGFVTRGIPFFRTPKMRSSHGLLVALAEAR
EEVFVMLLLWGAAAGIVAVQGVPSRDLLIWVAMLLVQSLPYLAALVMALLSSLPKPREELAGGAERIGG

>Pseudomonas_aeruginosa_PA01_[NdvB] PseudoCAP: Synthesis of periplasmic glucan
MSSRKIGLNLVVIVALAALFTGIWALYNRPVSVPDWPERISGFSFSPFRLNQNPQSGRYPSAEQMRTDLELVARHTHSIR
TYSVQGALGDIPALAEAFGLRVSLGIWLGPDLASNEAEIARAIRIANESPSVVRVIVGNEALFRREVTAEQLIAYLDRVR
AAVKVPVTTAEQWHVYREHPELAQHVDLIAAHVLPYWEATPVADAVDFVLERARELKAAFPRKPLLLAEVGWPSNGRMRG
SAEATPADQAIYLRRLTNALNGEGYSYFVIEAFDQPWKVSAEGSVGAYWGVYNADRKAKFNFTGPVVPIPKWRALAIASA
VLAVLAFTLLLIDSSSLRQRGRTFLAVVSFACASVLVWIAYDYSQQYSTWFSLTVGALLGVGALGVVIVLFTEAHELAEA
VWTRKRRRPFLPITAARAYRPKVSIHVPCYNEPPELLKQTLDALARLDYPDYEVLVIDNNTRDPAVWQPVEAHCARLGER
FRFFHVAPLEGFKAGALNFALGHVAADVEVVAVIDADYCVDPDWLRHMVPHFGDPRIAVVQSPQDYRDQHESAFKRLCYA
EYKGFFHIGMVTRNDRDAIIEHGTMTMIRRSVLDELRWPEWCITEDAELGLRVFEKGLSAAYFERSYGKGVMPDTFIDFK
KQRFRWAYGAIQIMKRHTDALLRGRGPDGSRLTRGQRYHFVAGWLPWIADGLNIFFTLGALLWSAAMIIVPKRVDPPLLI
FAILPLALFVFKVGKILFLYRRTVGVDLRDSFFAALAGLSLSHTIAKAVLYGFVTRGIPFFRTPKMRSSHGLLVALAEAR
EEVFVMLLLWGAAAGIVAVQGVPSRDLLIWVAMLLVQSLPYLAALVMALLSSLPKPREELAGGAEQIGG

>Pseudomonas_aeruginosa_PA14_[NdvB] PseudoCAP: Beta-(1-3)-glucosyl transferase

MSSRKIGLNLVVIVALAALFTGIWALYNRPVSVPDWPERISGFSFSPFRLNQNPQSGRYPSAEQMRADLELVARHTHSIR
TYSVQGALGDIPALAEAFGLRVSLGIWLGPDLAGNEAEIARAIRIANESPSVVRVIVGNEALFRREVTAEQLIAYLDRVR
AAVKVPVTTAEQWHVYREHPELAQHVDLIAAHVLPYWEATPVADAVDFVLERARELKAAFPRKPLLLAEVGWPSNGRMRG
SAEATPADQAIYLRRLTNALNGEGYSYFVIEAFDQPWKVSAEGSVGAYWGVYNADRKAKFNFTGPVVPIPKWRALAIASA
VLAVLAFTLLLIDSSSLRQRGRTFLAVVSFACASVLVWIAYDYSQQYSTWFSLTVGALLGVGALGVVIVLFTEAHELAEA
VWTRKRRRPFLPITAAQAYRPKVSIHVPCYNEPPELLKQTLDALARLDYPDYEVLVIDNNTRDPAVWQPVEAHCARLGER
FRFFHVAPLEGFKAGALNFALGHVAADVEVVAVIDADYCVDPDWLRHMVPHFGDPRIAVVQSPQDYRDQHESAFKRLCYA
EYKGFFHIGMVTRNDRDAIIEHGTMTMIRRSVLDELRWPEWCITEDAELGLRVFEKGLSAAYFERSYGKGVMPDTFIDFK
KQRFRWAYGAIQIMKRHTDALLRGRGPDGSRLTRGQRYHFVAGWLPWIADGLNIFFTLGALLWSAAMIIVPKRVDPPLLI
FAILPLALFAFKVGKILFLYRRTVGVDLRDSFFAALAGLSLSHTIAKAVLYGFVTRGIPFRTPKMRSSHGLLVALAEARE
EVFVMLLLWGAAAGIVAVQGVPSRDLLIWVAMLLVQSLPYLAALVMALLSSLPKPREELAGGAEQIGG

>Pseudomonas_aeruginosa_19BR_[Orphan] PseudoCAP: Beta-(1-3)-glucosyl transferase
MSSRKIGLNLVVIVALAALFTGIWALYNRPVSVPDWPERISGFSFSPFRLNQNPQSGRYPSAEQMRTDLELVARHTHSIR
TYSVQGALGDIPALAEAFGLRVSLGIWLGPDLASNEAEIARAIRIANESPSVVRVIVGNEALFRREVTAEQLIAYLDRVR
AAVKVPVTTAEQWHVYREHPELAQHVDLIAAHVLPYWEATPVADAVDFVLERARELKAAFPRKPLLLAEVGWPSNGRMRG
SAEATPADQAIYLRRLTNALNGEGYSYFVIEAFDQPWKVSAEGSVGAYWGVYNADRKAKFNFTGPVVPIPKWRALAIASA
VLAVLAFTLLLIDSSSLRQRGRTFLAVVSFACASVLVWIAYDYSQQYSTWFSLTVGALLGVGALGVVIVLFTEAHELAEA
VWTRKRRRPFLPITAAQAYRPKVSIHVPCYNEPPELLKQTLDALARLDYPDYEVLVIDNNTRNPAVWQPVEAHCARLGER
FRFFHVAPLEGFKAGALNFALGHVAADVEVVAVIDADYCVDPDWLRHMVPHFSDPRIAVVQSPQDYRDQHESAFKRLCYA
EYKGFFHIGMVTRNDRDAIIEHGTMTMIRRSVLDELRWPEWCITEDAELGLRVFEKGLSAAYFERSYGKGVMPDTFIDFK
KQRFRWAYGAIQIMKRHTDALLRGRGLDGSRLTRGQRYHFVAGWLPWIADGLNIFFTLGALLWSAAMIIVPKRVDPPLLI
FAILPLALFAFKVGKILFLYRRTVGVDLRDSFFAALAGLSLSHTIAKAVLYGFVTRGIPFFRTPKMRSSHGLLVALAEAR
EEVFVMLLLWGAAAGIVAVQGVPSRDLLIWVAMLLVQSLPYLAALVMALLSSLPKPREELAGGAERIGG

>Pseudomonas_aeruginosa_3573_[Orphan] PseudoCAP: Beta-(1-3)-glucosyl transferase
MSSRKIGLNLVVIVALAALFTGIWALYNRPVSVPDWPERISGFSFSPFRLNQNPQSGRYPSAEQMRADLELVARHTHSIR
TYSVQGALGDIPALAEAFGLRVSLGIWLGPDLAGNEAEIARAIRIANESPSVVRVIVGNEALFRREVTAEQLIAYLDRVR
AAVKVPVTTAEQWHVYREHPELAQHVDLIAAHVLPYWEATPVADAVDFVLERARELKAAFPRKPLLLAEVGWPSNGRMRG
SAEATPADQAIYLRRLTNALNGEGYSYFVIEAFDQPWKVSAEGSVGAYWGVYNADRKAKFNFTGPVVPIPKWRALAIASA
VLAVLAFTLLLIDSSSLRQRGRTFLAVVSFACASVLVWIAYDYSQQYSTWFSLTVGALLGVGALGVVIVLFTEAHELAEA
VWTRKRRRPFLPITAAQAYRPKVSIHVPCYNEPPELLKQTLDALARLDYPDYEVLVIDNNTRDPAVWQPVEAHCARLGER
FRFFHVAPLEGFKAGALNFALGHVAADVEVVAVIDADYCVDPDWLRHMVPHFGDPRIAVVQSPQDYRDQHESAFKRLCYA
EYKGFFHIGMVTRNDRDAIIEHGTMTMIRRSVLDELRWPEWCITEDAELGLRVFEKGLSAAYFERSYGKGVMPDTFIDFK
KQRFRWAYGAIQIMKRHTDALLRGRSPDGSRLTRGQRYHFVAGWLPWIADGLNIFFTLGALLWSAAMIIVPKRVDPPLLI
FAILPLALFVFKVGKILFLYRRTVGVDLRDSFFAALAGLSLSHTIAKAVLYGFVTRGIPFFRTPKMRSSHGLLVALAEAR
EEVFVMLLLWGAAAGIVAVQGVPSRDLLIWVAMLLVQSLPYLAALVMALLSSLPKPREELAGGAERIGG

>Pseudomonas_alkylphenolia_KL28_[Orphan] PseudoCAP: Beta-(1-3)-glucosyl
transferase
MSSRKFGINLVIVMAIAALFTGFWALINRPVSAPDWPEQISGFSYSPFRLGESPQKGQYPTEDELRQDLEQLSKLTDSIR
IYTVEGTQADIPRLAEEFGLRVTLGVWISPDLERNEREIQKAIELANSSRSVVRVMVGNEALFRKEITPEALIQYLDRVR
AAVKVPVTTSEQWHIWKEHPELAKHVDLVAAHILPYWEFIPMKDAGQFVLDRARDLKQLFPRKPLLLSEVGWPSNGRMRG
GADATPADQAIYLRTLVNKLNRQGYNYFVIEAYDQPWKASDEGSVGAYWGVFNAARQQKFNFEGPVVAIPQWRVLAVGSV
VLAMLSLTLLLIDGSALRQRGRTFLTFIAFLCGSVLVWIGYDYSQQYSTWFSLTVGFLLALGALGVFIVLLTEAHELAEA
VWIHKRRREFLPVQADSAYRPKVSVHVPCYNEPPEMVKQTLNALAALDYPDYEVLIIDNNTKDPAVWEPIKAHCEMLGER
FKFFHVSPLAGFKGGALNYLIPHTAKDAEVIAVIDSDYCVDRNWLKHMVPHFSDPKIAVVQSPQDYRDQNESTFKKLCYS
EYKGFFHIGMVTRNDRDAIIQHGTMTMTRRSVLEELGWADWCICEDAELGLRVFEKGYSAAYSHESYGKGLMPDTFIDFK
KQRFRWAYGAIQIIKRHAASLLRGKDSELTRGQRYHFLAGWLPWIADGMNIFFTVGALLWSAAMIIVPQRVDPPLLIFAI
PPLALFVFKVGKIVFLYRRAVGVNMKDAFAAALAGLALSHTIAKAVLYGFFTSSIPFFRTPKNADSHGLLVALSEAREEL
FIMLLLWGAAAGIYLVQGLPSNDMRFWVTMLLVQSLPYLAALIMAMLSSLPKPVDAAQPAQAS

>Pseudomonas_antarctica_BS2772_[Orphan] BLAST: A0A1H0DGE3 Exo-beta-1,3-
glucanase, GH17 family
MRAVRRRPTPLFVPHPLEPEIAMSSRKFGLNLVVVLAIAALFTGFWALINRPVTAPNWPEQISGFSYSPFQQGQFPQKDQ
YPSDEEMRRDLEIMSKLTDNIRTYSVDGTLGDIPKLAEEFGLRVTLGIWISPDLERNEREIQRAIELANSSRSVVRVVVG
NEALFREEITPQALIVLLDRVRAAVKVPVTTSEQWHIWEKNPQLAKHVDLIAAHILPFWEFIPMDKAGQYVLDRARDLKK
LFPKKPLLLSEVGWPSNGRMRGGNETSPADQAIYLRTLVNKLNRQGYNYFVIEAFDQPWKVSDEGSAGAYWGVYNAARQQ
KFNFEGPVVAIPQWRVLAIGSVVLALLSLTLLMIDGSSLRQRGRTFLTFIAFLCGSVLVWIGYDYSQQYSTWFSVTVGIL
LALGALGVFIVLLTEAHELAEAVWTHKRRREFLPVEGDSDYRPKVSIHVPCYNEPPEMVKQTLDALAALDYPDYEVLIID
NNTKDPAVWEPVRDYCETLGPRFKFFHVAPLAGFKGGALNYLIPHTAKDAEVIAVIDSDYCVSPNWLKHMVPHFADPKIA

VVQSPQDYRDQNESTFKKLCYAEYKGFFHIGMVTRNDRDAIIQHGTMTMTRRSVLEELGWADWCICEDAELGLRVFEKGL
SAAYYHDSYGKGLMPDTFIDFKKQRFRWAYGAIQIIKRHTASLLRGKGTELTRGQRYHFLAGWLPWVADGMNIFFTVGAL
LWSAAMIIVPTRVDPPLLIFAIPPLALFVFKVGKIVFLYRRAVGVNLKDAFCAALAGLALSHTIAKAVLYGFFTTSIPFF
RTPKNADNHGFWVAISEAREEMFIMLLLWGAALGIYLVQGLPSNDIRFWVVMLLVQSLPYVAAMIMAFLSSLPKPAPAVE
PATAE

>Pseudomonas_antarctica_PAMC_27494_[Orphan] BLAST: A0A172YX49 Beta-(1-3)-
glucosyl transferase
MSSRKFGLNLVVVLAIAALFTGFWALINRPVTAPNWPEQISGFSYSPFQQGQFPQKDQYPSDEEMRRDLEIMSKLTDNIR
TYSVDGTLGDIPKLAEEFGLRVTLGIWISPDLERNEREIQRAIELANSSRSVVRVVVGNEALFREEITPQALIVLLDRVR
GAVKVPVTTSEQWHIWEKNPQLAKHVDLIAAHILPFWEFIPMDKAGQYVLDRARDLKKLFPKKPLLLSEVGWPSNGRMRG
GNETSPADQAIYLRTLVNKLNRQGYNYFVIEAFDQPWKVSDEGSAGAYWGVYNAARQQKFNFEGPVVAIPQWRVLAIGSV
VLALLSLTLLMIDGSALRQRGRTFLTFIAFLCGSVLVWIGYDYSQQYSTWFSVTVGILLALGALGVFIVLLTEAHELAEA
VWTHKRRREFLPVEGDSDYRPKVSIHVPCYNEPPEMVKQTLDALAALDYPDYEVLIIDNNTKDPAVWEPVRDYCETLGPR
FKFFHVSPLAGFKGGALNYLIPHTAKDAEVIAVIDSDYCVSPNWLKHMVPHFADPKIAVVQSPQDYRDQNESTFKKLCYA
EYKGFFHIGMVTRNDRDAIIQHGTMTMTRRSVLEELGWADWCICEDAELGLRVFEKGLSAAYYHDSYGKGLMPDTFIDFK
KQRFRWAYGAIQIIKRHTASLLRGKGTELTRGQRYHFLAGWLPWVADGMNIFFTVGALLWSAAMIIVPTRVDPPLLIFAI
PPLALFVFKVGKIVFLYRRAVGVNLKDAFCAALAGLALSHTIAKAVLYGFFTTSIPFFRTPKNADNHGFWVAISEAREEM
FIMLLLWGAALGIYLVQGLPSNDIRFWVVMLLVQSLPYVAALIMAFLSSLPKPAPKGEPATAE

>Pseudomonas_azotoformans_F77_[Orphan] BLAST: A0A1U9PTL1 Beta-(1-3)-glucosyl
transferase
MASRKFGLNLVIVLAIAALFTGFWALINRPVTTPNWPEQISGFSYSPFQQGQYPQKGQYPTDDQMRRDLEIMSKLTDNIR
TYSVDGTLGDIPKLAEEFGLRVTLGIWISPDLERNEREIQRAIEIANSSRSVVRVVVGNEALFREEITPEALIVLLDRVR
AAVKVPVTTSEQWHIWEKNPQLAKHVDLIAAHILPFWEYIPMDKAGQYVLDRARDLKKMFPKKPLLLSEVGWPSNGRMRG
GNETSPADQAIYLRTLVNKLNRQGFNYFVIEAFDQPWKVSDEGSAGAYWGVYNAARQQKFNFDGPVVAIPQWRVLAIGSV
VLALLSLTLLMIDGSALRQRGRTFLTFIAFLCGSVLVWIGYDYSQQYSTWFSVTVGILLALGALGVFIVLLTEAHELAEA
VWTHKRRREFLPVEGDSDYRPKVSIHVPCYNEPPEMVKQTLDALAALDYPDYEVLIIDNNTKDPAVWEPVRDYCDTLGPR
FKFFHVAPLAGFKGGALNYLIPHTAKDAEVIAVIDSDYCVSPNWLKHMVPHFADPKIAVVQSPQDYRDQNESTFKKLCYA
EYKGFFHIGMVTRNDRDAIIQHGTMTMTRRSVLEELGWADWCICEDAELGLRVFEKGLSAAYYHDSYGKGLMPDTFIDFK
KQRFRWAYGAIQIIKRHTASLLRGKDTELTRGQRYHFLAGWLPWVADGMNIFFTVGALLWSAAMIIVPTRVDPPLLIFAI
PPLALFVFKVGKIIFLYRRAVGVNLKDAFCAALAGLALSHTIAKAVLYGFFTTSIPFFRTPKNADNHGFWVAISEAREEM
FIMLLLWGAALGIYLVQGLPSNDIRFWVVMLLVQSLPYVAALVMAFLSSLPKPAPKAEPATAE

>Pseudomonas_azotoformans_S4_[Orphan] BLAST: A0A127HXK8 Beta-(1-3)-glucosyl
transferase
MASRKFGLNLVIVLAIAALFTGFWALINRPVTTPNWPEQISGFSYSPFQQGQYPQKDQYPTDDQMRRDLEIMSKLTDNIR
TYSVDGTLGDIPKLAEEFGLRVTLGIWISPDLERNEREIQRAIEIANSSRSVVRVVVGNEALFREEITPEALIVLLDRVR
AAVKVPVTTSEQWHIWEKNPQLAKHVDLIAAHILPFWEYIPMDKAGQYVLDRARDLKKLFPKKPLLLSEVGWPSNGRMRG
GNETSPADQAIYLRTLVNKLNRQGFNYFVIEAFDQPWKVSDEGSAGAYWGVYNAARQQKFNFEGPVVAIPQWRVLAIGSV
VLALLSLTLLMIDGSALRQRGRTFLTFIAFLCGSVLVWIGYDYSQQYSTWFSVTVGILLALGALGVFIVLLTEAHELAEA
VWTHKRRREFLPVEGDSDYRPKVSIHVPCYNEPPEMVKQTLDALAALDYPDYEVLIIDNNTKDPAVWEPVRDYCETLGPR
FKFFHVAPLAGFKGGALNYLIPHTAKDAEVIAVIDSDYCVSPNWLKHMVPHFADPKIAVVQSPQDYRDQNESTFKKLCYA
EYKGFFHIGMVTRNDRDAIIQHGTMTMTRRSVLEELGWADWCICEDAELGLRVFEKGLSAAYYHDSYGKGLMPDTFIDFK
KQRFRWAYGAIQIIKRHTASLLRGKDTELTRGQRYHFLAGWLPWVADGMNIFFTVGALLWSAAMIIVPTRVDPPLLIFAI
PPLALFVFKVGKIIFLYRRAVGVNLKDAFCAALAGLALSHTIAKAVLYGFFTTSIPFFRTPKNADNHGFWVAISEAREEM
FIMLLLWGAALGIYLVQGLPSNDIRFWVVMLLVQSLPYVAALIMAFLSSLPKPAAAPEPAPAA

>Pseudomonas_balearica_DSM_6083_[Orphan] PseudoCAP: Beta-(1-3)-glucosyl
transferase
MSARKLGLNLVMVVAVASLFTGLWAWFNQPVDAPDWPDQISGYSFSPFRLHQSPQDNIYPSDDEIRADLELLSNQTDNIR
TYSVDGTLGDIPHLAEELGMRVTLGIWLSNDLEANERQIARGIEIARNERSVIRVVVGNEALFREEVTVEQMIGYLDRVR
SALKVPVTTAEQWHIWQKYPELARHVDLIAAHVLPYWEFIPREDSVDFVLERARELKKQFPKKPLLLAEVGWPSNGRTRG
GAEADQAEQAIYLRTLVNKLNAKGFNYFVIEAFDQPWKAGEEGAVGAYWGVYNAARQPKFPLTGPVVEIPQWRLLASASG
ILALLALALLLIDGSALRQRGRTFLTIVAFLAATMLVWIAYDFSQQYSTWFSLTVGVLLALGALGVMVVLLTEAHELAET
VWKRKRVRPFLPVTDETGYRPMVSVHVPCYNEPPEMVKQTLNALARLDYPNFEVLVIDNNTRDPAVWEPVQAHCEALGER
FRFFHVAPLAGFKGGALNYALERTHPDAEVVAVIDSDYCVSPDWLRHMVPHFADPKIAVVQSPQDYRDEHESLFKKLCYA
EYKGFFHIGMVTRNDRDAIIQHGTMTMTRRKVLDELKWADWCITEDAELGLRVFEKGLSAAYFEQSYGKGLMPDTFIDFK
KQRFRWAYGAMQIMKRHLASLFFGKGSELTRGQRYHFVAGWLPWVADGLNIFFTAGALLWSAAMIIVPNRVDPPLLIFAL
PPLALFFFKLGKILFLYRRAVGVNMRDAASAAIAGLALSHTIAKAVLFGMVTRSIPFFRTPKMRSNHGLLLALAEAREEA
FVMLLLWGAALGIAITQPQPGLDVMFWVAVLLIQSLPYVAALAMAMLSSLPKPEQHAQAKPA

>Pseudomonas_canadensis_36C8_[Orphan] BLAST: A0A423F6Q6 Beta-(1-3)-glucosyl transferase
MASRKFGLNLVIVLAIAALFTGFWALINRPVTTPNWPEQISGFSYSPFQQGQYPQKDQYPTDDQMRRDLEIMSKLTDNIR
TYSVDGTLGDIPKLAEEFGLRVTLGIWISPDLERNEREIQRAIEIANSSRSVVRVVVGNEALFREEITPEALIVLLDRVR
AAVKVPVTTSEQWHIWEKNQLAKHVDLIAAHILPFWEYIPMDKAGQYVLDRARDLKKLFPKKPLLLSEVGWPSNGRMRGG
NETSPADQAIYLRTLVNKLNRQGFNYFVIEAFDQPWKVSDEGSAGAYWGVYNAARQQKFNFEGPVVAIPQWRVLAIGSVV
LALLSLTLLMIDGSALRQRGRTFLTFIAFLCGSVLVWIGYDYSQQYSTWFSVTVGILLALGALGVFIVLLTEAHELAEAV
WTHKRRREFLPVEGDSDYRPKVSIHVPCYNEPPEMVKQTLDALAALDYPDYEVLIIDNNTKDPAVWEPVRDYCETLGPRF
KFFHVAPLAGFKGGALNYLIPHTAKDAEVIAVIDSDYCVSPNWLKHMVPHFADPKIAVVQSPQDYRDQNESTFKKLCYAE
YKGFFHIGMVTRNDRDAIIQHGTMTMTRRSVLEELGWADWCICEDAELGLRVFEKGLSAAYYHDSYGKGLMPDTFIDFKK
QRFRWAYGAIQIIKRHTASLLRGKDTELTRGQRYHFLAGWLPWVADGMNIFFTVGALLWSAAMIIVPTRVDPPLLIFAIP
PLALFVFKVGKIIFLYRRAVGVNLKDAFCAALAGLALSHTIAKAVLYGFFTTSIPFFRTPKNADNHGFWVAISEAREEMF
IMLLLWGAALGIYLVQGLPSNDIRFWVVMLLVQSLPYVAALIMAFLSSLPKPAAAPEPAPAA

>Pseudomonas_chlororaphis_PCL1606_[Orphan] PseudoCAP: Beta-(1-3)-glucosyl transferase
MSSRKFGLNLVVVLAIAALFTGFWALINRPVSAPNWPAQISGFSYSPFQLGQFPQKEQYPTDDEMRRDLEIMSKLTDNIR
TYSVDGTLENIPKLAEEFGLRVTLGIWISPDLERNEREITRAIDIANSSRSVVRVVVGNEAIFRKEITADQLSVLLDRVR
AAVKVPVTTSEQWHVWEEHPELAKHVDLIAAHVLPYWEFIPMDKAGQFVLDRARDLKNMFPKKPLLLSEVGWPSNGRMRG
GADASPADQAIYLRNLVNKLNRQGYNYFVIEAFDQPWKASDEGSVGAYWGVFNAARQQKFNFEGPVVAIPQWRVLAIGSV
VLALLSLTLLMIDGSALRQRGRTFLTFIAFLCGSVLVWIGYDYSQQYSTWFSLTVGFLLALGALGVFIVLLTEAHELAEA
VWVHKRRREFLPVEGDSGYRPKVSIHVPCYNEPPEMVKQTLDALANLDYPDYEVLIIDNNTKNPAVWEPVRDYCETLGPR
FKFFHVAPLAGFKGGALNYLIPHTAKDAEVIAVIDSDYCVDRNWLKHMVPHFADPKIAVVQSPQDYRDQNESTFKKLCYA
EYKGFFHIGMVTRNDRDAIIQHGTMTMTRRSVLEELGWADWCICEDAELGLRVFEKGLSAAYYHDSYGKGLMPDTFIDFK
KQRFRWAYGAIQIIKRHTASLLRGKDTELTRGQRYHFLAGWLPWVADGMNIFFTLGALLWSAAMIIVPQRVDPPLLIFAI
PPLALFVFKVGKIIFLYRRAVGVNLKDAFCAALAGLALSHTIAKAVLYGFFTSSIPFFRPKNADNHGFWVAISEAREELF
IMLLLWGAALGIYLVQGGLPSNDMRFWVTMLLVQSLPYVAALVMAFLSSLPKPVAKAEPASAA

>Pseudomonas_chlororaphis_YL-1_[Orphan] PseudoCAP: Beta-(1-3)-glucosyl transferase
MSSRKFGLNLVVVLAIAALFTGFWALVNRPVSAPNWPAQISGFSYSPFQLGQFPQKDQFPTDDEMRRDLEIMSKLTDNIR
TYSVDGTLENIPKLAEEFGLRVTLGIWISPDLERNEREITRAIDIANNSRSVVRVVVGNEAIFRKEITADQLSVLLDRVR
AAVKVPVTTSEQWHVWEEHPELAQHVDLIAAHVLPYWEFIPMDKAGQFVLDRARDLKKMFPKKPLLLSEVGWPSNGRMRG
GADASPADQAIYLRNLVNKLNRQGYNYFVIEAFDQPWKASDEGSVGAYWGVFNAARQQKFNFEGPVVAIPQWRVLAIGSV
VLALLSLTLLMIDGSALRQRGRTFLTFIAFLCGSVLVWIGYDYSQQYSTWFSLTVGFLLALGALGVFIVLLTEAHELAEA
VWVHKRRREFLPVEGDSSYRPKVSIHVPCYNEPPEMVKQTLNALANLDYPDFEVLIIDNNTKDPAVWEPVQAYCETLGPR
FKFFHVAPLAGFKGGALNYLIPHTAKDAEVIAVIDSDYCVDRNWLKHMVPHFADPKIAVVQSPQDYRDQNESTFKKLCYA
EYKGFFHIGMVTRNDRDAIIQHGTMTMTRRSVLEELGWADWCICEDAELGLRVFEKGLSAAYYHESYGKGLMPDTFIDFK
KQRFRWAYGAIQIIKRHTASLLRGKDTELTRGQRYHFLAGWLPWVADGMNIFFTLGALLWSAAMIIVPQRVDPPLLIFAI
PPLALFVFKVGKIIFLYRRAVGVNLKDAFCAAIAGLALSHTIAKAVLYGFFTSSIPFFRTPKNADNHGFWVAISEAREEL
FIMLLLWGAALGIYLVQGGLPSNDMRFWVTMLLVQSLPYVAALVMAFLSSLPKPLAESAPVAAA

>Pseudomonas_chlororaphis_30-84_[Orphan] PseudoCAP: Beta-(1-3)-glucosyl transferase
MSSRKFGLNLVVVLAIAALFTGFWALVNRPVSAPNWPAQISGFSYSPFQLGQFPQKDQFPTDDEMRRDLEIMSKLTDNIR
TYSVDGTLENIPKLAEEFGLRVTLGIWISPDLERNEREITRAIDIANNSRSVVRVVVGNEAIFRKEITADQLSVLLDRVR
AAVKVPVTTSEQWHVWKEHPELAQHADLIAAHVLPYWESIPMDKAGQFVLDRARDLKQMFPKKPLLLSEVGWPSNGRMRG
GADASPADQAIYLRNLVNKLNRQGYNYFVIEAFDQPWKASDEGSVGAYWGVFNAARQQKFNFEGPVVAIPQWRVLAIGSV
VLALLSLTLLMIDGSALRQRGRTFLTFIAFLCGSVLVWIGYDYSQQYSTWFSLTVGFLLALGALGVFIVLLTEAHELAEA
VWVHKRRREFLPVEGDSNYRPKVSIHVPCYNEPPEMVKQTLNALANLDYPDFEVLIIDNNTKDPAVWEPVQAYCETLGPR
FKFFHVAPLAGFKGGALNYLIPHTAKDAEVIAVIDSDYCVDRNWLKHMVPHFADPKIAVVQSPQDYRDQNESTFKKLCYA
EYKGFFHIGMVTRNDRDAIIQHGTMTMTRRSVLEELGWADWCICEDAELGLRVFEKGLSAAYYHTSYGKGLMPDTFIDFK
KQRFRWAYGAIQIIKRHTASLLRGKDTELTRGQRYHFLAGWLPWVADGMNIFFTLGALLWSAAMIIVPQRVDPPLLIFAI
PPLALFVFKVGKIIFLYRRAVGVNLKDAFCAAVAGLALSHTIAKAVLYGFFTSSIPFFRTPKNADNHGFWVAISEAREEL
FIMLLLWGAALGIYLVQGGLPSNDMRFWVTMLLVQSLPYVAALVMAFLSSLPKPLAESAPVAAA

>Pseudomonas_cremoricolorata_ND07_[Orphan] PseudoCAP: Beta-(1-3)-glucosyl transferase
MPSRKFGLNLVVVLAIAALFTGFWALINRPVSAPAWPEQISGFSYSPFRLGESPQRGQYPTDDEMRQDLEQMSKLTDSIR
IYTVEGTQADIPRLAEELGLRVTLGVWISPDQERNEREINKAIELANTSRSVVRVVVGNEALFRKEITAPALIQYLDRVR

AAVKVPVTTSEQWHIWKENPELAGHVDLIAAHILPYWEFIPMRESVTFVLDRARDLKLQFPRKPLLLSEVGWPSNGRMRG
GADATQADQAIYLRTLVNTLNRQGYNYFVIEAYDQPWKASDEGSVGAYWGVFNAERQQKFNFEGPVVAIPQWRALAVASV
VLAMIALTVLLIDGSALRQRGRTFLTFITFLCGSVLVWIAYDYSQQYSTWFSLSVGVLLALGALGVFIVLLTEAHELAEA
VWTHKRRREFLPVEGDSHYRPKVSVHVPCYNEPPEMVKQTLDALAALDYPDYEVLVIDNNTRDPAVWEPLKAHCEALGER
FKFFHVAPLAGFKGGALNYLLPHTAEDAEVIAVIDSDYCVDRNWLKHMVPHFADPKIAVVQSPQDYRDQHESTFKKLCYS
EYKGFFHIGMVTRNDRDAIIQHGTMTMTRRSVLQELGWADWCICEDAELGLRVFEKGLSAAYAHNSYGKGLMPDTFIDFK
KQRFRWAYGAIQIIKHHASALLRGKGSELTRGQRYHFLAGWLPWVADGMNIFFTVGALLWSAAMIIVPHRVDPPLMIFAI
PPLALFFFKVGKIVFLYRRAVGVDLRDAFAAALAGLALSHTIAKAVLYGFFTSSMPFIRTPKHADSHGLFKALAEAREEV
FIMLLLWGAAAGIYLVQGLPSSDMRFWVAMLLVQSLPYLAALVMALMSSLPKPKEAA

>Pseudomonas_cremoris_WS_5106_[Orphan] BLAST: A0A7X1AN88 Glycosyltransferase
MASRKFGLNLVIVLAIAALFTGFWALINRPVTAPNWPEQISGFSYSPFQQGQFPQKDQYPTDDQMRRDLEIMSKLTDNIR
TYSVDGTLGDIPKLAEEFGLRVTLGIWISPDLERNEREIQRAIEIANSSRSVVRVVVGNEALFREEITPEALIVLLDRVR
AAVKVPVTTSEQWHIWEKNPQLAKHVDLIAAHILPFWEYIPMDKAGQYVLDRAKDLKKLFPKKPLLLSEVGWPSNGRMRG
GNETSPADQAIYLRTLVNKLNRQGYNYFVIEAFDQPWKVSDEGSAGAYWGVYNAARQQKFNFEGPVVAIPQWRVLAIGSV
VLALLSLTLLMIDGSALRQRGRTFLTFIAFLCGSVLVWIGYDYSQQYSTWFSVTVGILLALGALGVFIVLLTEAHELAEA
VWTHKRRREFLPVEGDSDYRPKVSIHVPCYNEPPEMVKQTLDALAALDYPDYEVLIIDNNTKDPAVWEPVRDYCETLGPR
FKFFHVAPLAGFKGGALNYLIPHTAKDAEVIAVIDSDYCVSPNWLKHMVPHFADPKIAVVQSPQDYRDQNESTFKKLCYA
EYKGFFHIGMVTRNDRDAIIQHGTMTMTRRSVLEELGWADWCICEDAELGLRVFEKGLSAAYYHDSYGKGLMPDTFIDFK
KQRFRWAYGAIQIIKRHTASLLRGKDTELTRGQRYHFLAGWLPWVADGMNIFFTVGALLWSAAMIIVPTRVDPPLLIFAI
PPLALFVFKVGKIIFLYRRAVGVNLKDAFCAALAGLALSHTIAKAVLYGFFTTSIPFFRTPKNADNHGFWVAISEAREEM
FIMLLLWGAALGIYLVQGLPSNDIRFWVVMLLVQSLPYVAALIMAFLSSLPKPAAEPEPAPAA

>Pseudomonas_deceptionensis_DSM_26521_[Orphan] PseudoCAP: Beta-(1-3)-glucosyl
transferase
MSSRKFGLNLVVVLAIAALFTGFWALINRPVSAPNWPEQISGFSYSPFQQGQYPQKDQYPTDDEMRRDLEILSKLTDNIR
TYSVDGTLGDIPKLAEEFGLRVTIGIWISPDLERNEREIQKAIELANNSRSVVRVVVGNEALFREEITPEDLIVLLDRVR
AAVKVPVTTSEQWHIWEKYPQLAKHVDLIAAHILPYWEFIPVDKAGEFVLDRARDLKKMFPKKPLLLSEVGWPSNGRMRG
GADATPADQAIYLRTLVNTLNRRGYNYFVIEAFDQPWKASDEGSVGAYWGVYNAARQQKFNFEGPVVAIPQWRVLAIGSV
VLALLSLALLLIDGSALRQRGRTFLTFTAFLCGSVLVWIGYDYSQQYSTWFSLTVGFLLGLGALGVFIVLLTEAHELAEA
VWIRKRRREFLPVDSDDAYRPKVSIHVPCYNEPPEMVKQTLNALANLDYPDFEVLIIDNNTKDPAVWEPVRDYCATLGPR
FKFFHVAPLAGFKGGALNYLIPHTAPDAEVIAVIDSDYCVDPNWLKHMVPHFADPKIAIVQSPQDYRDQNESTFKKLCYS
EYKGFFHIGMVTRNDRDAIIQHGTMTMTRRSVLEELGWADWCICEDAELGLRVFEKGYSAAYSHNSYGKGLMPDTFIDFK
KQRFRWAYGAIQIIKRHTSSLLRGKDTELTRGQRYHFLAGWLPWVADGMNIFFTVGALLWSAAMIIVPTRVDPPLLIFAI
PPLALFVFKVGKIIFLYRRAVGVNLKDAFAAALAGLALSHTIAKAVLYGFFTTSIPFFRTPKNADNHGFWVAISEAREEV
FIMLLLWGAALGIFLVQGLPSNDMRFWVVMLLVQSLPYLAALIMAFMSSLPKPVEATEEQPAA

>Pseudomonas_denitrificans_ATCC_13867_[Orphan] PseudoCAP: Family 2 glycosyl
transferase
MPARKFGLNLVVFVALAALFTGFWALYNRPVTVPAWPESISGFSFSPFRLNQNPQKNQFPSDDEIRSDLELVAKQTDNIR
TYSVKGSLADIPRLAEELGMRVSLGIWIGPDEAENEAEIERGIEIANNSRSVVRVIVGNEALFRREVTVEQMTAYLDRVR
KAVKVPVTTAEQWHIYEKYPELAKHVDLIAAHVLPYWEFTPMDDSVQFVLDRARELRARFPRKPLLLAEVGWPSNGRMRG
GADATGADQAIYLRRLTNALNKKGYNYFVVEAFDQPWKVGDEGSVGAYWGVYNAQRQPKFNFEGPVVNIPQWRALAVASV
VMALLALTLLMIDGSALRQRGRTFLTVVAFAGGSVLVWIAYDYSQQYSTWFSLTVGGLLGIGALGVFIVLLTEAHELAET
VWVRKRRRPFDPVLTDTSYRPKVSVHVPCYNEPPEMLKKTLDALAKLDYPDYEVLIIDNNTKDPAVWEPVRDYCEVLGPR
FRFFHVAPLAGFKGGALNYILPHTAPDAEVVAVIDADYCVEPNWLKQMVPHFSDPKIAVVQSPQDYRDGEENVFKKLCYA
EYKGFFHIGMVTRNDRDAIIQHGTMTMIRRTVMDELKWADWTICEDAELGLRVFEKGYSAAYSHQSFGKGLMPDTFIDYK
KQRFRWAYGAIQIMKGHARALFQGKDSKLTLGQRYHFIAGWLPWIADGMNIFFTVGALLWSSAMIIVPKRVDPPLLIFAI
PPLALFFFKFGKIMFLYRRAVGVNLLRSFQAAVAGLALSHTIAKAVLYGAFTKTIPFFRTPKMASNHGILVALAEAREEV
FIMLLLWGAALGIVLVQGVPSRDMMFWVSMLLVQSLPYLAALVMALLSSAPGPQPAPAADAAPAR

>Pseudomonas_entomophila_L48_[Orphan] PseudoCAP: Glycosyl transferase family
protein
MSSRKFGLNLVVVLAIAALFTGFWALINRPVSAPAWPEQISGFSYSPFRLGQSPQKGQYPSEDEIRQDLEQLNKLTDNIR
IYTVEGTQAEVPRLAEELGLRVTLGIWISDDLERNEREIEKAVGLANTSRSVVRVVVGNEALFRKEVTAEQMIGYLDRVR
AAVKVPVTTSEQWHIWKEHPELAKHVDLIAAHILPYWEFMPMKDSVQFVLDRARELRKQFPRKPLLLSEVGWPSNGRMRG
GADATQADQAIYLRTLVNTLNRRGYNYFVIEAYDQPWKASDEGSVGAYWGVFNAARQQKFNFEGPIVAIPQWRALAVVSV
VLAMIALTVLLIDGSALRQRGRTFLTFITFLCGSVLVWIGYDYSQQYSTWFSLTVGVLLALGALGVFIVLLTEAHELAEV
WTHKRRREFLPVQDDSAYRPKVSVHVPCYNEPPEMVKQTLDALAALDYPDYEVLVIDNNTKDPAVWEPLKAHCEKLGERF
KFFHVAPLAGFKGGALNYLIPHTAKDAEVIAVIDSDYCVDRNWLKHMVPHFADPKIAVVQSPQDYRDQHESAFKKLCYSE
YKGFFHIGMVTRNDRDAIIQHGTMTMTRRTVLEELGWAEWCICEDAELGLRVFEKGLSAAYAHNSYGKGLMPDTFIDFKK

QRFRWAYGAIQIIKHHAGALLRGKGSELTRGQRYHFLAGWLPWIADGMNIFFTVGALLWSAAMIIVPHRVDPPLMMFAIP
PLALFFFKVGKILFLYRRAVGVDLKDAFAAALAGLALSHTIAKAVLYGFFTSSMPFFRTPKNADSHGVLVAISEAREELF
IMLMLWGSALGIYLVQGLPSSDMRFWVAMLLVQSLPYLAALVMALLSSLPKPAEKAAEPQQA

>Pseudomonas_extremaustralis_PgKB38_[Orphan] BLAST: A0A5M9IZB4 Cellulose synthase 1 (AcsB) but changed in this work
MSSRKFGLNLVVVLAIAALFTGFWALINRPVTTPNWPEQISGFSYSPFQQGQYPQKDQYPTDDQMRRDLEIMSKLTDNIR
TYSVDGTLGDIPKLAEEFGLRVTLGIWISPDLERNEREIQRAIEIANSSRSVVRVVVGNEALFREEITPEALIVLLDRVR
AAVKVPVTTSEQWHIWEKNPQLAKHVDLIAAHILPFWEYIPMDKAGQYVLDRARDLKKLFPKKPLLLSEVGWPSNGRMRG
GNETSPADQAIYLRTLVNKLNRQGYNYFVIEAFDQPWKVSDEGSAGAYWGVYNAARQQKFNFEGPVVAIPQWRVLAIGSV
VLALLSLTLLMIDGSALRQRGRTFLTFIAFLCGSVLVWIGYDYSQQYSTWFSVTVGILLALGALGVFIVLLTEAHELAEA
VWTHKRRREFLPVVGDSDYRPKVSIHVPCYNEPPDMVKQTLDALAALDYPDYEVLIIDNNTKDPAVWEPVRDYCATLGPR
FKFFHVAPLAGFKGGALNYLIPHTAKDAEVIAVIDSDYCVSPNWLKHMVPHFADPKIAVVQSPQDYRDQNESTFKKLCYA
EYKGFFHIGMVTRNDRDAIIQHGTMTMTRRSVLEELGWADWCICEDAELGLRVFEKGLSAAYYHDSYGKGLMPDTFIDFK
KQRFRWAYGAIQIIKRHTASLLRGKDTELTRGQRYHFLAGWLPWVADGMNIFFTVGALLWSAAMIIVPTRVDPPLLIFAI
PPLALFVFKVGKIIFLYRRAVGVNLKDAFCAALAGLALSHTIAKAVLYGFFTTSIPFFRTPKNADNHGFWVAISEAREEM
FIMLLLWGAALGIYLVQGLPSNDMRFWVVMLLVQSLPYVAALIMAFLSSLPKPAPKVEPVTVA

>Pseudomonas_fluorescens_ICMP_3512_[Orphan] BLAST: A0A3M3Y0A6 Glyco_trans_2-like domain-containing protein
MEPEIAMSSRKFGLNLVVVLAIAALFTGFWALINRPVTAPNWPEQISGFSYSPFQQGQFPQKDQYPSDEEMRRDLEIMSK
LTDNIRTYSVDGTLGDIPKLAEEFGLRVTLGIWISPDLERNEREIQRAIELANSSRSVVRVVVGNEALFREEITPQALIV
LLDRVRAAVKVPVTTSEQWHIWEKNPQLAKHVDLIAAHILPFWEFIPVDKAGQYVLDRAKDLKKLFPKKPLLLSEVGWPS
NGRMRGGNETSPADQAVYLRTLVNKLNRQGYNYFVIEAFDQPWKVSDEGSAGAYWGVYNAARQQKFNFEGPVVAIPQWRV
LAIGSVVLALLSLTLLMIDGSALRQRGRTFLTFIAFLCGSVLVWIGYDYSQQYSTWFSVTVGILLALGALGVFIVLLTEA
HELAEAVWTHKRRREFLPVEGDSDYRPKVSIHVPCYNEPPEMVKQTLDALAALDYPDYEVLIIDNNTKDPAVWEPVRDYC
ETLGPRFKFFHVSPLAGFKGGALNYLIPHTAKDAEVIAVIDSDYCVSPNWLKHMVPHFADPKIAVVQSPQDYRDQNESTF
KKLCYAEYKGFFHIGMVTRNDRDAIIQHGTMTMTRRSVLEELGWADWCICEDAELGLRVFEKGLSAAYYHDSYGKGLMPD
TFIDFKKQRFRWAYGAIQIIKRHTASLLRGKGTELTRGQRYHFLAGWLPWVADGMNIFFTVGALLWSAAMIIVPTRVDPP
LLIFAIPPLALFVFKVGKIIFLYRRAVGVNLKDAFCAALAGLALSHTIAKAVLYGFFTTSIPFFRTPKNADNHGFWVAIS
EAREEMFIMLLLWGAALGIYLVQGLPSNDIRFWVVMLLVQSLPYVAALIMAFLSSLPKPAPKGEPATAE

>Pseudomonas_fluorescens_ICMP_11288_[Orphan] BLAST: A0A0W0HGY2 Beta-(1-3)-glucosyl transferase
MSSRKFGLNLVVVLAIAALFTGFWALINRPVTTPNWPEQISGFSYSPFQQGQFPQKDQYPTDDQMRQDLAIMSKLTDNIR
TYSVDGTLGDIPKLAEEFGLRVTLGIWISPDLERNEREIQRAIEIANTSRSVVRVVVGNEALFREEITPEALIVLLDRVR
AAVKVPVTTSEQWHIWEKNPQLAKHVDLIAAHILPFWEFIPMDKAGQYVLDRARDLKKLFPKKPLLLSEVGWPSNGRMRG
GNESSPADQAIYLRTLVNKLNRQGYNYFVIEAFDQPWKVSDEGSAGAYWGVYNAARQQKFNFEGPVVAIPQWRVLAIGSV
VLALLSLTLLMIDGSALRQRGRTFLTFIAFLCGSVLVWIGYDYSQQYSTWFSVTVGILLALGALGVFIVLLTEAHELAEA
VWTHKRRREFLPVEGDSHYRPKVSIHVPCYNEPPEMVKQTLDALAALDYPDFEVLIIDNNTKDPAVWEPVRDYCETLGPR
FKFFHVAPLAGFKGGALNYLIPHTAKDAEVIAVIDSDYCVSPNWLKHMVPHFADPKIAVVQSPQDYRDQNESTFKKLCYA
EYKGFFHIGMVTRNDRDAIIQHGTMTMTRRSVLEELGWADWCICEDAELGLRVFEKGLSAAYYHDSYGKGLMPDTFIDFK
KQRFRWAYGAIQIIKRHTASLLRGKGTELTRGQRYHFLAGWLPWVADGMNIFFTVGALLWSAAMIIVPTRVDPPLLIFAI
PPLALFVFKVGKIIFLYRRAVGVNLKDAFCAALAGLALSHTIAKAVLYGFFTTSIPFFRTPKNADNHGFWVAISEAREEM
FIMLLLWGAALGIYLVQGLPSNDIRFWVVMLLVQSLPYVAALIMAFLSSLPKPAPKVEPATAE

>Pseudomonas_fluorescens_KF1_[Orphan] BLAST: A0A7M2JCF1 Glycosyltransferase
MSSRKFGLNLVVVLAIAALFTGFWALINRPVTTPNWPEQISGFSYSPFQQGQYPQKDQYPTDDQMRRDLEIMSKLTDNIR
TYSVDGTLGDIPKLAEEFGLRVTLGIWISPDLERNEREIQRAIEIANSSRSVVRVVVGNEALFREEITPEALIVLLDRVR
AAVKVPVTTSEQWHIWEKNPQLAKHVDLIAAHILPFWEYIPMDKAGQYVLDRARDLKKLFPKKPLLLSEVGWPSNGRMRG
GNESSPADQAIYLRTLVNKLNRQGYNYFVIEAFDQPWKVSDEGSAGAYWGVYNAARQQKFNFEGPVVAIPQWRVLAIGSV
VLALLSLTLLMIDGSALRQRGRTFLTFIAFLCGSVLVWIGYDYSQQYSTWFSVTVGILLALGALGVFIVLLTEAHELAEA
VWTHKRRREFLPVVGDSDYRPKVSIHVPCYNEPPEMVKQTLDALAALDYPDYEVLIIDNNTKDPAVWEPVRDYCATLGPR
FKFFHVAPLAGFKGGALNYLIPHTAKDAEVIAVIDSDYCVSPNWLKHMVPHFADPKIAVVQSPQDYRDQNESTFKKLCYA
EYKGFFHIGMVTRNDRDAIIQHGTMTMTRRSVLEELGWADWCICEDAELGLRVFEKGLSAAYYHDSYGKGLMPDTFIDFK
KQRFRWAYGAIQIIKRHTASLLRGKDTELTRGQRYHFLAGWLPWVADGMNIFFTVGALLWSAAMIIVPTRVDPPLLIFAI
PPLALFVFKVGKIIFLYRRAVGVNLKDAFCAALAGLALSHTIAKAVLYGFFTTSIPFFRTPKNADNHGFWVAISEAREEM
FIMLLLWGAALGIYLVQGLPSNDMRFWVVMLLVQSLPYVAALIMAFLSSLPKPAPKVEPVTVA

>Pseudomonas_fluorescens_LMG_5329_[Orphan] BLAST: A0A0A1YUI3 Beta-(1-3)-glucosyl transferase

MASRKFGLNLVIVLAIAALFTGFWALINRPVTTPNWPEQISGFSYSPFQQGQYPQKDQYPTDDQMRRDLEIMSKLTDNIR
TYSVDGTLGDIPKLAEEFGLRVTLGIWISPDLERNEREIQRAIEIANSSRSVVRVVVGNEALFREEITPEALIVLLDRVR
AAVKVPVTTSEQWHIWEKNPQLAKHVDLIAAHILPFWEYIPMDKAGQYVLDRARDLKKLFPKKPLLLSEVGWPSNGRMRG
GNESSPADQAIYLRTLVNKLNRQGFNYFVIEAFDQPWKVSDEGSAGAYWGVYNAARQQKFNFEGPVVAIPQWRVLAIGSV
VLALLSLTLLMIDGSALRQRGRTFLTFIAFLCGSVLVWIGYDYSQQYSTWFSVTVGILLALGALGVFIVLLTEAHELAEA
VWTHKRRREFLPVEGDSDYRPKVSIHVPCYNEPPEMVKQTLDALAALDYPDYEVLIIDNNTKDPAVWEPVRDYCETLGPR
FKFFHVAPLAGFKGGALNYLIPHTAKDAEVIAVIDSDYCVSPNWLKHMVPHFADPKIAVVQSPQDYRDQNESTFKKLCYA
EYKGFFHIGMVTRNDRDAIIQHGTMTMTRRSVLEELGWADWCICEDAELGLRVFEKGLSAAYYHDSYGKGLMPDTFIDFK
KQRFRWAYGAIQIIKRHTASLLRGKDTELTRGQRYHFLAGWLPWVADGMNIFFTVGALLWSAAMIIVPTRVDPPLLIFAI
PPLALFVFKVGKIIFLYRRAVGVNLKDAFCAALAGLALSHTIAKAVLYGFFTTSIPFFRTPKNADNHGFWVAISEAREEM
FIMLLLWGAALGIYLVQGLPSNDIRFWVVMLLVQSLPYVAALIMAFLSSLPKPAAAPEPAPAA

>Pseudomonas_fluorescens_SBW25_[BcsA] PseudoCAP: Cellulose synthase catalytic subunit
MTDTTSSTPFVEGRAEQRLNGAIARFNRWPSAPRTVLVVASCVLGAMLLLGIISAPLDLYSQCLFAAVCFLAVLVLRKIP
GRLAILALVVLSLVASLRYMFWRLTSTLGFETWVDMFFGYGLVAAEFYALIVLIFGYVQTAWPLRRTPVWLKTEPEEWPT
VDVFIPTYNEALSIVKLTIFAAQAMDWPKDKLRVHVLDDGRRDDFREFCRKVGVNYIRRDNNFHAKAGNLNEALKVTDGE
YIALFDADHVPTRSFLQVSLGWFLKDPKLAMLQTPHFFFSPDPFEKNLDTFRAVPNEGELFYGLVQDGNDLWNATFFCGS
CAVIRREPLLEIGGVAVETVTEDAHTALKLNRLGYNTAYLAIPQAAGLATESLSRHINQRIRWARGMAQIFRTDNPLLGK
GLKWGQRICYANAMLHFFYGLPRLVFLTAPLAYLIFGAEIFHASALMIVAYVLPHLVHSSLTNSRIQGRFRHSFWNEVYE
TVLAWYILPPVLVALVNPKAGGFNVTDKGGIIDKQFFDWKLARPYLVLLAVNLIGLGFGIHQLIWGDASTAVTVAINLTW
TLYNLIITSAAVAVASEARQVRSEPRVSAKLPVSIICADGRVLDGTTQDFSQNGFGLMLSDGHSITQGERVQLVLSRNGQ
DSLFDARVVFSKGAQIGAQFEALSLRQQSELVRLTFSRADTWAASWGAGQPDTPLAALREVGSIGIGGLFTLGRATLHEL
RLALSRTPTKPLDTLMDKP

>Pseudomonas_fluorescens_SBW25_[Orphan] PseudoCAP: Putative beta-(1-3)-glucosyl transferase
MASRKFGLNLVIVLAIAALFSGFWALINRPVTAPNWPEQISGFSYSPFQQGQYPQKDQYPTDDQMRRDLEIMSKLTDNIR
TYSVDGTLGDIPKLAEEFGLRVTLGIWISPDLERNEREIQRAIEIANSSRSVVRVVVGNEALFREEITPEALIVLLDRVR
AAVKVPVTTSEQWHIWEKNPQLAKHVDLIAAHILPFWEYIPMDKAGQYVLDRARDLKKLFPKKPLLLSEVGWPSNGRMRG
GNETSPADQAIYLRTLVNKLNRQGFNYFVIEAFDQPWKVSDEGSAGAYWGVYNAARQQKFNFDGPVVAIPQWRVLAIGSV
VLALLSLTLLMIDGSSLRQRGRTFLTFIAFLCGSVLVWIGYDYSQQYSTWFSVTVGILLALGALGVFIVLLTEAHELAEA
VWTHKRRREFLPVEGDSDYPKVSIHVPCYNEPPEMVKQTLDALAALDYPDYEVLIIDNNTKDPAVWEPVRDYCETLGPRF
KFFHVAPLAGFKGGALNYLIPHTAKDAEVIAVIDSDYCVSPNWLKHMVPHFADPKIAVVQSPQDYRDQNESTFKKLCYAE
YKGFFHIGMVTRNDRDAIIQHGTMTMTRRSVLEELGWAWCICEDAELGLRVFEKGLSAAYYHDSYGKGLMPDTFIDFKKQ
RFRWAYGAIQIIKRHTASLLRGKGTELTRGQRYHFLAGWLPWVADGMNIFFTVGALLWSAAMIIVPTRVDPPLLIFAPPL
ALFVFKVGKIIFLYRRAVGVNLKDAFCAALAGLALSHTIAKAVLYGFFTTSIPFFRTPKNADNHGFWVAISEAREEMFIM
LLLWGAALGIYLVQGLPSNDIRFWVVMLLVQSLPYVAALVMAFLSSLPKPAPKVELATAE

>Pseudomonas_fluorescens_SS101_[Orphan] PseudoCAP: Beta-(1-3)-glucosyl transferase
MASRKFGLNLVVVLAIAALFTGFWALINRPVTAPNWPDQISGFSYSPFQQGQYPQKDQYPTDEQMRQDLAIMSKLTDNIR
TYSVDGPLGDIPKLAEEFGLRVTLGIWISPDLERNEREIQRAIEIANTSRSVVRVVVGNEALFREEITPEALIVLLDRVR
AAVKVPVTTSEQWHIWEKNPQLAKHVDLIAAHILPFWEYIPMDKAGQYVLDRAKDLKKAFPKKPLLLSEVGWPSNGRMRG
GNESSPADQAIYLRTLVNKLNRQGYNYFVIEAFDQPWKVSDEGSAGAYWGVFNAARQQKFNFDGPVVAIPQWRVLAIGSV
VLALLSLTLLMIDGSALRQRGRTFLTFIAFLCGSVLVWIGYDYSQQYSTWFSVTVGVLLALGALGVFIVLLTEAHELAEA
VWTHKRRREFLPVEGDSDYRPKVSIHVPCYNEPPEMVKQTLDALAALDYPDYEVLIIDNNTKDPAVWEPVRDYCETLGPR
FKFFHVAPLAGFKGGALNYLIPHTAKDAEVIAVIDSDYCVSPNWLKHMVPHFADPKIAVVQSPQDYRDQNESTFKKLCYA
EYKGFFHIGMVTRNDRDAIIQHGTMTMTRRSVLEELGWADWCICEDAELGLRVFEKGLSAAYYHDSYGKGLMPDTFIDFK
KQRFRWAYGAIQIIKRHTASLIRGKDTELTRGQRYHFLAGWLPWVADGMNIFFTVGALLWSAAMIIVPTRVDPPLLIFAI
PPLALFVFKVGKIIFLYRRAVGVNLKDAFCAALAGLALSHTIAKAVLYGFFTSSIPFFRTPKNADNHGFWVAISEAREEM
FIMLLLWGAALGIYLVQGLPSNDIRFWVVMLLVQSLPYVAALIMAFLSSLPKPSAAPEAAPAA

>Pseudomonas_fluorescens_WH6_[Orphan] PseudoCAP: Beta-(1-3)-glucosyl transferase
MASRKFGLNLVVVLAIAALFTGFWALINRPVTAPNWPEQISGFSYSPFQQGQYPQKEQYPTDEQMRQDLAIMSKLTDNIR
TYSVDGTLGDIPKLAEEFGLRVTLGIWISPDLERNEREIQRAIEIANSSRSVVRVVVGNEALFREEITPEALIVLLDRVR
AAVKVPVTTSEQWHIWEKNPQLAKHVDLIAAHILPFWEFIPMDKAGQYVLDRARDLKKLFPKKPLLLSEVGWPSNGRMRG
GNEASPADQAIYLRTLVNKLNRQGYNYFVIEAFDQPWKVSDEGSAGAYWGVFNAARQQKFNFEGPVVAIPQWRVLAIGSV
VLALLSLTLLMIDGSALRQRGRTFLTFIAFLCGSVLVWIGYDYSQQYSTWFSVTVGILLALGALGVFIVLLTEAHELAEA
VWTHKRRREFLPVEGDSDYRPKVSIHVPCYNEPPEMVKQTLDALAALDYPDYEVLIIDNNTKDPAVWEPVRDYCETLGPR
FKFFHVAPLAGFKGGALNYLIPHTAKDAEVIAVIDSDYCVSPNWLKHMVPHFADPKIAVVQSPQDYRDQNESTFKKLCYA

EYKGFFHIGMVTRNDRDAIIQHGTMTMTRRSVLEELGWADWCICEDAELGLRVFEKGLSAAYYHDSYGKGLMPDTFIDFK
KQRFRWAYGAIQIIKRHTASLLRGKDTELTRGQRYHFLAGWLPWVADGMNIFFTVGALLWSAAMIIVPTRVDPPLLIFAI
PPLALFVFKVGKIIFLYRRAVGVNLKDAFCAALAGLALSHTIAKAVLYGFFTTSIPFFRTPKNADNHGFWVAISEAREEM
FIMLLLWGAALGIYLVQGLPSNDMRFWVVMLLVQSLPYVAALIMAFLSSLPKPAPAPEPAPAS

>Pseudomonas_fluorescens_WS_5037_[Orphan] BLAST: A0A1T2ZT86 Beta-(1-3)-glucosyl transferase
MSSRKFGLNLVVVLAIAALFTGFWALINRPVTAPNWPEQISGFSYSPFQQGQFPQKDQYPSDEEMRRDLEIMSKLTDNIR
TYSVDGTLGDIPKLAEEFGLRVTLGIWISPDLERNEREIQRAIELANSSRSVVRVVVGNEALFREEITPQALIVLLDRVR
AAVKVPVTTSEQWHIWEKNPQLAKHVDLIAAHILPFWEFIPVDKAGQYVLDRAKDLKKLFPKKPLLLSEVGWPSNGRMRG
GNETSPADQAVYLRTLVNKLNRQGYNYFVIEAFDQPWKVSDEGSAGAYWGVYNAARQQKFNFEGPVVAIPQWRVLAIGSV
VLALLSLTLLMIDGSALRQRGRTFLTFIAFLCGSVLVWIGYDYSQQYSTWFSVTVGILLALGALGVFIVLLTEAHELAEA
VWTHKRRREFLPVEGDSDYRPKVSIHVPCYNEPPEMVKQTLDALAALDYPDYEVLIIDNNTKDPAVWEPVRDYCETLGPR
FKFFHVSPLAGFKGGALNYLIPHTAKDAEVIAVIDSDYCVSPNWLKHMVPHFADPKIAVVQSPQDYRDQNESTFKKLCYA
EYKGFFHIGMVTRNDRDAIIQHGTMTMTRRSVLEELGWADWCICEDAELGLRVFEKGLSAAYYHDSYGKGLMPDTFIDFK
KQRFRWAYGAIQIIKRHTASLLRGKGTELTRGQRYHFLAGWLPWVADGMNIFFTVGALLWSAAMIIVPTRVDPPLLIFAI
PPLALFVFKVGKIIFLYRRAVGVNLKDAFCAALAGLALSHTIAKAVLYGFFTTSIPFFRTPKNADNHGFWVAISEAREEM
FIMLLLWGAALGIYLVQGLPSNDIRFWVVMLLVQSLPYVAALIMAFLSSLPKPAPKGEPATAE

>Pseudomonas_fulva_NBRC_16636_[Orphan] PseudoCAP: Beta-(1-3)-glucosyl transferase
MSSRKFGLNLVVVLAIAALFTGFWALINRPVSAPAWPEQISGFSYSPFRLGESPQKGQYPDDNEMRQDLEQMSKLTDSIR
IYTVEGTQAHIPKLAEEFGLRVTVGIWISPDLERNEREIATAIELANTSRSVVRVVVGNEALFREEVTPENLIQYLDRVR
AAVKVPVTTSEQWHIWKQNPQLAKHVDLIAAHILPYWEFVPMKDSVEFVLDRARELKHQFPRKPLLLSEVGWPSNGRMRG
GADATQADQAIYLRTLVNTLNRRGFNYFVIEAYDQPWKASDEGSVGAYWGVFNAERQQKFNFEGPVVAIPQWRALAVASV
VLAMIALMVLFIDGSALRQRGRTFLTFITFLCGSVLVWIAYDYSQQYSTWFSLTVGVLLALGALGVFIVLLTEAHELAEA
VWTHKRRREFLPVHGDSAYRPKVSVHVPCYNEPPEMVKQTLDALAALDYPDYEVLVIDNNTKDLAVWEPLKAHCEKLGER
FRFFHVAPLAGFKGGALNYLLPHTAKDAEVIAVIDSDYCVDRNWLKHMVPHFADPKIAVVQSPQDYRDQHESAFKKLCYS
EYKGFFHIGMVTRNDRDAIIQHGTMTMTRRSVLDELGWAEWCICEDAELGLRVFEKGLSAAYAHNSYGKGLMPDTFIDFK
KQRFRWAYGAIQIIKHHAAALLRGKGSELTRGQRYHFLAGWLPWIADGMNIFFTVGALLWSAAMIIVPHRVDPPLMIFAI
PPLALFFFKVGKIVFLYRRAVGVNLKDALAAALAGLALSHTIAKAVLYGFFTSSMPFFRTPKNADSHGLLVAISEAREEL
FIMLLLWGAAAGIYLVQGLPSSDMRFWVAMLLVQSLPYLAALVMAFLSSLPKPEAKATEKAVETP

>Pseudomonas_helleri_DSM_29165_[Orphan] PseudoCAP: Beta-(1-3)-glucosyl transferase
MSSRKFGLNLVVVLAIAALFTGFWALINRPVSAPNWPDQISGFSYSPFQQGQYPQKDQYPTDDEMRRDLEIMSKLTDNIR
TYSVDGTLGDIPKLAEEFGLRVTLGIWISPDLERNEREIQKAIELANNSRSVVRVVVGNEALFREEITPEDLAVLLDRVR
AAVKVPVTTSEQWHIWEKYPQLAKHVDLIAAHILPYWEFIPVDKAGQFVLDRARDLKKLFPKKPLLLSEVGWPSNGRMRG
GADASPADQAIYLRTLVNNLNRRGYNYFVIEAFDQPWKASDEGSVGAYWGVFNAARQQKFNFEGPVVAIPQWRVLAIGSV
VMGLLSLALLLIDGSALRQRGRTFLTFTAFLCGSALVWIGYDYSQQYSTWFSLTVGFLLGLGALGVFIVLLTEAHELAEA
VWIRKRRREFLPVESDDAYRPKVSIHVPCYNEPPEMVKQTLNALANLDYPDFEVLLIDNNTKDPAVWEPVRDYCATLGPR
FKFFHVAPLAGFKGGALNYLIPHTAPDAEVIAVIDSDYCVDRNWLKHMVPHFADPKIAIVQSPQDYRDHSESTFKKLCYS
EYKGFFHIGMVTRNDRDAIIQHGTMTMTRRSVLEELGWADWCICEDAELGLRVFEKGYSAAYSHNSYGKGLMPDTFIDFK
KQRFRWAYGAIQIIKRHASSLLLGKNTELTRGQRYHFLAGWLPWVADGMNIFFTVGALLWSAAMIIVPTRVDPPLLIFAI
PPLALFVFKVGKIIFLYRRAVGVNLKDAFAAALAGLALSHTIAKAVLYGFFTTSIPFFRTPKNADNHGFWVAISEAREEV
FIMLLLWGAALGIFLVQGLPSNDMRFWVVMLMVQSLPYLAALIMAFTSSLPKPVEAEQEQPAA

>Pseudomonas_knackmussii_B13_[Orphan] PseudoCAP: Beta-(1-3)-glucosyl transferase
MSSRKLGLNLVVLVALAALFTGAWALYNRPVSAPDWPETISGFSFSPFRLDQSPQSGNFPTDDQIRSDLELISKQTDNIR
TYSTKGTLADIPFLAEEYGMRVSLGIWIGPDEAENEAEIARGIEIANRSRSVVRVIVGNEALFRREVTRQQLIGYLDRVR
AAVKVPVTTAEQWHIYRKYPELAKHVDLIAAHVLPYWEFVPMEDSVQFVLDRARELRAEFPKKPLLLGEVGWPSNGRMRG
GATATQSDQAIYLRELTNALNKKGYSYFVVEAFDQPWKYTDEGSVGAYWGVYNAERQPKFNFTGPVVAIPKWRTLAIASV
VLALLTFTLLLIDGSALRQRGRTFLAVVSFACASVLVWIGYDYSQQYSTWFSITVGVLLGIGALGVVIVLFTEAHELAEA
VWTRRRRRLFLPVTADEAYRPKVSIHVPCYNEPPEMLKETLNALAKLDYPDFEVLVIDNNTKDPAVWEPVQAHCQLLGPR
FRFFHVAPLAGFKGGALNYALQFVAPDAEVIAVIDSDYCVDPDWLKHMVPHFADPQIAVVQSPQDYRDQHESTFKRLCYA
EYKGFFHIGMVTRNDRDAIIEHGTMTMVRRQVLDELKWAEWCITEDAELGLRVFERGLSAAYFERSYGKGLMPDTFIDFK
KQRFRWAYGAIQIMKRHTDALLRGRSPDGSKLTGGQRYHFVAGWLPWIADGMNIFFTIGALLWSAAMIIVPKRVDPPLLI
FAILPLTLFAFKVGKILFLYRRTVGVNLRDALFAALAGLSLSHTIAKAVLYGFVTSSIPFFRTPKMRSSHGLMVALAEAR
EEVFVMLLLWGAALGIVLVQGVVDPDLLFWVVMLLVQSLPYLAALIMALLSSLPKPREEEVLSGSEQVG

>Pseudomonas_lini_DSM_16768_[Orphan] PseudoCAP: Beta-(1-3)-glucosyl transferase

MSSRKFGLNLVVVLAIAALFTGFWALVNRPVTAPNWPEQISGFSYSPFQQGQYPQKAQWPTDDEMRRDLEIMSKLTDNIR
TYSVDGTLENIPKLAEEFGLRVTLGIWISPDEERNEREITRAIEIANTSRSVVRVIVGNEAIFRKEITAAELSLILDRVR
AAVKVPVTTSEQWHVWQENPSLAKHVDLIAAHILPYWEFVPVDKAGQFVLDRARDLKKMFPKKPLLLSEVGWPSNGRMRG
GADASPADQAIYLRTLVNKLNRQGFNYFVIEAFDQPWKASDEGSVGAYWGVFNAARQQKFNFEGPVVAIPQWRVLAIGSV
VLALLSLTLLMIDGSALRQRGRTFLTFIAFLCGSVLVWIGYDYSQQYSTWFSLTVGFLLALGALGVFIVLLTEAHELAEA
VWTHKRRREFLPVVGDSDYRPKVSIHVPCYNEPPEMVKQTLNALANLDYPDFEVLIIDNNTKDPAVWEPVRDYCETLGPR
FKFFHVAPLAGFKGGALNYLIPHTAKDAEVIAVIDSDYCVDPNWLKHMVPHFADPKIAVVQSPQDYRDQNESTFKKLCYA
EYKGFFHIGMVTRNDRDAIIQHGTMTMTRRSVLEELGWADWCICEDAELGLRVFEKGLSAAYYHDSYGKGLMPDTFIDFK
KQRFRWAYGAIQIIKRHTASLLRGKDTELTRGQRYHFLAGWLPWVADGMNIFFTIGALLWSSAMIIVPQRVDPPLLIFAI
PPLALFVFKVGKIIFLYRRAVGVNLKDAFCAALAGLALSHTIAKAVLYGFFTSSIPFFRTPKNADNHGFWVAISEAREEV
FIMLLLWGAALGIFLVNGLPSNDMRFWVTMLLVQSLPYLAALIMAFLSSLPKPVAKPEPATA

>Pseudomonas_lundensis_DSM_6252_[Orphan] PseudoCAP: Beta-(1-3)-glucosyl
transferase
MSSRKFGLNLVVVLAIAALFTGFWALINRPVSAPNWPEQISGFSYSPFQQGQYPQKDQYPSDDEMRRDLEIMSKLTDNIR
TYSVDGTLGDIPKLAEEFGLRVTIGIWISPDQARNEREIQKAIELANNSRSVVRVVVGNEALFREEITPEELIVLLDRVR
AAVKVPVTTSEQWHIWEKYPQLAKHVDLIAAHILPYWEFIPVDKAGEFVLDRARDLKKLFPKKPLLLSEVGWPSNGRMRG
GADASPADQAIYLRNLVNTLNRRGYNYFVIEAFDQPWKASDEGSVGAYWGVYNAARQQKFNFEGPVVAIPQWRVLAIGSV
VMGLLSLALLLIDGSALRQRGRTFLTFTAFLCGSVLVWIGYDYSQQYSTWFSLTVGFLLGLGALGVFIVLLTEAHELAEA
VWIRKRRREFLPVDSDNAYRPKVSIHVPCYNEPPAMVKQTLDALANLDYPDFEVLIIDNNTKDPAVWEPVRDYCATLGPR
FKFFHVAPLAGFKGGALNYLIPHTAPDAEVIAVIDSDYCVDRNWLKHMVPHFADPKIAIVQSPQDYRDQTESTFKKLCYS
EYKGFFHIGMVTRNDRDAIIQHGTMTMTRRSVLEELGWADWCICEDAELGLRVFEKGYSAAYSHNSYGKGLMPDTFIDFK
KQRFRWAYGAIQIIKRHAASLLWGKGSQLTRGQRYHFLAGWLPWVADGMNIFFTVGALLWSAAMIIVPTRVDPPLLIFAI
PPLALFVFKVGKIIFLYRRAVGVNLKDAFAAALAGLALSHTIAKAVLYGFFTTSIPFFRTPKNADNHGFWVAISEAREEV
FIMLLLWGAAVGIFFVQGLPSNDMRFWVVMLLVQSLPYLAALVMAFMSSLPKPVEAPEELPAA

>Pseudomonas_lurida_LMG_21995_[Orphan] BLAST: A0A2A9D9Q0 Exo-beta-1,3-glucanase
(GH17 family)
MASRKFGLNLVIVLAIAALFTGFWALINRPVTTPNWPEQISGFSYSPFQQGQYPQKDQYPTDDQMRRDLEIMSKLTDNIR
TYSVDGTLGDIPKLAEEFGLRVTLGIWISPDLERNEREIQRAIEIANSSRSVVRVVVGNEALFREEITPEALIVLLDRVR
AAVKVPVTTSEQWHIWEKNPKLANHVDLIAAHILPFWEYIPMDKAGQYVLDRARDLKKMFPKKPLLLSEVGWPSNGRMRG
GNETSPADQAIYLRTLVNKLNRQGFNYFVIEAFDQPWKVSDEGSAGAYWGVYNAARQQKFNFDGPVVAIPQWRVLAIGSV
LALLSLTLLMIDGSALRQRGRTFLTFIAFLCGSVLVWIGYDYSQQYSTWFSVTVGILLALGALGVFIVLLTEAHELAEAV
WTHKRRREFLPVVGDSDYRPKVSIHVPCYNEPPEMVKQTLDALAALDYPDYEVLIIDNNTKDPAVWEPVRDYCETLGPR
FKFFHVAPLAGFKGGALNYLIPHTAKDAEVIAVIDSDYCVSPNWLKHMVPHFADPKIAVVQSPQDYRDQNESTFKKLCYA
EYKGFFHIGMVTRNDRDAIIQHGTMTMTRRSVLEELGWADWCICEDAELGLRVFEKGLSAAYYHDSYGKGLMPDTFIDFK
KQRFRWAYGAIQIIKRHTASLLRGKDTELTRGQRYHFLAGWLPWVADGMNIFFTVGALLWSAAMIIVPTRVDPPLLIFAI
PPLALFVFKVGKIIFLYRRAVGVNLKDAFCAALAGLALSHTIAKAVLYGFFTTSIPFFRTPKNADNHGFWVAISEAREEM
FIMLLLWGAALGIYLVQGLPSNDIRFWVVMLLVQSLPYVAALVMAFLSSLPKPAPKVEPATVE

>Pseudomonas_lurida_MYb17_[Orphan] BLAST: A0A2R3HSF8 Beta-(1-3)-glucosyl
transferase
MASRKFGLNLVIVLAIAALFTGFWALINRPVTTPNWPEQISGFSYSPFQQGQYPQKDQYPTDDQMRRDLEIMSKLTDNIR
TYSVDGTLGDIPKLAEEFGLRVTLGIWISPDLERNEREIQRAIEIANSSRSVVRVVVGNEALFREEITPEALIVLLDRVR
AAVKVPVTTSEQWHIWEKNPKLANHVDLIAAHILPFWEYIPMDKAGQYVLDRARDLKKMFPKKPLLLSEVGWPSNGRMRG
GNETSPADQAIYLRTLVNKLNRQGFNYFVIEAFDQPWKVSDEGSAGAYWGVYNAARQQKFNFDGPVVAIPQWRVLAIGSV
VLALLSLTLLMIDGSALRQRGRTFLTFIAFLCGSVLVWIGYDYSQQYSTWFSVTVGILLALGALGVFIVLLTEAHELAEA
VWTHKRRREFLPVVGDSDYRPKVSIHVPCYNEPPEMVKQTLDALAALDYPDYEVLIIDNNTKDPAVWEPVRDYCETLGPR
FKFFHVAPLAGFKGGALNYLIPHTAKDAEVIAVIDSDYCVSPNWLKHMVPHFADPKIAVVQSPQDYRDQNESTFKKLCYA
EYKGFFHIGMVTRNDRDAIIQHGTMTMTRRSVLEELGWADWCICEDAELGLRVFEKGLSAAYYHDSYGKGLMPDTFIDFK
KQRFRWAYGAIQIIKRHTASLLRGKDTELTRGQRYHFLAGWLPWVADGMNIFFTVGALLWSAAMIIVPTRVDPPLLIFAI
PPLALFVFKVGKIIFLYRRAVGVNLKDAFCAALAGLALSHTIAKAVLYGFFTTSIPFFRTPKNADNHGFWVAISEAREEM
FIMLLLWGAALGIYLVQGLPSNDIRFWVVMLLVQSLPYVAALVMAFLSSLPKPAPKVEPPTVE

>Pseudomonas_lutea_DSM_17257_[Orphan] PseudoCAP: Beta-(1-3)-glucosyl transferase
MPSRKFGLNLVVVVAIAALFTGFWALINRPVTAPNWPEQISGFSYSPFRLGQNPQKDLYPSDEEMRQDLELMSKQTDNIR
IYSVDGTLKDIPKLAEEFGLRVTLGIWISPDLERNEREITTAIELANTTRSVVRVVVGNEALYREEIKPKDLMAALDRVR
AAVKVPVTTSEQWHIWEKYPELAKHVDLIAAHILPYWEYIPMDKAGQYVLDRARELKRMFPKKPLLLSEVGWPSNGHMRG
GADATQADQAVYLRTLVNKLNRQGFNYFVIEAFDQPWKASDEGSVGAYWGVYNAARQQKFNFEGPVVAIPQWRVLAIGSA
VLAMLALALLLIDGSALRQRGRTFLTFIAFLCGSVLVWIGYDYSQQYSTWFSLLVGFLLALGAFGVFIVLLTEAHELAEA
VWTHKRRREFLPVEGDSAYRPKVSIHVPCYNEPPEMVKQTLNALAALDYPDFEVLLIDNNTKDPAVWEPVKAHCEMLGPR

FKFFHVAPLAGFKGGALNYLIPHTAADAEVIAVIDSDYCVDRNWLKHMVPHFADPKIAVVQSPQDYRDQHESAFKKLCYS
EYKGFFYIGMVTRNDRDAIIQHGTMTMTRRSVLEELGWADWCICEDAELGLRVFEKGYSAAYAHHSFGKGLMPDTFIDFK
KQRFRWAYGAIQIIKRHAASLLRGKDTELTRGQRYHFLAGWLPWVADGMNIFFTVGALLWSSAMIIVPNRVDPPLLIFAI
PPLALFFFKVGKIIFLYRRAVGVNLTDAFYAALAGLALSHTIAKAVLYGFFTTSIPFFRTPKNADSHGLLVAISEAREEL
FIMLLLWGAALGICLVQGLPSNDMRFWVTMLLVQSLPYLAALIMAFLSSLPKPKSAEEPATA

>Pseudomonas_mandelii_JR-1_[Orphan] PseudoCAP: Beta-(1-3)-glucosyl transferase
MSSRKFGLNLVVVLAIAALFTGFWALINRPVTAPNWPEQISGFSYSPFQQGQYPQKEQYPTDDEMRRDLEIMSKLTDNIR
TYSVDGSLGDIPKLAEEFGLRVTLGIWISPDEERNEREILRAIELANTSRSVVRVVVGNEAIFRKEITAAELSIILDRVR
AAVKVPVTTSEQWHVWEENPSLAKHVDLIAAHVLPYWEHVPMEQSGQFVLDRARDLKKMFPKKPLLLSEVGWPSNGRMRG
GADASPADQAIYLRTLVNKLNRQGFNYFVIEAFDQPWKASDEGSVGAYWGVFNAARQQKFNFEGPVVAIPQWRVLAVGSV
VLALLSLTLLMIDGSALRQRGRTFLTFIAFLCGSVLVWIGYDYSLQYSTWFSLTIGFLLALGALGVFIVLLTEAHELAEA
VWTHKRRREFLPVVGESDYRPKVSIHVPCYNEPPEMVKQTLNALANLDYPDFEVLIIDNNTKDPAVWEPVRDYCATLGPR
FKFFHVAPLAGFKGGALNYLIPHTAKDAEVIAVIDSDYCVDPNWLKHMVPHFADPKIAVVQSPQDYRDQNESTFKKLCYA
EYKGFFHIGMVTRNDRDAIIQHGTMTMTRRSVLEELGWADWCICEDAELGLRVFEKGLSAAYYHTSYGKGLMPDTFIDFK
KQRFRWAYGAIQIIKRHTTSLLRGKGSELTRGQRYHFLAGWLPWVADGMNIFFTVGALLWSAAMIIVPQRVDPPLLIFAI
PPLALFVFKVGKIIFLYRRAVGVNLKDAFCAALAGLALSHTIAKAVLYGFFTSSIPFFRTPKNADNHGFWVAISEAREEV
FIMLLLWGAALGIYLVNGMPSNDMRFWVTMLLVQSLPYLAALIMAFLSSLPKPVAEAATAPAV

>Pseudomonas_marginalis_ICMP_3555_[Orphan] BLAST: A0A3M3W7M8 Glyco_trans_2-like domain-containing protein
MASRKFGLNLVIVLAIAALFSGFWALINRPVAAPNWPEQISGFSYSPFQQGQYPQKDQYPTDDQMRRDLEIMSKLTDNIR
TYSVDGTLGDIPKLAEEFGLRVTLGIWISPDLERNEREIQRAIEIANSSRSVVRVVVGNEALFREEITPEALIVLLDRVR
AAVKVPVTTSEQWHIWEKNPQLAKHVDLIAAHILPFWEYIPMDKAGQYVLDRARDLKKLFPKKPLLLSEVGWPSNGRMRG
GNETSPADQAIYLRTLVNKLNRQGFNYFVIEAFDQPWKVSDEGSAGAYWGVYNAARQQKFNFDGPVVAIPQWRVLAIGSV
VLALLSLTLLMIDGSSLRQRGRTFLTFIAFLCGSVLVWIGYDYSQQYSTWFSVTVGILLALGALGVFIVLLTEAHELAEA
VWTHKRRREFLPVEGDSDYRPKVSIHVPCYNEPPEMVKQTLDALAALDYPDYEVLIIDNNTKDPAVWEPVRDYCETLGPR
FKFFHVAPLAGFKGGALNYLIPHTAKDAEVIAVIDSDYCVSPNWLKHMVPHFADPKIAVVQSPQDYRDQNESTFKKLCYA
EYKGFFHIGMVTRNDRDAIIQHGTMTMTRRSVLEELGWADWCICEDAELGLRVFEKGLSAAYYHDSYGKGLMPDTFIDFK
KQRFRWAYGAIQIIKRHTASLLRGKGTELTRGQRYHFLAGWLPWVADGMNIFFTVGALLWSAAMIIVPTRVDPPLLIFAI
PPLALFVFKVGKIIFLYRRAVGVNLKDAFCAALAGLALSHTIAKAVLYGFFTTSIPFFRTPKNADNHGFWVAISEAREEM
FIMLLLWGAALGIYLVQGLPSNDIRFWVVMLLVQSLPYVAALVMAFLSSLPKPAPKVELATAE

>Pseudomonas_monteilii_SB3101_[Orphan] PseudoCAP: Beta-(1-3)-glucosyl transferase
MSSRKFGLNLVVVLAIAALFTGFWALINRPVSAPAWPEQISGFSYSPFRLGESPQKGQYPSDDEMRQDLEQLSKLTDSIR
IYTVEGTQADIPRLAEEFGLRVTLGIWISPDLERNEREIATAIQLANTSRSVVRVVVGNEALFREEVTPENLIKYLDRVR
AAVKVPVTTSEQWHIWKENPQLAKHVDLIAAHILPYWEFVPMKDSVEFVLDRARELKHQFPRKPLLLSEVGWPSNGRMRG
GADATQADQAIYLRTLVNTLNRRGYNYFVIEAYDQPWKASDEGSVGAYWGVYNAERQQKFNFDGPVVAIPQWRALAVASV
VLAMIALMVLFIDGSALRQRGRTFLTFITFLCGSVLVWIAYDYSQQYSTWFSLTVGVLLALGALGVFIVLLTEAHELAEA
VWIHKRRREFLPVQADSAYRPKVSVHVPCYNEPPEMVKQTLDALAALDYPDYEVLVIDNNTKDPAVWEPLKAHCEKLGER
FRFFHVAPLAGFKGGALNYLIPHTAKDAEVIAVIDSDYCVDRNWLKHMVPHFADPKIAVVQSPQDYRDQHESAFKKLCYS
EYKGFFHIGMVTRNDRDAIIQHGTMTMTRRSVLEELGWAEWCICEDAELGLRVFEKGLSAAYAHNSYGKGLMPDTFIDFK
KQRFRWAYGAIQIIKHHASALLRGKGSELTRGQRYHFLAGWLPWIADGMNIFFTIGALLWSAAMIIVPHRVDPPLMIFAI
PPLALFFFKVGKIIFLYRRAVGVNLKDAFAAALAGLALSHTIAKAVLYGFFTSSMPFFRTPKNADSHGLLVAISEAREEL
FIMLLLWGAALGIYLVQGLPSSDMRFWVAMLLVQSLPYVAALVMAFLSSLPKPGEKAAEAQQA

>Pseudomonas_moraviensis_R28-S_[Orphan] PseudoCAP: Beta-(1-3)-glucosyl transferase
MSSRKFGLNLVVVLAIAALFTGFWALVNRPVTAPNWPQQISGFSYSPFQQGQFPQKDQYPSDDEMRRDLEIMSKLTDNIR
IYSVDGSLGDIPKLAEEFGLRVTLGIWISPDQERNEREITRAIELANTSRSVVRVVVGNEALFREEITPEALIVLLDRVR
AAVKVPVTTSEQWHIWEKYPQLAKHVDLIAAHVLPYWEFIPVDKAGQFVFDRARDLKKLFPKKPLLLSEVGWPSNGRMRG
GADASPADQAIYLRTLVNKLNRQGFNYFVIEAFDQPWKASDEGSVGAYWGVFNAARQQKFNFEGPVVAIPQWRVLAIGSV
VLALLSLTLLMIDGSALRQRGRTFLTFIAFLCGSVLVYIGYDYSQQYSTWFSLTVGFLLALGALGVFIVLLTEAHELAEA
VWIHKRRREFLPVLGDSDYRPKVSIHVPCYNEPPEMVKQTLDALAALDYPDFEVLIIDNNTKDPAVWEPVRDYCATLGPR
FKFFHVSPLAGFKGGALNYLIPHTAKDAEVIAVIDSDYCVHPNWLKHMVPHFADPKIAVVQSPQDYRDQNESTFKKLCYA
EYKGFFHIGMVTRNDRDAIIQHGTMTMTRRSVLEELGWADWCICEDAELGLRVFEKGLSAAYYHDSYGKGLMPDTFIDFK
KQRFRWAYGAIQIIKRHTRSLLRGKDTELTRGQRYHFLAGWLPWVADGMNIFFTVGALLWSAAMIIVPQRVDPPLLIFAI
PPLALFVFKVGKIIFLYRRAVGVNLKDAFCAALAGLALSHTIAKAVLYGFFTSSIPFFRTPKNADNHGFWVAISEAREEL
FIMLLLWGAALGIFLVQGIPSNDMRFWVTMLLVQSLPYLAALIMAFLSSLPKPVAKAEPAPVV

>Pseudomonas_mosselii_DSM_17497_[Orphan] PseudoCAP: Beta-(1-3)-glucosyl transferase
MPSRKFGLNLVVVLAIAALFTGFWALINRPVSAPAWPEQISGFSYSPFRLGQSPQKGQYPSEDEIRQDLEQLNKLTDNIR
IYTVEGTQAEVPRLAEELGLRITLGIWISNDQERNEREIEKAIGLANTSRSVVRVVVGNEALFREEVSAEQLIGYLDRVR
AAVKVPVTTSEQWHIWKEHPELAKHVDLIAAHILPYWEFVPMKDSVQFVLDRARELRKQFPRKPLLLSEVGWPSNGRMRG
GADATQADQAIYLRTLVNTLNRRGYNYFVIEAYDQPWKASDEGSVGAYWGVYNAERQQKFNFEGPIVAIPQWRALAVASV
VLAMIALAILLIDGSALRQRGRTFLTFITFLCGSVLVWIAYDYSQQYSTWFSLTVGVLLALGALGVFIVLLTEAHELAEA
VWIHKRRREFLPVQGDSAYRPKVSVHVPCYNEPPEMVKQTLDALAALDYPDYEVLVIDNNTKDPAVWEPLKAHCEKLGER
FRFFHVAPLAGFKGGALNYLIPHTAKDAEVIAVIDSDYCVDRNWLKHMVPHFADPKIAVVQSPQDYRDQHESAFKKLCYS
EYKGFFHIGMVTRNDRDAIIQHGTMTMTRRSVLDELGWAEWCICEDAELGLRVFEKGLSAAYAHNSYGKGLMPDTFIDFK
KQRFRWAYGAIQIIKHHAGALLRGKGSELTRGQRYHFLAGWLPWIADGMNIFFTVGALLWSAAMIIVPHRVDPPLMMFAI
PPLALFFFKVGKIVFLYRRAVGVNLKDAFAAALAGLALSHTIAKAVLYGFFTSSMPFFRTPKNADSHGVLVAISEAREEL
FIMLLLWGAALGIYLVQGLPSSDMRFWVAMLLVQSLPYLAALVMALLSSLPKPSEKVAEPQQA

>Pseudomonas_nitroreducens_Aramco_J_[Orphan] PseudoCAP: Beta-(1-3)-glucosyl transferase
MSSRKFGLNLVVFVALAALFTGFWALYNRPVSVPDWPESISGFSFSPFRLNQNPQKDQFPSDDEIRSDLELVSRNTDNIR
TYSVKGSLADIPRLAEELGMRVSLGIWIGPDEAENEAEIERGIEIANNSRSVVRVIVGNEALFRREVTVEQLTAYLDRVR
KAVKVPVTTAEQWHIYEKYPEMAKHVDLIAAHVLPYWEYTPMDNAVPFVLERAKELRAKFPRKPLLLAEVGWPSNGRMRG
GADATQADQAIYLRTLTNALNKRGYNYFVVEAFDQPWKVGDEGSVGAYWGVYNAQRQPKFNFTGPVVNIPQWRALAVASV
VMALLALTLLMIDGSALRQRGRTFLTVVAFAGGSVLVWIAYDYSQQYSTWFSLTVGGLLGIGALGVFIVLLTEAHELAET
VWVRKRRRPFDPVLTDTGYRPKVSVHVPCYNEPPEMMKKTLDALSRLDYPDFEVLIIDNNTKDPAVWEPVRDYCEVLGPR
FRFFHVAPLAGFKGGALNYILPHTAPDVEVVAVIDADYCVEPNWLKQMVPHFSDPKIAVVQSPQDYHDGEENVFKKLCYA
EYKGFFHIGMVTRNDRDAIIQHGTMTMIRRTVMDELKWADWTICEDAELGLRVFEKGYSAAYSHQSFGKGVMPDTFIDYK
KQRFRWAYGAIQIMKGHARALFQGKDSKLTLGQRYHFIAGWLPWIADGMNIFFTVGALLWSSAMIIVPKRVDPPLLIFAI
PPLALFFFKFGKIMFLYRRAVGVNLLRSFQAAVAGLALSHTIAKAVLYGAFTKTIPFFRTPKMASNHGLLVALAEAREEV
FIMLLLWGAALGIVLVQGVPSRDMMFWVAMLLVQSLPYLAALVMALLSAAPKAQEAPAADVAPAN

>Pseudomonas_orientalis_CDVBN20_[Orphan] BLAST: A0A7V8ZXK8 Glycosyltransferase
MASRKFGLNLVIVLAIAALFTGFWALINRPVTAPNWPEQISGFSYSPFQQGQYPQKDQYPTDEQMRRDLEIMSKLTDNIR
TYSVDGTLGDIPKLAEEFGLRVTLGIWISPDLERNEREIQRAIEIANSSRSVVRVVVGNEALFREEITPEALIVLLDRVR
AAVKVPVTTSEQWHIWEKNPQLAKHVDLIAAHILPFWEFIPMDKAGQYVLDRARDLKKLFPKKPLLLSEVGWPSNGRMRG
GNETSPADQAVYLRTLVNKLNRQGFNYFVIEAFDQPWKVSDEGSAGAYWGVFNAARQQKFNFEGPVVAIPQWRVLAIGSV
VLALLSLTLLMIDGSALRQRGRTFLTFIAFLCGSVLVWIGYDYSQQYSTWFSVTVGILLALGALGVFIVLLTEAHELAEA
VWTHKRRREFLPVEGESDYRPKVSIHVPCYNEPPEMVKQTLDALAALDYPDYEVLIIDNNTKDPAVWEPVRDYCATLGPR
FKFFHVAPLAGFKGGALNYLIPHTAKDAEVIAVIDSDYCVSPNWLKHMVPHFADPKIAVVQSPQDYRDQNESTFKKLCYA
EYKGFFHIGMVTRNDRDAIIQHGTMTMTRRSVLEELGWADWCICEDAELGLRVFEKGLSAAYYHDSYGKGLMPDTFIDFK
KQRFRWAYGAIQIIKRHTASLLRGKGTELTRGQRYHFLAGWLPWVADGMNIFFTVGALLWSAAMIIVPTRVDPPLLIFAI
PPLALFVFKVGKIIFLYRRAVGVNLKDAFCAALAGLALSHTIAKAVLYGFFTTSIPFFRTPKNADNHGFWVAISEAREEM
FIMLLLWGAALGIYLVQGLPSNDMRFWVVMLLVQSLPYVAALIMAFLSSLPKPAPKAEPVTAV

>Pseudomonas_orientalis_DSM_17489_[Orphan] BLAST: A0A0R3A0H2 Beta-(1-3)-glucosyl transferase
MASRKFGLNLVVVLAIAALFTGFWALINRPVTAPNWPEQISGFSYSPFQQGQYPQKDQYPTDEQMRQDLAIMSKLTDNIR
TYSVDGTLGDIPKLAEEFGLRVTLGIWISPDLERNEREIQRAIEIANSSRSVVRVVVGNEALFREEITPEALIVLLDRVR
AAVKVPVTTSEQWHIWEKNPQLAKHVDLIAAHILPFWEFIPMDKAGQYVLDRARDLKKLFPKKPLLLSEVGWPSNGRMRG
GNETSPADQAVYLRTLVNKLNRQGFNYFVIEAFDQPWKVSDEGSAGAYWGVFNAARQQKFNFEGPVVAIPQWRVLAIGSV
VLALLSLTLLMIDGSALRQRGRTFLTFIAFLCGSVLVWIGYDYSQQYSTWFSVTVGILLALGALGVFIVLLTEAHELAEA
VWTHKRRREFLPVEGESDYRPKVSIHVPCYNEPPDMVKQTLDALAALDYPDYEVLIIDNNTKDPAVWEPVRDYCETLGPR
FKFFHVAPLAGFKGGALNYLIPHTAKDAEVIAVIDSDYCVSPNWLKHMVPHFADPKIAVVQSPQDYRDQNESTFKKLCYA
EYKGFFHIGMVTRNDRDAIIQHGTMTMTRRSVLEELGWADWCICEDAELGLRVFEKGLSAAYYHDSYGKGLMPDTFIDFK
KQRFRWAYGAIQIIKRHTASLLRGKGTELTRGQRYHFLAGWLPWVADGMNIFFTVGALLWSAAMIIVPTRVDPPLLIFAI
PPLALFVFKVGKIIFLYRRAVGVNLKDAFCAALAGLALSHTIAKAVLYGFFTTSIPFFRTPKNADNHGFWVAISEAREEM
FIMLLLWGAALGIYLVQGLPSNDMRFWVVMLLVQSLPYVAALIMAFLSSLPKPAPKAEPVTAV

>Pseudomonas_orientalis_F9_[Orphan] BLAST: A0A2L0RSS1 Beta-(1-3)-glucosyl transferase
MASRKFGLNLVIVLAIAALFTGFWALINRPVTAPNWPEQISGFSYSPFQQGQYPQKDQYPTDEQMRRDLEIMSKLTDNIR
TYSVDGTLGDIPKLAEEFGLRVTLGIWISPDLERNEREIQRAIEIANNSRSVVRVVVGNEALFREEITPEALIVLLDRVR
AAVKVPVTTSEQWHIWEKNPQLARHVDLIAAHILPFWEFIPMDKAGQYVLDRARDLKKLFPKKPLLLSEVGWPSNGRMRG
GNETSPADQAVYLRTLVNKLNRQGFNYFVIEAFDQPWKVSDEGSAGAYWGVFNAARQQKFNFEGPVVAIPQWRVLAIGSV

VLALLSLTLLMIDGSALRQRGRTFLTFIAFLCGSVLVWIGYDYSQQYSTWFSVTVGILLALGALGVFIVLLTEAHELAEA
VWTHKRRREFLPVEGESDYRPKVSIHVPCYNEPPEMVKQTLDALAALDYPDYEVLIIDNNTKDPAVWEPVRDYCETLGPR
FKFFHVAPLAGFKGGALNYLIPHTAKDAEVIAVIDSDYCVSPNWLKHMVPHFADPKIAVVQSPQDYRDQNESTFKKLCYA
EYKGFFHIGMVTRNDRDAIIQHGTMTMTRRSVLEELGWADWCICEDAELGLRVFEKGLSAAYYHDSYGKGLMPDTFIDFK
KQRFRWAYGAIQIIKRHTASLLRGKGTELTRGQRYHFLAGWLPWVADGMNIFFTVGALLWSAAMIIVPTRVDPPLLIFAI
PPLALFVFKVGKIIFLYRRAVGVNLKDAFCAALAGLALSHTIAKAVLYGFFTTSIPFFRTPKNADNHGFWVAISEAREEM
FIMLLLWGAALGIYLVQGLPSNDMRFWVVMLLVQSLPYVAALIMAFLSSLPKPAPKAEPVTAV

>Pseudomonas_orientalis_133NRW_[Orphan] BLAST: A0A4Q7CY06 Glycosyltransferase
MASRKFGLNLVVVLAIAALFTGFWALINRPVTAPNWPEQISGFSYSPFQQGQYPQKDQYPTDEQMRQDLAIMSKLTDNIR
TYSVDGTLGDIPKLAEEFGLRVTLGIWISPDLERNEREIQRAIEIANTSRSVVRVVVGNEALFREEITPEALIVLLDRVR
AAVKVPVTTSEQWHIWEKNPQLAKHVDLIAAHILPFWEFIPMDKAGQYVLDRARDLKKLFPKKPLLLSEVGWPSNGRMRG
GNETSPADQAVYLRTLVNKLNRQGFNYFVIEAFDQPWKVSDEGSAGAYWGVFNAARQQKFNFEGPVVAIPQWRVLAIGSV
VLALLSLTLLMIDGSALRQRGRTFLTFIAFLCGSVLVWIGYDYSQQYSTWFSVTVGILLALGALGVFIVLLTEAHELAEA
VWTHKRRREFLPVEGESDYRPKVSIHVPCYNEPPEMVKQTLDALAALDYPDYEVLIIDNNTKDPAVWEPVRDYCQTLGPR
FKFFHVAPLAGFKGGALNYLIPHTAKDAEVIAVIDSDYCVSPNWLKHMVPHFADPKIAVVQSPQDYRDQNESTFKKLCYA
EYKGFFHIGMVTRNDRDAIIQHGTMTMTRRSVLEELGWADWCICEDAELGLRVFEKGLSAAYYHDSYGKGLMPDTFIDFK
KQRFRWAYGAIQIIKRHTASLLRGKGTELTRGQRYHFLAGWLPWVADGMNIFFTVGALLWSAAMIIVPTRVDPPLLIFAI
PPLALFVFKVGKIIFLYRRAVGVNLKDAFCAALAGLALSHTIAKAVLYGFFTTSIPFFRTPKNADNHGFWVAISEAREEM
FIMLLLWGAALGIYLVQGLPSNDMRFWVVMLLVQSLPYVAALIMAFLSSLPKPAPKAEPVTAV

>Pseudomonas_otitidis_LNU-E-001_[Orphan] PseudoCAP: Glucosyl transferase
MSSRKIGLNLVVIVALAALFTGIWALYNRPVSVPDWPERISGFSFSPFRLNQNPQSGRYPSAEQMRTDLELVARHTHSIR
TYSVQGALGDIPALAEAFGLRVSLGIWLGPDLASNEAEIARAIRIANESPSVVRVIVGNEALFRREVTAEQLIAYLDRVR
AAVKVPVTTAEQWHVYREHPELAQHVDLIAAHVLPYWEATPVADAVDFVLERARELKAAFPRKPLLLAEVGWPSNGRMRG
SAEATPADQAIYLRRLTNALNGEGYSYFVIEAFDQPWKVSAEGSVGAYWGVYNADRKAKFNFTGPVVPIPKWRALAIASA
VLAVLAFTLLLIDSSSLRQRGRTFLAVVSFACASVLVWIAYDYSQQYSTWFSLTVGALLGVGALGVVIVLFTEAHELAEA
VWTRKRRRPFLPITAAQAYRPKVSIHVPCYNEPPELLKQTLDALARLDYPDYEVLVIDNNTRDPAVWQPVEAHCARLGER
FRFFHVAPLEGFKAGALNFALGHVAADVEVVAVIDADYCVDPDWLRHMVPHFGDPRIAVVQSPQDYRDQHESAFKRLCYA
EYKGFFHIGMVTRNDRDAIIEHGTMTMIRRSVLDELRWPEWCITEDAELGLRVFEKGLSAAYFERSYGKGVMPDTFIDFK
KQRFRWAYGAIQIMKRHTDALLRGRGPDGSRLTRGQRYHFVAGWLPWIADGLNIFFTLGALLWSAAMIIVPKRVDPPLLI
FAILPLALFVFKVGKILFLYRRTVGVDLRDSFFAALAGLSLSHTIAKAVLYGFVTRGIPFFRTPKMRSSHGLLVALAEAR
EEVFVMLLLWGAAAGIVAVQGVPSRDLLIWVAMLLVQSLPYLAALVMALLSSLPKPREELAGGAERIGG

>Pseudomonas_panacis_PgKB35_[Orphan] BLAST: A0A5M9I977 Cellulose synthase 1 (BcsA) but changed in this work
MASRKFGLNLVIVLAIAALFTGFWALINRPVTAPNWPEQISGFSYSPFQQGQYPQKDQYPTDEQMRRDLEIMSKLTDNIR
TYSVDGTLGDIPKLAEEFGLRVTLGIWISPDLERNEREVQRAIEIANSSRSVVRVVVGNEALFREEITPEALIVLLDRVR
AAVKVPVTTSEQWHIWEKNPQLAKHVDLIAAHILPFWEYIPMDKAGQYVLDRARDLKKLFPKKPLLLSEVGWPSNGRMRG
GNETSPADQAIYLRTLVNKLNRQGYNYFVIEAFDQPWKVSDEGSAGAYWGVYNAARQQKFNFEGPVVAIPQWRVLAIGSV
VLALLSLTLLMIDGSSLRQRGRTFLTFIAFLCGSVLVWIGYDYSQQYSTWFSVTVGILLALGALGVFIVLLTEAHELAEA
VWTHKRRREFLPVEGESDYRPKVSIHVPCYNEPPEMVKQTLDALAALDYPDYEVLIIDNNTKDPAVWEPVRDYCETLGPR
FKFFHVAPLAGFKGGALNYLIPHTAKDAEVIAVIDSDYCVSPNWLKHMVPHFADPKIAVVQSPQDYRDQNESTFKKLCYA
EYKGFFHIGMVTRNDRDAIIQHGTMTMTRRSVLEELGWADWCICEDAELGLRVFEKGLSAAYYHDSYGKGLMPDTFIDFK
KQRFRWAYGAIQIIKRHTASLLRGKDTELTRGQRYHFLAGWLPWVADGMNIFFTVGALLWSAAMIIVPTRVDPPLLIFAI
PPLALFVFKVGKIIFLYRRAVGVNLKDAFCAALAGLALSHTIAKAVLYGFFTTSIPFFRTPKNADNHGFWVAISEAREEM
FIMLLLWGAALGIYLVQGLPSNDIRFWVVMLLVQSLPYVAALIMAFLSSLPKPAVAPEPAPVA

>Pseudomonas_panacis_WS_4668_[Orphan] BLAST: A0A7Y1NL58 Glycosyltransferase
MASRKFGLNLVIVLAIAALFTGFWALINRPVTAPNWPEQISGFSYSPFQQGQYPQKDQYPTDEQIRRDLEIMSKLTDNIR
TYSVDGTLGDIPKLAEEFGLRVTLGIWISPDLERNEREVQRAIEIANSSRSVVRVVVGNEALFREEITPEALIVLLDRVR
AAVKVPVTTSEQWHIWEKNPQLAKHVDLIAAHILPFWEYIPMDKAGQYVLDRARDLKKLFPKKPLLLSEVGWPSNGRMRG
GNETSPADQAIYLRTLVNKLNRQGYNYFVIEAFDQPWKVSDEGSAGAYWGVYNAARQQKFNFEGPVVAIPQWRVLAIGSV
VLALLSLTLLMIDGSSLRQRGRTFLTFIAFLCGSVLVWIGYDYSQQYSTWFSVTVGILLALGALGVFIVLLTEAHELAEA
VWTHKRRREFLPVEGESDYRPKVSIHVPCYNEPPEMVKQTLDALAALDYPDYEVLIIDNNTKDPAVWEPVRDYCETLGPR
FKFFHVAPLAGFKGGALNYLIPHTAKDAEVIAVIDSDYCVSPNWLKHMVPHFADPKIAVVQSPQDYRDQNESTFKKLCYA
EYKGFFHIGMVTRNDRDAIIQHGTMTMTRRSVLEELGWADWCICEDAELGLRVFEKGLSAAYYHDSYGKGLMPDTFIDFK
KQRFRWAYGAIQIIKRHTASLLRGKDTELTRGQRYHFLAGWLPWVADGMNIFFTVGALLWSAAMIIVPTRVDPPLLIFAI
PPLALFVFKVGKIIFLYRRAVGVNLKDAFCAALAGLALSHTIAKAVLYGFFTTSIPFFRTPKNADNHGFWVAISEAREEM
FIMLLLWGAALGIYLVQGLPSNDIRFWVVMLLVQSLPYVAALIMAFLSSLPKPAVAPEPAPAA

>Pseudomonas_parafulva_YAB-1_[Orphan] PseudoCAP: Beta-(1-3)-glucosyl transferase
MSSRKFGLNLVVVLAIAALFTGFWALINRPVSAPAWPEQISGFSYSPFRLGESPQKGQYPDDNEMRQDLEQMSKLTDSIR
IYTVEGTQAHIPKLAEEFGLRVTVGIWISPDLERNEREIATAIELANTSRSVVRVVVGNEALFREEVTPENLIQYLDRVR
AAVKVPVTTSEQWHIWKQNPQLAKHVDLIAAHILPYWEFVPMKDSVEFVLDRARELKHQFPRKPLLLSEVGWPSNGRMRG
GADATQADQAIYLRTLVNTLNRRGFNYFVIEAYDQPWKASDEGSVGAYWGVFNAERQQKFNFEGPVVAIPQWRALAVASV
VLAMIALMVLFIDGSALRQRGRTFLTFITFLCGSVLVWIAYDYSQQYSTWFSLTVGVLLALGALGVFIVLLTEAHELAEA
VWTHKRRREFLPVHGDSAYRPKVSVHVPCYNEPPEMVKQTLDALAALDYPDYEVLVIDNNTKDPAVWEPLKAHCEKLGER
FRFFHVAPLAGFKGGALNYLLPHTAKDAEVIAVIDSDYCVDRNWLKHMVPHFADPKIAVVQSPQDYRDQHESAFKKLCYS
EYKGFFHIGMVTRNDRDAIIQHGTMTMTRRSVLDELGWAEWCICEDAELGLRVFEKGLSAAYAHNSYGKGLMPDTFIDFK
KQRFRWAYGAIQIIKHHAAALLRGKGSELTRGQRYHFLAGWLPWIADGMNIFFTVGALLWSAAMIIVPHRVDPPLMIFAI
PPLALFFFKVGKIVFLYRRAVGVNLKDALAAALAGLALSHTIAKAVLYGFFTSSMPFFRTPKNADSHGLLVAISEAREEL
FIMLLLWGAAAGIYLVQGLPSSDMRFWVAMLLVQSLPYLAALVMAFLSSLPKPEAKATEKAVETP

>Pseudomonas_plecoglossicida_NyZ12_[Orphan] PseudoCAP: Beta-(1-3)-glucosyl transferase
MSSRKFGLNLVVVLAIAALFTGFWALINRPVSAPAWPEQISGFSYSPFRLGESPQKGQYPSDDEMRQDLEQLSKLTDSIR
IYTVEGTQADIPRLAEEFGLRVTLGIWISPDLERNEREIATAIQLANTSRSVVRVVVGNEALFREEVTPENLIKYLDRVR
AAVKVPVTTSEQWHIWKENPQLAKHVDLIAAHILPYWEFVPMKDSVEFVLDRARELKHQFPRKPLLLSEVGWPSNGRMRG
GADATQADQAIYLRTLVNTLNRRGYNYFVIEAYDQPWKASDEGSVGAYWGVYNAERQQKFNFDGPVVAIPQWRALAVASV
VLAMIALMVLFIDGSALRQRGRTFLTFITFLCGSVLVWIAYDYSQQYSTWFSLTVGVLLALGALGVFIVLLTEAHELAEA
VWIHKRRREFLPVQADSAYRPKVSVHVPCYNEPPEMVKQTLDALAALDYPDYEVLVIDNNTKDPAVWEPLKAHCEKLGER
FRFFHVAPLAGFKGGALNYLIPHTAKDAEVIAVIDSDYCVDRNWLKHMVPHFADPKIAVVQSPQDYRDQHESAFKKLCYS
EYKGFFHIGMVTRNDRDAIIQHGTMTMTRRSVLEELGWAEWCICEDAELGLRVFEKGLSAAYAHNSYGKGLMPDTFIDFK
KQRFRWAYGAIQIIKHHASALLRGKGSELTRGQRYHFLAGWLPWIADGMNIFFTIGALLWSAAMIIVPHRVDPPLMIFAI
PPLALFFFKVGKIIFLYRRAVGVNLKDAFAAALAGLALSHTIAKAVLYGFFTSSMPFFRTPKNADSHGLLVAISEAREEL
FIMLLLWGAALGIYLVQGLPSSDMRFWVAMLLVQSLPYVAALVMAFLSSLPKPGEKAAEAQQA

>Pseudomonas_poae_RE_1-1-14_[Orphan] PseudoCAP: Putative beta-(1-3)-glucosyl transferase
MSSRKFGLNLVVVLAIAALFTGFWALINRPVTTPNWPEQISGFSYSPFQQGQYPQKNQYPSDDQMRRDLEIMSKLTDNIR
TYSVDGTLGDIPKLAEEFGLRVTLGIWISPDLERNEREVQRAIEIANSSRSVVRVVVGNEALFREEITPEALIVLLDRVR
AAVKVPVTTSEQWHIWEKNPQLAKHVDLIAAHILPFWEFIPMDKAGQYVLDRARDLKKLFPKKPLLLSEVGWPSNGRMRG
GNESSPADQAIYLRTLVNKLNRQGFNYFVIEAFDQPWKVSDEGSAGAYWGVFNAARQQKFNFEGPVVAIPQWRVLAIGSV
VLALLSLTLLMIDGSALRQRGRTFLTFIAFLCGSVLVWIGYDYSQQYSTWFSLTVGFLLALGAMGVFIVLLTEAHELAEA
VWTHKRRREFLPVEGDSDYRPKVSIHVPCYNEPPEMVKQTLDALAALDYPDYEVLIIDNNTKDPAVWEPVRDYCETLGPR
FKFFHVSPLAGFKGGALNYLIPHTAADAEVIAVIDSDYCVSPNWLKHMVPHFADPKIAVVQSPQDYRDQNESTFKKLCYA
EYKGFFHIGMVTRNDRDAIIQHGTMTMTRRSVLEELGWADWCICEDAELGLRVFEKGLSAAYYHDSYGKGLMPDTFIDFK
KQRFRWAYGAIQIIKRHTASLLRGKDTELTRGQRYHFLAGWLPWVADGMNIFFTVGALLWSAAMIIVPARVDPPLLIFAI
PPLALFVFKVGKIIFLYRRAVGVNLKDAFCAALAGLALSHTIAKAVLYGFFTTSIPFFRTPKNADNHGFWVAISEAREEM
FIMLLLWGAALGIYLVQGLPSNDMRFWVVMLLVQSLPYVAALIMAFLSSLPKPAAAPEPAPAA

>Pseudomonas_protegens_CHA0_[Orphan] PseudoCAP: glycosyl transferase, group 2 family protein
MSSRKFGLNLVIVLAIAALFTGFWALINRPVSAPDWPEQISGFSYSPFQQGQFPQKDQYPSDEMRRDLEIMSKLTDNIRT
YSVDGSLEDIPRLAEEFGLRVTLGIWISPDLERNEREIMRAIELANTSRSVVRVVVGNEAIFRKEITADQLSVILDRVRA
AVKVPVTTSEQWHVWEENPGLAKHVDLIAAHILPYWEFIPVDKAEQFVLDRARDLKKMFPKKPLLLSEVGWPSNGRMRGG
ADASPADQAIYLRTLVNKLNRQGYNYFVIEAFDQPWKASDEGSVGAYWGVFNAARQQKFNFEGPVVAIPQWRVLAIGSVV
LALLSLTLLMIDGSALRQRGRTFLTFIAFLCGSVLVWIGYDYSQQYSTWFSLTVGFLLALGALGVFIVLLTEAHELAEAV
WIHKRRREFLPVEGDSSYRPKVSIHVPCYNEPPEMVKQTLNALANLDYPDFEVLIIDNNTKDPAVWEPVQAYCETLGPRF
KFFHVAPLAGFKGGALNYLLPHTAKDAEVIAVIDSDYCVDRNWLKHMVPHFADPKIAIVQSPQDYRDQNESTFKKLCYAE
YKGFFHIGMVTRNDRDAIIQHGTMTMTRRTVLEELGWADWCICEDAELGLRVFEKGLSAAYHHESYGKGLMPDTFIDFKK
QRFRWAYGAIQIIKRHTASLLRGKNTELTRGQRYHFLAGWLPWVADGMNIFFTVGALLWSAAMIIVPQRVDPPLLIFAIP
PLALFVFKVGKIIFLYRRAVGVNLKDAFCAALAGLALSHTIAKAVLYGFFTSSIPFFRTPKNADNHGFWVAISEAREELF
IMLLLWGAALGIFLVQGLPSNDMRFWVTMLLVQSLPYVAALVMAFLSSLPKPAEESQPAPAT

>Pseudomonas_putida_KT2440_[BcsA] PseudoCAP: Cellulose synthase and translocator subunit
MTLNPLSAYTWFTVRGARLPVAWLFTLGVWLAFLFLRLESPAWQALLAERQRLYPQLAGKRPTLGDPLRLLIQSLWLLLR
RQPQVRTARPGRRALGAVRMHLRAAGGVARHYRGLLIDALQQVPARYRASAFKHQASARLRGLSVFARRAFYSVLTVFAL
SLALLCVTEPFGYLAQLMFICLLLVIALLVRHMPGRFPTLMLIVLSTIISCRYLWWRYTSTLNWNDTTDLVFGVILLAAE
TYSWFVLILGYIQTSWPLQRKPANLPANTRHWPTVDLLIPTYNEDLSVVRTTVMAALGLDWPRECLRIYILDDGRRDAFR

AFADEVGVGYIVRPDSKHAKAGNLNHALGVTDSELIAIFDCDHVPVRSFLQLTVGWFLKDAKLALVQTPHHFFSPDPFER
NLGSFRRRPNEGELFYGLIQDGNDMWNAAFFCGSCAVLRRTALESIGGFAVETVTEDAHTALRLHRQGWTSAYLSIPQAA
GLATESLSAHIGQRIRWARGMVQIFRTDNPLFGRGLSLFQRVCYANAMLHFLAGLPRLVFLTAPLAFLLLHAYIIYAPAL
MILLYVLPHMIHASLTNSRMQGRYRQTFWGEVYETVLAWYIARPTTVALFAPKKGTFNVTAKGGLMEQEQFDWRIAQPYL
WLAALNVVGLGFAVWRLVTGPTAEIGTVIVSSLWVIYNLLIIGAAVAVAAEVRQVRRAHRVQMRLPAGLMLASGHAYPCT
LVDYSDGGIGLQVQPGLELKPGEQVRLLLNRGQREFAFQACVTRTVGQHVGLVFRDLALQQRIDLVHCTFARADAWLGWN
EQHEVERPLRSLIDVLKLGGVGYVRLVEHMPPWLRAWLRPLRSLASWLASYWPRTPQAIPSMNPVDRDA

>Pseudomonas_putida_KT2440_[Orphan] PseudoCAP: Beta-(1-3)-glucosyl transferase
MSSRKFGLNLVVVLAIAALFTGFWALINRPVSAPAWPEQISGFSYSPFRLGESPQKGQYPTDDEMRQDLEQLSKLTDSIR
IYTVEGTQADVPRLAEEFGLRVTLGIWISPDLERNEREIATAIQLANTSRSVVRVVVGNEALFREEVTPENLIKYLDRVR
AAVKVPVTTSEQWHIWKEHPELARHVDLIAAHILPYWEFVPMKDSVEFVLERARELKHQFPRKPLLLSEVGWPSNGRMRG
GADATQADQAIYLRTLVNTLNRRGYNYFVIEAYDQPWKASDEGSVGAYWGVYNAERQQKFNFDGPVVAIPQWRALAVASV
VLAMIALMVLFIDGSALRQRGRTFLTFITFLCGSVLVWIAYDYSQQYSTWFSLTVGVLLALGALGVFIVLLTEAHELAEA
VWIHKRRREFLPVQADTAYRPKVSVHVPCYNEPPEMVKQTLDALAALDYPDYEVLVIDNNTKDPAVWEPLKAHCEKLGER
FKFFHVAPLAGFKGGALNYLIPHTAKDAEVIAVIDSDYCVDRNWLKHMVPHFADPKIAVVQSPQDYRDQHESAFKKLCYS
EYKGFFHIGMVTRNDRDAIIQHGTMTMTRRSVLEELGWAEWCICEDAELGLRVFEKGLSAAYAHNSYGKGLMPDTFIDFK
KQRFRWAYGAIQIIKHHAGALLRGKGSQLTRGQRYHFLAGWLPWIADGMNIFFTIGALLWSAAMIIVPHRVDPPLMIFAI
PPLALFFFKVGKIIFLYRRAVGVNLKDAFAAALAGLALSHTIAKAVLYGFFTSSMPFFRTPKNADSHGLLVAISEAREEL
FIMVLLWGAALGIYLVQGLPSSDMRFWVAMLLVQSLPYVAALVMAFLSSLPKPAEKAAQAQQA

>Pseudomonas_putida_S610_[Orphan] PseudoCAP: Glycosyl transferase 2 family protein
MSSRKFGLNLVVVLAIAALFTGFWALINRPVSAPAWPEQISGFSYSPFRLGESPQKGQYPDDNEMRQDLEQMSKLTDSIR
IYTVEGTQAHIPKLAEEFGLRVTVGIWISPDLERNEREIATAIELANTSRSVVRVVVGNEALFREEVTPENLIQYLDRVR
AAVKVPVTTSEQWHIWKQNPQLAKHVDLIAAHILPYWEFVPMKDSVEFVLDRARELKHQFPRKPLLLSEVGWPSNGRMRG
GADATQADQAIYLRTLVNTLNRRGFNYFVIEAYDQPWKASDEGSVGAYWGVFNAERQQKFNFEGPVVAIPQWRTLAVASV
VLAMIALMVLFIDGSALRQRGRTFLTFITFLCGSVLVWIAYDYSQQYSTWFSLTVGVLLALGALGVFIVLLTEAHELAEA
VWTHKRRREFLPVHGDSAYRPKVSVHVPCYNEPPEMVKQTLDALAALDYPDYEVLVIDNNTKDPAVWEPLKAHCEKLGER
FRFFHVAPLAGFKGGALNYLLPHTAKDAEVIAVIDSDYCVDRNWLKHMVPHFADPKIAVVQSPQDYRDQHESAFKKLCYS
EYKGFFHIGMVTRNDRDAIIQHGTMTMTRRSVLDELGWAEWCICEDAELGLRVFEKGLSAAYAHNSYGKGLMPDTFIDFK
KQRFRWAYGAIQIIKHHAAALLRGKGSELTRGQRYHFLAGWLPWIADGMNIFFTVGALLWSAAMIIVPHRVDPPLMIFAI
PPLALFFFKVGKIVFLYRRAVGVNLKDALAAAVAGLALSHTIAKAVLYGFFTSSMPFFRTPKNADSHGLLVAISEAREEL
FIMLLLWGAAAGIYLVQGLPSSDMRFWVAMLLVQSLPYLAALVMAFLSSLPKPEEQATQKAVETP

>Pseudomonas_putida_W619_[Orphan] PseudoCAP: Glycosyl transferase family protein
MSSRKFGLNLVIVLAIAALFTGFWALVNRPVSAPAWPEQISGFSYSPFRLGESPQKGQYPSDAEMRQDLEQMNKLTDSIR
IYTVEGTQADIPRLAEELGLRVTLGIWISPDLERNEREIATAIELANTSRSVVRVVVGNEALFREEVTPEALIQYLDRVR
AAVKVPVTTSEQWHIWKEHPELAKHVDLIAAHILPYWEFVPMKDSVEFVLDRARELKQQFPRKPLLLSEVGWPSNGRMRG
GADATQADQAIYLRTLVNTLNRRGYNYFVIEAYDQPWKASDEGSVGAYWGVFNAERQQKFNFEGPVVAIPQWRALAVASV
VLAMIALTVLLIDGSALRQRGRTFLTFITFLCGSVLVWIGYDYSQQYSTWFSLTVGVLLALGALGVFIVLLTEAHELAEA
AWTHKRRREFLPVQADSAYRPKVSVHVPCYNEPPEMVKQTLDALAALDYPDYEVLVIDNNTKDPAVWEPLKAHCEKLGER
FKFFHVAPLAGFKGGALNYLLPHTAKDAEVIAVIDSDYCVDRNWLKHMVPHFADPKIAVVQSPQDYRDQHESAFKKLCYS
EYKGFFHIGMVTRNDRDAIIQHGTMTMTRRTVLEELGWAEWCICEDAELGLRVFEKGLSAAYAHNSYGKGLMPDTFIDFK
KQRFRWAYGAIQIIKHHAAALLRGKGSELTRGQRYHFLAGWLPWVADGMNIFFTVGALLWSAAMIIVPHRVDPPLMIFAI
PPLALFFFKVAKIIFLYRRAVGVNLKDAFAAALAGLALSHTIAKAVLYGFFTSSMPFFRTPKNADSHGLLVAISEAREEL
FIMLLLWGAAAGIYLVQGLPSSDMRFWVAMLLVQSLPYLAALVMALLSSLPKPEHKMAEPQEA

>Pseudomonas_putida_YKD221_[Orphan] PseudoCAP: Beta-(1-3)-glucosyl transferase
MSSRKFGLNLVVVLAIAALFTGFWALINRPVSAPAWPEQISGFSYSPFRLGESPQKGQYPTDDEMRQDLEQLSKLTDSIR
IYTVEGTQADVPRLAEEFGLRVTLGIWISPDLERNEREIATAIQLANTSRSVVRVVVGNEALFREEVTPENLIKYLDRVR
AAVKVPVTTSEQWHIWKEHPELARHVDLIAAHILPYWEFVPMKDSVEFVLDRARELKHQFPRKPLLLSEVGWPSNGRMRG
GADATQADQAIYLRTLVNTLNRRGYNYFVIEAYDQPWKASDEGSVGAYWGVYNAERQQKFNFDGPVVAIPQWRALAVASV
VLAMIALMVLFIDGSALRQRGRTFLTFITFLCGSVLVWIAYDYSQQYSTWFSLTVGVLLALGALGVFIVLLTEAHELAEA
VWIHKRRREFLPVQADSAYRPKVSVHVPCYNEPPEMVKQTLDALAALDYPDYEVLVIDNNTKDPAVWEPLKAHCEKLGER
FKFFHVAPLAGFKGGALNYLIPHTAKDAEVIAVIDSDYCVDRNWLKHMVPHFADPKIAVVQSPQDYRDQHESAFKKLCYS
EYKGFFHIGMVTRNDRDAIIQHGTMTMTRRSVLEELGWAEWCICEDAELGLRVFEKGLSAAYAHNSYGKGLMPDTFIDFK
KQRFRWAYGAIQIIKHHAGALLRGKGSQLTRGQRYHFLAGWLPWIADGMNIFFTIGALLWSAAMIIVPHRVDPPLMIFAI
PPLALFFFKVGKIIFLYRRAVGVNLKDAFAAALAGLALSHTIAKAVLYGFFTSSMPFFRTPKNADSHGLLVAISEAREEL
FIMLLLWGAALGIYLVQGLPSSDMRFWVAMLLVQSLPYVAALVMAFLSSLPKPAEKAAQAQQA

>Pseudomonas_reactans_IPO375_[Orphan] BLAST: A0A7Y7ZPL8 Glycosyltransferase
MASRKFGLNLVIVLAIAALFTGFWALINRPVTTPNWPEQISGFSYSPFQQGQYPQKDQYPTDDQMRRDLEIMSKLTDNIR
IYSVDGTLGDIPKLAEEFGLRVTLGIWISPDLERNEREIQRAIEIANSSRSVVRVVVGNEALFREEITPEALIVLLDRVR
AAVKVPVTTSEQWHIWEKNPQLAKHVDLIAAHILPFWEYIPMDKAGQYVLDRARDLKKLFPKKPLLLSEVGWPSNGRMRG
GNETSPADQAIYLRTLVNKLNRQGFNYFVIEAFDQPWKVSDEGSAGAYWGVYNAARQQKFNFEGPVVAIPQWRVLAIGSV
VLALLSLTLLMIDGSALRQRGRTFLTFIAFLCGSVLVWIYDYSQQYSTWFSVTVGILLALGALGVFIVLLTEAHELAEAV
WTHKRRREFLPVEGDSDYRPKVSIHVPCYNEPPEMVKQTLDALAALDYPDYEVLIIDNNTKDPAVWEPVRDYCETLGPRF
KFFHVAPLAGFKGGALNYLIPHTAKDAEVIAVIDSDYCVSPNWLKHMVPHFADPKIAVVQSPQDYRDQNESTFKKLCYAE
YKGFFHIGMVTRNDRDAIIQHGTMTMTRRSVLEELGWADWCICEDAELGLRVFEKGLSAAYYHDSYGKGLMPDTFIDFKK
QRFRWAYGAIQIIKRHTASLLRGKDTELTRGQRYHFLAGWLPWVADGMNIFFTVGALLWSAAMIIVPTRVDPPLLIFAIP
PLALFVFKVGKIIFLYRRAVGVNLKDAFCAALAGLALSHTIAKAVLYGFFTTSIPFFRTPKNADNHGFWVAISEAREEMF
IMLLLWGAALGIYLVQGLPSNDIRFWVVMLLVQSLPYVAALIMAFLSSLPKPAAAPEPAPAA

>Pseudomonas_reactans_P8021_[Orphan] BLAST: A0A7Y8FZP0 Glycosyltransferase
MASRKFGLNLVIVLAIAALFTGFWALINRPVTTPNWPEQISGFSYSPFQQGQYPQKDQYPTDDQMRRDLEIMSKLTDNIR
TYSVDGTLGDIPKLAEEFGLRVTLGIWISPDLERNEREIQRAIEIANSSRSVVRVVVGNEALFREEITPEALIVLLDRVR
AAVKVPVTTSEQWHIWEKNPQLAKHVDLIAAHILPFWEYIPMDKAGQYVLDRARDLKKLFPKKPLLLSEVGWPSNGRMRG
GNETSPADQAIYLRTLVNKLNRQGFNYFVIEAFDQPWKVSDEGSAGAYWGVYNAARQQKFNFEGPVVAIPQWRVLAIGSV
VLALLSLTLLMIDGSALRQRGRTFLTFIAFLCGSVLVWIGYDYSQQYSTWFSVTVGILLALGALGVFIVLLTEAHELAEA
VWTHKRRREFLPVEGDSDYRPKVSIHVPCYNEPPEMVKQTLDALAALDYPDYEVLIIDNNTKDPAVWEPVRDYCETLGPR
FKFFHVAPLAGFKGGALNYLIPHTAKDAEVIAVIDSDYCVSPNWLKHMVPHFADPKIAVVQSPQDYRDQNESTFKKLCYA
EYKGFFHIGMVTRNDRDAIIQHGTMTMTRRSVLEELGWADWCICEDAELGLRVFEKGLSAAYYHDSYGKGLMPDTFIDFK
KQRFRWAYGAIQIIKRHTASLLRGKDTELTRGQRYHFLAGWLPWVADGMNIFFTVGALLWSAAMIIVPTRVDPPLLIFAI
PPLALFVFKVGKIIFLYRRAVGVNLKDAFCAALAGLALSHTIAKAVLYGFFTTSIPFFRTPKNADNHGFWVAISEAREEM
FIMLLLWGAALGIYLVQGLPSNDIRFWIVMLLVQSLPYVAALIMAFLSSLPKPAAAPEPAPAA

>Pseudomonas_resinovorans_CA10_[Orphan] PseudoCAP: Glucan biosynthesis protein
MPSRKFGLNLVVFVAVAALFTGIWALYNRPVTAPDWPEQISGYSFSPFRADQNPQQNRYPTDEQIRQDLELLSKQTDNIR
TYSVDGTLADIPRLAEEFGLRVTLGVWISPDEERNEREIAKAIEIANASRSVVRVVVGNEALFRREISRKDLMVYLDRVR
SAVKVPVTTSEQWHIWLKYPELAKHVDLVAAHVLPYWEFVPMEDSTDFVLERAKDLKKAFPKKPLLLSEVGWPSNGRMRG
GADASQADQAIYLRTLVNALNAKGYNYFVIEAFDQPWKASDEGSVGAYWGVYNLDRQPKFAFEGPVIAIPQWRILAVASV
VMALLALALMLIDGSALRQRGRTFLTFVAFAGGSVLVWIAYDYSQQYSTWFSLTVGMLLGIGALGVFIVLLTEAHELAEA
VWVRKRRRPFLPVLADTAYRPKVSVHVPCYNEPPEMVKQTLDALANLDYPDYEVLIIDNNTKDPAVWEPVRDYCEQLGPR
FKFFHVAPLEGFKGGALNYILPYTAPDAEVIAVIDSDYCVDRNWLKYMVPHFADPDIAVVQSPQDYRDGNENTFKRLCYA
EYKGFFHIGMITRNDRNAIIQHGTMTMIRRNVMDELKWADWTICEDAELGLRVFKSGYAAAYAHQSFGRGLMPDTFIDYK
KQRFRWAYGAIQIMKGHARSLFLGKDSKLKQGQRYHFIAGWLPWVADGLNIFFTAGALLWSAAMIIVPQRVDPPLLIFAI
PPLALFFFKVGKIVFLYQRAVGVNLKDAFCAAVAGLALSHTIAKAVLYGFFTKSIPFFRTPKMRSNHGLLMALAEAREEV
FIMLLLWGAAIGIAVVQGMPSPDVKFWVAMLLVQSLPYLAALIMALLSSLPKGQEEQDEEVATA

>Pseudomonas_salomonii_ICMP_11288_[Orphan] BLAST: A0A3M4Q1B9 Glyco_trans_2-like domain-containing protein
MSSRKFGLNLVVVLAIAALFTGFWALINRPVTTPNWPEQISGFSYSPFQQGQFPQKDQYPTDDQMRQDLAIMSKLTDNIR
TYSVDGTLGDIPKLAEEFGLRVTLGIWISPDLERNEREIQRAIEIANTSRSVVRVVVGNEALFREEITPEALIVLLDRVR
AAVKVPVTTSEQWHIWEKNPQLAKHVDLIAAHILPFWEFIPMDKAGQYVLDRARDLKKLFPKKPLLLSEVGWPSNGRMRG
GNESSPADQAIYLRTLVNKLNRQGYNYFVIEAFDQPWKVSDEGSAGAYWGVYNAARQQKFNFEGPVVAIPQWRVLAIGSV
VLALLSLTLLMIDGSALRQRGRTFLTFIAFLCGSVLVWIGYDYSQQYSTWFSVTVGILLALGALGVFIVLLTEAHELAEA
VWTHKRRREFLPVEGDSHYRPKVSIHVPCYNEPPEMVKQTLDALAALDYPDFEVLIIDNNTKDPAVWEPVRDYCETLGPR
FKFFHVAPLAGFKGGALNYLIPHTAKDAEVIAVIDSDYCVSPNWLKHMVPHFADPKIAVVQSPQDYRDQNESTFKKLCYA
EYKGFFHIGMVTRNDRDAIIQHGTMTMTRRSVLEELGWADWCICEDAELGLRVFEKGLSAAYYHDSYGKGLMPDTFIDFK
KQRFRWAYGAIQIIKRHTASLLRGKGTELTRGQRYHFLAGWLPWVADGMNIFFTVGALLWSAAMIIVPTRVDPPLLIFAI
PPLALFVFKVGKIIFLYRRAVGVNLKDAFCAALAGLALSHTIAKAVLYGFFTTSIPFFRTPKNADNHGFWVAISEAREEM
FIMLLLWGAALGIYLVQGLPSNDIRFWVVMLLVQSLPYVAALIMAFLSSLPKPAPKVEPATAE

>Pseudomonas_simiae_WCS417_[Orphan] PseudoCAP: Beta-(1-3)-glucosyl transferase
MASRKFGLNLVIVLAIAALFTGFWALINRPVTTPNWPEQISGFSYSPFQQGQYPQKNQFPTDDQMRRDLEIMSKLTDNIR
TYSVDGTLGDIPKLAEEFGLRVTLGIWISPDLERNEREIQRAIEIANTSRSVVRVVVGNEAVFRKDITPEALIVLLDRVR
AAVKVPVTTSEQWDIWEKNPQLAKHVDLIAAHILPFWEYIPMDKAGQYVLDRARDLKKLFPKKPLLLSEVGWPSNGRMRG
GNETSPADQAIYLRTLVNKLNRQGFNYFVIEAFDQPWKVSDEGSAGAYWGVYNAARQQKFNFEGPVVAIPQWRVLAIGSV
VLALLSLTLLMIDGSALRQRGRTFLTFIAFLCGSVLVWIGYDYSQQYSTWFSVTVGILLALGALGVFIVLLTEAHELAEA
VWTHKRRREFLPVEGDSDYRPKVSIHVPCYNEPPEMVKQTLDALAALDYPDYEVLIIDNNTKDPAVWEPVRDYCETLGPR
FKFFHVAPLAGFKGGALNYLIPHTAKDAEVIAVIDSDYCVSPNWLKHMVPHFADPKIAVVQSPQDYRDQNESTFKKLCYA

EYKGFFHIGMVTRNDRDAIIQHGTMTMTRRSVLEELGWADWCICEDAELGLRVFEKGLSAAYYHDSYGKGLMPDTFIDFK
KQRFRWAYGAIQIIKRHTASLLRGKDTELTRGQRYHFLAGWLPWVADGMNIFFTVGALLWSAAMIIVPTRVDPPLLIFAI
PPLALFVFKVGKIIFLYRRAVGVNLKDAFCAALAGLALSHTIAKAVLYGFFTTSIPFFRTPKNADNHGFWVAISEAREEM
FIMLLLWGAALGIYLVQGLPSNDIRFWVVMLLVQSLPYVAALIMAFLSSLPKPSAKAEPVTAA

>Pseudomonas_strain_ADAK22_[Orphan] BLAST: A0A7Z3A4T9 Glycosyltransferase
MSSRKFGLNLVVVLAIAALFTGFWALINRPVTTPNWPEQISGFSYSPFQQGQFPQKDQYPSDEEMRRDLEIMSKLTDNIR
TYSVDGTLGDIPKLAEEFGLRVTLGIWISPDLARNEREIQRAIELANSSRSVVRVVVGNEALFREEITPEALIALLDRVR
AAVKVPVTTSEQWHIWEKNPQLAKHVDLIAAHILPFWEFIPMDKAGQYVLDRARDLKKLFPKKPLLLSEVGWPSNGRMRG
GNETSPADQAIYLRTLVNKLNRQGYNYFVIEAFDQPWKVSDEGSAGAYWGVYNAARQQKFNFEGPVVAIPQWRVLAIGSV
VLALLSLTLLMIDGSALRQRGRTFLTFIAFLCGSVLVWIGYDYSQQYSTWFSVTVGILLALGALGVFIVLLTEAHELAEA
VWTHKRRREFLPVEGDSDYRPKVSIHVPCYNEPPEMVKQTLDALAALDYPDYEVLIIDNNTKDPAVWEPVRDYCETLGPR
FKFFHVSPLAGFKGGALNYLIPHTAKDAEVIAVIDSDYCVSPNWLKHMVPHFADPKIAVVQSPQDYRDQNESTFKKLCYA
EYKGFFHIGMVTRNDRDAIIQHGTMTMTRRSVLEELGWADWCICEDAELGLRVFEKGLSAAYYHDSYGKGLMPDTFIDFK
KQRFRWAYGAIQIIKRHTASLLRGKGTELTRGQRYHFLAGWLPWVADGMNIFFTVGALLWSAAMIIVPTRVDPPLLIFAI
PPLALFVFKVGKIIFLYRRAVGVNLKDAFCAALAGLALSHTIAKAVLYGFFTTSIPFFRTPKNADNHGFWVAISEAREEM
FIMLLLWGAALGIYLVQGLPSNDIRFWVVMLLVQSLPYVAALIMAFLSSLPKPAPKVEPATAE

>Pseudomonas_strain_AP19_[Orphan] BLAST: A0A1E4WYG1 Beta-(1-3)-glucosyl
transferase
MSSRKFGLNLVVVLAIAALFTGFWALINRPVTAPNWPEQISGFSYSPFQQGQFPQKDQYPSDEEMRRDLEIMSKLTDNIR
TYSVDGTLGDIPKLAEEFGLRVTLGIWISPDLERNEREIQRAIELANSSRSVVRVVVGNEALFREEITPQALIVLLDRVR
AAVKVPVTTSEQWHIWEKNPQLAKHVDLIAAHILPFWEFIPVDKAGQYVLDRAKDLKKLFPKKPLLLSEVGWPSNGRMRG
GNETSPADQAVYLRTLVNKLNRQGYNYFVIEAFDQPWKVSDEGSAGAYWGVYNAARQQKFNFEGPVVAIPQWRVLAIGSV
VLALLSLTLLMIDGSALRQRGRTFLTFIAFLCGSVLVWIGYDYSQQYSTWFSVTVGILLALGALGVFIVLLTEAHELAEA
VWTHKRRREFLPVEGDSDYRPKVSIHVPCYNEPPEMVKQTLDALAALDYPDYEVLIIDNNTKDPAVWEPVRDYCETLGPR
FKFFHVSPLAGFKGGALNYLIPHTAKDAEVIAVIDSDYCVSPNWLKHMVPHFADPKIAVVQSPQDYRDQNESTFKKLCYA
EYKGFFHIGMVTRNDRDAIIQHGTMTMTRRSVLEELGWADWCICEDAELGLRVFEKGLSAAYYHDSYGKGLMPDTFIDFK
KQRFRWAYGAIQIIKRHTASLLRGKGTELTRGQRYHFLAGWLPWVADGMNIFFTVGALLWSAAMIIVPTRVDPPLLIFAI
PPLALFVFKVGKIIFLYRRAVGVNLKDAFCAALAGLALSHTIAKAVLYGFFTTSIPFFRTPKNADNHGFWVAISEAREEM
FIMLLLWGAALGIYLVQGLPSNDIRFWVVMLLVQSLPYVAALIMAFLSSLPKPAPKGEPATAE

>Pseudomonas_strain_C_49-2_[Orphan] BLAST: A0A432ACG5 Glycosyltransferase
MASRKFGLNLVIVLAIAALFTGFWALINRPVTTPNWPEQISGFSYSPFQQGQYPQKDQYPTDDQMRRDLEIMSKLTDNIR
TYSVDGTLGDIPKLAEEFGLRVTLGIWISPDLERNEREIQRAIEIANSSRSVVRVVVGNEALFREEITPEALIVLLDRVR
AAVKVPVTTSEQWHIWEKNPQLAKHVDLIAAHILPFWEYIPMDKAGQYVLDRARDLKKLFPKKPLLLSEVGWPSNGRMRG
GNETSPADQAIYLRTLVNKLNRQGFNYFVIEAFDQPWKVSDEGSAGAYWGVYNAARQQKFNFEGPVVAIPQWRVLAIGSV
VLALLSLTLLMIDGSALRQRGRTFLTFIAFLCGSVLVWIGYDYSQQYSTWFSVTVGILLALGALGVFIVLLTEAHELAEA
VWTHKRRREFLPVEGDSDYRPKVSIHVPCYNEPPEMVKQTLDALAALDYPDYEVLIIDNNTKDPAVWEPVRDYCETLGPR
FKFFHVAPLAGFKGGALNYLIPHTAKDAEVIAVIDSDYCVSPNWLKHMVPHFADPKIAVVQSPQDYRDQNESTFKKLCYA
EYKGFFHIGMVTRNDRDAIIQHGTMTMTRRSVLEELGWADWCICEDAELGLRVFEKGLSAAYYHDSYGKGLMPDTFIDFK
KQRFRWAYGAIQIIKRHTASLLRGKDTELTRGQRYHFLAGWLPWVADGMNIFFTVGALLWSAAMIIVPTRVDPPLLIFAI
PPLALFVFKVGKIIFLYRRAVGVNLKDAFCAALAGLALSHTIAKAVLYGFFTTSIPFFRTPKNADNHGFWVAISEAREEM
FIMLLLWGAALGIYLVQGLPSNDIRFWVVMLLVQSLPYVAALIMAFLSSLPKPAAAPEPVPAA

>Pseudomonas_strain_DBG-1_[Orphan] Unpublished genome: BcsA-like dapE-associated
Orphan
MSSRKFGLNLVVVLAIAALFTGFWALINRPVSAPNWPEQISGFSYSPFQQGQYPQKDQYPTDDEMRRDLEIMSKLTDNIR
IYSVDGSLQDIPKLAEEFGLRVTLGIWISPDQERNEREITRAIELANTSRSVVRVVVGNEAIFRKEITAQELSVLLDRVR
AAVKVPVTTSEQWHVWEEHPELAKHVDLIAAHVLPYWEFIPVDKAGQFVFDRARDLKKLFPKKPLLLSEVGWPSNGRMRG
GADASPADQAIYLRTLVNKLNRQGFNYFVIEAFDQPWKASDEGSVGAYWGVFNAARQQKFNFEGPVVAIPQWRVLAIGSV
VLALLSLTLLMIDGSALRQRGRIFLTFIAFLCGSVLVWIGYDYSLQYSTWFSLTVGFLLALGALGVFIVLLTEAHELAEA
VWIHKRRREFLPVEGDSSYRPKVSIHVPCYNEPPDMVKQTLDALAALDYPDYEVLIIDNNTKDPAVWEPVRDYCETLGPR
FKFFHVSPLAGFKGGALNYLIPHTAKDAEVIAVIDSDYCVHPNWLKHMVPHFADPKIAVVQSPQDYRDQNESTFKKLCYA
EYKGFFHIGMVTRNDRDAIIQHGTMTMTRRSVLEELGWADWCICEDAELGLRVFEKGLSAAYYHDSYGKGLMPDTFIDFK
KQRFRWAYGAIQIIKRHTGSLLRGKDTELTRGQRYHFLAGWLPWVADGMNIFFTVGALLWSAAMIIVPQRVDPPLLIFAI
PPLALFVFKVGKIIFLYRRAVGVNLKDAFCAALAGLALSHTIAKAVLYGFFTSSIPFFRTPKNADNHGFWVAISEAREEL
FIMLLLWGAALGIFLVNGMPSNDMRFWVVMLLVQSLPYLAALIMAFLSSLPKPAAAAEPAPVV

>Pseudomonas_strain_DBG-3_[Orphan] Unpublished genome: BcsA-like dapE-associated
Orphan

MSSRKFGLNLVVVLAIAALFTGFWALINRPVTTPNWPEQISGFSYSPFQQGQFPQKDQYPSDEEMRRDLEIMSKLTDNIR
TYSVDGTLGDIPKLAEEFGLRVTLGIWISPDLARNEREIQRAIELANSSRSVVRVVVGNEALFREEITPEALIALLDRVR
AAVKVPVTTSEQWHIWEKNPQLAKHVDLIAAHILPFWEFIPMDKAGQYVLDRARDLKKLFPKKPLLLSEVGWPSNGRMRG
GNETSPADQAIYLRTLVNKLNRQGYNYFVIEAFDQPWKVSDEGSAGAYWGVYNAARQQKFNFEGPVVAIPQWRVLAIGSV
VLALLSLTLLMIDGSALRQRGRTFLTFIAFLCGSVLVWIGYDYSQQYSTWFSVTVGILLALGALGVFIVLLTEAHELAEA
VWTHKRRREFLPVEGDSDYRPKVSIHVPCYNEPPEMVKQTLDALAALDYPDYEVLIIDNNTKDPAVWEPVRDYCETLGPR
FKFFHVSPLAGFKGGALNYLIPHTAKDAEVIAVIDSDYCVSPNWLKHMVPHFADPKIAVVQSPQDYRDQNESTFKKLCYA
EYKGFFHIGMVTRNDRDAIIQHGTMTMTRRSVLEELGWADWCICEDAELGLRVFEKGLSAAYYHDSYGKGLMPDTFIDFK
KQRFRWAYGAIQIIKRHTASLLRGKGTELTRGQRYHFLAGWLPWVADGMNIFFTVGALLWSAAMIIVPTRVDPPLLIFAI
PPLALFVFKVGKIIFLYRRAVGVNLKDAFCAALAGLALSHTIAKAVLYGFFTTSIPFFRTPKNADNHGFWVAISEAREEM
FIMLLLWGAALGIYLVQGLPSNDIRFWVVMLLVQSLPYVAALIMAFLSSLPKPAPKVEPATAE

>Pseudomonas_strain_DBG-6_[Orphan] Unpublished genome: BcsA-like dapE-associated Orphan
MSSRKFGLNLVVVLAIAALFTGFWALINRPVTTPNWPEQISGFSYSPFQQGQFPQKDQYPTDDQMRQDLAIMSKLTDNIR
TYSVDGTLGDIPKLSEEFGLRVTLGIWISPDLERNEREIQRAIEIANTSRSVVRVVVGNEALFREEITPEALIVLLDRVR
AAVKVPVTTSEQWHIWEKNPQLAKHVDLIAAHILPFWEFIPMDKAGQYVLDRARDLKKLFPKKPLLLSEVGWPSNGRMRG
GNESSPADQAIYLRTLVNKLNRQGYNYFVIEAFDQPWKVSDEGSAGAYWGVYNAARQQKFNFEGPVVAIPQWRVLAIGSV
VLALLSLTLLMIDGSALRQRGRTFLTFIAFLCGSVLVWIGYDYSQQYSTWFSVTVGILLALGALGVFIVLLTEAHELAEA
VWTHKRRREFLPVEGDSHYRPKVSIHVPCYNEPPEMVKQTLDALAALDYPDFEVLIIDNNTKDPAVWEPVRDYCETLGPR
FKFFHVSPLAGFKGGALNYLIPHTAKDAEVIAVIDSDYCVSPNWLKHMVPHFADPKIAVVQSPQDYRDQNESTFKKLCYA
EYKGFFHIGMVTRNDRDAIIQHGTMTMTRRSVLEELGWADWCICEDAELGLRVFEKGLSAAYYHDSYGKGLMPDTFIDFK
KQRFRWAYGAIQIIKRHTASLLRGKGTELTRGQRYHFLAGWLPWVADGMNIFFTVGALLWSAAMIIVPTRVDPPLLIFAI
PPLALFVFKVGKIIFLYRRAVGVNLKDAFCAALAGLALSHTIAKAVLYGFFTTSIPFFRTPKNADNHGFWVAISEAREEM
FIMLLLWGAALGIYLVQGLPSNDMRFWVVMLLVQSLPYVAALIMAFLSSLPKPAPKVEPATAE

>Pseudomonas_strain_DBG-15_[Orphan] Unpublished genome: BcsA-like dapE-associated Orphan
MSSRKFGLNLVVVLAIAALFTGFWALINRPVTTPNWPEQISGFSYSPFQQGQFPQKDQYPSDEEMRRDLEIMSKLTDNIR
TYSVDGTLGDIPKLAEEFGLRVTLGIWISPDLARNEREIQRAIELANSSRSVVRVVVGNEALFREEITPEALIALLDRVR
AAVKVPVTTSEQWHIWEKNPQLAKHVDLIAAHILPFWEFIPMDKAGQYVLDRARDLKKLFPKKPLLLSEVGWPSNGRMRG
GNETSPADQAIYLRTLVNKLNRQGYNYFVIEAFDQPWKVSDEGSAGAYWGVYNAARQQKFNFEGPVVAIPQWRVLAIGSV
VLALLSLTLLMIDGSALRQRGRTFLTFIAFLCGSVLVWIGYDYSQQYSTWFSVTVGVLLALGALGVFIVLLTEAHELAEA
VWTHKRRREFLPVEGDSDYRPKVSIHVPCYNEPPEMVKQTLDALAALDYPDYEVLIIDNNTKDPAVWEPVRDYCETLGPR
FKFFHVSPLAGFKGGALNYLIPHTAKDAEVIAVIDSDYCVSPNWLKHMVPHFADPKIAVVQSPQDYRDQNESTFKKLCYA
EYKGFFHIGMVTRNDRDAIIQHGTMTMTRRSVLEELGWADWCICEDAELGLRVFEKGLSAAYYHDSYGKGLMPDTFIDFK
KQRFRWAYGAIQIIKRHTASLLRGKGTELTRGQRYHFLAGWLPWVADGMNIFFTVGALLWSAAMIIVPTRVDPPLLIFAI
PPLALFVFKVGKIIFLYRRAVGVNLKDAFCAALAGLALSHTIAKAVLYGFFTTSIPFFRTPKNADNHGFWVAISEAREEM
FIMLLLWGAALGIYLVQGLPSNDIRFWVVMLLVQSLPYVAALIMAFLSSLPKPAPKVEPATAE

>Pseudomonas_strain_DBG-16_[BcsA] Unpublished genome: Cellulose synthase catalytic subunit [UDP-forming]
MTDTTSSTPFVEGRAEQRLNGAIARFNRWPTALRTVLVFTSCVLGALLLLGIISAPLDLYSQCLFAAVCFMAVLVLRKIP
GRLAILALVVLSLVASLRYMFWRLTSTLGFETWVDMFFGYGLVAAEFYALVVLIFGYVQTAWPLRRTPVWLKTEPEEWPT
VDVFIPTYNEALSIVKLTIFAAQAMDWPKDKLRVHVLDDGRRDDFRDFCRKVGVNYIRRDNNFHAKAGNLNEALKITDGE
YVALFDADHVPTRSFLQVSLGWFLKDPKLAMLQTPHFFFSPDPFEKNLDTFRAVPNEGELFYGLVQDGNDLWNATFFCGS
CAVIRRKPLLEIGGVAVETVTEDAHTALKLNRLGYNTAYLAIPQAAGLATESLSRHINQRIRWARGMAQIFRTDNPLLGK
GLKWGQRICYANAMLHFFYGLPRLVFLTAPLAYLVFGAEIFHASALMIVAYVLPHLVHSSLTNSRIQGRFRHSFWNEVYE
TVLAWYILPPVLVALVNPKAGGFNVTDKGGIIDKQFFDWKLARPYLVLLAVNLVGLGCGIHQLVWGDESTAVTVAINLTW
TLYNLIITSAAVAVASEARQVRSEPRVSAKLPVSIICADGRVLDGVTQDFSQNGFGLMLSDGHSITQGERVQLVLSRNGQ
DSLFDARVVFSKGAQIGAQFEALSLRQQSELVRLTFSRADTWAASWGAGQPDTPLAALREVGSIGIGGLFTLGRATLHEL
RLALSRTPKQPLDTLMDKP

>Pseudomonas_strain_DBG-16_[Orphan] Unpublished genome: BcsA-like dapE-associated Orphan
MSSRKFGLNLVVVLAIAALFTGFWALINRPVTTPNWPEQISGFSYSPFQQGQYPQKNQFPTDEQMRQDLAIMSKLTDNIR
TYSVDGTLGDIPKLAEEFGLRVTLGIWISPDLERNEREIQRAIEIANNSRSVVRVVVGNEAVFRKDITPEALIVLLDRVR
AAVKVPVTTSEQWDIWEKNPQLAKHVDLIAAHILPFWEYIPMDKAGQYVLDRAKDLKKLFPKKPLLLSEVGWPSNGRMRG
GNETSPADQAIYLRTLVNKLNRQGYNYFVIEAFDQPWKVSDEGSAGAYWGVFNGARQQKFNFEGPVVAIPQWRVLAIGSV
VLALLSLTLLMIDGSALRQRGRTFLTFIAFLCGSVLVWIGYDYSQQYSTWFSVTVGILLALGALGVFIVLLTEAHELAEA
VWTHKRRREFLPVEGDSDYRPKVSIHVPCYNEPPEMVKQTLDALAALDYPDYEVLIIDNNTKDPAVWEPVRDYCETLGPR

FKFFHVAPLAGFKGGALNYLIPHTAKDAEVIAVIDSDYCVSPNWLKHMVPHFADPKIAVVQSPQDYRDQNESTFKKLCYA
EYKGFFHIGMVTRNDRDAIIQHGTMTMTRRSVLEELGWADWCICEDAELGLRVFEKGLSAAYYHDSYGKGLMPDTFIDFK
KQRFRWAYGAIQIIKRHTASLLRGKDTELTRGQRYHFLAGWLPWVADGMNIFFTVGALLWSAAMIIVPTRVDPPLLIFAI
PPLALFVFKVGKIIFLYRRAVGVNLKDAFCAALAGLALSHTIAKAVLYGFFTTSIPFFRTPKNADNHGFWVAISEAREEM
FIMLLLWGAALGIYLVQGLPSNDIRFWVVMLLVQSLPYVAALIMAFLSSLPKPAPAPEPAPAA

>Pseudomonas_strain_DBG-23_[BcsA] Unpublished genome: Cellulose synthase catalytic subunit [UDP-forming]
MTDTTSSTPFVEGRAEQRLNGAIARFNRWPTALRTVLVFTSCVLGALLLLGIISAPLDLYSQCLFAAVCFMAVLVLRKIP
GRLAILALVVLSLVASLRYMFWRLTSTLGFETWVDMFFGYGLVAAEFYALIVLIFGYVQTAWPLRRTPVWLKTEPEEWPT
VDVFIPTYNEALSIVKLTIFAAQAMDWPKDKLRVHVLDDGRRDDFRDFCRKVGVNYIRRDNNFHAKAGNLNEALKITDGE
YVALFDADHVPTRSFLQVSLGWFLKDPKLAMLQTPHFFFSPDPFEKNLDTFRAVPNEGELFYGLVQDGNDLWNATFFCGS
CAVIRRKPLLEIGGVAVETVTEDAHTALKLNRLGYNTAYLAIPQAAGLATESLSRHINQRIRWARGMAQIFRTDNPLLGK
GLKWGQRICYANAMLHFFYGLPRLVFLTAPLAYLVFGAEIFHASALMIVAYVLPHLVHSSLTNSRIQGRFRHSFWNEVYE
TVLAWYILPPVLVALVNPKAGGFNVTDKGGIIDKQFFDWKLARPYLVLLAVNLVGLGCGIHQLVWGDESTAVTVAINLTW
TLYNLIITSAAVAVASEARQVRSEPRVSAKLPVSIICADGRVLDGVTQDFSQNGFGLMLSDGHSITQGERVQLVLSRNGQ
DSLFDARVVFSKGAQIGAQFEALSLRQQSELVRLTFSRADTWAASWGAGQPDTPLAALREVGSIGIGGLFTLGRATLHEL
RLALSRTPKQPLDTLMDKP

>Pseudomonas_strain_DBG-23_[Orphan] Unpublished genome: BcsA-like dapE-associated Orphan
MSSRKFGLNLVVVLAIAALFTGFWALINRPVTTPNWPEQISGFSYSPFQQGQYPQKNQFPTDEQMRQDLAIMSKLTDNIR
TYSVDGTLGDIPKLAEEFGLRVTLGIWISPDLERNEREIQRAIEIANNSRSVVRVVVGNEAVFRKDITPEALIVLLDRVR
AAVKVPVTTSEQWDIWEKNPQLAKHVDLIAAHILPFWEYIPMDKAGQYVLDRAKDLKKLFPKKPLLLSEVGWPSNGRMRG
GNETSPADQAIYLRTLVNKLNRQGYNYFVIEAFDQPWKVSDEGSAGAYWGVFNGARQQKFNFEGPVVAIPQWRVLAIGSV
VLALLSLTLLMIDGSALRQRGRTFLTFIAFLCGSVLVWIGYDYSQQYSTWFSVTVGILLALGALGVFIVLLTEAHELAEA
VWTHKRRREFLPVEGDSDYRPKVSIHVPCYNEPPEMVKQTLDALAALDYPDYEVLIIDNNTKDPAVWEPVRDYCETLGPR
FKFFHVAPLAGFKGGALNYLIPHTAKDAEVIAVIDSDYCVSPNWLKHMVPHFADPKIAVVQSPQDYRDQNESTFKKLCYA
EYKGFFHIGMVTRNDRDAIIQHGTMTMTRRSVLEELGWADWCICEDAELGLRVFEKGLSAAYYHDSYGKGLMPDTFIDFK
KQRFRWAYGAIQIIKRHTASLLRGKDTELTRGQRYHFLAGWLPWVADGMNIFFTVGALLWSAAMIIVPTRVDPPLLIFAI
PPLALFVFKVGKIIFLYRRAVGVNLKDAFCAALAGLALSHTIAKAVLYGFFTTSIPFFRTPKNADNHGFWVAISEAREEM
FIMLLLWGAALGIYLVQGLPSNDIRFWVVMLLVQSLPYVAALIMAFLSSLPKPAPAPEPAPAA

>Pseudomonas_strain_FH1_[Orphan] BLAST: W2D6X9 Putative beta-(1-3)-glucosyl transferase
MASRKFGLNLVIVLAIAALFTGFWALINRPVTTPNWPEQISGFSYSPFQQGQYPQKDQYPTDDQMRRDLEIMSKLTDNIR
TYSVDGTLGDIPKLAEEFGLRVTLGIWISPDLERNEREIQRAIEIANSSRSVVRVVVGNEALFREEITPEALIVLLDRVR
AAVKVPVTTSEQWHIWEKNPQLAKHVDLIAAHILPFWEYIPMDKAGQYVLDRARDLKKLFPKKPLLLSEVGWPSNGRMRG
GNETSPADQAIYLRTLVNKLNRQGFNYFVIEAFDQPWKVSDEGSAGAYWGVYNAARQQKFNFEGPVVAIPQWRVLAIGSV
VLALLSLTLLMIDGSALRQRGRTFLTFIAFLCGSVLVWIGYDYSQQYSTWFSVTVGILLALGALGVFIVLLTEAHELAEA
VWTHKRRREFLPVEGDSDYRPKVSIHVPCYNEPPEMVKQTLDALAALDYPDYEVLIIDNNTKDPAVWEPVRDYCETLGPR
FKFFHVAPLAGFKGGALNYLIPHTAKDAEVIAVIDSDYCVSPNWLKHMVPHFADPKIAVVQSPQDYRDQNESTFKKLCYA
EYKGFFHIGMVTRNDRDAIIQHGTMTMTRRSVLEELGWADWCICEDAELGLRVFEKGLSAAYYHDSYGKGLMPDTFIDFK
KQRFRWAYGAIQIIKRHTASLLRGKDTELTRGQRYHFLAGWLPWVADGMNIFFTVGALLWSAAMIIVPTRVDPPLLIFAI
PPLALFVFKVGKIIFLYRRAVGVNLKDAFCAALAGLALSHTIAKAVLYGFFTTSIPFFRTPKNADNHGFWVAISEAREEM
FIMLLLWGAALGIYLVQGLPSNDIRFWVVMLLVQSLPYVAALIMAFLSSLPKPAAAPEPAPAA

>Pseudomonas_strain_IB20_[Orphan] BLAST: A0A263NWD8 Beta-(1-3)-glucosyl transferase
MSSRKFGLNLVVVLAIAALFTGFWALINRPVTAPNWPEQISGFSYSPFQQGQFPQKDQYPSDEEMRRDLEIMSKLTDNIR
TYSVDGTLGDIPKLAEEFGLRVTLGIWISPDLERNEREIRAIELANSSRSVVRVVVGNEALFREEITPEALIVLLDRVRG
AVKVPVTTSEQWHIWEKNPQLAKHVDLIAAHILPFWEFIPMDKAGQYVLDRARDLKKLFPKKPLLLSEVGWPSNGRMRGG
NETSPADQAIYLRTLVNKLNRQGYNYFVIEAFDQPWKVSDEGSAGAYWGVYNAARQQKFNFEGPVVAIPQWRVLAIGSVV
LALLSLTLLMIDGSSLRQRGRTFLTFIAFLCGSVLVWIGYDYSQQYSTWFSVTVGILLALGALGVFIVLLTEAHELAEAV
WTHKRRREFLPVEGDSDYRPKVSIHVPCYNEPPEMVKQTLDALAALDYPDYEVLIIDNNTKDPAVWEPVRDYCETLGPFK
FFHVSPLAGFKGGALNYLIPHTAKDAEVIAVIDSDYCVSPNWLKHMVPHFADPKIAVVQSPQDYRDQNESTFKKLCYAEY
KGFFHIGMVTRNDRDAIIQHGTMTMTRRSVLEELGWADWCICEDAELGLRVFEKGLSAAYYHDSYGKGLMPDTFIDFKKQ
RFRWAYGAIQIIKRHTASLLRGKDTELTRGQRYHFLAGWLPWVADGMNIFFTVGALLWSAAMIIVPTRVDPPLLIFAIPP
LALFVFKVGKIVFLYRRAVGVNLKDAFCAALAGLALSHTIAKAVLYGFFTTSIPFFRTPKNADNHGFWVAISEAREEMFI
MLLLWGAALGIYLVQGLPSNDIRFWVVMLLVQSLPYVAALIMAFLSSLPKPAPAGEPATAE

>Pseudomonas_strain_LBUM920_[Orphan] BLAST: A0A3G8CXC2 Beta-(1-3)-glucosyl transferase
MSSRKFGLNLVVVLAIAALFTGFWALINRPVTTPNWPEQISGFSYSPFQQGQFPQKDQYPSDEEMRRDLEIMSKLTDNIR
TYSVDGTLGDIPKLAEEFGLRVTLGIWISPDLARNEREIQRAIELANSSRSVVRVVVGNEALFREEITPEALIALLDRVR
AAVKVPVTTSEQWHIWEKNPQLAKHVDLIAAHILPFWEFIPMDKAGQYVLDRARDLKKLFPKKPLLLSEVGWPSNGRMRG
GNETSPADQAIYLRTLVNKLNRQGYNYFVIEAFDQPWKVSDEGSAGAYWGVYNAARQQKFNFEGPVVAIPQWRVLAIGSV
VLALLSLTLLMIDGSALRQRGRTFLTFIAFLCGSVLVWIGYDYSQQYSTWFSVTVGILLALGALGVFIVLLTEAHELAEA
VWTHKRRREFLPVEGDSDYRPKVSIHVPCYNEPPEMVKQTLDALAALDYPDYEVLIIDNNTKDPAVWEPVRDYCETLGPR
FKFFHVSPLAGFKGGALNYLIPHTAKDAEVIAVIDSDYCVSPNWLKHMVPHFADPKIAVVQSPQDYRDQNESTFKKLCYA
EYKGFFHIGMVTRNDRDAIIQHGTMTMTRRSVLEELGWADWCICEDAELGLRVFEKGLSAAYYHDSYGKGLMPDTFIDFK
KQRFRWAYGAIQIIKRHTASLLRGKGTELTRGQRYHFLAGWLPWVADGMNIFFTVGALLWSAAMIIVPTRVDPPLLIFAI
PPLALFVFKVGKIIFLYRRAVGVNLKDAFCAALAGLALSHTIAKAVLYGFFTTSIPFFRTPKNADNHGFWVAISEAREEM
FIMLLLWGAALGIYLVQGLPSNDIRFWVVMLLVQSLPYVAALIMAFLSSLPKPAPKVEPATAE

>Pseudomonas_strain_Leaf15_[Orphan] BLAST: A0A0Q4EZK1 Beta-(1-3)-glucosyl transferase
MSSRKFGLNLVVVLAIAALFTGFWALINRPVTTPNWPEQISGFSYSPFQQGQFPQKDQYPTDDQMRQDLAIMSKLTDNIR
TYSVDGTLGDIPKLAEEFGLRVTLGIWISPDLERNEREIQRAIEIANTSRSVVRVVVGNEALFREEITPEALIVLLDRVR
AAVKVPVTTSEQWHIWEKNPQLAKHVDLIAAHILPFWEFIPMDKAGQYVLDRARDLKKLFPKKPLLLSEVGWPSNGRMRG
GNESSPADQAIYLRTLVNKLNRQGYNYFVIEAFDQPWKVSDEGSAGAYWGVYNAARQQKFNFEGPVVAIPQWRVLAIGSV
VLALLSLTLLMIDGSALRQRGRTFLTFIAFLCGSVLVWIGYDYSQQYSTWFSVTVGILLALGALGVFIVLLTEAHELAEA
VWTHKRRREFLPVEGDSDYRPKVSIHVPCYNEPPEMVKQTLDALAALDYPDFEVLIIDNNTKDPAVWEPVRDYCETLGPR
FKFFHVAPLAGFKGGALNYLIPHTAKDAEVIAVIDSDYCVSPNWLKHMVPHFADPKIAVVQSPQDYRDQNESTFKKLCYA
EYKGFFHIGMVTRNDRDAIIQHGTMTMTRRSVLEELGWADWCICEDAELGLRVFEKGLSAAYYHDSYGKGLMPDTFIDFK
KQRFRWAYGAIQIIKRHTASLLRGKGTELTRGQRYHFLAGWLPWVADGMNIFFTVGALLWSAAMIIVPTRVDPPLLIFAI
PPLALFVFKVGKIIFLYRRAVGVNLKDAFCAALAGLALSHTIAKAVLYGFFTTSIPFFRTPKNADNHGFWVAISEAREEM
FIMLLLWGAALGIYLVQGLPSNDIRFWVVMLLVQSLPYVAALIMAFLSSLPKPAPKVEPATAE

>Pseudomonas_strain_Leaf98_[Orphan] BLAST: A0A327GS23 Beta-(1-3)-glucosyl transferase
MSSRKFGLNLVVVLAIAALFTGFWALINRPVTTPNWPEQISGFSYSPFQQGQFPQKDQYPTDDQMRQDLAIMSKLTDNIR
TYSVDGTLGDIPKLAEEFGLRVTLGIWISPDLERNEREIQRAIEIANTSRSVVRVVVGNEALFREEITPEALIVLLDRVR
AAVKVPVTTSEQWHIWEKNPQLAKHVDLIAAHILPFWEFIPMDKAGQYVLDRARDLKKLFPKKPLLLSEVGWPSNGRMRG
GNESSPADQAIYLRTLVNKLNRQGYNYFVIEAFDQPWKVSDEGSAGAYWGVYNAARQQKFNFEGPVVAIPQWRVLAIGSV
VLALLSLTLLMIDGSALRQRGRTFLTFIAFLCGSVLVWIGYDYSQQYSTWFSVTVGILLALGALGVFIVLLTEAHELAEA
VWTHKRRREFLPVEGDSDYRPKVSIHVPCYNEPPEMVKQTLDALAALDYPDFEVLIIDNNTKDPAVWEPVRDYCETLGPR
FKFFHVAPLAGFKGGALNYLIPHTAKDAEVIAVIDSDYCVSPNWLKHMVPHFADPKIAVVQSPQDYRDQNESTFKKLCYA
EYKGFFHIGMVTRNDRDAIIQHGTMTMTRRSVLEELGWADWCICEDAELGLRVFEKGLSAAYYHDSYGKGLMPDTFIDFK
KQRFRWAYGAIQIIKRHTASLLRGKGTELTRGQRYHFLAGWLPWVADGMNIFFTVGALLWSAAMIIVPTRVDPPLLIFAI
PPLALFVFKVGKIIFLYRRAVGVNLKDAFCAALAGLALSHTIAKAVLYGFFTTSIPFFRTPKNADNHGFWVAISEAREEM
FIMLLLWGAALGIYLVQGLPSNDIRFWVVMLLVQSLPYVAALIMAFLSSLPKPAPKVEPATAE

>Pseudomonas_strain_NS1_2017_[Orphan] BLAST: A0A223VPF4 Beta-(1-3)-glucosyl transferase
MASRKFGLNLVIVLAIAALFTGFWALINRPVTTPNWPEQISGFSYSPFQQGQYPQKDQYPTDDQMRRDLEIMSKLTDNIR
TYSVDGTLGDIPKLAEEFGLRVTLGIWISPDLERNEREIQRAIEIANSSRSVVRVVVGNEALFREEITPEALIVLLDRVR
AAVKVPVTTSEQWHIWEKNPQLAKHVDLIAAHILPFWEYIPMDKAGQYVLDRARDLKKLFPKKPLLLSEVGWPSNGRMRG
GNETSPADQAIYLRTLVNKLNRQGFNYFVIEAFDQPWKVSDEGSAGAYWGVYNAARQQKFNFEGPVVAIPQWRVLAIGSV
VLALLSLTLLMIDGSALRQRGRTFLTFIAFLCGSVLVWIGYDYSQQYSTWFSVTVGILLALGALGVFIVLLTEAHKLAEA
VWTHKRRREFLPVEGDSDYRPKVSIHVPCYNEPPEMVKQTLDALAALDYPDYEVLIIDNNTKDPAVWEPVRDYCETLGPR
FKFFHVAPLAGFKGGALNYLIPHTAKDAEVIAVIDSDYCVSPNWLKHMVPHFADPKIAVVQSPQDYRDQNESTFKKLCYA
EYKGFFHIGMVTRNDRDAIIQHGTMTMTRRSVLEELGWADWCICEDAELGLRVFEKGLSAAYYHDSYGKGLMPDTFIDFK
KQRFRWAYGAIQIIKRHTASLLRGKDTELTRGQRYHFLAGWLPWVADGMNIFFTVGALLWSAAMIIVPTRVDPPLLIFAI
PPLALFVFKVGKIIFLYRRAVGVNLKDAFCAALAGLALSHTIAKAVLYGFFTTSIPFFRTPKNADNHGFWVAISEAREEM
FIMLLLWGAALGIYLVQGLPSNDIRFWVVMLLVQSLPYVAALIMAFLSSLPKPAVKAEPVTAV

>Pseudomonas_strain_NZ092_[BcsA] Unpublished genome: Cellulose synthase catalytic subunit [UDP-forming]
MIRDPNPTSHLPGRSELRLNDWSTRFKQWPGALRRALILGGALVAGLLLVSIISAPLDLYTQCLFAALCFGSALLIKRLP
GRLPILALIVLSLVASLRYLYWRLTDTLGFDGWLDILFGYGLVLAEIYALVVLVFGYVQTAWPLRRRPVMLAGDPGDWPT
VDVFIPTYNETLSIVKLSIFAAQAMDWPKDKLRVHVLDDGRRDEFRRFCQSIGVNYITRDNNFHAKAGNLNEALKVTDGE

YIAMFDADHVPTRSFLQIGMGWFLKDPKLAMLQTPHFFFSPDPFEKNLNTFRAVPNEGELFYGLVQDGNDLWNATFFCGS
CAILRRKPLEEIGGVAVETVTEDAHTALKLNRAGYNTAYLAIPQAAGLATESLSRHISQRIRWARGMAQIFRLDNPLVGK
GLNLGQRICYANAMLHFFYGLPRLVFLTAPLAYLIFGAQIFHASALMITAYALPHLFHSNLTNSCIQGRFRHSFWNEVYE
TVLAWYIMPPVLMALVNPKFGGFNVTDKGGIIDKEYFDWKLARPYIVLLVLNVTGLGFGIHQLIWGAPDTAITVLINCVW
TLYNLIITSAAVAVASETRQVRAEPRVRAELPVRIELADGTEVYGTTQDFSQRGLGLKLPAGTTVAAGERVRVSLFRNSR
TSQFDTTVVFSKGQYLGALFDPLSLRQQSELVRMTFSRADTWAASWGSGKLDTPLSALREVSAIGLVGMGALLKAVALQA
RDRLVARSAPSQPLEPLMDKS

>Pseudomonas_strain_NZ092_[Orphan] Unpublished genome: BcsA-like dapE-associated Orphan
MSSRKFGLNLVVVLAIAALFTGFWALINRPVSAPDWPEHISGFSYSPFQQGQFPQKDQFPTDDDMRRDLEIISKLTDNIR
IYSVDGTLQDIPKLAEEFGLRVTLGIWISPDQERNEREITRAIELANTSRSVVRVVVGNEAIFRKEITAKELGVLLDRVR
AAVKVPVTTSEQWHIWEHNPELAKHVDLIAAHILPYWEFPMEKSGQYVLDRARELKRMFPKKPLLLSEVGWPSNGRMRGG
ADATQADQAIYLRTLVNKLNRQGFNYFVIEAFDQPWKASDEGSVGAYWGVYNAARQQKFNFEGPVVAIPQWRVLAIGSVV
LAMLSLAMLLIDGSALRQRGRTFLTFIAFLCGSVLVWIGYDYSQQYSTWFSLTVGFLLALGALGVFIVLLTEAHELAEAV
WTHKRRREFLPVEGDSHYRPKVSIHVPCYNEPPEMVKQTLDALAALDYPDYEVLIIDNNTKDPAVWEPVQAYCETLGPRF
RFFHVAPLAGFKGGALNYLIPHTAKDAEVIAVIDSDYCVDRNWLKHMVPHFADPKIAIVQSPQDYRDQNESTFKKLCYSE
YKGFFHIGMVTRNDRDAIIQHGTMTMTRRSVLEELGWADWCICEDAELGLRVFEKGLSAAYHHHSYGKGLMPDTFIDFKK
QRFRWAYGAIQIIKRHTASLLRGKDTELTRGQRYHFLAGWLPWVADGMNIFFTFGALLWSSAMIIVPQRVDPPLLIFAIP
PLALFVFKVGKILFLYRRAVGVNMKDAFAAALAGLALSHTIAKAVLYGFFTSSIPFFRTPKNADNHGLWVAISEAREELF
IMLLLWGAALGIFLVQGLPSNDMRFWVTMLLVQSLPYLAALVMAFVSSLPKPVEAPEQVPAA

>Pseudomonas_strain_OV546_[Orphan] BLAST: A0A1I7KL48 Exo-beta-1,3-glucanase, GH17 family
MASRKFGLNLVIVLAIAALFSGFWALINRPVTAPNWPEQISGFSYSPFQQGQYPQKDQYPTDDQMRRDLEIMSKLTDNIR
TYSVDGTLGDIPKLAEEFGLRVTLGIWISPDLERNEREIQRAIEIANSSRSVVRVVVGNEALFREEITPEALIVLLDRVR
AAVKVPVTTSEQWHIWEKNPQLAKHVDLIAAHILPFWEYIPMDKAGQYVLDRARDLKKLFPKKPLLLSEVGWPSNGRMRG
GNETSPADQAIYLRTLVNKLNRQGFNYFVIEAFDQPWKVSDEGSAGAYWGVYNAARQQKFNFDGPVVAIPQWRVLAIGSV
VLALLSLTLLMIDGSSLRQRGRTFLTFIAFLCGSVLVWIGYDYSQQYSTWFSVTVGILLALGALGVFIVLLTEAHELAEA
VWTHKRRREFLPVEGDSDYRPKVSIHVPCYNEPPEMVKQTLDALAALDYPDYEVLIIDNNTKDPAVWEPVRDYCETLGPR
FKFFHVAPLAGFKGGALNYLIPHTAKDAEVIAVIDSDYCVSPNWLKHMVPHFADPKIAVVQSPQDYRDQNESTFKKLCYA
EYKGFFHIGMVTRNDRDAIIQHGTMTMTRRSVLEELGWADWCICEDAELGLRVFEKGLSAAYYHDSYGKGLMPDTFIDFK
KQRFRWAYGAIQIIKRHTASLLRGKGTELTRGQRYHFLAGWLPWVADGMNIFFTVGALLWSAAMIIVPTRVDPPLLIFAI
PPLALFVFKVGKIIFLYRRAVGVNLKDAFCAALAGLALSHTIAKAVLYGFFTTSIPFFRTPKNADNHGFWVAISEAREEM
FIMLLLWGAALGIYLVQGLPSNDIRFWVVMLLVQSLPYVA

>Pseudomonas_strain_PGPPP1_[Orphan] BLAST: A0A257DV11 Beta-(1-3)-glucosyl transferase
MASRKFGLNLVIVLAIAALFTGFWALINRPVTAPNWPEQISGFSYSPFQQGQFPQKDQYPTDDQMRRDLEIMSKLTDNIR
TYSVDGTLGDIPKLAEEFGLRVTLGIWISPDLERNEREIQRAIEIANSSRSVVRVVVGNEALFREEITPEALIVLLDRVR
AAVKVPVTTSEQWHIWEKNPQLAKHVDLIAAHILPFWEYIPMDKAGQYVLDRAKDLKKLFPKKPLLLSEVGWPSNGRMRG
GNETSPADQAIYLRTLVNKLNRQGYNYFVIEAFDQPWKVSDEGSAGAYWGVYNAARQQKFNFEGPVVAIPQWRVLAIGSV
VLALLSLTLLMIDGSALRQRGRTFLTFIAFLCGSVLVWIGYDYSQQYSTWFSVTVGILLALGALGVFIVLLTEAHELAEA
VWTHKRRREFLPVEGDSDYRPKVSIHVPCYNEPPEMVKQTLDALAALDYPDYEVLIIDNNTKDPAVWEPVRDYCETLGPR
FKFFHVAPLAGFKGGALNYLIPHTAKDAEVIAVIDSDYCVSPNWLKHMVPHFADPKIAVVQSPQDYRDQNESTFKKLCYA
EYKGFFHIGMVTRNDRDAIIQHGTMTMTRRSVLEELGWADWCICEDAELGLRVFEKGLSAAYYHDSYGKGLMPDTFIDFK
KQRFRWAYGAIQIIKRHTASLLRGKDTELTRGQRYHFLAGWLPWVADGMNIFFTVGALLWSAAMIIVPTRVDPPLLIFAI
PPLALFVFKVGKIIFLYRRAVGVNLKDAFCAALAGLALSHTIAKAVLYGFFTTSIPFFRTPKNADNHGFWVAISEAREEM
FIMLLLWGAALGIYLVQGLPSNDIRFWVVMLLVQSLPYVAALIMAFLSSLPKPAAEPEPAPAA

>Pseudomonas_strain_P7759_[Orphan] BLAST: A0A7Y7Z2D7 Glycosyltransferase
MASRKFGLNLVIVLAIAALFTGFWALINRPVTTPNWPEQISGFSYSPFQQGQYPQKDQYPTDDQMRRDLEIMSKLTDNIR
TYSVDGTLGDIPKLAEEFGLRVTLGIWISPDLERNEREIQRAIEIANSSRSVVRVVVGNEALFREEITPEALIVLLDRVR
AAVKVPVTTSEQWHIWEKNPQLAKHVDLIAAHILPFWEYIPMDKAGQYVLDRARDLKKLFPKKPLLLSEVGWPSNGRMRG
GNETSPADQAIYLRTLVNKLNRQGFNYFVIEAFDQPWKVSDEGSAGAYWGVYNAARQQKFNFEGPVVAIPQWRVLAIGSV
VLALLSLTLLMIDGSALRQRGRTFLTFIAFLCGSVLVWIGYDYSQQYSTWFSVTVGILLALGALGVFIVLLTEAHELAEA
VWTHKRRREFLPVEGDSDYRPKVSIHVPCYNEPPEMVKQTLDALAALDYPDYEVLIIDNNTKDPAVWEPVRDYCETLGPR
FKFFHVAPLAGFKGGALNYLIPHTAKDAEVIAVIDSDYCVSPNWLKHMVPHFADPKIAVVQSPQDYRDQNESTFKKLCYA
EYKGFFHIGMVTRNDRDAIIQHGTMTMTRRSVLEELGWADWCICEDAELGLRVFEKGLSAAYYHDSYGKGLMPDTFIDFK
KQRFRWAYGAIQIIKRHTASLLRGKDTELTRGQRYHFLAGWLPWVADGMNIFFTVGALLWSAAMIIVPTRVDPPLLIFAI

PPLALFVFKVGKIIFLYRRAVGVNLKDAFCAALAGLALSHTIAKAVLYGFFTTSIPFFRTPKNADNHGFWVAISEAREEM
FIMLLLWGAALGIYLVQGLPSNDIRFWVVMLLVQSLPYVAALIMAFLSSLPKPAAAPEPAPAA

>Pseudomonas_strain_QC2_[Orphan] BLAST: A0A2N5EYD7 Beta-(1-3)-glucosyl
transferase
MASRKFGLNLVIVLAIAALFSGFWALINRPVTAPNWPEQISGFSYSPFQQGQYPQKDQYPTDDQMRRDLEIMSKLTDNIR
TYSVDGTLGDIPKLAEEFGLRVTLGIWISPDLERNEREIQRAIEIANSSRSVVRVVVGNEALFREEITPEALIVLLDRVR
AAVKVPVTTSEQWHIWEKNPQLAKHVDLIAAHILPFWEYIPMDKAGQYVLDRARDLKKLFPKKPLLLSEVGWPSNGRMRG
GNETSPADQAIYLRTLVNKLNRQGFNYFVIEAFDQPWKVSDEGSAGAYWGVYNAARQQKFNFDGPVVAIPQWRVLAIGSV
VLALLSLTLLMIDGSSLRQRGRTFLTFIAFLCGSVLVWIGYDYSQQYSTWFSVTVGILLALGALGVFIVLLTEAHELAEA
VWTHKRRREFLPVEGDSDYRPKVSIHVPCYNEPPEMVKQTLDALAALDYPDYEVLIIDNNTKDPAVWEPVRDYCETLGPR
FKFFHVAPLAGFKGGALNYLIPHTAKDAEVIAVIDSDYCVSPNWLKHMVPHFADPKIAVVQSPQDYRDQNESTFKKLCYA
EYKGFFHIGMVTRNDRDAIIQHGTMTMTRRSVLEELGWADWCICEDAELGLRVFEKGLSAAYYHDSYGKGLMPDTFIDFK
KQRFRWAYGAIQIIKRHTASLLRGKGTELTRGQRYHFLAGWLPWVADGMNIFFTVGALLWSAAMIIVPTRVDPPLLIFAI
PPLALFVFKVGKIIFLYRRAVGVNLKDAFCAALAGLALSHTIAKAVLYGFFTTSIPFFRTPKNADNHGFWVAISEAREEM
FIMLLLWGAALGIYLVQGLPSNDIRFWVVMLLVQSLPYVAALVMAFLSSLPKPAPKVELATAE

>Pseudomonas_strain_RGB_[Orphan] BLAST: A0A558FZH0 Glycosyltransferase
MSSRKFGLNLVVVLAIAALFTGFWALINRPVTTPNWPEQISGFSYSPFQQGQFPQKDQYPSDEEMRRDLEIMSKLTDNIR
TYSVDGTLGDIPKLAEEFGLRVTLGIWISPDLARNEREIQRAIELANSSRSVVRVVVGNEALFREEITPEALIALLDRVR
AAVKVPVTTSEQWHIWEKNPQLAKHVDLIAAHILPFWEFIPMDKAGQYVLDRARDLKKLFPKKPLLLSEVGWPSNGRMRG
GNETSPADQAIYLRTLVNKLNRQGYNYFVIEAFDQPWKVSDEGSAGAYWGVYNAARQQKFNFEGPVVAIPQWRVLAIGSV
VLALLSLTLLMIDGSALRQRGRTFLTFIAFLCGSVLVWIGYDYSQQYSTWFSVTVGILLALGALGVFIVLLTEAHELAEA
VWTHKRRREFLPVEGDSDYRPKVSIHVPCYNEPPEMVKQTLDALAALDYPDYEVLIIDNNTKDPAVWEPVRDYCETLGPR
FKFFHVSPLAGFKGGALNYLIPHTAKDAEVIAVIDSDYCVSPNWLKHMVPHFADPKIAVVQSPQDYRDQNESTFKKLCYA
EYKGFFHIGMVTRNDRDAIIQHGTMTMTRRSVLEELGWADWCICEDAELGLRVFEKGLSAAYYHDSYGKGLMPDTFIDFK
KQRFRWAYGAIQIIKRHTASLLRGKGTELTRGQRYHFLAGWLPWVADGMNIFFTVGALLWSAAMIIVPTRVDPPLLIFAI
PPLALFVFKVGKIIFLYRRAVGVNLKDAFCAALAGLALSHTIAKAVLYGFFTTSIPFFRTPKNADNHGFWVAISEAREEM
FIMLLLWGAALGIYLVQGLPSNDMRFWVVMLLVQSLPYVAALIMAFLSSLPKPAPKVEPVTAE

>Pseudomonas_strain_R9.37_[Orphan] BLAST: A0A2P8LHR7 Beta-(1-3)-glucosyl
transferase
MSSRKFGLNLVVVLAIAALFTGFWALINRPVTTPNWPEQISGFSYSPFQQGQFPQKDQYPSDEEMRRDLEIMSKLTDNIR
TYSVDGTLGDIPKLAEEFGLRVTLGIWISPDLARNEREIQRAIELANSSRSVVRVVVGNEALFREEITPEALIALLDRVR
AAVKVPVTTSEQWHIWEKNPKLAKHVDLIAAHILPFWEFIPMDKAGQYVLDRARDLKKLFPKKPLLLSEVGWPSNGRMRG
GNETSPADQAIYLRTLVNKLNRQGYNYFVIEAFDQPWKVSDEGSAGAYWGVYNAARQQKFNFEGPVVAIPQWRVLAIGSV
VLALLSLTLLMIDGSALRQRGRTFLTFIAFLCGSVLVWIGYDYSQQYSTWFSVTVGILLALGALGVFIVLLTEAHELAEA
VWTHKRRREFLPVEGDSDYRPKVSIHVPCYNEPPEMVKQTLDALAALDYPDYEVLIIDNNTKDPAVWEPVRDYCETLGPR
FKFFHVSPLAGFKGGALNYLIPHTAKDAEVIAVIDSDYCVSPNWLKHMVPHFADPKIAVVQSPQDYRDQNESTFKKLCYA
EYKGFFHIGMVTRNDRDAIIQHGTMTMTRRSVLEELGWADWCICEDAELGLRVFEKGLSAAYYHDSYGKGLMPDTFIDFK
KQRFRWAYGAIQIIKRHTASLLRGKGTELTRGQRYHFLAGWLPWVADGMNIFFTVGALLWSAAMIIVPTRVDPPLLIFAI
PPLALFVFKVGKIIFLYRRAVGVNLKDAFCAALAGLALSHTIAKAVLYGFFTTSIPFFRTPKNADNHGFWVAISEAREEM
FIMLLLWGAALGIYLVQGLPSNDIRFWVVMLLVQSLPYVAALIMAFLSSLPKPAPKVEPATAE

>Pseudomonas_strain_S10E_269_[Orphan] BLAST: A0A2M9GA00 Beta-(1-3)-glucosyl
transferase
MASRKFGLNLVIVLAIAALFTGFWALINRPVTTPNWPEQISGFSYSPFQQGQYPQKGQYPTDDQMRRDLEIMSKLTDNIR
TYSVDGTLGDIPKLAEEFGLRVTLGIWISPDLERNEREIQRAIEIANSSRSVVRVVVGNEALFREEITPEALIVLLDRVR
AAVKVPVTTSEQWHIWEKNPQLAKHVDLIAAHILPFWEYIPMDKAGQYVLDRARDLKKMFPKKPLLLSEVGWPSNGRMRG
GNETSPSDQAIYLRTLVNKLNRQGFNYFVIEAFDQPWKVSDEGSAGAYWGVYNAARQQKFNFDGPVVAIPQWRVLAIGSV
VLALLSLTLLMIDGSALRQRGRTFLTFIAFLCGSVLVWIGYDYSQQYSTWFSVTVGILLALGALGVFIVLLTEAHELAEA
VWTHKRRREFLPVEGDSDYRPKVSIHVPCYNEPPEMVKQTLDALAALDYPDYEVLIIDNNTKDPAVWEPVRDYCETLGPR
FKFFHVAPLAGFKGGALNYLIPHTAKDAEVIAVIDSDYCVSPNWLKHMVPHFADPKIAVVQSPQDYRDQNESTFKKLCYA
EYKGFFHIGMVTRNDRDAIIQHGTMTMTRRSVLEELGWADWCICEDAELGLRVFEKGLSAAYYHDSYGKGLMPDTFIDFK
KQRFRWAYGAIQIIKRHTASLLRGKDTELTRGQRYHFLAGWLPWVADGMNIFFTVGALLWSAAMIIVPTRVDPPLLIFAI
PPLALFVFKVGKIIFLYRRAVGVNLKDAFCAALAGLALSHTIAKAVLYGFFTTSIPFFRTPKNADNHGFWVAISEAREEM
FIMLLLWGAALGIYLVQGLPSNDIRFWVVMLLVQSLPYVAALVMAFLSSLPKPAPKAEPATVE

>Pseudomonas_strain_WP001_[Orphan] BLAST: A0A2S4CB68 Beta-(1-3)-glucosyl
transferase

MASRKFGLNLVVVLAIAALFTGFWALINRPVTAPNWPEQISGFSYSPFQQGQYPQKDQYPTDEQMRQDLAIMSKLTDNIR
TYSVDGTLGDIPKLAEEFGLRVTLGIWISPDLERNEREIQRAIEIANTSRSVVRVVVGNEALFREEITPEALIVLLDRVR
AAVKVPVTTSEQWHIWEKNPQLAKHVDLIAAHILPFWEFIPMDKAGQYVLDRARDLKKLFPKKPLLLSEVGWPSNGRMRG
GNETSPADQAVYLRTLVNKLNRQGFNYFVIEAFDQPWKVSDEGSAGAYWGVFNAARQQKFNFEGPVVAIPQWRVLAIGSV
VLALLSLTLLMIDGSALRQRGRTFLTFIAFLCGSVLVWIGYDYSQQYSTWFSVTVGILLALGALGVFIVLLTEAHELAEA
VWTHKRRREFLPVEGESDYRPKVSIHVPCYNEPPEMVKQTLDALAALDYPDYEVLIIDNNTKDPAVWEPVRDYCQTLGPR
FKFFHVAPLAGFKGGALNYLIPHTAKDAEVIAVIDSDYCVSPNWLKHMVPHFADPKIAVVQSPQDYRDQNESTFKKLCYA
EYKGFFHIGMVTRNDRDAIIQHGTMTMTRRSVLEELGWADWCICEDAELGLRVFEKGLSAAYYHDSYGKGLMPDTFIDFK
KQRFRWAYGAIQIIKRHTASLLRGKGTELTRGQRYHFLAGWLPWVADGMNIFFTVGALLWSAAMIIVPTRVDPPLLIFAI
PPLALFVFKVGKIIFLYRRAVGVNLKDAFCAALAGLALSHTIAKAVLYGFFTTSIPFFRTPKNADNHGFWVAISEAREEM
FIMLLLWGAALGIYLVQGLPSNDIRFWVVMLLVQSLPYVAALIMAFLSSLPKPAPKAEPVTAV

>Pseudomonas_strain_WS_5532_[Orphan] BLAST: A0A7Y0VZZ7 Glycosyltransferase
MSSRKFGLNLVVVLAIAALFTGFWALINRPVTTPNWPEQISGFSYSPFQQGQFPQKDQYPTDDQMRQDLAIMSKLTDNIR
TYSVDGTLGDIPKLAEEFGLRVTLGIWISPDLERNEREIQRAIEIANNSRSVVRVVVGNEALFREEITPEALIVLLDRVR
AAVKVPVTTSEQWHIWEKNPQLAKHVDLIAAHILPFWEFIPMDKAGQYVLDRARDLKKLFPKKPLLLSEVGWPSNGRMRG
GNESSPADQAIYLRTLVNKLNRQGYNYFVIEAFDQPWKVSDEGSAGAYWGVYNAARQQKFNFEGPVVAIPQWRVLAIGSV
VLALLSLTLLMIDGSALRQRGRTFLTFIAFLCGSVLVWIGYDYSQQYSTWFSVTVGILLALGALGVFIVLLTEAHELAEA
VWTHKRRREFLPVEGDSDYRPKVSIHVPCYNEPPEMVKQTLDALAALDYPDFEVLIIDNNTKDPAVWEPVRDYCETLGPR
FKFFHVSPLAGFKGGALNYLIPHTAKDAEVIAVIDSDYCVSPNWLKHMVPHFADPKIAVVQSPQDYRDQNESTFKKLCYA
EYKGFFHIGMVTRNDRDAIIQHGTMTMTRRSVLEELGWADWCICEDAELGLRVFEKGLSAAYYHDSYGKGLMPDTFIDFK
KQRFRWAYGAIQIIKRHTALLRGKGTELTRGQRYHFLAGWLPWVADGMNIFFTVGALLWSAAMIIVPTRVDPPLLIFAIP
PLALFVFKVGKIIFLYRRAVGVNLKDAFCAALAGLALSHTIAKAVLYGFFTTSIPFFRTPKNADNHGFWVAISEAREEMF
IMLLLWGAALGIYLVQGLPSNDIRFWVVMLLVQSLPYVAALIMAFLSSLPKPAPKVEPATAE

>Pseudomonas_strain_44_R_15_[Orphan] BLAST: A0A1B5DKC0 Cellulose synthase catalytic subunit [UDP-forming] but changed in this work
MASRKFGLNLVVVLAIAALFTGFWALINRPVTAPNWPEQISGFSYSPFQQGQYPQKDQYPTDEQMRQDLAIMSKLTDNIR
TYSVDGTLGDIPKLAEEFGLRVTLGIWISPDLERNEREIQRAIEIANTSRSVVRVVVGNEALFREEITPEALIVLLDRVR
AAVKVPVTTSEQWHIWEKNPQLAKHVDLIAAHILPFWEFIPMDKAGQYVLDRARDLKKLFPKKPLLLSEVGWPSNGRMRG
GNETSPADQAVYLRTLVNKLNRQGFNYFVIEAFDQPWKVSDEGSAGAYWGVFNAARQQKFNFEGPVVAIPQWRVLAIGSV
VLALLSLTLLMIDGSALRQRGRTFLTFIAFLCGSVLVWIGYDYSQQYSTWFSVTVGILLALGALGVFIVLLTEAHELAEA
VWTHKRRREFLPVEGESDYRPKVSIHVPCYNEPPEMVKQTLDALAALDYPDYEVLIIDNNTKDPAVWEPVRDYCQTLGPR
FKFFHVAPLAGFKGGALNYLIPHTAKDAEVIAVIDSDYCVSPNWLKHMVPHFADPKIAVVQSPQDYRDQNESTFKKLCYA
EYKGFFHIGMVTRNDRDAIIQHGTMTMTRRSVLEELGWADWCICEDAELGLRVFEKGLSAAYYHDSYGKGLMPDTFIDFK
KQRFRWAYGAIQIIKRHTASLLRGKGTELTRGQRYHFLAGWLPWVADGMNIFFTVGALLWSAAMIIVPTRVDPPLLIFAI
PPLALFVFKVGKIIFLYRRAVGVNLKDAFCAALAGLALSHTIAKAVLYGFFTTSIPFFRTPKNADNHGFWVAISEAREEM
FIMLLLWGAALGIYLVQGLPSNDIRFWVVMLLVQSLPYVAALIMAFLSSLPKPAPKAEPVTAV

>Pseudomonas_strain_58_R_3_[Orphan] BLAST: A0A1B5D768 Cellulose synthase catalytic subunit [UDP-forming] but changed in this work
MSSRKFGLNLVVVLAIAALFTGFWALINRPVTTPNWPEQISGFSYSPFQQGQFPQKDQYPTDDQMRQDLAIMSKLTDNIR
TYSVDGTLGDIPKLAEEFGLRVTLGIWISPDLERNEREIQRAIEIANTSRSVVRVVVGNEALFREEITPEALIVLLDRVR
AAVKVPVTTSEQWHIWEKNPQLAKHVDLIAAHILPFWEFIPMDKAGQYVLDRARDLKKLFPKKPLLLSEVGWPSNGRMRG
GNESSPADQAIYLRTLVNKLNRQGYNYFVIEAFDQPWKVSDEGSAGAYWGVYNAARQQKFNFEGPVVAIPQWRVLAIGSV
VLALLSLTLLMIDGSALRQRGRTFLTFIAFLCGSVLVWIGYDYSQQYSTWFSVTVGILLALGALGVFIVLLTEAHELAEA
VWTHKRRREFLPVEGDSHYRPKVSIHVPCYNEPPEMVKQTLDALAALDYPDFEVLIIDNNTKDPAVWEPVRDYCETLGPR
FKFFHVSPLAGFKGGALNYLIPHTAKDAEVIAVIDSDYCVSPNWLKHMVPHFADPKIAVVQSPQDYRDQNESTFKKLCYA
EYKGFFHIGMVTRNDRDAIIQHGTMTMTRRSVLEELGWADWCICEDAELGLRVFEKGLSAAYYHDSYGKGLMPDTFIDFK
KQRFRWAYGAIQIIKRHTASLLRGKGTELTRGQRYHFLAGWLPWVADGMNIFFTVGALLWSAAMIIVPTRVDPPLLIFAI
PPLALFVFKVGKIIFLYRRAVGVNLKDAFCAALAGLALSHTIAKAVLYGFFTTSIPFFRTPKNADNHGFWVAISEAREEM
FIMLLLWGAALGIYLVQGLPSNDMRFWVVMLLVQSLPYVAALIMAFLSSLPKPAPKVEPATAE

>Pseudomonas_strain_2995_[Orphan] BLAST: A0A2G5NL94 Beta-(1-3)-glucosyl transferase
MSSRKFGLNLVVVLAIAALFTGFWALINRPVTTPNWPEQISGFSYSPFQQGQFPQKDQYPTDDQMRQDLAIMSKLTDNIR
TYSVDGTLGDIPKLAEEFGLRVTLGIWISPDLERNEREIQRAIEIANNSRSVVRVVVGNEALFREEITPEALIVLLDRVR
AAVKVPVTTSEQWHIWEKNPQLAKHVDLIAAHILPFWEFIPMDKAGQYVLDRARDLKKLFPKKPLLLSEVGWPSNGRMRG
GNESSPADQAIYLRTLVNKLNRQGYNYFVIEAFDQPWKVSDEGSAGAYWGVYNAARQQKFNFEGPVVAIPQWRVLAIGSV
VLALLSLTLLMIDGSALRQRGRTFLTFIAFLCGSVLVWIGYDYSQQYSTWFSVTVGILLALGALGVFIVLLTEAHELAEA
VWTHKRRREFLPVEGDSDYRPKVSIHVPCYNEPPEMVKQTLDALAALDYPDFEVLIIDNNTKDPAVWEPVRDYCETLGPR

FKFFHVSPLAGFKGGALNYLIPHTAKDAEVIAVIDSDYCVSPNWLKHMVPHFADPKIAVVQSPQDYRDQNESTFKKLCYA
EYKGFFHIGMVTRNDRDAIIQHGTMTMTRRSVLEELGWADWCICEDAELGLRVFEKGLSAAYYHDSYGKGLMPDTFIDFK
KQRFRWAYGAIQIIKRHTASLLRGKGTELTRGQRYHFLAGWLPWVADGMNIFFTVGALLWSAAMIIVPTRVDPPLLIFAI
PPLALFVFKVGKIIFLYRRAVGVNLKDAFCAALAGLALSHTIAKAVLYGFFTTSIPFFRTPKNADNHGFWVAISEAREEM
FIMLLLWGAALGIYLVQGLPSNDIRFWVVMLLVQSLPYVAALIMAFLSSLPKPAPKVEPATAE

>Pseudomonas_synxantha_BG33R_[Orphan] PseudoCAP: Beta-(1-3)-glucosyl transferase
MASRKFGLNLVIVLAIAALFTGFWALINRPVTAPNWPEQISGFSYSPFQQGQYPQKNQFPTEEQMRQDLAIMSKLTDNIR
TYSVDGSLGEIPKLAEEFGLRVTLGIWISPDLERNEREIQRAIEIANNSRSVVRVVVGNEALFRDEITPEALIVLLDRVR
AAVKVPVTTSEQWHIWEKNPQLAKHVDLIAAHILPFWEYIPMDKAGQYVLDRAKDLKKAFPKKPLLLSEVGWPSNGRMRG
GNESSPADQAIYLRTLVNKLNRQGYNYFVIEAFDQPWKVSDEGSAGAYWGVFNAARQQKFNFEGPVVAIPQWRVLAIGSV
VLALLSLTLLMIDGSALRQRGRTFLTFIAFLCGSVLVWIGYDYSQQYSTWFSLIVGVLLALGALGVFIVLLTEAHELAEA
VWTHKRRREFLPVEGDSDYRPKVSIHVPCYNEPPEMVKQTLDALAALDYPDYEVLIIDNNTKDPAVWEPVRDYCETLGPR
FKFFHVAPLAGFKGGALNYLIPHTAKDAEVIAVIDSDYCVSPNWLKHMVPHFADPKIAVVQSPQDYRDQNESTFKKLCYA
EYKGFFHIGMVTRNDRDAIIQHGTMTMTRRSVLEELGWADWCICEDAELGLRVFEKGLSAAYYHDSYGKGLMPDTFIDFK
KQRFRWAYGAIQIIKRHTASLIRGKDTELTRGQRYHFLAGWLPWVADGMNIFFTVGALLWSAAMIIVPTRVDPPLLIFAI
PPLALFVFKVGKIIFLYRRAVGVNLKDAFCAALAGLALSHTIAKAVLYGFFTTSIPFFRTPKNADNHGFWVAISEAREEM
FIMLLLWGAALGIYLVQGLPSNDIRFWVVMLLVQSLPYVAALIMAFLSSLPKPAPKAEPATAV

>Pseudomonas_syringae_B728a_[Orphan] PseudoCAP: Glycosyl transferase family
protein
MDENSNNTGTWPTNLFKTRLNYPPNWPAMISGVAYAPFRPGQSPYKQIFPTRDQIREDLLLLRSITRNIRTYSVEGTLMH
IPELAEALGMNVTLGVWITEDETHNIEEINAGIELANRYSSVQRLVLGNEVLFRDDVPIDLLIHYLQTARRAVNVPVSTS
EIWTQWYETPDLVRHVDFIAAHILPFWEGVSALDATAITLAHANELRTRFPDTPLILSEIGWPSKAIAKRRMTTSDAEHS
IYLRNQIPLLDQHGHDYFVIEAFDQHWKTEEGLPGPNWGLFDAKRRLKLHVNGPVKMPVNMLSEILRLITRLKPESWPAG
ALIIVLAYCALSGFGMHYSQPLPAWLALPVVFIWAASVLTYMGIETHEFLEACWGPDTPRSFSPVHRPVGPAPKVSLHVP
CYNEPPDMVKRTLDSLQTLDYPDFEVLVIDNNTQDPAIWKPIERYCQQLGPRFRFFHVSPLPGFKAGALNYLLRHTAEDA
EVVAVIDADYCVHRQWLKHMVPHFTDPKVAVIQSPQDYRDGHESLFKCCCQAEYRGFFNIGMVIRNDHDAIIQHGTMTLI
RRSALDRLGWAEWCICEDAELGLRMLESGFSTGYAAISYGKGLTPDTFMDFKKQRYRWAYGAMQIVKRHAGSLIAGNCAS
LSAMQRYHFIAGWMPWVAEGMNYLLTLAALAWSMAMILKPETFGPLPWIFSTSLILMFALRSFKMIVLYRRLVSTHIKEA
LAAILAGMALYPTLGKAVLAGLFTSAMPFYRTPKHTSANRIGQNLLDVREELSTLAISWIAIVLLLTGRASIDTNSGFWI
TMLFAQSLPYLAAITMAILSARANRPALPTT

>Pseudomonas_syringae_DC3000_[BcsA] PseudoCAP: Cellulose synthase catalytic
subunit [UDP-forming]
MTNLSLDASPSRTRSSLWLNALSERFGQQSRTLRRALKTVAIVVGLLLMALVVTVPLDLYAQCFFALACFAAMLVIRKIP
GRISVLALVTLSLLASLRYMYWRLTSTLDFDNWLDSLLGYGLIVAEFYTLIVIVLGYVQTAWPLHRKPVIMPSDSSQWPT
VDVFIPSYNEALSIVKLTIFAAQSIDWPRDKLRVYVLDDGRREDFREFCEQVGVGYLTRENNYHAKAGNLNEALKSTDGE
YIAMFDADHVPTRSFLQVAMGWFLKDSKLAMLQTPHFFFSPDPFEKNLDTFRSVPNEGELFYGLLQDGNDLWNATFFCGS
CAVLRRSSLLEIGGVATETVTEDAHTALKLNRAGYNTAYLAIPQAAGLATESLSRHVAQRIRWARGMAQIFRTDNPLLGK
GLSIGQRLCYANSMLHFFYGLPRLVFLTAPLAYLLFGAEVMHASALMITAYVLPHLAHASLTNSRIQGRFRHSFWNEVYE
AVLAWYIMGPVLMALINPKFGGFNVTDKGGVVEEKFFDWTLARPYIVLLTLNAVVFALGIYSLYQLGWNNDAITLTIIIN
MAWTIYNIIITSAAIAVASEIRQVRTEPRVQARLPIRVTRADGVVFNAVTQDFSQTGLGLVLPVDAGIDSGDSITVSLYR
GAQTSHFPATVMFCRDGYLGTRFDDLSLRQQSELVRLTFGRADTWASTWGHGKPDTPLAALREVSHVGVRGVVELLKATR
KDFGRLLPTRKKTSPPPAN

>Pseudomonas_syringae_DC3000_[Orphan] PseudoCAP: Glycosyl transferase family
protein
MSIYRMEHSLDMNKKISDAPIWPVNSFKSVVTKVPDWPDSISGLAYNPFRPGQSPYKHIYPTREQIKEDLLLIRPLTRHV
RTYSVEQTLACIPEIAEELGMSVTLGIWIGWDEKRNDRELIEGVKLANQYPSVRRLIIGNETLLRNDVTVSQLIDYMQTA
RQGVNVPISTSEGWQQWHDTPELADHADFIAAHVLPFREFVPVTQAGSAVLARANELRLMFPEKPLILSEIGWPDKGNFR
RRTTAYVAEQSIYLRSQLALLNQSGLDYFVREAFDQQWKTEEGLPGPHWGLFDAQRKIKLPLQGPVKIRASWRSEVPRLV
ADWQPDNWRTTVLIFAALYTLLVGVGISYAQPLSMWVALPIALVWVTSLLIGTGIQGYEFLESCWGPEKPRSFPPLRAYP
GPLPKVSIHVPCYNEPPDMVKLTLDALQRLDYPNFEVLIIDNNTQDPEVWEPIEQYCRQLGPRFRLFHVNPLSGFKSGAL
NYLLDYTAKDAEIVAAIDADYCVHRHWLKHMAPYFACPDIAVIQVPQDYRDGDDSLFKRCCQAEYRVFFNIGMVIRNDHD
AIIQHGTMTLIRNSVLQRLRWAEWSICEDAELGLRILENGFSTGYVAISYGKGLIPDTFMDFKKQRYRWAYGVIQILKRH
TGSLIAGTCEALTPIQRYHFIAGWMPWIAGGINYFLAIAVLLWSMAMIIQPDTLEPVPWIFSSSLLLMFVLGVCKAISLY
QRLASTDIKDAFAAIIASMALYSVVGKAVLSSAFTSGLPFFRTPKQTSGSGLGKALLDVREDLYMAVVWWVMTVSLCFRK
EAIGPDLGFWVAIMFAQSLPYVAAMIMAILSALANRPSRSTT

>Pseudomonas_syringae_ICMP_9617_[Orphan] PseudoCAP: Beta-(1-3)-glucosyl transferase
MNHTATINFNRLKDGFSPKKLFQRRLIEVPDWPASISGFSYAPFRPGQSARKKIYPTREQIKEDLLLIKPFTQNIRTYSV
EGTLAYIPEIAEELGMSVSLGAWISQDKARNAQELKTAIEITNRFSCVQRLMVGNEVLFRGDLSPEQLIEHIKTARHSVK
VPVATSDTWMQWLEAPELVEHSDFIAAHILPFWERFSAEEAASIVINQARQLQQEFPDKTLILSEIGWPSQGNATRRAST
TAAEQSIYLRTQISVLAQLDCPYFVIEAFDQPWKTGEGTPGPHWGFFNSQRKLKLQLYGPVNAPIRWRSTLLNSVIRLRP
DSSRVTLAVTVALICALIILALEYSQSLPLWITMPVSVLWATCLLAGIAIESHEFLEAIWGPEQPRMFLPARFKYDQAPK
VSLHVPCYDEPPDMVKRTLDALQNLDYPNFEVLVIDNNTPDRATWEPVEQHCQRLGSRFKFFHVSPLAGFKAGALNYLIG
QTAPDAEIVAVIDADYCVNRLWLKHMVPHFANPKIGIIQVPQDYSDGDKNLFKYCCHAEYKGFFEVGMVIRNDHDAIIQH
GTMTLIRRSALDRLGWAQWCICEDAELGLRMLENGFSTGYTPLSYGKGLTPDTFNDFKKQRFRWAYGAVQIVKQHSWSLI
AGRSEALSTMQRYHFLAGWVPWAAEGVNYLLVFATLLWSAAMILRPEMLYPVPWIFSTSLLLMFTLRIVKVFFLYQQRVG
SDVTEAMAAILAGMALYPTIGRAVLSGLFTSGLPFFRTPKQSSCNSFRQTIIEARQDFYVVMISLVAIVLLYIRKDTVDP
DLGFWIAMLVAQSLPYLAAITMAILSARATRPALSTA

>Pseudomonas_syringae_NCPPB_4273_[Orphan] PseudoCAP: Beta-(1-3)-glucosyl transferase
MEHIADMDDNLSKRNIWPAHSLTTMASEVPDWPENIPGVSYSPFRPGQSPYTHLYPTREQITEDLLLIRPFTRHIRTYSV
EGTLACIPEIAEALGMSVTLGVWITWDEKHNDRDLLTGVDLANRFSSVQRLLIGHEALLRNDVTVDQLIAYMSTARRYVD
VPVSTSEGWKQWHETPELAEHADFIAAHILPFKEAVPVTEASARVLARADELKLMFPDKPLIISEVGWPGKGNFRRRITT
YRAEQSIYLRHQLALLEQHGHDYFVMEAFDQPWKTSEGLPGPHWGLFDAQRKMKLQLKGPVKTRASWQSEIPRVIAGLRP
DAWRTTAVMCAVAYAVLVWVGMSYAQPLSAWIALPIALAWATGIMIVVGTQGYEFLESFWGAENPRSFPPTRAYPGPWPK
VSIHVPCYNEPPDMVKLTLDALQKLDYPDFEVLVIDNNTQDPNVWKPVQAYCRQLGAHFRFFHVNPLSGFKAGALNYLLE
YTAEDAGIVAAIDADYCVHRHWLKHMVSHFADPDIAVIQVPQDYRDGDESLFKRYCEAEYRVFFNIGMVIRNDHDAIIQH
GTMTLIRKSVLQRLRWAEWCICEDAELGLRILEHGFSTGYAAISYGKGLMPDTFMDFKKQRYRWTYGAMQIFKRHAASLL
AGTGTALTPVQRYYFIAGWVPWMAGGVNYFLALAVLLWSMAMIVEPDLLEPVPWLFSASLLLMFVLGIFKAFTLYQRLAN
TDIKDALAAMLASTALYSVIGKAVLSALFTSGLPFFRTPKQTARTRFGQAVREATEDVCMIVLWWAAIILLCIRKETIDP
DLGFWIAMLFAQSVPYLAAITMTILSARATRPAPSTA

>Pseudomonas_syringae_UMAF0158_[Orphan] PseudoCAP: Beta-(1-3)-glucosyl transferase
MEHIADMDEYSNKAGTWLKNLFKTRFNYPPNWPAMISGVAYAPFRPGQSPYKQIFPTRDQIREDLLRLRSITQNIRTYSV
GGTLMHIPELAEALGMNVTLGVWITEDETHNIEEINAGIELANRYSSVQRLVLGNEVLFRDDVPIDLLILYLQTARRAVN
VPVSTSEIWTQWYETPELVRHVDFIAAHILPFWEGVSALDATAITLAHANELRTRFPDTPLILSEIGWPSKAIAKRRMTT
SDAEHSIYLRNQIPLLDQHDHDYFVIEAFDQHWKTEEGLPGPNWGLFDAKRRLKLHINGPVKIPVNMLSEILRLITRLKP
ESWPTGALIIVLAYCVLSGFGMHYSQPLPAWLALPVAFIAASVLTYVGIETHEFLEACWGPDTPRSFRPVRRPVGPAPKV
SLHVPCCNEPPDMVKRTLDSLQKLDYPDFEVLVIDNNTQDPAIWKPIERYCQQLGPRFQFFHVSPLPGFKAGALNYLLRH
TAEDAEVVAVIDADYCVHRQWLKHMVPHFTDPKVAVIQSPQDYRDGHESLFKYCCQAEQGFFNIGMVIRNDHDAIIQHGT
MTLIRRSALDRLGWAEWCICEDAELGLRMLESGFSTGYAAISYGKGLTPDTFMDFKKQRYRWAYGAMQIVKRHAGSLIAG
NCASLSAMQRYHFIAGWMPWVAEGMNYLLTLAALAWSMAMILKPETFGPLPWIFSTSLILMFALRSLKMIVLYRQLVSTH
TKEALAAILAGMALYPTLGKAVLAGLFTSAMPFYRTPKHTSANRIGQNLLDVREELSTLAISWIAIVLLLTGRASIDTNS
GFWITMLFAQSLPYLAAITMAILSARATRPARSTT

>Pseudomonas_syringae_41a_[Orphan] PseudoCAP: Beta-(1-3)-glucosyl transferase
MDDNLSKGNIWPARSLTTMASEVPDWPENIPGVSYSPFRPGQSPYTHLYPTREQITEDLLLIRPLTRHIRTYSVEGTLAC
IPEIAEALGMSVTLGVWITWDEKHNDRDLLTGVDLANRFSGVQRLLIGHEALLRNDVTVDQLIAYMNTARRYVEVPVSTS
EGWQQWHETPELAEHADFIAAHILPFKEAVPVTEASALVLARADELKLMFPDKPLIISEVGWPGKGNFRRRITTYRAEQS
IYLRHQLSLLEQHGHDYFVMEAFDQPWKTSEGLPGPHWGLFDARRNMKLQLKGPVKTRASWQSEIPRIIAGLQPDAWRTT
AVMCAVAYAVLVWVGMSYAQPLSAWIALPIALAWATCLMIVVVTQGYEFLESFWGAENPRSFPPTRAYPGPWPKVSIHVP
CYNEPPDMVKLTLDALQKLDYPDFEVLVIDNNTQDPNVWKPIQAYCRQLGARFRFLHVNRLSGFKAGALNYLLEYTAEDA
GIVAAIDADYCAHRHWLKHMVSHFADPDIAVIQVPQDYRDGDESLFKRYCEAEYRVFFNIGMVIRNDHDAIIQHGTMTLI
RKSVLQRLRWAEWCICEDAELGLRILEHGFSTGYAAISYGKGLMPDTFMDFKKQRYRWTYGAMQIFKRHAGSLLAGTGTA
LTPVQRYYFIAGWVPWMAGGVNYFLALAVLLWSMAMIVEPDLLEPVPWLFSASLLLMFVLGIFKAFTLYQRLANTDIKDA
LAAMLASTALYSVIGKAVLSALFTSGLPFVRTPKQTAHTRFGQAVREAAEDVCMILLWWAAIILLCIRKETIDPDLGFWI
TMLFAQSVPYLAAITMAVLSARATRPAPSTA

>Pseudomonas_taeanensis_MS-3_[Orphan] PseudoCAP: Beta-(1-3)-glucosyl transferase
MPSRKFGFNLVMVLAVAALFTGIWALYNRPVSAPDWPEQISGYSFSPFRQGQDPQNNSYPSDDEIRADLELLTQQTDNIR
TYSVDGSLADIPRLAEEFGLRVTLGIWISPDSARNEREIAKAIELANSSRSVVRVVVGNEALFRREVTAQQLSDYLDRVR
AAVKVPVTTSEQWHIWEQNPELVKHVDLIAAHILPYWEFMPMEQSTEFVLERAKELKRLFPKKPLLLSEVGWPSNGRMRG
GAEATPADQALYLRTLLNTLNAKGYSYFVIEAFDQPWKATDEGSVGAYWGVYNLDRQAKFAFEGPVVAIPQWRLLAIASV
VMALLALALLLIDGSALRQRGRTFLTFVAFAGGSVLVWIGYDYSQQYSTWFSATVGLLLGIGALGVFIVLLTEAHELAET

VWTSSRRRPFQPVLADSAYRPKVSIHVPCYNEPAEMVKQTLDALANLDYPDFEVLIIDNNTKDPAVWQPVQAYCEILGPR
FRFFHVAPLAGFKGGALNYILPHTAKDAEVIAVIDSDYCVDRNWLKHMVPHFADPKIAVVQSPQDYRDDQESTFKKLCYA
EYKGFFHIGMVTRNDRNAIIQHGTMTMIRRSVMDELQWADWTICEDAELGLRVFKKGYAAAYAHDSFGKGLMPDTFIDFK
KQRFRWAYGAIQIMKGHARSLFLGQGSELNRGQRYHFIAGWLPWVADGLNIFFTVGALLWSAAMIIVPQRVDPPLLIFAI
PPLALFVFKVAKIVFLYQRAVGVNLKKAFCAALAGLSLSHTIAKAVLYGFFTTSIPFFRTPKMASNHGIRVALMEAREEV
FIMLLLWGAAAGITVVQGMPSTDVMFWVAMLLVQSLPYLAALIMALLSSLPKPQVKTTDEPIAV

>Pseudomonas_taetrolens_DSM_21104_[Orphan] PseudoCAP: Beta-(1-3)-glucosyl
transferase
MSSRKFGLNLVVVLAIAALFTGFWALINRPVSAPDWPDQISGFSYSPFQQGQYPQKDQFPSDDEMRRDLEILSKLTDNIR
TYSVDGTLGDIPKLAEEFGLRVTLGIWISPDQERNEREIQKAIELANNSRSIVRVVVGNEALFREEITPEELIVLLDRVR
AAVKVPVTTSEQWHIWEKYPQLASHVDLIAAHILPYWEFIPMDQAGEFVLDRARDLKKIFPKKPLLLSEVGWPSNGRMRG
GADATPADQAVYLRTLVNTLNRRGYNYFVIEAFDQPWKASDEGSVGAYWGVYNAARQQKFNFEGPVVAIPQWRVLAIGSV
VLALLSLALLLIDGSALRQRGRTFLTFTAFLCGSVLVWIGYDYSQQYSTWFSLTVGFLLGLGALGVFIVLLTEAHELAEA
VWIRKRRREFLPVESDDAYRPKVSIHVPCYNEPPEMVKQTLNALANLDYPDFEVLLIDNNTKDPAVWEPVRDYCATLGPR
FKFFHVAPLAGFKGGALNYLIPHTAPDAEVIAVIDSDYCVDPNWLKHMVPHFADPKIAIVQSPQDYRDQSESTFKKLCYS
EYKGFFHIGMVTRNDRDAIIQHGTMTMTRRSVLEELGWADWCICEDAELGLRVFEKGYSAAYSHNSYGKGLMPDTFIDFK
KQRFRWAYGAIQIIKRHTSSLLRGKDTHLTRGQRYHFLAGWLPWVADGMNIFFTVGALLSAAMIIVPTRVDPPLLIFAIP
PLALFVFKVGKIIFLYRRAVGVDLKDAFAAALAGLALSHTIAKAVLYGFFTSSIPFFRTPKNADNHGFWVALSEAREEVF
IMLLLWGAALGIFFVQGLPSNDMRFWVVMLLVQSLPYLAALIMAFMSSLPKPVEATEPQPAA

>Pseudomonas_taiwanensis_DSM_21245_[Orphan] PseudoCAP: Glycosyl transferase
family protein
MSSRKFGLNLVVVLAIAALFTGFWALINRPVTAPAWPEQISGLSYSPFRLGESPQKGQYPSDDEIRQDLEQMSKLTDSIR
IYTVEGTQADIPRLAEELGLRVTLGIWISPDLERNEREIATAIELANKSRSVVRVVVGNEALFREEVTPEALIQYLDRVR
AAVKVPVTTSEQWHIWKEHPELAKHVDLIAAHILPYWEFVPMKDSVEFVLDRARELKQQFPRKPLLLSEVGWPSNGRMRG
GADATQADQAIYLRTLVNTLNRRGYNYFVIEAYDQPWKASDEGSVGAYWGVFNAERQQKFNFDGPVVAIPQWRALAVASV
VLAMIALTVLLIDGSALRQRGRTFLTFITFLCGSVLVWIAYDYSQQYSTWFSLTVGVLLALGALGVFIVLLTEAHELAEA
VWIHKRRREFLPVQADTAYRPKVSVHVPCYNEPPDMVKQTLDALAALDYPDYEVLVIDNNTKDPAVWEPLKAHCEKLGER
FKFFHVAPLAGFKGGALNYIPHTAKDAEVIAVIDSDYCVDRNWLKHMVPHFADPKIAVVQSPQDYRDQHESAFKKLCYSE
YKGFFHIGMVTRNDRDAIIQHGTMTMTRRSVLEELGWAEWCICEDAELGLRVFEKGLSAAYAHNSYGKGLMPDTFIDFKK
QRFRWAYGAIQIIKHHAGALLRGKGSELTRGQRYHFLAGWLPWIADGMNIFFTVGALLWSAAMIIVPHRVDPPLMIFAIP
PLALFFFKVGKIIFLYRRAVGVNLKDAFAAALAGLALSHTIAKAVLYGFFTSSMPFFRTPKNADSHGLMVAISEAREEL
FIMLLLWGAAAGIYLVQGLPSSDMRFWVAMLLVQSLPYLAALVMAFLSSLPKPADKLAEPQEA

>Pseudomonas_thermotolerans_J53_[Orphan] PseudoCAP: Beta-(1-3)-glucosyl
transferase
MPSRKFGLNLVVFVAVAALFTGLWALVNRPVSAPDWPEQISGFSFSPFRLGQNPQKNQYPSEAQILEDLELVSRQTESIR
TYSVEGSLAEIPRLAEEFGLRVTLGIWISPDQARNEREIAKAIEIANTTRSVVRVVVGNEALFRREISVEQLIHYLDRVR
SAVKVPVTTSEQWHIWQEHPELADHVDLIAAHVLPYWEFVPMEEATQFVLDRARDLRRQFPKKPLLLSEVGWPSNGRMRG
GAEASQADQAIYLRTLLHELNAKGYSYFVIEAFDQPWKAAEEGSVGAYWGVYNAERQPKFAFEGPIVAIPQWRLLAVLSV
VLGLLSLTLLLIDSSSLRQHGRTFLSFVAFAGGSALVWIAYDYSQQYSTWFSLTVGVLLGFGALGVFIVLLTEAHEWAET
VWVRKRRRLFLPVLADEAYRPKVSIHVPCYNEPPEMLKQTLDALANLDYPDYEVLIIDNNTKDPAVWEPVRDYCEKLGPR
FRFFHVAPLAGFKGGALNYILPHTAPDAEVVAVIDSDYCVEPNWLKCMVPHFADPKIAVVQSPQDYRDGEQSTFKKLCYA
EYKGFFHIGMVTRNDRNAIIQHGTMTMIRRSVMDQLKWADWTICEDAELGLRVFEQGYQAAYARQSFGRGLMPDTFIDYK
KQRFRWAYGAIQIMKGHARSLFLGKGSQLTLGQRYHFIAGWLPWIADGLNVFFTIGALLWSAAMIIVPQRVDPPLMIFAI
PPLALFFFKFGKIMYLYRKAVGVDLKRSFQAAIAGLALSHTIAKAVIYGAFTRTIPFFRTPKMASSSGLLVALAEAREEV
FIMLLLWGAALGIGIVQGFPSNDVKFWVATLLVQSLPYLAALVMALLSSMPQAQEAPQGDEVPA

>Pseudomonas_trivialis_IHBB745_[Orphan] PseudoCAP: Beta-(1-3)-glucosyl
transferase
MASRKFGLNLVIVLAIAALFTGFWALINRPVTAPNWPEQISGFSYSPFQQGQYPQKNQFPTDDQMRRDLEIMSKLTDNIR
TYSVDGTLGDIPKLAEEFGLRVTLGIWISPDLERNEREIQRAIEIANTSRSVVRVVVGNEAVFRKDITPEALIVLLDRVR
AAVKVPVTTSEQWDIWEKNPQLAKHVDLIAAHILPFWEYIPMDKAGQYVLDRAKDLKKLFPKKPLLLSEVGWPSNGRMRG
GNETSPADQAIYLRTLVNKLNRQGYNYFVIEAFDQPWKVSDEGSAGAYWGVFNGARQQKFNFEGPVVAIPQWRVLAIGSV
VLALLSLTLLMIDGSALRQRGRTFLTFIAFLCGSVLVWIGYDYSQQYSTWFSVTVGILLALGALGVFIVLLTEAHELAEA
VWTHKRRREFLPVEGESDYRPKVSIHVPCYNEPPDMVKQTLDALAALDYPDYEVLIIDNNTKDPAVWEPVRDYCETLGPR
FKFFHVAPLAGFKGGALNYLIPHTAKDAEVIAVIDSDYCVSPNWLKHMVPHFADPKIAVVQSPQDYRDQNESTFKKLCYA
EYKGFFHIGMVTRNDRDAIIQHGTMTMTRRSVLEELGWADWCICEDAELGLRVFEKGLSAAYYHDSYGKGLMPDTFIDFK
KQRFRWAYGAIQIIKRHTASLLRGKDTELTRGQRYHFLAGWLPWVADGMNIFFTVGALLWSAAMIIVPTRVDPPLLIFAI

PPLALFVFKVGKIIFLYRRAVGVNLKDAFCAALAGLALSHTIAKAVLYGFFTTSIPFFRTPKNADNHGFWVAISEAREEM
FIMLLLWGAALGIYLVQGLPSNDIRFWVVMLLVQSLPYVAALIMAFLSSLPKPAAKAEPVTAV

>Pseudomonas_tuomuerensis_JCM_14085_[Orphan] PseudoCAP: Beta-(1-3)-glucosyl
transferase
MNHPTRPLLTLVIVVAIAALFIGAWALVNRPMPAPNWPEQVAGYSFSPFRPGQDPRDNRYPSDEDIRGDLQLVSGQTNNI
RTYSVEGSQADIPRLAEEFGLRVTLGVWISTDLERNEREITRAIELANQSRSVVRVLVGNEALYRGEIEPEALIAYIRRV
REAVKVPVSTSEQWHIWQEHPQLAREVDLIAAHILPYWEFIPRQHATAFVLERVQDLKKQFPGKPLLLSEVGWPSNGRAR
GPAEASPADQAIYLRTLVNALNLRGYNYFIIEAFDQPWKMDDEGSVGAYWGVWNLERQPKFAFSGPVIAIPQWRLLAIGS
AVLAVLALTLLLIDGSNLRTHGRAFLALVAFAFGSLLVWMAYDYSLQYATWFTLTVGALLGVGALGVFIVLLTEAHELAE
TVWLSRRRRPFPPVTRDDAYRPKVSVHVPCYNEPPDMLKRTLDALSRLDYPDFEVLVIDNNTKDPAVWQPVQAHCERLGP
RFRFFHVAPLEGFKGGALNWVLPHTAPDAEVIAVIDADYCVEPDWLRHMVPHFADPKIAVVQSPQDFRDGEASTFKRLCY
AEYKGFFHIGMVTRNERDAIIQHGTMTMTRRSVLDALGWADWCITEDAELGLRVFEQGLSAGYSERSYGRGLMPDTFSDY
KKQRFRWAYGAIQIMKHHARCLLLGKDCQLTSGQRYHFIAGWLPWIADGLNIFFTLGALLWSAAMIIVPQRVDPPLLIFA
LPPLALFVFKVGKMLYLYRRAVGVSLTDALYAAVAGLALSYTIAKAVLFGFVTRSIPFFRTPKLASRSGLFKALAEAREE
VFVMLLLWCAAAGLLLTEDNPGGDLYFWVLVLLVQSLPYLAALIMALLSSLPARERVETLATEG

>Pseudomonas_umsongensis_20MFCvi1.1_[Orphan] PseudoCAP: Beta-(1-3)-glucosyl
transferase
MSSRKFGLNLVVVLAIAALFTGFWALVNRPVTAPNWPEQISGFSYSPFQQGQYPQKDQYPTDDEMRRDLEIMSKLTDNIR
TYSVDGTLEDIPKLAEEFGLRVTLGIWISPDQERNEREIQRAIQLANTSRSVVRVVVGNEAIFRKEITAAELSVILDRVR
AAVKVPVTTSEQWHVWEEHPELAKHVDLIAAHVLPYWEFIPVDKAGQFVLDRARDLKKMFPKKPLLLSEVGWPSNGRMRG
GADASPADQAIYLRTLVNKLNRQGFNYFVIEAFDQPWKASDEGSVGAYWGVFNAARQQKFNFEGPVVAIPQWRVLAIGSV
VLALLSLTLLMIDGSALRQRGRTFLTFIAFLCGSVLVWIGYDYSQQYSTWFSLTVGFLLALGALGVFIVLLTEAHELAEA
VWTHKRRREFLPVVGDSDYRPKVSIHVPCYNEPPEMVKQTLNALANLDYPDFEVLIIDNNTKDPAVWEPVRDYCETLGPR
FKFFHVAPLAGFKGGALNYLIPHTAKDAEVIAVIDSDYCVHPNWLKHMVPHFADPKIAVVQSPQDYRDQNESTFKKLCYA
EYKGFFHIGMVTRNDRDAIIQHGTMTMTRRSVLEELGWADWCICEDAELGLRVFEKGLSAAYYHDSYGKGLMPDTFIDFK
KQRFRWAYGAIQIIKRHTASLLRGKDTELTRGQRYHFLAGWLPWVADGMNIFFTIGALLWSAAMIIVPQRVDPPLLIFAI
PPLALFVFKVGKIIFLYRRAVGVNLKDAFCAALAGLALSHTIAKAVLYGFFTSSIPFFRTPKNADNHGFWVAISEAREEL
FIMLLLWGAALGIFLVQGIPSNDMRFWVTMLLVQSLPYLAALIMAFLSSLPKPSVEAAPVPA

>Pseudomonas_veronii_R4_[Orphan] PseudoCAP: Beta-(1-3)-glucosyl transferase
MSSRKFGLNLVVVLAIAALFTGFWALINRPVTAPNWPEQISGFSYSPFQQGQYPQKDQYPTDDQMRRDLEIMSKLTDNIR
TYSVDGTLEDIPKLAEEFGLRVTLGIWISPDLERNEREIQRAIEIANSSRSVVRVVVGNEALFRKEITPEALIVLLDRVR
AAVKVPVTTSEQWHIWEHNPQLAKHVDLIAAHILPYWEHIPVDQAGQFVLDRARDLKKTFPKKPLLLSEVGWPSNGRMRG
GADASPADQAIYLRTLVNKLNRQGFNYFVIEAFDQPWKASDEGSVGAYWGVYNAARQQKFNFEGPVVAIPQWRVLAIGSV
VLALLSLTLLMIDGSALRQRGRTFLTFIAFLCGSVLVWIGYDYSQQYSTWFSLTVGFLLALGALGVFIVLLAEAHELAEA
VWTHKRRREFLPVVGDSDYRPKVSIHVPCYNEPPEMVKQTLDALAALDYPDYEVLIIDNNTKDPAVWEPVRDYCATLGPR
FKFFHVAPLAGFKGGALNYLIPHTAKDAEVIAVIDSDYCVSPNWLKHMVPHFADPKIAVVQSPQDYRDQNESTFKKLCYA
EYKGFFHIGMVTRNDRDAIIQHGTMTMTRRSVLEELGWADWCICEDAELGLRVFEKGLSAAYYHESYGKGLMPDTFIDFK
KQRFRWAYGAIQIIKRHTASLLRGKDTELTRGQRYHFLAGWLPWVADGMNIFFTVGALLWSAAMIIVPTRVDPPLLIFAI
PPLALFVFKVGKIIFLYRRAVGVNLKDAFCAALAGLALSHTIAKAVLYGFFTTSIPFFRTPKNADNHGFWVAISEAREEM
FIMLLLWGAALGIYLVQGLPSADMRFWVVMLLVQSLPYVAALIMAFLSSLPKPAPKAEPATAL

>Pseudomonas_viridiflava_LMCA8_[Orphan] PseudoCAP: Beta-(1-3)-glucosyl
transferase
MEWTSLRDLYRLKLRTLASSLFKKTIEGVPDWPDTLYGFSYAPYRPGQDPYKKIFPTREQIKEDLVLIRPLARHIRTYSV
EGTLADIPEIAETLGMQVTLGVWIAQDLKHNANELATAIDITRRSRNVERLLVGNEVLFRADVSISQLITYLEQARQQVE
VPVSTSEIWVQWHETPELSEHTDFIAAHILPFWEGLTPSQASNFVIDRADELRQMYPGKPLLMSEIGWPSRSSTATSVTT
STAEQSFYLRTQVAQLTKRNEQYFIIEAFDQPWKTEEGLAGPHWGVFDAQRRIKLQRSGPLETRVNWGSIYRRLIETSRP
RSLPRAVFSAATAYGLLVWAGIATAKALPLWALIPMSMVWAACFLTGMAVEVHEYLEIQWCPRKRRIFRPQRQQIKPLPK
VSVHVPCCNEPPEMVEQTLKALKNLDYPDYEVLVIDNNTQEQAVWKPVEQACQRLGKRFRFFHVEAMPGYKAGALNYLME
RTAPDVKVIAVVDADYCVDRNWLRHMAPHFEAPNIAVIQAPQNFRDSHESLFKYCCNAEYRGFFNIGMVIRNDHDAIIQH
GTMTLVRRDVLQRLRWSDGCICEDAELGLRILENGLSTGYSPISYGKGLTPDTFIHFKKQRYRWAYGAVQIVKQHAKSLF
STRGKTLTAMQRYHFLAGWVPWAAEGLNYLLTLAALSWSTAMIIAPYIVKPVPWLFSTSLLSALLFRTLKVIYLYRYQIS
TDIREALAAILAGMALYPTIGKAVLSGTFTSRLPFFRTPKQTQGGSSGRGLIEAREELCVTLFWLAIIGLHASNAGSDPD
LGFWKAMLFAQSLPYLAAVVMAVLSGKASRLVRPSG

>Pseudomonas_vranovensis_DSM_16006_[Orphan] PseudoCAP: Beta-(1-3)-glucosyl
transferase

MSSRKFGINLVIVMAIAALFTGFWALINRPVSAPDWPEQISGFSYSPFRLGESPQKGQYPNEDELRQDLEQLSKLTDSIR
IYTVEGTQAEIPRLAEEFGLRVTLGVWISPDLERNEREIQKAIELANSSRSVVRVMVGNEALFREEITPEALIQYLDRVR
AAVKVPVTTSEQWHIWKEHPELAKHVDLIAAHILPYWEFIPMKEAGQFVLDRARDLKQLFPRKSLLLSEVGWPSNGRMRG
GADATQADQAIYLRTLVNTLNRQGYNYFVIEAYDQPWKASDEGSVGAYWGVFNAARQQKFNFEGPVVAIPQWRVLAVGSV
VLAMLSLTLLLIDGSALRQRGRTFLTFIAFLCGSVLVWIGYDYSQQYSTWFSLTVGFLLALGALGVFIVLLTEAHELAEA
VWIHKRRREFLPVQADSAYRPKVSVHVPCYNEPPEMVKQTLNALAALDYPDYEVLIIDNNTKDPAVWEPIKAHCEMLGER
FKFFHVSPLAGFKGGALNYLIPHTAKDAEVIAVIDSDYCVDRNWLKHMVPHFADPKIAVVQSPQDYRDQNESTFKKLCYS
EYKGFFHIGMVTRNDRDAIIQHGTMTMTRRSVLEELGWADWCICEDAELGLRVFEKGYSAAYSHESYGKGLMPDTFIDFK
KQRFRWAYGAIQIIKRHSAALLRGKDSELTRGQRYHFLAGWLPWIADGMNIFFTVGALLWSAAMIIVPQRVDPPLLIFAI
PPLALFVFKVGKIVFLYRRAVGVNLKDAFAAALAGLALSHTIAKAVLYGFFTSSIPFIRTPKHADSHGLLVAISEAREEL
FIMVLLWGAAAGIYLVQGLPSNDMRFWVTMLLVQSLPYLAALIMAMLSSLPKPVDAVQPAQAS

>Pseudomonas_weihenstephanensis_DSM_29166_[Orphan] PseudoCAP: Beta-(1-3)-glucosyl transferase
MSSRKFGLNLVVVLAIAALFTGFWALINRPVSAPNWPEQISGFSYSPFQQGQYPQKDQYPSDDEMRRDLEIMSKLTDNIR
TYSVDGTLGDIPKLAEEFGLRVTIGIWISPDLERNEREIQKAIELANNSRSIVRVVVGNEALFREEITPEELIVLLDRVR
AAVKVPVTTSEQWHIWEKYPQLAKHVDLIAAHILPYWEFIPVDKAGEFVLDRARDLKKMFPKKPLLLSEVGWPSNGRMRG
GADATPADQAIYLRNLVNTLNRRGYNYFVIEAFDQPWKASDEGSVGAYWGVYNAARQQKFNFEGPVVAIPQWRVLAIGSV
VMGLLSLALLLIDGSALRQRGRTFLTFTAFLCGSVLVWIAYDYSQQYSTWFSLTVGFLLGLGALGVFIVLLTEAHELAEA
VWVRKRRREFLPVEADDAYRPKVSIHVPCYNEPPAMVKQTLDALANLDYPDFEVLIIDNNTKDPAVWEPVRDYCATLGPR
FKFFHVAPLAGFKGGALNYLIPHTAPDAEVIAVIDSDYCVDRNWLKHMVPHFADPKIAIVQSPQDYRDQNESTFKKLCYS
EYKGFFHIGMVTRNDRDAIIQHGTMTMTRRSVLEELGWADWCICEDAELGLRIFEKGYSAAYSHNSYGKGLMPDTFIDFK
KQRFRWAYGAIQIIKRHASSLLRGKDSQLTRGQRYHFLAGWLPWVADGMNIFFTVGALLWSAAMIIVPTRVDPPLLIFAI
PPLALFVFKVGKIIFLYRRTVGVNLKDAFAAALAGLALSHTIAKAVLYGFFTTSIPFFRTPKNADNHGFWVALSEAREEV
FIMLLLWGAALGICFVQGLPSNDMRFWVVMLLVQSLPYLAALVMAFMSSLPKPVDAPKEQPAA

>Rhizobium_meliloti_1021_[NdvB] Reference protein: P20471 Cyclic beta-(1,2)-glucan synthase, NdvB
MLQNTTQSNLPREPEAKQIDYNDSIRSTYFSIDDLRACGASLAEKGTSALPGFFPFEFRARHRENEKEILRVYRATAADV
EAGASITPAAEWLLDNHHVVEEAIQEVRRDFPRRFYRQLPTLSVSGTVIPRTMALAWLYVAHTHSTVTRESITAMVEGFQ
EHETLKIGELWALPSILRFVLIENLRRIAIRVERSRGMRRKANEVADQLIRLNDPEGCRTLLVESEALAADNTFIAQLLY
RMRDGSQSSGAVIAWIEERLERRGTDVEEALVAEQNRLSSGNATMSNIIRSLREIDDTDWAVWFESVSKIDATLREGSDY
AALDFGSRNTYRDTIEKLARRSGHSEHEVTEIAIEMVEEAKAAAAVEAPLQEPNVGSFLVGKQRLALEKRIGYSPSIFQH
LIRSVRKLDWFAIAGPNILLTILAMIVVYAFVSPMDIPSGAKLIMLLLFALPASEGAMGLFNTVFTLFAKPSRLVGYEFL
DGIPEDARTLVVVPCLIAKRDHVDELVRNLEVHYLANPRGEIYFALLSDWADSKSEEAPADTDVLEYAKREIASLSARYA
YDGKTRFFLLHRRRLYNEAEGVWMGWERKRGKLHELNLLLRGDRDTSFLQGANMVPEGVQYVMTLDSDTRLMRDAVTKLV
GKLYHPINRPVVNPRTQEVVTGYSLLQPRVTPSLTTGSEASAFQRIFTINRGIDPYVFTVSDVYQDIAGEGSFTGKGLYH
VDAFEAALKSRIEENAVLSHDLLEGSYARCALVTDIELVEDFPIRYEVEMSRQHRWARGDWQLLPYIFNPKNGLSMLGRW
KMYDNLRRSLIPVAWLAASVMGWYYMEPTPALIWQLVLIFSLFVAPTLSLISGIMPRRNDIVARAHLHTVLSDIRAANAQ
VALRIVFIAHNAAMMADAIVRSLYRTFVSRKLMLEWRTAAQVQSAGHGSIGDYFRAMWTAPALALVSLALAAISDTGLPF
IGLPFALIWAASPAVAWFVSQSAETEDQLVVSEEAIEEMRKIARRTWRYFEAFVTAEQNFLPPDNFQETPQPVLAERTSP
TNIGVYLLSVMSARSFGWIGFEETITRLEQTIATIDRMPKYRGHLFNWYRTRGLEPMEPRYVSSVDSGNLAGHLIAVSSM
CREWAEAPSAHVQGNLDGIGDVAAILKEALNELPDDRKTVRPLRRLVEERIAGFQNALAAVKRERELASIRVINLAVLAR
DMHKLTVNLDHEVRTVQSGEVATWAGSLVAACEAHIADGVFDLGAIEALRQRLLVLKERARDIAFSMDFSFLFRPERRLL
SIGYRVNANELDEACYDLLASEARLTSLFAIAKGDLPTEHWYKLGRPIVPIGARGALVSWSGSMFEYLMPPLVMQERQGG
ILNQTNNLVVQEQINHGRRLGTPWGISEAAFNARDHELTYQYTNFGVPTLGLKRGLGQNAVIAPYASILACMYDPKSALA
NLARLREVGALGAYGYHDAVDFTPTRVPEGQKCAVVRNYYAHHHGMSVAAVANVVFNGQLREWFHADPVIEAAELLLQEK
APRDIPVMAAKREPEALGKGQADLLRPEVRVVEDPINQDRETVLLSNGHYSVMLTATGAGYARWNGQSVTRWTPDPVEDR
TGTFIFLRDTVTGDWWSATAEPRRAPGEKTVTRFGDDKAEFVKTVGDLTSEVECIVATEHDAEGRRVILLNTGTEDRFIE
VTSYAEPVLAMDDADSSHPTFSKMFLRTEISRHGDVIWVSRNKRSPGDPDIEVAHLVTDNAGSERHTQAETDRRRFLGQG
RTLAEAAAFDPGATLSGTDGFTLDPIVSLRRVVRVPAGKKVSVIFWTIAAPDREGVDRAIDRYRHPETFNHELIHAWTRS
QVQMRHVGITSKEAASFQMLGRYLVYPDMHLRADAETVKTGLASQSALWPLAISGDFPIFCLRINDDGDLGIAREALRAQ
EYLRARGITADLVVVNERASSYAQDLQHTLDSMCENLRLRGLSDGPRQHIFAVRRDLMEPETWSTLISASRAVFHARNGT
ISDQIARATSLYSKSSEKKEEGAEMLLPVIREADARTAVELDGGDLDFWNGFGGFAEDGREYAVRLRGGEATPQPWINVI
SNEQFGFHVSAEGAAFSWSRNSRDYQLTPWTNDAVVNRPGEAIFVRDMASGAVLTPYAALSRRKSALFETRHGLGYSRFL
STQDELEIEAMHTVHRTLPAKLVRLTIRNRSSAARKLRVYGYAEWVLGNNRSRTAPFVLSEWDESAKTLVATNPYSIDYP
GRCAFFASDGDIAGYTASRREFLGRAGGILAPQAVISGAELTGSTDVDGDACAALATDITVEAGVERQVTFFLGDADNPD
QVRAVLEELRADSFGAALEAAKAFWGDFTGVVKVETPDRAFNHMINHWLPYQALGCRIMARSAFYQASGAFGFRDQLQDT
LAFLIHRPALARAQILNAAARQFVEGDVQHWWLPGTDAGVRTMISDDVVWLAHAVAHYCAVTGEEDILKEKVPFITGPAL
EEGQHDSFYKPDVADEVGDVYEHCARALDLAIHRTGANGLPLILGGDWNDGMNRVGEAGEGTSVWLGWFLAGTLRAFLPY
ARARKDKPRVALWERHLEALKDALEQAGWDGDYYRRGYYDDDTPLGSAENGECRIDSIAQSWSTLSGEGDKERSLRAMDA

VMAELVDPEKRIVRLFTPPLETTKQDPGYIKAYPPGVRENGGQYTHAATWVVLAFAAQERAEEAWRTFRMLNPVSHALSQ
VDAEHYRVEPYVVAADIYGEGALAGRGGWTWYTGSAGWLYRAGVEGILGIRKRGDKLLIRPVLPSEWPGYSAEVRVNGTT
HRISVSRDSKSGEPVVSVNNSVTKNAHEGVLL

>Rhizomucor_miehei_CAU432_[Glucanosyltransferase_RmBgt17A] BLAST: A0A0M3KKZ6
Beta-1,3-glucanosyltransferase, RmBgt17A
MASMTGGQQMGRGSQTFYGINYGVNENSCPTVDSMKNDFNVLKPYTNRVRTFALSVCNQASLALAATQALGMRIYLGMWI
DRPDTFDNEMNALKNILANNDVSNVDGLIVGSEVLYRGDTDPQSLANYIKQVKELVAPHGIKVATADVYYKFPEVVVKEL
DFLMMNAFPYWEGVTIDNAADTLMSHYDQVVGASLGKPVKISETGWPSAGGNFQSSVASVENENKYLHDVLCRVKQRNID
LLYFSAFDEPYRGGVEAHFGVLGSDRNTKPGITIEAGC

>Rhodobacter_sphaeroides_2.4.1_[BcsA] Reference protein: A0A3G6W9S6 Cellulose
synthase catalytic subunit [UDP-forming]
MTVRAKARSPLRVVPVLLFLLWVALLVPFGLLAAAPVAPSAQGLIALSAVVLVALLKPFADRMVPRFLLLSAASMLVMRY
WFWRLFETLPPPALDASFLFALLLFAVETFSISIFFLNGFLSADPTDRPFPRPLQPEELPTVDILVPSYNEPADMLSVTL
AAAKNMIYPARLRTVVLCDDGGTDQRCMSPDPELAQKAQERRRELQQLCRELGVVYSTRERNEHAKAGNMSAALERLKGE
LVVVFDADHVPSRDFLARTVGYFVEDPDLFLVQTPHFFINPDPIQRNLALGDRCPPENEMFYGKIHRGLDRWGGAFFCGS
AAVLRRRALDEAGGFAGETITEDAETALEIHSRGWKSLYIDRAMIAGLQPETFASFIQQRGRWATGMMQMLLLKNPLFRR
GLGIAQRLCYLNSMSFWFFPLVRMMFLVAPLIYLFFGIEIFVATFEEVLAYMPGYLAVSFLVQNALFARQRWPLVSEVYE
VAQAPYLARAIVTTLLRPRSARFAVTAKDETLSENYISPIYRPLLFTFLLCLSGVLATLVRWVAFPGDRSVLLVVGGWAV
LNVLLVGFALRAVAEKQQRRAAPRVQMEVPAEAQIPAFGNRPLTATVLDASTSGVRLLVRLPGVGDPHPALEAGGLIQFQ
PKFPDAPQLERMVRGRIRSARREGGTVMVGVIFEAGQPIAVRETVAYLIFGESAHWRTMREATMRPIGLLHGMARILWMA
AASLPKTARDFMDEPARRRRRHEEPKEKQAHLLAFGTDFSTEPDWAGELLDPTAQVSARPNTVAWGSN

>Rhodocyclaceae_strain_[Benzoate-transporter] InterPro PF00332-PF13641:
A0A2K9LB09 Benzoate transporter
MKHTASLIYRLMIASAIAALVAYAQYGIWQYLARGTEFSGSAQSIKGFAYTAFQRDQSPLKGTYPTDEQVGADLDLLARS
ADAIRTYSVRDIPAQLTEAGKRDLLVTAGAWISTDEAENRAEVDALIEAARKMRHIERVIVGNEVLLRGDLELEQLIAHL
DRARKALNKPVSTAEPWHVWLKNPELVRHVDFITVHLLPYHEGIPVDAALDYALMRYDELARTFPNKRIVIGEIGWPSRG
PTLKSFDGATEAVASVENQARFIHALLAHPRTARLDYYLMEAIDQPWKVEVEGWAGAYWGLYNADRQQKYALEGVVARDP
HWQKKASTAAALAMLPMLLVAFLLSDWNLFGRIFLAGLIQACVSTLIIGLNVPVDYYLNQRDLIGLTVLIAATVLTAAVL
LSHGFEFGEVLFKRRWRRRFMPLPPHPPERQPFVSIHLACHNEPPEMVIATLDSLAQLNYTNFEVLVLDNNTKDEAKWKP
LQARCAELGPRFRFFHLMDWPGFKAGALNYGLQVTDPRAEVIGVVDADYVVDPDWLAGLVPHFDSPDVAVVQAPQAHRDW
ENHPFRRMCNWEFEGFFRIGMHHRNERNALIQHGTMTLVRRLALQEVGGWSEWCICEDTELGLRLIEAGYDTRYVDHIYG
RGLTPSDFAAIKSQRFRWAFGAMQILKHHMPYMLGPSRLNLAQRYHFLTGWFAWLGDALQLVFALASLAWTLGILLFPKA
FGLPVTALALPVLAFMIFKAALGPILYRRTMDCPWRDILGASILSVGLAHAVARGVFAGLWKKHGTFVVTPKGWKAKGAL
AFFGPIREELGLLGALVLGIVAILWVHGAEQIETRLWAGILGLQCIPYLASIACQVAAYLPERTAPQAAAKAGSAPALQS

>Rhodospirillum_centenum_ATCC_51521_[Glycosyltransferase] InterPro PF00332-
PF13641: B6IR89 Glycosyl transferase, group 2 family protein
MRKILTMRGSAWAVLVLLVLGNIGAWAFSNRPAMPERPWSGTIAGVAFNPYKVGQDPTEGKHPSRQDIAGDLDVVAPYVL
SVRTYSALDGLEAIPELAAQRGLLATAGAWISQDLGANEQQINALVRIARRNDNVKRVMVGNEVMYRGDLEVEQLIDYVQ
RVKRQVDVPVSTAEPPYVWHEHPELAEAVDFITIHLLPYWEKVPIEQAMDQILTEYNRIKTAYPDKHILIGEVGWPSDGP
YRGGAEASLVNQATFIRSFLNLAGEQRWDFYIMEAFDQPWKRVLGGPVEANWGIWNADRQLKFPMDGSVWEVPGWPVLCA
IATALAFLPIFWFVLRRDDLKPAGQFFYGALIQTVASALVWTWTEAVTAGMGPLSTIVFALLLTAQIIILLAVMLIDGLEL
TEVVWTQNWKRRFEPIRGDSSGYAPKVSIHVPCYNEPAHMVIETLDALARMTYPNFEVLVIDNNTRDEAVWKPLEEHCRK
LGANFRFFHLPKWPGYKAGALNFGIAMTAPDAEVIAVIDSDYQVRPDWLSATVPYFRNPKVAFVQSPQDYREWNDHPFHR
MINWEYQGFFKIGMIQRNERNAIIQHGTMTLIRTQVLKDVGWWAEWCICEDAELGLRLFEQGYESVYMPDSFGQGLVPDS
FAGYKTQRFRWAYGAVQIIKRHWREFLPGGKRLTFGQKYHFVTGWLPWFADAAHMAFVIGGVFWSAGLLLLPRYFELPPT
VFLFPTLSVFFFKVICGLWLYEARVQCSLLDKISAAVAGMALTHTVGRAVWQGLFTQGRPFVRTPKCENQPALIQGFLNA
KEEVYLMLGLWIAAAAVAWRIGGDNRDAYIWSLMLVVQSLPYIAALVVSCLNVLPKGRDAADDALPAPAGAGGND

>Saccharomyces_cerevisiae_ATCC_204508_[Glucan_glucosidase_BGL2] BLAST: P15703
Glucan 1,3-beta-glucosidase, BGL2
MRFSTTLATAATALFFTASQVSAIGELAFNLGVKNNDGTCKSTSDYETELQALKSYTSTVKVYAASDCNTLQNLGPAAEA
EGFTIFVGVWPTDDSHYAAEKAALQTYLPKIKESTVAGFLVGSEALYRNDLTASQLSDKINDVRSVVADISDSDGKSYSG
KQVGTVDSWNVLVAGYNSAVIEASDFVMANAFSYWQGQTMQNASYSFFDDIMQALQVIQSTKGSTDITFWVGETGWPTDG
TNFESSYPSVDNAKQFWKEGICSMRAWGVNVIVFEAFDEDWKPNTSGTSDVEKHWGVFTSSDNLKYSLDCDFS

>Saccharomyces_cerevisiae_ATCC_204508_[Glucosidase_SCW4] BLAST: P53334 Probable
family 17 glucosidase, SCW4

MRLSNLIASASLLSAATLAAPANHEHKDKRAVVTTTVQKQTTIIVNGAASTPVAALEENAVVNSAPAAATSTTSSAASVA
TAAASSSENNSQVSAAASPASSSAATSTQSSSSSQASSSSSSGEDVSSFASGVRGITYTPYESSGACKSASEVASDLAQL
TDFPVIRLYGTDCNQVENVFKAKASNQKVFLGIYYVDQIQDGVNTIKSAVESYGSWDDVTTVSIGNELVNGNQATPSQVG
QYIDSGRSALKAAGYTGPVVSVDTFIAVINNPELCDYSDYMAVNAHAYFDKNTVAQDSGKWLLEQIQRVWTACDGKKNVV
ITESGWPSKGETYGVAVPSKENQKDAVSAITSSCGADTFLFTAFNDYWKADGAYGVEKYWGILSNEFAGTSTPCS

>Schizosaccharomyces_pombe_972_[Glucan_synthase_Ags1] Reference protein: Q9USK8 Cell wall alpha-1,3-glucan synthase, Ags1
MHGLQGLCFRRAVIALALLLFHSVFAAPYSEDEEPWNLNQNKNASSVLEYSGEWADHDFFPSPDNWRMSFITVILDRWYD
GDPSNNDIEKTPFEYDISEVSFRNGGDIVGLELSLDYLEGLGTQGIYIAGTPFVNMPWGADQYSPLDYTILDHHLGTIDQ
WRSTITAMHERGMYLVVDLTVATLGDLIGMVGHLNDTSGVDFNLNEYNAMWKTSEYRYVDFNFTNVYNTSCEYPRFWGED
GGPVSIQFKGCYVSDFDHYGDTEAFGSHPDWQRQLSKFASVQDRLRDWKPDVAEKLMHLSCLVISMLDVDGFRIDKATQM
TADFIVDWSMYVRECAAKYNKKNFLIVGEVTGSSSYGSIYYNRGRQPDQRPPNVTAAFNYTSDESQYSLRDSDHYGFDGS
AFHYSIYRALLRFLGMDSKMEIDFDVSSVLTTAWNGIQINEDAVNINTGTVEPLHMYGVANHDVFRWGAIENGTARLILG
TMITSLLFPGIPLLYYGDEQGMYVLDNSANNYLYGRQAMNSARAWYIHGCYNGSATSYPTVDLSPAQRGCQDSWNYLDHF
DIASAHRNVYRNIHSIRRHYLSLSEGWRFDHIANWTDDVYFPDSQPYASPMGLYSVLRGPMKEIQDFDSITNASNVSKSE
VWVLYANRNDTHLWSYDCTDEDSAIIGPWKSGTTLRNLIYPYDTIELEDSWNSSWGCIPNIELDPYAFKLYVPEEDFIEN
DPIITSLTPEHDARVVASGNEIDLTIEFSRSMDCDSIKNALSVVSSTRPKNTTAVIDVDSSFCRNYSEDASTSLHGQTAG
RFAWYGTLTNIDPGIHRISLKSVPTSDFSSRTLSTDNFLIRVGSTNNPIVHYSANYSDTLLIMQDGDLYINHSAPGAVLF
RYSTDFQSHWSDWEEYNGGLTKVQASNWTGTRRQGWEGHHIHVQYWSDLGGSANHMQQSDYGFKYRRFLPHMFLEGDFNE
YGYDSGVENRFLQKSDFYWEAGFISETYPAAIQLNVWGMNPDGIPDKTRVYGSQGNSTVLSRSDPASLVGNNITIYHPPP
HGYLSYKILLRDDDMIYRLAPSGEWGVSIAIYVLCIVIPPLSAIVVSWAFKNSFYTVKFNKHGNNDLGKFYPLKSLVPFR
KKNDLDSPAKVTPVVSGVSARKKKCVLIATLEYDITDWKIRIKIGGLGVMAQLMAQHLKHEDLVWVVPCVGDVVYPEAEE
ASPIEVKIIDQTYTINVYHHYLDNIKYVLLDAPVFRRQTSKEPYPARMDDLGSAIFYSAWNQCIAEVIRRNPIDIYHIND
YHGALAPCYLLPDIIPCALSLHNAEFQGLWPLRTPEEKEEVCAVYNISQRVCTKYVQFGNVFNLLHAAVSYIRIHQKGFG
AVGVSNKYGKRSWARYPIFWGLKKIGKLPNPDPTDTDEIVDDKAVAITDIDPDMEKSKVEHKRLAQEWAGLEVNEKYDLL
VFVGRWSSQKGIDLIADIAPSLLESYKVQLICVGPIIDLYGKFAAEKLDVLQKKYPTRVFSQPKFTQLPPYIFSGADFAL
IPSRDEPFGLVAVEFGRKGALGIGARVGGLGQMPGWWYSVESSATPHLLKQFEQACQQALSSSQRTRARLRARSAKQRFP
VSQWKAKLEALTDGCIKCSQKYGRNSRSRSSFYSLIHESFSRSSEVLPTSSDTNLDAKRAEEAEMIMIETPPTAEANTGA
KLDRSLSLGSRRGPGHTTEDDASDGLDTIQEESMTAGDSTSGGSDISRYRAERLNPDSHSPSEYSFDSGDYEFDPQRSYY
YDDLFDDDTTIRNAPSFRPQMGSFDAEHAVGATFSQDDLSDPARSVDSDSVSPPLPPFVAGSNPNARNNNNPYFYGNLHT
ESSLSLASVMSGKEKRDFSLTRVEETFTDEDGQALRSFSEKLQKLNAKNSKDDLCIEQYLMKSERSFFHERRAIKLGLQK
PNKLHVNELSSHSGTEESESLSNGQTSYDDIIAMTDESNYTQLGDDDFKTIHGLKKFMLFKIYDWPIYSIFLALGQILAA
TAYQLTLFTGTSNIQTYEIYSVCAFFIGASFVWWFMFARLPSYYVLSIPWLFYAVALFLVGLPAFDTVAPGRVWITNVAA
WIYAIASASGSIFFSLNFGEEGAVQTRIWVFRACLVQGVQQVWSAALWYWGAHLNKRLTAGEANTFKMSPAIPSITWPLS
AVSILIFALLFKGLPEYYRQLSGSIPAFYKSLLRRKLVVWFCISVFLQNFWLSSLNGRSWSYLWDIGNIHQWQIFLLIVA
FYIVLWALLLGVLAWISRTHSWIICVFGVGLGAPRWLQQFWATSNIGLYLPWAGYSGPYLGRTLWLWLGVLDAIQSVGIG
MILLQTLTRRHVASTLMTGQIVGAVATMIGRGASPNREGPANVFIDFTKWNHGDGSSILASAPFWINIICQLAICVGYLA
FFRRENLSRP

>Schizosaccharomyces_pombe_972_[Glucan_glucosidase_Bgl2] BLAST: O13990 Glucan 1,3-beta-glucosidase, Bgl2
MQFLSSFVFAALALLPLSAMAVDEAASEIASSTKPASTNGTLSFCLGVKHADGTCKYTDDYLADFEVLAPYTNMIRTYAT
SDCNTLEYLLPALAQSPYNFSAILGVWPTDDAHYDLEKQALMQYLPQYGVDHVRAITVGSEVLYRNDLPADVLAERIYDV
RGLVQQKLGFDVPVGTADSWNLWAGGSGDVVITASDFIMSNDFPYWQGQNTSNMTNTFISDTLAALERVQSVKGTNNVTF
WVGETGWPTDGPSYGEADATVDIASEFFQEALCNIRRKGIDIFFFEAFDEDWKGDSSSVEPYFGAMYSNRTLKYNLNCTS
E

>Salmonella_typhi_CT18_[BcsA] BLAST: Q8Z291 Cellulose synthase catalytic subunit [UDP-forming]
MSALSRWLLIPPVSARLSERYQGYRRHGASPFSAALGCLWTILAWIVFPLEHPRWQRIRDGHKALYPHINAARPRPLDPA
RYLIQTLWLVMISSTKERHEPRWRSFARLKDVRGRYHQWMDTLPERVRQKTTHLEKEKELGHLSNGARRFILGVIVTFSL
ILALICITQPFNPLSQFIFLLLLWGVALLVRRMPGRFSALMLIVLSLTVSCRYIWWRYTSTLNWDDPVSLVCGLILLFAE
TYAWIVLVLGYFQVVWPLNRQPVPLPKEMSQWPTVDIFVPTYNEDLNVVKNTIYASLGIDWPKDKLNIWILDDGGRESFR
QFARHVGVHYIARATHEHAKAGNINNALKHAKGEFVAIFDCDHVPTRSFLQMTMGWFLKEKQLAMMQTPHHFFSPDPFER
NLGRFRKTPNEGTLFYGLVQDGNDMWDATFFCGSCAVIRRKPLDEIGGIAVETVTEDAHTSLRLHRRGYTSAYMRIPQSA
GLATESLSAHIGQRIRWARGMVQIFRLDNPLFGKGLKLAQRLCYLNAMFHFLSGIPRLIFLTAPLAFLLLHAYIIYAPAL
MIALFVIPHMVHASLTNSKIQGKYRHSFWSEIYETVLAWYIAPPTLVALINPHKGKFNVTAKGGLVEEKYVDWVISRPYI
FLVLLNLLGVAAGVWRYYYGPENETLTVIVSLVWVFYNLVILGGAVAVSVESKQVRRAHRVEIAMPGAIAREDGHLFSCT
VHDFSDGGLGIKINGQAQVLEGQKVNLLLKRGQQEYVFPTQVVRVTGNEVGLQLMPLTTKQHIDFVQCTFARADTWALWQ
DSFPEDKPLESLLDILKLGFRGYRHLAEFAPPSVKVIFRSLTALIAWIVSFIPRRPERQAAIQPSDRVMAQAQQ

>Salmonella_typhimurium_LT2_[BcsA] BLAST: Q93IN2 Cellulose synthase catalytic subunit [UDP-forming]
MSALSRWLLIPPVSARLSERYQGYRRHGASPFSAALGCLWTILAWIVFPLEHPRWQRIRDGHKALYPHINAARPRPLDPA
RYLIQTLWLVMISSTKERHEPRWRSFARLKDVRGRYHQWMDTLPERVRQKTTHLEKEKELGHLSNGARRFILGVIVTFSL
ILALICITQPFNPLSQFIFLLLLWGVALLVRRMPGRFSALMLIVLSLTVSCRYIWWRYTSTLNWDDPVSLVCGLILLFAE
TYAWIVLVLGYFQVVWPLNRQPVPLPKEMSQWPTVDIFVPTYNEDLNVVKNTIYASLGIDWPKDKLNIWILDDGGRESFR
HFARHVGVHYIARTTHEHAKAGNINNALKHAKGEFVAIFDCDHVPTRSFLQMTMGWFLKEKQLAMMQTPHHFFSPDPFER
NLGRFRKTPNEGTLFYGLVQDGNDMWDATFFCGSCAVIRRKPLDEIGGIAVETVTEDAHTSLRLHRRGYTSAYMRIPQAA
GLATESLSAHIGQRIRWARGMVQIFRLDNPLFGKGLKLAQRLCYLNAMFHFLSGIPRLIFLTAPLAFLLLHAYIIYAPAL
MIALFVIPHMVHASLTNSKIQGKYRHSFWSEIYETVLAWYIAPPTLVALINPHKGKFNVTAKGGLVEEKYVDWVISRPYI
FLVLLNLLGVAAGVWRYYYGPENETLTVIVSLVWVFYNLVILGGAVAVSVESKQVRRAHRVEIAMPGAIAREDGHLFSCT
VHDFSDGGLGIKINGQAQVLEGQKVNLLLKRGQQEYVFPTQVVRVTGNEVGLQLMPLTTKQHIDFVQCTFARADTWALWQ
DSFPEDKPLESLLDILKLGFRGYRHLAEFAPPSVKVIFRSLTALIAWIVSFIPRRPERQAAIQPSDRVMAQAQQ

>Starkeya_novella_ATCC_8093_[Glycosyltransferase] InterPro PF00332-PF13641: D6ZYK0 Glycosyl transferase family 2
MRPAVRVAAAAVAAVTCIHAFVWLALREEVTAPAVPDRFQSMSFAPYGRDTNPDKGEATTEAQIRSDIEAVAPYTRAVRT
YASTGNLDLVTKIAAEKGLKTTIGAWLDEDEDRNAREITNAIDVARKNSNVMGIVVGNESILRAERTPQQLIDTIRKVKS
QTNVPVTTGETWDVWLDHPELVSAVDYIAAHILPYWEGVPADQVVDRTIGIYDRLRAAYPGKRIVIAEFGWPSAGYNRDA
AVPGELEQAKVIRTFAARADALGIEYNLIEAFDAPWKSFEGSVGQYWGMLDADRELKFSLSGPLTPATYTATAAIALLLG
IAFSLPVFRFARLTMTQAVVMIGAANLVGAWIATVTDHWLTHYVVGGNKVTFIVSLVLLVPLVVVMLYRIEELATIAFGR
SPRRLLRAGNAAQPTRTPKVSIHIPAYKEPPEMLKQTLDSVARLNWPNFECLVIINNTPDPAFWEPIEEHCRELGERFKF
INLPKVAGFKAGALREAMLQTAPDAEIIGVIDADYVVDPNWLMDLVPTFEDPTVGIVQAPQDHRDANRSLLHEAMNTEYA
GFFDIGMVQRNEHDAIVVHGTMCLMRRAAMVEAGDWSSETICEDTDLGLSIVERGWKSHYTNTRYGWGLLPDDFASFKKQ
RHRWAYGGMQIIKKHWRRMLPNGGTRLTPAQRREFSIGWVSWLGSESIGALVAVLSLLWVPFVLAFGIAVPQHILTMPIL
FCFGIYLLHFIALYRLRVATTPVRMLGAAFAAMAVQFTVAKAVYDSFRYKDLAFARTAKGSWLADAARAFPALPEAIIGS
GLMLSAIALRMTNWHAVVEIDLFAFALAIQSLPFLAAAGIGWLEGSPLNAFPTWASLRARALALLPGRAAQAEAIPEKVR
S

>Thauera_chlorobenzoica_[Glycosyltransferase] InterPro PF00332-PF13641: A0A1H5YFF0 Glycosyl hydrolase family 1 protein
MKNAATFATRLAIAVAIAIAVAFAQYWIWQQFNRGAEFIGTNQSIKGFAYNGFQRDQSPLTGTYPSRENLAADLDLLGRM
SDGLRTYGVTDMPELLGLAGERDMMVTAGAWLDADKTRNAREIAALLEVAPKMRHIERVMVGNEAVLRGDLTVSELIGYL
DQVRKTLRKPVSTAEPWHVWLRYPELAKHVDYITVHLLPYHEGVPVEAAVEYAFQRYDEVARAHPRKKIVVGEIGWPSRG
PTIGAALPSLDNEARFIREFLAHPRTARIDYFLMEAIDQPWKVDVEGWAGPYWGMFNADREQKFALEGIIERDPHWSTKA
SNAAALAFLPMLLIAFLLADWSIFGRLFLAALIQACVSTLIIGLNVPVDYYLTQRDLVGLILLIGATGLTAAVLLSHGFE
FGEVLFKKKWQRRFLPLPPHAPEQQPFVSIHLACYNEPPEMVIATIDSLAQMNYQNFEVLVLDNNTRDEALWKPLEKRCA
ELGSRFRFFHLANWPGFKAGALNYGLKVTDPRAAVVGVVDADYVVEPDWLACLIPHFDQSEVAVVQAPQAHRDWETQPFK
RMCNWEFDGFFRIGMHHRNERNALIQHGTMTLVRRRALEEVGGWSEWCICEDTELGLRLIEKGYDTRYVDHIFGRGLTPS
GFAAIKSQRFRWAFGAMQILKAHLPQMLGRSTLNLAQRYHFLTGWFAWLGDALQLVFAFGSLLWTLGILLFPKAFGLPVI
ALALPILGFMAFKAALGPILYRRTMDCPWKDILGASILSVGLAHAIARGVFAGLVKKKGVFVVTPKGWRGGGALAFFNPI
REEIGMLVALLMGAATLVAQRGADNLETQLWVGILCLQCIPYVAAILCQVAAYMPERNSGDTPRGTLLPQA

>Thiocystis_violascens_ATCC_17096_[Glucanase] InterPro PF00332-PF13641: I3YBZ5 Exo-beta-1,3-glucanase
MNRSINLVIPLLIAALTATAWWLLNQPSEEPPWPNRIQGFSFAPMRADDDPSLKKFPSVEDIDADLALLEGRAHAVRTYT
VEDTLAEIPRLAAAHDLNVTLGAWIGAEPAANETEIKRLAEVLNEGHRNLVRVIVGNESILRRDLPVSELIGYLDRVRKL
TWLPVSTAEPWHVWLKYPKLVEHVDFITVHLLPYWEGLPVDQAVDYAVSRYNELKEAYPKKPIVIGEVGWPSNGRRNRGA
EPSPTNQTRFLRRFLARAEAENYIYYVMEAFDQPWKAKTEGATGNYWGVYNANRQAKFEFSQPVVRIPQWRELAGLSVVM
GVLLLVFLYRDSATLAKRGKGFLALVTYAISTAAVWIVYDYTRQYMTPATAIVGVLLLIGGIGVIVLLMAEAHEWAESIW
LNKWRRPFPLRTVPDEQLPFVSVHVPAYNEPPELLCATLDALAALDYPRFEVLVIDNNTKDPKVWEPVQAHCETLGERFR
FFHVDPLAGYKAGALNFALRQTDPAAEVIAVIDADYLVIPTWLRHLVPGFADPEVAIVQAPQDYRDADQNAFKAMCMAEY
RGFFHLGMVTRNERNAIIQHGTMTMIRRQTLDEVDGWAEWCITEDAELGLRLFEAGHKALYIPCTYGRGLMPDTFADFQK
QRYRWAYGAVRILLRHRRELLGLRKTALTAGQRYHFVAGWLPWFADGFNLLFNFAALGWSVAMVMFPDTITPPYLTVALV
PLVLFVFKMSKSLVLYRRRVTATLRQSLAAGLAGLALSHTISRAMFGGLVTGKLGFFRTPKMAEAPAVVRALADTREEGL
FAFAFLLAAGLVLLRDDAYMLDVRIWVAVLVVQSIPYLASVLVSLVSAWQRLPADLVGVMSEMNGQRLSGVQPVSREG

>Thiosulfatimonas_sediminis_[Glycosyltransferase] InterPro PF00332-PF13641: A0A6F8PUG3 Glycosyl transferase, NdvB
MHNFSVVVLLISIIFGTYAYFNQPIEEPAWPNDIPGFAFSPYQKGQSPFLEKYPTPEQIDRDLELLSGKAHAIRTYTVDK

VFAQIPQLAYKYKINLALGAWLDKDQARNTAEIERFIQLIEQPPYNIVRAIVGNEVLLREDLSPEQLIAVLQDVRAKTSV
PVGTAEPWHVWIKHPELVDEVDFIAVHMLPFWEGIALEDSIEYIVDKMNLLRSTFPGKPIVITEVGWPSKGRAIKKAEAS
RANQAIFMRRFIQRAQQEKYIFYMMEAFDQPWKNRLEGSVGGHWGLYDVDRQLKFEQQTPIIALPEWRFLAAASALLAVA
MIAFLLIDSGALRKRGRSFLAVLAFLISSLVIWMIYDYSSEYHNWVGVLVGILLLLGVVGVIVVILTEAHEWAEAIWYHQ
RRRPLIEVPVQQGVCPFVSIHVPAYNEPPEMMKETLLALSQLDYPCFEVLVIDNNTRDESVWRPVEDFCAELGERFRFYH
VSPLSGFKAGALNYALQRTAEQAEIIAVIDSDYRVNPDWLKRMVGQFHEPNVAIVQAPQDYWDYSQNAFKAMCYAEYKGF
FHIGMVTRNERNAIIQHGTMTMVRHRVLQAVGGWGETTITEDAELGLRIFEQGYAAVYSEHSFGKGVMPDTYIDYKKQRY
RWAYGAMQILREHAGALLDWNKTQLKPGQRYHFFAGWLPWIADGFNYIFTVLAIIWTGLMLSDPVQYNAPDWMISIIPIV
FFGFKMSKMLVLYMGHVGANLRTALAAAISGLALSHTIAKAVLWGLFVGRKMPFLRTPKLADSHNLWQALLDAYEEALMA
AILLTAAVLLYWKFGFDTFEINLWFAVLLVQSIPYFASLMLSIISAIPNLRGDWVRFKSDLSNQ

>Xanthobacter_autotrophicus_ATCC_BAA-1158_[Glycosyltransferase] InterPro
PF00332-PF13641: A7INQ0 Glycosyl transferase family 2
MRPGLAAAAAVIAVVTSIHAAMWLSLRPEVSAPNITDRFQSLSFAPFSRDMSPEGEAPTNAAQIRSDMQVVAPYTRGVRT
YSSTNGKELIAPIAGEQGIRVTAGAWLNRQSDKDTGEIIPKAKAANDREIAGVIEVARQNRNVQAVVVGNETLLRGDMSD
DELAELIRSVKRQVNVPVTSGEIWNTWLDHPKVASSVDFILAHILPYWEDVPADKVVDYTIDAYNRLRAAYPGKRIVIGE
FGWPSHGYNRGASVPDPLAQAMIIRDFVARADALGIEYNIIEAFDLPKKQNEGSVGQYWGVFDADRDLKFPLSGPIYDHT
YNQTTILALLLGVLFTLPLLRMRELTMSQGLVLAGAANGVAAWLAMVVDYWLNHYVTGGDYVTLALSVVMLVPLVFVLLY
RIEEMAAIAFGSGPRRLIDARKAAVVPTVPSRFPKVSIHVPAYREPPEMLKQTIDALAALEYPNFEAIIIINNTPDPAMV
EPVREYCAALGERFKFINAEKVAGFKAGALRIALDATAPDAEIIGVIDADYVVTPDWLKELVPVFDDPTVGLVQAPQDHR
DADRSLLHEAMNAEYAGFFDIGMVQRNEDDAIVVHGTMCLIRRAAMLEAGNWSSDTICEDTDLGLTIAENGWKTHYTRKR
YGYGLLPDSFEAFKKQRHRWAYGGFQIIKKHWRKFLPNRSRLTTAQKRHFVLGWISWLGSESVGAVMAIASLAFVPFVLL
FGVSVPAHVLTLPILITFLVYLMHFVSLYRLRVETTPMRMLGAAVAASAVQYTVAKAVLDGFRYKDLAFARTAKGNNWLA
GAARSFPALPEATLGGLLLLAGIALLVLNNWPGVALTKNDWRHIREINLYGIALMVQSLPFVAAALIGAFEPSRFNQVAF
WRALGAKLSLAPRRPGLTPPPAVAD

>Xanthobacter_strain_YN2_[Glycosyltransferase] InterPro PF00332-PF13641:
A0A7U3E1T5 Glycosyltransferase
MRPGLAAAAAVIAVVTSFHAAMWLSLRPEATAPNVTDRFQSLSFAPFSRSMSPEGEAPTNAAQIRSDINAVAPYTRAVRT
YSSTNGKELIAPIAGEQGIKVTAGAWLNRETDKKTGEVIAKAREANQRELAGVVDVARQNQNVQAVVVGNETLLRDDMND
EELATLIRSVKRQVNAPVTTGEIWNTWLDHPKVASSVDFILAHILPYWEDVPANQVVDYTINAYNRLRAAYPGKRIVIGE
FGWPSHGFNRGASVPDPIEQAKIIRDFVARADALGIEYNIIEAFDLPKKQNEGSVGQYWGVFNADRQLKFPLTGPIFDHT
YNQTGTLAVLLGVLFALPLLRMRDLTVSQGFVLAAAASGVAAWLALVVDYWLNHYVTGGDYITLALSVVMLVPLVFVLLY
RVEELAAVAFGRGPARLVEAQKLGAPSRFPKVSIHVPAYREPPEMLKQTIDALARLEYPNFEAIIIVNNTPDPAMVEPVR
DHCALLGDRFKFINAEKVSGFKAGALRIALEATAPDAEIIGVIDADYVVTPDWLKDLVPVFDDPTVGLVQAPQEHRDGAR
SPLHEAMNHEYAGFFDIGMVQRNEDDAIVVHGTMCLIRRAAMMEAGNWSSDTICEDTDLGLSIAENGWKTHYTRKRYGYG
LLPDTFEAFKKQRHRWAYGGFQIIKKHWRRFLPNNSRLTTAQKRHFILGWVSWLGSESVGAFMAIASLAFVPFVLLFGVS
VPAHVLTLPILVTFLVYLMHFVSLYRLRVATSPLRMFGAALAASAVQFTVAKAVLDGFRYKDLAFARTAKGNNWLAGAAR
SFPALPEAILGGLLLVSGMVLLVLNNWPGLALTKNDWKHIREIDLYGVALMVQSLPFVAAAVIGLFEPSRLNDFATWRAI
GARLSAVPRRLGITPPPAVAD

>Zoogloeaceae_strain_Par-f-2_[Benzoate_transporter] InterPro PF00332-PF13641:
A0A2S0QWP1 Benzoate transporter
MKHTASLIYRLVIASAIAALVAYAQYGIWQYLARGTEFSGSAQSIKGFAYTAFQRDQSPLKGTYPTDEQVGADLDLLARS
ADAIRTYSVRDIPAQLTEAGKRDLLVTAGAWISTDETENRAEVDALIEAARKMRHIERVIVGNEVLLRGDLELEQLIAHL
DRARKALNKPVSTAEPWHVWLKNPELVRHVDFITVHLLPYHEGIPVDAALDYALMRYDELARTFPNKRIVIGEIGWPSRG
PTLKSFDGATEAVASVENQARFIHALLAHPRTARLDYYLMEAIDQPWKVEVEGWAGAYWGLYNADRQQKYALEGVVARDP
HWQKKASTAAALAMLPMLLVAFLLSDWNLFGRIFLAGLIQACVSTLIIGLNVPVDYYLNQRDLIGLTVLIAATVLTAAVL
LSHGFEFGEVLFKRRWRRRFMPLPPHAPERQPFVSIHLACHNEPPEMVIATLDSLAQLNYANFEVLVLDNNTKDEAKWKP
LQARCAELGPRFRFFHLMDWPGFKAGALNYGLQVTDPRAEVIGVVDADYVVDPDWLAGLVPHFDSPDVAVVQAPQAHRDW
ENHPFRRMCNWEFEGFFRIGMHHRNERNALIQHGTMTLVRRLALQEVGGWSEWCICEDTELGLRLIEAGYDTRYVDHIYG
RGLTPSDFAAIKSQRFRWAFGAMQILKHHMPYMLGPSRLNLAQRYHFLTGWFAWLGDALQLVFALASLAWTLGILLFPKA
FGLPVTALALPVLAFMIFKAALGPILYRRTMDCPWRDILGASILSVGLAHAVARGVFAGLWKKHGTFVVTPKGWKAKGAL
AFFGPIREELGLLGALVLGIVAILWVHGAEQIETRLWAGILGLQCIPYLASIACQVAAYLPERTAPQAAAKAGSAPALQN
