# Supplementary material for: Bioinformatics characterization of BcsA-like orphan proteins suggest they form a novel family of pseudomonad cyclic-β-glucan synthases
Source: PLoS One. 2023 Jun 2;18(6):e0286540. doi: 10.1371/journal.pone.0286540 (PMC10237404; doi:10.1371/journal.pone.0286540)
Supplement: S2 File — This is a list of single-domain homology models and predicted structure models (PDB files) generated in this work and available from DOI: XXX (to be added after acceptance). (PDF) [file pone.0286540.s006.pdf]

Supporting File S2. **Single-domain homology and predicted structure models.**

This is a list of single-domain homology models and predicted structure models (PDB files) generated in this work and available from DOI: XXX (to be added after acceptance).

**AlphaFold_Ab_ED-1**	AlphaFold predicted structure of the *Arcobacter butzleri* ED-1 Orphan protein.
**AlphaFold_Az_DN11**	AlphaFold predicted structure of the *Azoarcus* strain DN11 Orphan protein.
**AlphaFold_Pa_PA01**	AlphaFold predicted structure of the *Pseudomonas aeruginosa* PA01 Orphan protein.
**AlphaFold_Pf_SBW25**	AlphaFold predicted structure of the *Pseudomonas fluorescens* SBW25 Orphan protein.
**AlphaFold_Pf_SBW25_SP**	AlphaFold predicted structure of a truncated *Pseudomonas fluorescens* SBW25 Orphan protein lacking the signal peptide sequence.
**AlphaFold_Pp_KT2400**	AlphaFold predicted structure of the *Pseudomonas putida* KT2440 Orphan protein.
**AlphaFold_Ps_DC3000**	AlphaFold predicted structure of the *Pseudomonas syringae* DC3000 Orphan protein.
**AlphaFold_Pv_LMCA8**	AlphaFold predicted structure of the *Pseudomonas viridiflava* LMCA8 Orphan protein.
**AlphaFold_Mm_MC09**	AlphaFold predicted structure of the *Methylomonas methanica* MC09 Orphan protein.
**Phyre2_Pf_SBW25_GH17**	Phyre^2 homology model of the GH17 domain of the *Pseudomonas fluorescens* SBW25 Orphan protein.
**Phyre2_Pf_SBW25_GT2**	Phyre^2 homology model of the TM region and GT2 domain of the *Pseudomonas fluorescens* SBW25 Orphan protein.
I**nterFOLD6_Pf_SBW25**	InterFOLD6 predicted structure of the *Pseudomonas fluorescens* SBW25 Orphan protein.
**RoseTTAFold_Pf_SBW25**	RoseTTAFold predicted structure of the *Pseudomonas fluorescens* SBW25 Orphan protein.
**SWISSMODEL_Pf_SBW25_GH17**	SWISS-MODEL homology model of the GH17 domain of the *Pseudomonas fluorescens* SBW25 Orphan protein.
**SWISSMODEL_Pf_SBW25_GT2**	SWISS-MODEL homology model of the TM region and GT2 domain of the *Pseudomonas fluorescens* SBW25 Orphan protein.
**TrRosetta_Pf_SBW25**	TrRosetta predicted structure of the *Pseudomonas fluorescens* SBW25 Orphan protein.
