# Supplementary material for: Bioinformatics characterization of BcsA-like orphan proteins suggest they form a novel family of pseudomonad cyclic-β-glucan synthases
Source: PLoS One. 2023 Jun 2;18(6):e0286540. doi: 10.1371/journal.pone.0286540 (PMC10237404; doi:10.1371/journal.pone.0286540)
Supplement: S3 File — Clustal Omega [70] was used to produce a multiple sequence alignment of 26 Orphan proteins identified in Pseudomonas aeruginosa AZPAE12140, BL14, PAK, PA01, PA14, LESB58, 19BR and 3573, P. fluorescens ICMP 11288, ICMP 3512, KF1, LMG 5329, SBW25, SS101, WH6 and WS 5037, P. putida KT2440, S610, W619 and YKD221, and P. syringae B728a, DC3000, ICMP 9617, NCPPB 4273, UMAF0158 and 41a strains, using the PaPA14 Orphan (NdvB) as the reference sequence (see S1 File for protein sequences). Orphan proteins show 95.3–100.0% coverage and 46.9–99.2% sequence identity normalised by aligned length. The Mview [70] file was copied and annotated to show the signal peptide sequence predicted by Proteus2 [79], the first (GH17) and second (GT2) Orphan domains, and conserved domains, motifs, and residues found in homologous fungal proteins and BcsA proteins. (PDF) [file pone.0286540.s007.pdf]

Supporting File S3. **Multiple sequence alignment of 26 Orphan proteins from representative *P. aeruginosa*, *P. fluorescens*, *P. putida* and *P. syringae* strains.**

Multiple Sequence Alignment produced by Clustal Omega and Mview using the *Pseudomonas aeruginosa* PA14 sequence as the reference. Key pseudomonads are **highlighted**. Percent coverage (and sequence identity (ID) normalised by length shown for each sequence. The signal sequence identified in the *Pa* PA01, *Pp* KT2440 and *Pf* SBW25 Orphan proteins is mapped for *Pf* SBW25 and indicated by the **yellow** line (no signal sequence was identified in the *Ps* DC3000 Orphan protein). The first (GH17) Domain is mapped for *Pf* SBW25 and indicated by the **green** line and the second (GT2) domain by the **blue** line. The **red** line indicates the position of the gating loop in *Rs* BcsA. Consensus sequences (100% and 70%) and conserved domains, motifs, and residues found in homologous fungal proteins and BcsA proteins are indicated in **red** at the bottom of each section if present.

```
   Species                     Coverage & ID                    .         .         .         .         .         .         .         . 80

1  P. aeruginosa PA14          100.0% 100.0%   -----------MSSRKIGLNLVVIVALAALFTGIWA---LYNRPVSVPDWPERISGFSFSPFRLNQNPQSGRYPSAEQMR
2  P. aeruginosa 19BR          100.0%  99.2%   -----------MSSRKIGLNLVVIVALAALFTGIWA---LYNRPVSVPDWPERISGFSFSPFRLNQNPQSGRYPSAEQMR
3  P. aeruginosa BL14          100.0%  99.8%   -----------MSSRKIGLNLVVIVALAALFTGIWA---LYNRPVSVPDWPERISGFSFSPFRLNQNPQSGRYPSAEQMR
4  P. aeruginosa 3573          100.0%  99.5%   -----------MSSRKIGLNLVVIVALAALFTGIWA---LYNRPVSVPDWPERISGFSFSPFRLNQNPQSGRYPSAEQMR
5  P. aeruginosa LESB58        100.0%  99.2%   -----------MSSRKIGLNLVVIVALAALFTGIWA---LYNRPVSVPDWPERISGLSFSPFRLNQNPQSGRYPSAEQMR
6  P. aeruginosa PA01          100.0%  99.4%   -----------MSSRKIGLNLVVIVALAALFTGIWA---LYNRPVSVPDWPERISGFSFSPFRLNQNPQSGRYPSAEQMR
7  P. aeruginosa AZPAE12140    100.0%  99.5%   -----------MSSRKIGLNLVVIVALAALFTGIWA---LYNRPVSVPDWPERISGFSFSPFRLNQNPQSGRYPSAEQMR
8  P. aeruginosa PAK           100.0%  99.4%   -----------MSSRKIGLNLVVIVALAALFTGIWA---LYNRPVSVPDWPERISGFSFSPFRLNQNPQSGRYPSAEQMR

9  P. fluorescens SBW25         99.0%  73.7%   -----------MASRKFGLNLVIVLAIAALFSGFWA---LINRPVTAPNWPEQISGFSYSPFQQGQYPQKDQYPTDDQMR
10 P. fluorescens SS101         99.3%  73.6%   -----------MASRKFGLNLVVVLAIAALFTGFWA---LINRPVTAPNWPDQISGFSYSPFQQGQYPQKDQYPTDEQMR
11 P. fluorescens ICMP 3512     99.3%  73.3%   -----MEPEIAMSSRKFGLNLVVVLAIAALFTGFWA---LINRPVTAPNWPEQISGFSYSPFQQGQFPQKDQYPSDEEMR
12 P. fluorescens WS 5037       99.3%  73.8%   -----------MSSRKFGLNLVVVLAIAALFTGFWA---LINRPVTAPNWPEQISGFSYSPFQQGQFPQKDQYPSDEEMR
13 P. fluorescens ICMP 11288    99.3%  73.9%   -----------MSSRKFGLNLVVVLAIAALFTGFWA---LINRPVTTPNWPEQISGFSYSPFQQGQFPQKDQYPTDDQMR
14 P. fluorescens WH6          99.3%  73.9%   -----------MASRKFGLNLVVVLAIAALFTGFWA---LINRPVTAPNWPEQISGFSYSPFQQGQYPQKEQYPTDEQMR
15 P. fluorescens KF1          99.3%  74.1%   -----------MSSRKFGLNLVVVLAIAALFTGFWA---LINRPVTTPNWPEQISGFSYSPFQQGQYPQKDQYPTDDQMR
16 P. fluorescens LMG 5329     99.3%  73.7%   -----------MASRKFGLNLVIVLAIAALFTGFWA---LINRPVTTPNWPEQISGFSYSPFQQGQYPQKDQYPTDDQMR

17 P. putida S610              99.5%  75.7%   -----------MSSRKFGLNLVVVLAIAALFTGFWA---LINRPVSAPAWPEQISGFSYSPFRLGESPQKGQYPDDNEMR
18 P. putida W619              99.3%  76.4%   -----------MSSRKFGLNLVIVLAIAALFTGFWA---LVNRPVSAPAWPEQISGFSYSPFRLGESPQKGQYPSDAEMR
19 P. putida KT2440            99.3%  76.3%   -----------MSSRKFGLNLVVVLAIAALFTGFWA---LINRPVSAPAWPEQISGFSYSPFRLGESPQKGQYPTDDEMR
20 P. putida YKD221            99.3%  76.3%   -----------MSSRKFGLNLVVVLAIAALFTGFWA---LINRPVSAPAWPEQISGFSYSPFRLGESPQKGQYPTDDEMR

21 P. syringae ICMP 9617       95.3%  46.3%   -----MNHTATI-------------NFNRLKDGFSPKKLFQRRLIEVPDWPASISGFSYAPFRPGQSARKKIYPTREQIK
22 P. syringae B728a           95.3%  46.1%   -----------M-------------DENSNNTGTWPTNLFKTRLNYPPNWPAMISGVAYAPFRPGQSPYKQIFPTRDQIR
23 P. syringae UMAF0158        95.0%  45.2%   -----MEHIADM-------------DEYSNKAGTWLKNLFKTRFNYPPNWPAMISGVAYAPFRPGQSPYKQIFPTRDQIR
24 P. syringae DC3000          95.3%  45.4%   MSIYRMEHSLDM-------------NKKISDAPIWPVNSFKSVVTKVPDWPDSISGLAYNPFRPGQSPYKHIYPTREQIK
25 P. syringae NCPPB 4273      95.3%  47.3%   -----MEHIADM-------------DDNLSKRNIWPAHSLTTMASEVPDWPENIPGVSYSPFRPGQSPYTHLYPTREQIT
26 P. syringae 41a             95.3%  46.9%   -----------M-------------DDNLSKGNIWPARSLTTMASEVPDWPENIPGVSYSPFRPGQSPYTHLYPTREQIT

   Consensus/100%                              ...........h.............s.....tsh.....h.ph...sPsWPt.IsGhuasPFp.sp.s.pt.aPsttphp
   Consensus/70%                               ...........MuSRKhGLNLVlllAlAALFTGhWA...LhNRPVosPsWPEpISGFSaSPFp.sQ.PQpspYPos-QMR

   Signal sequence & first domain
```

	Species	Coverage	ID	 160
1	*P. aeruginosa* PA14	100.0%	100.0%	ADLELVARHTHSIRTYSVQGALGDIPALAEAFGLRVSLGIWLGPDLAGNEAEIARAIRIANESPSVVRVIVGNEALFRRE
2	*P. aeruginosa* 19BR	100.0%	99.2%	TDLELVARHTHSIRTYSVQGALGDIPALAEAFGLRVSLGIWLGPDLASNEAEIARAIRIANESPSVVRVIVGNEALFRRE
3	*P. aeruginosa* BL14	100.0%	99.8%	ADLELVARHTHSIRTYSVQGALGDIPALAEAFGLRVSLGIWLGPDLAGNEAEIARAIRIANESPSVVRVIVGNEALFRRE
4	*P. aeruginosa* 3573	100.0%	99.5%	ADLELVARHTHSIRTYSVQGALGDIPALAEAFGLRVSLGIWLGPDLAGNEAEIARAIRIANESPSVVRVIVGNEALFRRE
5	*P. aeruginosa* LESB58	100.0%	99.2%	TDLELVARHTHSIRTYSVQGALGDIPALAEAFGLRVSLGIWLGPDLASNEAEIARAIRIANESPSVVRVIVGNEALFRRE
6	***P. aeruginosa* PA01**	100.0%	99.4%	TDLELVARHTHSIRTYSVQGALGDIPALAEAFGLRVSLGIWLGPDLASNEAEIARAIRIANESPSVVRVIVGNEALFRRE
7	*P. aeruginosa* AZPAE12140	100.0%	99.5%	TDLELVARHTHSIRTYSVQGALGDIPALAEAFGLRVSLGIWLGPDLASNEAEIARAIRIANESPSVVRVIVGNEALFRRE
8	*P. aeruginosa* PAK	100.0%	99.4%	TDLELVARHTHSIRTYSVQGALGDIPALAEAFGLRVSLGIWLGPDLASNEAEIARAIRIANESPSVVRVIVGNEALFRRE
9	***P. fluorescens* SBW25**	99.0%	73.7%	RDLEIMSKLTDNIRTYSVDGTLGDIPKLAEEFGLRVTLGIWISPDLERNEREIQRAIEIANSSRSVVRVVVGNEALFREE
10	*P. fluorescens* SS101	99.3%	73.6%	QDLAIMSKLTDNIRTYSVDGPLGDIPKLAEEFGLRVTLGIWISPDLERNEREIQRAIEIANTSRSVVRVVVGNEALFREE
11	*P. fluorescens* ICMP 3512	99.3%	73.3%	RDLEIMSKLTDNIRTYSVDGTLGDIPKLAEEFGLRVTLGIWISPDLERNEREIQRAIELANSSRSVVRVVVGNEALFREE
12	*P. fluorescens* WS 5037	99.3%	73.8%	RDLEIMSKLTDNIRTYSVDGTLGDIPKLAEEFGLRVTLGIWISPDLERNEREIQRAIELANSSRSVVRVVVGNEALFREE
13	*P. fluorescens* ICMP 11288	99.3%	73.9%	QDLAIMSKLTDNIRTYSVDGTLGDIPKLAEEFGLRVTLGIWISPDLERNEREIQRAIEIANTSRSVVRVVVGNEALFREE
14	*P. fluorescens* WH6	99.3%	73.9%	QDLAIMSKLTDNIRTYSVDGTLGDIPKLAEEFGLRVTLGIWISPDLERNEREIQRAIEIANSSRSVVRVVVGNEALFREE
15	*P. fluorescens* KF1	99.3%	74.1%	RDLEIMSKLTDNIRTYSVDGTLGDIPKLAEEFGLRVTLGIWISPDLERNEREIQRAIEIANSSRSVVRVVVGNEALFREE
16	*P. fluorescens* LMG 5329	99.3%	73.7%	RDLEIMSKLTDNIRTYSVDGTLGDIPKLAEEFGLRVTLGIWISPDLERNEREIQRAIEIANSSRSVVRVVVGNEALFREE
17	*P. putida* S610	99.5%	75.7%	QDLEQMSKLTDSIRIYTVEGTQAHIPKLAEEFGLRVTVGIWISPDLERNEREIATAIELANTSRSVVRVVVGNEALFREE
18	*P. putida* W619	99.3%	76.4%	QDLEQMNKLTDSIRIYTVEGTQADIPRLAEELGLRVTLGIWISPDLERNEREIATAIELANTSRSVVRVVVGNEALFREE
19	***P. putida* KT2440**	99.3%	76.3%	QDLEQLSKLTDSIRIYTVEGTQADVPRLAEEFGLRVTLGIWISPDLERNEREIATAIQLANTSRSVVRVVVGNEALFREE
20	*P. putida* YKD221	99.3%	76.3%	QDLEQLSKLTDSIRIYTVEGTQADVPRLAEEFGLRVTLGIWISPDLERNEREIATAIQLANTSRSVVRVVVGNEALFREE
21	*P. syringae* ICMP 9617	95.3%	46.3%	EDLLLIKPFTQNIRTYSVEGTLAYIPEIAEELGMSVSLGAWISQDKARNAQELKTAIEITNRFSCVQRLMVGNEVLFRGD
22	*P. syringae* B728a	95.3%	46.1%	EDLLLLRSITRNIRTYSVEGTLMHIPELAEALGMNVTLGVWITEDETHNIEEINAGIELANRYSSVQRLVLGNEVLFRDD
23	*P. syringae* UMAF0158	95.0%	45.2%	EDLLRLRSITQNIRTYSVGGTLMHIPELAEALGMNVTLGVWITEDETHNIEEINAGIELANRYSSVQRLVLGNEVLFRDD
24	***P. syringae* DC3000**	95.3%	45.4%	EDLLLIRPLTRHVRTYSVEQTLACIPEIAEELGMSVTLGIWIGWDEKRNDRELIEGVKLANQYPSVRRLIIGNETLLRND
25	*P. syringae* NCPPB 4273	95.3%	47.3%	EDLLLIRPFTRHIRTYSVEGTLACIPEIAEALGMSVTLGVWITWDEKHNDRDLLTGVDLANRFSSVQRLLIGHEALLRND
26	*P. syringae* 41a	95.3%	46.9%	EDLLLIRPLTRHIRTYSVEGTLACIPEIAEALGMSVTLGVWITWDEKHNDRDLLTGVDLANRFSGVQRLLIGHEALLRND
	Consensus/100%			tDL..ht.hTpplRhYoVtts.h.lPtlAEthGhpVolGhWls.D.ttN.t-l.tulplsNp..sV.Rlhl GpEsLhRt-
	Consensus/70%			pDLtlhu+hTcsIRTYSVpGsLuDIPtLAEtFGLRVoLGIWluPDLtpNEtEItpAIclANpS.SVVRVlVGNEALFRcE
	Motifs & residues			D R Y A G W VGNE L
	First domain			

	Species	Coverage	& ID	 240
1	*P. aeruginosa* PA14	100.0%	100.0%	VTAEQLIAYLDRVRAAVKVPVTTAEQWHVYREHPELAQHVDLIAAHVLPYWEATPVADAVDFVLERARELKAAFPRKPLL
2	*P. aeruginosa* 19BR	100.0%	99.2%	VTAEQLIAYLDRVRAAVKVPVTTAEQWHVYREHPELAQHVDLIAAHVLPYWEATPVADAVDFVLERARELKAAFPRKPLL
3	*P. aeruginosa* BL14	100.0%	99.8%	VTAEQLIAYLDRVRAAVKVPVTTAEQWHVYREHPELAQHVDLIAAHVLPYWEATPVADAVDFVLERARELKAAFPRKPLL
4	*P. aeruginosa* 3573	100.0%	99.5%	VTAEQLIAYLDRVRAAVKVPVTTAEQWHVYREHPELAQHVDLIAAHVLPYWEATPVADAVDFVLERARELKAAFPRKPLL
5	*P. aeruginosa* LESB58	100.0%	99.2%	VTAEQLIAYLDRVRAAVKVPVTTAEQWHVYREYPELAQHVDLIAAHVLPYWEATPVADAVDFVLERARELKAAFPRKPLL
6	***P. aeruginosa* PA01**	100.0%	99.4%	VTAEQLIAYLDRVRAAVKVPVTTAEQWHVYREHPELAQHVDLIAAHVLPYWEATPVADAVDFVLERARELKAAFPRKPLL
7	*P. aeruginosa* AZPAE12140	100.0%	99.5%	VTAEQLIAYLDRVRAAVKVPVTTAEQWHVYREHPELAQHVDLIAAHVLPYWEATPVADAVDFVLERARELKAAFPRKPLL
8	*P. aeruginosa* PAK	100.0%	99.4%	VTAEQLIAYLDRVRAAVKVPVTTAEQWHVYREHPELAQHVDLIAAHVLPYWEATPVADAVDFVLERARELKAAFPRKPLL
9	***P. fluorescens* SBW25**	99.0%	73.7%	ITPEALIVLLDRVRAAVKVPVTTSEQWHIWEKNPQLAKHVDLIAAHILPFWEYIPMDKAGQYVLDRARDLKKLFPKKPLL
10	*P. fluorescens* SS101	99.3%	73.6%	ITPEALIVLLDRVRAAVKVPVTTSEQWHIWEKNPQLAKHVDLIAAHILPFWEYIPMDKAGQYVLDRAKDLKKAFPKKPLL
11	*P. fluorescens* ICMP 3512	99.3%	73.3%	ITPQALIVLLDRVRAAVKVPVTTSEQWHIWEKNPQLAKHVDLIAAHILPFWEFIPVDKAGQYVLDRAKDLKKLFPKKPLL
12	*P. fluorescens* WS 5037	99.3%	73.8%	ITPQALIVLLDRVRAAVKVPVTTSEQWHIWEKNPQLAKHVDLIAAHILPFWEFIPVDKAGQYVLDRAKDLKKLFPKKPLL
13	*P. fluorescens* ICMP 11288	99.3%	73.9%	ITPEALIVLLDRVRAAVKVPVTTSEQWHIWEKNPQLAKHVDLIAAHILPFWEFIPMDKAGQYVLDRARDLKKLFPKKPLL
14	*P. fluorescens* WH6	99.3%	73.9%	ITPEALIVLLDRVRAAVKVPVTTSEQWHIWEKNPQLAKHVDLIAAHILPFWEFIPMDKAGQYVLDRARDLKKLFPKKPLL
15	*P. fluorescens* KF1	99.3%	74.1%	ITPEALIVLLDRVRAAVKVPVTTSEQWHIWEKNPQLAKHVDLIAAHILPFWEYIPMDKAGQYVLDRARDLKKLFPKKPLL
16	*P. fluorescens* LMG 5329	99.3%	73.7%	ITPEALIVLLDRVRAAVKVPVTTSEQWHIWEKNPQLAKHVDLIAAHILPFWEYIPMDKAGQYVLDRARDLKKLFPKKPLL
17	*P. putida* S610	99.5%	75.7%	VTPENLIQYLDRVRAAVKVPVTTSEQWHIWKQNPQLAKHVDLIAAHILPYWEFVPMKDSVEFVLDRARELKHQFPRKPLL
18	*P. putida* W619	99.3%	76.4%	VTPEALIQYLDRVRAAVKVPVTTSEQWHIWKEHPELAKHVDLIAAHILPYWEFVPMKDSVEFVLDRARELKQQFPRKPLL
19	***P. putida* KT2440**	99.3%	76.3%	VTPENLIKYLDRVRAAVKVPVTTSEQWHIWKEHPELARHVDLIAAHILPYWEFVPMKDSVEFVLERARELKHQFPRKPLL
20	*P. putida* YKD221	99.3%	76.3%	VTPENLIKYLDRVRAAVKVPVTTSEQWHIWKEHPELARHVDLIAAHILPYWEFVPMKDSVEFVLDRARELKHQFPRKPLL
21	*P. syringae* ICMP 9617	95.3%	46.3%	LSPEQLIEHIKTARHSVKVPVATSDTWMQWLEAPELVEHSDFIAAHILPFWERFSAEEAASIVINQARQLQQEFPDKTLI
22	*P. syringae* B728a	95.3%	46.1%	VPIDLLIHYLQTARRAVNVPVSTSEIWTQWYETPDLVRHVDFIAAHILPFWEGVSALDATAITLAHANELRTRFPDTPLI
23	*P. syringae* UMAF0158	95.0%	45.2%	VPIDLLILYLQTARRAVNVPVSTSEIWTQWYETPELVRHVDFIAAHILPFWEGVSALDATAITLAHANELRTRFPDTPLI
24	***P. syringae* DC3000**	95.3%	45.4%	VTVSQLIDYMQTARQGVNVPISTSEGWQQWHDTPELADHADFIAAHVLPFREFVPVTQAGSAVLARANELRLMFPEKPLI
25	*P. syringae* NCPPB 4273	95.3%	47.3%	VTVDQLIAYMSTARRYVDVPVSTSEGWKQWHETPELAEHADFIAAHILPFKEAVPVTEASARVLARADELKLMFPDKPLI
26	*P. syringae* 41a	95.3%	46.9%	VTVDQLIAYMNTARRYVEVPVSTSEGWQQWHETPELAEHADFIAAHILPFKEAVPVTEASALVLARADELKLMFPDKPLI
	Consensus/100%			ls.p.LI.hhppsRt.VpVPlsTu-.W..a.p.PpLspHsDhIAAHlLPahEhhsh.pusthsltpAppLp..FPcpsLl
	Consensus/70%			lTsEtLIshLDRVRAAVKVPVTTuEQWHlaccpPpLApHVDLIAAHlLPaWEhhPhscAspaVL-RA+-LKthFP+KPLL
	Motifs & residues			---SD1--- ----SD2----
	First domain			

	Species	Coverage	ID	 320
1	*P. aeruginosa* PA14	100.0%	100.0%	LAEVGWPSNGRMRGSAEATPADQAIYLRRLTNALNGEGYSYFVIEAFDQPWKVSAEGSVGAYWGVYNADRKAKFNFTGPV
2	*P. aeruginosa* 19BR	100.0%	99.2%	LAEVGWPSNGRMRGSAEATPADQAIYLRRLTNALNGEGYSYFVIEAFDQPWKVSAEGSVGAYWGVYNADRKAKFNFTGPV
3	*P. aeruginosa* BL14	100.0%	99.8%	LAEVGWPSNGRMRGSAEATPADQAIYLRRLTNALNGEGYSYFVIEAFDQPWKVSAEGSVGAYWGVYNADRKAKFNFTGPV
4	*P. aeruginosa* 3573	100.0%	99.5%	LAEVGWPSNGRMRGSAEATPADQAIYLRRLTNALNGEGYSYFVIEAFDQPWKVSAEGSVGAYWGVYNADRKAKFNFTGPV
5	*P. aeruginosa* LESB58	100.0%	99.2%	LAEVGWPSNGRMRGSAEATPADQAIYLRRLTNALNGEGYSYFVIEAFDQPWKVSAEGSVGAYWGVYNADRKAKFNFTGPV
6	***P. aeruginosa* PA01**	100.0%	99.4%	LAEVGWPSNGRMRGSAEATPADQAIYLRRLTNALNGEGYSYFVIEAFDQPWKVSAEGSVGAYWGVYNADRKAKFNFTGPV
7	*P. aeruginosa* AZPAE12140	100.0%	99.5%	LAEVGWPSNGRMRGSAEATPADQAIYLRRLTNALNGEGYSYFVIEAFDQPWKVSAEGSVGAYWGVYNADRKAKFNFTGPV
8	*P. aeruginosa* PAK	100.0%	99.4%	LAEVGWPSNGRMRGSAEATPADQAIYLRRLTNALNGEGYSYFVIEAFDQPWKVSAEGSVGAYWGVYNADRKAKFNFTGPV
9	***P. fluorescens* SBW25**	99.0%	73.7%	LSEVGWPSNGRMRGGNETSPADQAIYLRTLVNKLNRQGFNYFVIEAFDQPWKVSDEGSAGAYWGVYNAARQQKFNFDGPV
10	*P. fluorescens* SS101	99.3%	73.6%	LSEVGWPSNGRMRGGNESSPADQAIYLRTLVNKLNRQGYNYFVIEAFDQPWKVSDEGSAGAYWGVFNAARQQKFNFDGPV
11	*P. fluorescens* ICMP 3512	99.3%	73.3%	LSEVGWPSNGRMRGGNETSPADQAVYLRTLVNKLNRQGYNYFVIEAFDQPWKVSDEGSAGAYWGVYNAARQQKFNFEGPV
12	*P. fluorescens* WS 5037	99.3%	73.8%	LSEVGWPSNGRMRGGNETSPADQAVYLRTLVNKLNRQGYNYFVIEAFDQPWKVSDEGSAGAYWGVYNAARQQKFNFEGPV
13	*P. fluorescens* ICMP 11288	99.3%	73.9%	LSEVGWPSNGRMRGGNESSPADQAIYLRTLVNKLNRQGYNYFVIEAFDQPWKVSDEGSAGAYWGVYNAARQQKFNFEGPV
14	*P. fluorescens* WH6	99.3%	73.9%	LSEVGWPSNGRMRGGNEASPADQAIYLRTLVNKLNRQGYNYFVIEAFDQPWKVSDEGSAGAYWGVFNAARQQKFNFEGPV
15	*P. fluorescens* KF1	99.3%	74.1%	LSEVGWPSNGRMRGGNESSPADQAIYLRTLVNKLNRQGYNYFVIEAFDQPWKVSDEGSAGAYWGVYNAARQQKFNFEGPV
16	*P. fluorescens* LMG 5329	99.3%	73.7%	LSEVGWPSNGRMRGGNESSPADQAIYLRTLVNKLNRQGFNYFVIEAFDQPWKVSDEGSAGAYWGVYNAARQQKFNFEGPV
17	*P. putida* S610	99.5%	75.7%	LSEVGWPSNGRMRGGADATQADQAIYLRTLVNTLNRRGFNYFVIEAYDQPWKASDEGSVGAYWGVFNAERQQKFNFEGPV
18	*P. putida* W619	99.3%	76.4%	LSEVGWPSNGRMRGGADATQADQAIYLRTLVNTLNRRGYNYFVIEAYDQPWKASDEGSVGAYWGVFNAERQQKFNFEGPV
19	***P. putida* KT2440**	99.3%	76.3%	LSEVGWPSNGRMRGGADATQADQAIYLRTLVNTLNRRGYNYFVIEAYDQPWKASDEGSVGAYWGVYNAERQQKFNFDGPV
20	*P. putida* YKD221	99.3%	76.3%	LSEVGWPSNGRMRGGADATQADQAIYLRTLVNTLNRRGYNYFVIEAYDQPWKASDEGSVGAYWGVYNAERQQKFNFDGPV
21	*P. syringae* ICMP 9617	95.3%	46.3%	LSEIGWPSQGNATRRASTTAAEQSIYLRTQISVLAQLDCPYFVIEAFDQPWKT-GEGTPGPHWGFFNSQRKLKLQLYGPV
22	*P. syringae* B728a	95.3%	46.1%	LSEIGWPSKAIAKRRMTTSDAEHSIYLRNQIPLLDQHGHDYFVIEAFDQHWKT-EEGLPGPNWGLFDAKRRLKLHVNGPV
23	*P. syringae* UMAF0158	95.0%	45.2%	LSEIGWPSKAIAKRRMTTSDAEHSIYLRNQIPLLDQHDHDYFVIEAFDQHWKT-EEGLPGPNWGLFDAKRRLKLHINGPV
24	***P. syringae* DC3000**	95.3%	45.4%	LSEIGWPDKGNFRRRTTAYVAEQSIYLRSQLALLNQSGLDYFVREAFDQQWKT-EEGLPGPHWGLFDAQRKIKLPLQGPV
25	*P. syringae* NCPPB 4273	95.3%	47.3%	ISEVGWPGKGNFRRRITTYRAEQSIYLRHQLALLEQHGHDYFVMEAFDQPWKT-SEGLPGPHWGLFDAQRKMKLQLKGPV
26	*P. syringae* 41a	95.3%	46.9%	ISEVGWPGKGNFRRRITTYRAEQSIYLRHQLSLLEQHGHDYFVMEAFDQPWKT-SEGLPGPHWGLFDARRNMKLQLKGPV
	Consensus/100%			luElGWPspu.hptt.ps..A-pulYLRp.hshLtt.shsYFVhEAaDQ.WKs.tEG.sGs.WGhasutRp.Kh.h.GPV
	Consensus/70%			LuEVGWPSNGRMRGus-sosADQAIYLRpLsNtLNtpGasYFVIEAFDQPWKsSsEGSsGAYWGVaNAtRptKFNFpGPV
	Motifs & residues			E GWP G G WK
	First domain			

	Species	Coverage	ID	 400
1	*P. aeruginosa* PA14	100.0%	100.0%	VPIPKWRALAIASAVLAVLAFTLLLIDSSSLRQRGRTFLAVVSFACASVLVWIAYDYSQQYSTWFSLTVGALLGVGALGV
2	*P. aeruginosa* 19BR	100.0%	99.2%	VPIPKWRALAIASAVLAVLAFTLLLIDSSSLRQRGRTFLAVVSFACASVLVWIAYDYSQQYSTWFSLTVGALLGVGALGV
3	*P. aeruginosa* BL14	100.0%	99.8%	VPIPKWRALAIVSAVLAVLAFTLLLIDSSSLRQRGRTFLAVVSFACASVLVWIAYDYSQQYSTWFSLTVGALLGVGALGV
4	*P. aeruginosa* 3573	100.0%	99.5%	VPIPKWRALAIASAVLAVLAFTLLLIDSSSLRQRGRTFLAVVSFACASVLVWIAYDYSQQYSTWFSLTVGALLGVGALGV
5	*P. aeruginosa* LESB58	100.0%	99.2%	VPIPKWRALAIASAVLAVLAFTLLLIDSSSLRQRGRTFLAVVSFACASVLVWIAYDYSQQYSTWFSLTVGALLGVGALGV
6	***P. aeruginosa* PA01**	100.0%	99.4%	VPIPKWRALAIASAVLAVLAFTLLLIDSSSLRQRGRTFLAVVSFACASVLVWIAYDYSQQYSTWFSLTVGALLGVGALGV
7	*P. aeruginosa* AZPAE12140	100.0%	99.5%	VPIPKWRALAIASAVLAVLAFTLLLIDSSSLRQRGRTFLAVVSFACASVLVWIAYDYSQQYSTWFSLTVGALLGVGALGV
8	*P. aeruginosa* PAK	100.0%	99.4%	VPIPKWRALAIASAVLAVLAFTLLLIDSSSLRQRGRTFLAVVSFACASVLVWIAYDYSQQYSTWFSLTVGALLGVGALGV
9	***P. fluorescens* SBW25**	99.0%	73.7%	VAIPQWRVLAIGSVVLALLSLTLLMIDGSSLRQRGRTFLTFIAFLCGSVLVWIGYDYSQQYSTWFSVTVGILLALGALGV
10	*P. fluorescens* SS101	99.3%	73.6%	VAIPQWRVLAIGSVVLALLSLTLLMIDGSALRQRGRTFLTFIAFLCGSVLVWIGYDYSQQYSTWFSVTVGVLLALGALGV
11	*P. fluorescens* ICMP 3512	99.3%	73.3%	VAIPQWRVLAIGSVVLALLSLTLLMIDGSALRQRGRTFLTFIAFLCGSVLVWIGYDYSQQYSTWFSVTVGILLALGALGV
12	*P. fluorescens* WS 5037	99.3%	73.8%	VAIPQWRVLAIGSVVLALLSLTLLMIDGSALRQRGRTFLTFIAFLCGSVLVWIGYDYSQQYSTWFSVTVGILLALGALGV
13	*P. fluorescens* ICMP 11288	99.3%	73.9%	VAIPQWRVLAIGSVVLALLSLTLLMIDGSALRQRGRTFLTFIAFLCGSVLVWIGYDYSQQYSTWFSVTVGILLALGALGV
14	*P. fluorescens* WH6	99.3%	73.9%	VAIPQWRVLAIGSVVLALLSLTLLMIDGSALRQRGRTFLTFIAFLCGSVLVWIGYDYSQQYSTWFSVTVGILLALGALGV
15	*P. fluorescens* KF1	99.3%	74.1%	VAIPQWRVLAIGSVVLALLSLTLLMIDGSALRQRGRTFLTFIAFLCGSVLVWIGYDYSQQYSTWFSVTVGILLALGALGV
16	*P. fluorescens* LMG 5329	99.3%	73.7%	VAIPQWRVLAIGSVVLALLSLTLLMIDGSALRQRGRTFLTFIAFLCGSVLVWIGYDYSQQYSTWFSVTVGILLALGALGV
17	*P. putida* S610	99.5%	75.7%	VAIPQWRTLAVASVVLAMIALMVLFIDGSALRQRGRTFLTFITFLCGSVLVWIAYDYSQQYSTWFSLTVGVLLALGALGV
18	*P. putida* W619	99.3%	76.4%	VAIPQWRALAVASVVLAMIALTVLLIDGSALRQRGRTFLTFITFLCGSVLVWIGYDYSQQYSTWFSLTVGVLLALGALGV
19	***P. putida* KT2440**	99.3%	76.3%	VAIPQWRALAVASVVLAMIALMVLFIDGSALRQRGRTFLTFITFLCGSVLVWIAYDYSQQYSTWFSLTVGVLLALGALGV
20	*P. putida* YKD221	99.3%	76.3%	VAIPQWRALAVASVVLAMIALMVLFIDGSALRQRGRTFLTFITFLCGSVLVWIAYDYSQQYSTWFSLTVGVLLALGALGV
21	*P. syringae* ICMP 9617	95.3%	46.3%	NAPIRWRSTLLNSVI--------------RLRPDSSRVTLAVTVALICALIIILALEYSQSLPLWITMPVSVLWAT---CL
22	*P. syringae* B728a	95.3%	46.1%	KMPVNMLSEILRLIT--------------RLKPESWPAGALIIVLAYCALSGFGMHYSQPLPAWLALPVVFIWAA---SV
23	*P. syringae* UMAF0158	95.0%	45.2%	KIPVNMLSEILRLIT--------------RLKPESWPTGALIIVLAYCVLSGFGMHYSQPLPAWLALPVAFI-AA---SV
24	***P. syringae* DC3000**	95.3%	45.4%	KIRASWRSEVPRLVA--------------DWQPDNWRTTVLIFAALYTLLVGVGISYAQPLSMWVALPIALVWVT---SL
25	*P. syringae* NCPPB 4273	95.3%	47.3%	KTRASWQSEIPRVIA--------------GLRPDAWRTTAVMCAVAYAVLVWVGMSYAQPLSAWIALPIALAWAT---GI
26	*P. syringae* 41a	95.3%	46.9%	KTRASWQSEIPRIIA--------------GLQPDAWRTTAVMCAVAYAVLVWVGMSYAQPLSAWIALPIALAWAT---CL
	Consensus/100%			....ph.s.h...hh..............thp.cs..hhhhh.hhhhshL.hhuhpYuQ.hshWhshslshh.sh...sl
	Consensus/70%			VsIPpWRsLAluSsVLAhluhhlLhIDuSuLRQRGRTFLshlsFhCuSVLVWIuYDYSQQYSTWFSlTVGhLLulGALGV

	Species	Coverage & ID		 480
1	*P. aeruginosa* PA14	100.0%	100.0%	VIVLFTEAHELAEAVWTRKRRRPFLPITAAQAYRPKVSIHVPCYNEPPELLKQTLDALARLDYPDYEVLVIDNNTRDPAV
2	*P. aeruginosa* 19BR	100.0%	99.2%	VIVLFTEAHELAEAVWTRKRRRPFLPITAAQAYRPKVSIHVPCYNEPPELLKQTLDALARLDYPDYEVLVIDNNTRNPAV
3	*P. aeruginosa* BL14	100.0%	99.8%	VIVLFTEAHELAEAVWTRKRRRPFLPITAAQAYRPKVSIHVPCYNEPPELLKQTLDALARLDYPDYEVLVIDNNTRDPAV
4	*P. aeruginosa* 3573	100.0%	99.5%	VIVLFTEAHELAEAVWTRKRRRPFLPITAAQAYRPKVSIHVPCYNEPPELLKQTLDALARLDYPDYEVLVIDNNTRDPAV
5	*P. aeruginosa* LESB58	100.0%	99.2%	VIVLFTEAHELAEAVWTRKRRRPFLPITAAQAYRPKVSIHVPCYNEPPELLKQTLDALARLDYPDYEVLVIDNNTRDPAV
6	***P. aeruginosa* PA01**	100.0%	99.4%	VIVLFTEAHELAEAVWTRKRRRPFLPITAARAYRPKVSIHVPCYNEPPELLKQTLDALARLDYPDYEVLVIDNNTRDPAV
7	*P. aeruginosa* AZPAE12140	100.0%	99.5%	VIVLFTEAHELAEAVWTRKRRRPFLPITAAQAYRPKVSIHVPCYNEPPELLKQTLDALARLDYPDYEVLVIDNNTRDPAV
8	*P. aeruginosa* PAK	100.0%	99.4%	VIVLFTEAHELAEAVWTRKRRRPFLPITAAQAYRPKVSIHVPCYNEPPELLKQTLDALARLDYPDYEVLVIDNNTRDPAV
9	***P. fluorescens* SBW25**	99.0%	73.7%	FIVLLTEAHELAEAVWTHKRRREFLPVEGDSDY-PKVSIHVPCYNEPPEMVKQTLDALAALDYPDYEVLIIDNNTKDPAV
10	*P. fluorescens* SS101	99.3%	73.6%	FIVLLTEAHELAEAVWTHKRRREFLPVEGDSDYRPKVSIHVPCYNEPPEMVKQTLDALAALDYPDYEVLIIDNNTKDPAV
11	*P. fluorescens* ICMP 3512	99.3%	73.3%	FIVLLTEAHELAEAVWTHKRRREFLPVEGDSDYRPKVSIHVPCYNEPPEMVKQTLDALAALDYPDYEVLIIDNNTKDPAV
12	*P. fluorescens* WS 5037	99.3%	73.8%	FIVLLTEAHELAEAVWTHKRRREFLPVEGDSDYRPKVSIHVPCYNEPPEMVKQTLDALAALDYPDYEVLIIDNNTKDPAV
13	*P. fluorescens* ICMP 11288	99.3%	73.9%	FIVLLTEAHELAEAVWTHKRRREFLPVEGDSHYRPKVSIHVPCYNEPPEMVKQTLDALAALDYPDFEVLIIDNNTKDPAV
14	*P. fluorescens* WH6	99.3%	73.9%	FIVLLTEAHELAEAVWTHKRRREFLPVEGDSDYRPKVSIHVPCYNEPPEMVKQTLDALAALDYPDYEVLIIDNNTKDPAV
15	*P. fluorescens* KF1	99.3%	74.1%	FIVLLTEAHELAEAVWTHKRRREFLPVVGDSDYRPKVSIHVPCYNEPPEMVKQTLDALAALDYPDYEVLIIDNNTKDPAV
16	*P. fluorescens* LMG 5329	99.3%	73.7%	FIVLLTEAHELAEAVWTHKRRREFLPVEGDSDYRPKVSIHVPCYNEPPEMVKQTLDALAALDYPDYEVLIIDNNTKDPAV
17	*P. putida* S610	99.5%	75.7%	FIVLLTEAHELAEAVWTHKRRREFLPVHGDSAYRPKVSVHVPCYNEPPEMVKQTLDALAALDYPDYEVLVIDNNTKDPAV
18	*P. putida* W619	99.3%	76.4%	FIVLLTEAHELAEAAWTHKRRREFLPVQADSAYRPKVSVHVPCYNEPPEMVKQTLDALAALDYPDYEVLVIDNNTKDPAV
19	***P. putida* KT2440**	99.3%	76.3%	FIVLLTEAHELAEAVWIHKRRREFLPVQADTAYRPKVSVHVPCYNEPPEMVKQTLDALAALDYPDYEVLVIDNNTKDPAV
20	*P. putida* YKD221	99.3%	76.3%	FIVLLTEAHELAEAVWIHKRRREFLPVQADSAYRPKVSVHVPCYNEPPEMVKQTLDALAALDYPDYEVLVIDNNTKDPAV
21	*P. syringae* ICMP 9617	95.3%	46.3%	LAGIAIESHEFLEAIWGPEQPRMFLPARFKYDQAPKVSLHVPCYDEPPDMVKRTLDALQNLDYPNFEVLVIDNNTPDRAT
22	*P. syringae* B728a	95.3%	46.1%	LTYMGIETHEFLEACWGPDTPRSFSPVHRPVGPAPKVSLHVPCYNEPPDMVKRTLDSLQTLDYPDFEVLVIDNNTQDPAI
23	*P. syringae* UMAF0158	95.0%	45.2%	LTYVGIETHEFLEACWGPDTPRSFRPVRRPVGPAPKVSLHVPCCNEPPDMVKRTLDSLQKLDYPDFEVLVIDNNTQDPAI
24	***P. syringae* DC3000**	95.3%	45.4%	LIGTGIQGYEFLESCWGPEKPRSFPPLRAYPGPLPKVSIHVPCYNEPPDMVKLTLDALQRLDYPNFEVLIIDNNTQDPEV
25	*P. syringae* NCPPB 4273	95.3%	47.3%	MIVVGTQGYEFLESFWGAENPRSFPPTRAYPGPWPKVSIHVPCYNEPPDMVKLTLDALQKLDYPDFEVLVIDNNTQDPNV
26	*P. syringae* 41a	95.3%	46.9%	MIVVVTQGYEFLESFWGAENPRSFPPTRAYPGPWPKVSIHVPCYNEPPDMVKLTLDALQKLDYPDFEVLVIDNNTQDPNV
	Consensus/100%			hhhhhhpsaEhhEuhWh.cp.R.F.Ph.h..t..PKVSlHVPChsEPP-hlK.TLDuLttLDYPsaEVLlIDNNT.s.th
	Consensus/70%			hIVLhTEAHELAEAVWs+KRRR.FLPlpuspsYRPKVSIHVPCYNEPPEhlKQTLDALAtLDYPDYEVLlIDNNT+DPAV
	Motifs & residues			D
	Second domain			

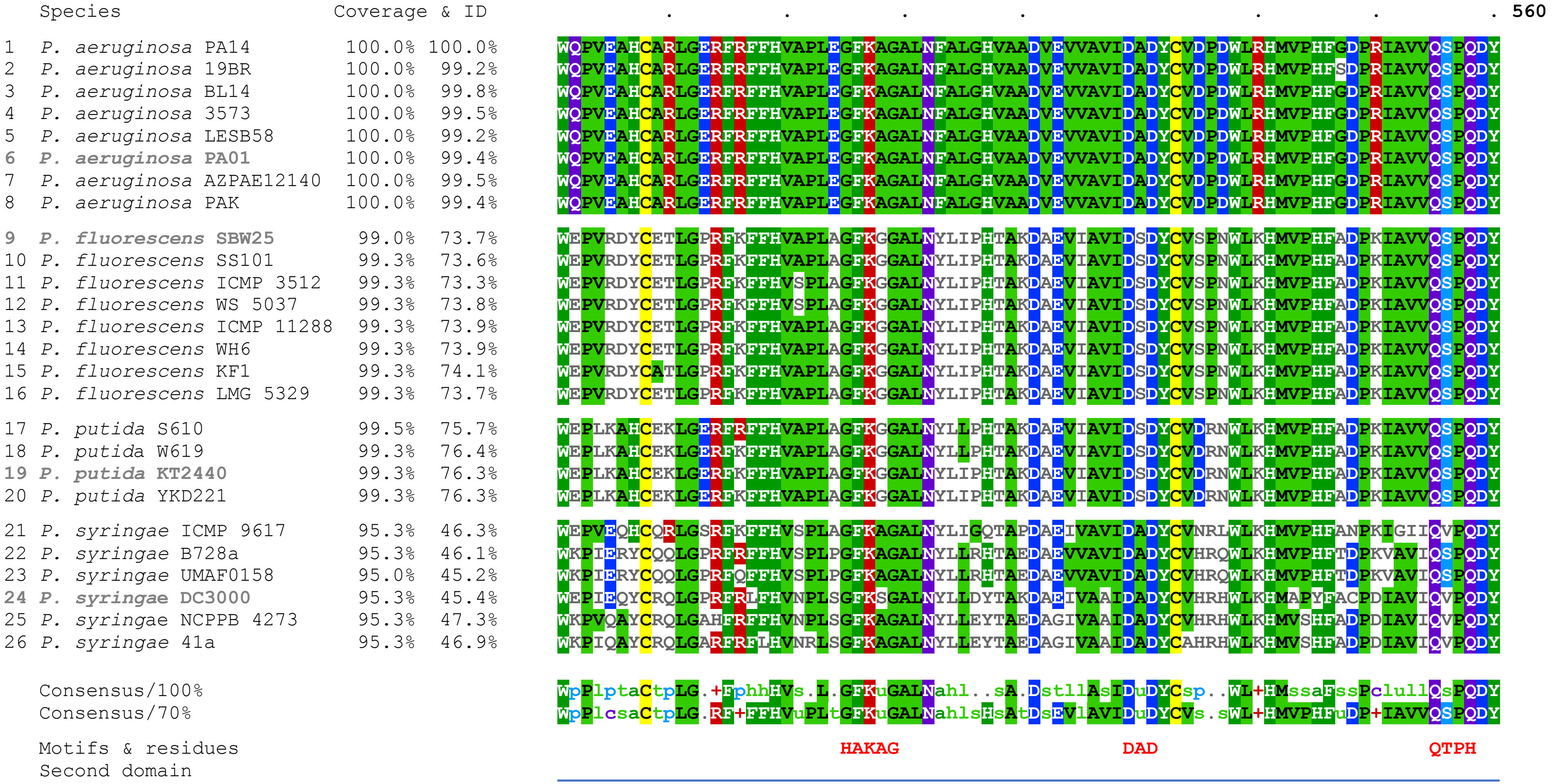
Species
Coverage & ID
560
1 P. aeruginosa PA14 100.0% 100.0% WQPVEAHCARLGERFRFFHVAPLEGFKAGALNFALGHVAADVEVVAVIDADYCVDPDWLRHMVPHFGDPRIAVVQSPQDY
2 P. aeruginosa 19BR 100.0% 99.2% WQPVEAHCARLGERFRFFHVAPLEGFKAGALNFALGHVAADVEVVAVIDADYCVDPDWLRHMVPHFSDPRIAVVQSPQDY
3 P. aeruginosa BL14 100.0% 99.8% WQPVEAHCARLGERFRFFHVAPLEGFKAGALNFALGHVAADVEVVAVIDADYCVDPDWLRHMVPHFGDPRIAVVQSPQDY
4 P. aeruginosa 3573 100.0% 99.5% WQPVEAHCARLGERFRFFHVAPLEGFKAGALNFALGHVAADVEVVAVIDADYCVDPDWLRHMVPHFGDPRIAVVQSPQDY
5 P. aeruginosa LESB58 100.0% 99.2% WQPVEAHCARLGERFRFFHVAPLEGFKAGALNFALGHVAADVEVVAVIDADYCVDPDWLRHMVPHFGDPRIAVVQSPQDY
6 P. aeruginosa PA01 100.0% 99.4% WQPVEAHCARLGERFRFFHVAPLEGFKAGALNFALGHVAADVEVVAVIDADYCVDPDWLRHMVPHFGDPRIAVVQSPQDY
7 P. aeruginosa AZPAE12140 100.0% 99.5% WQPVEAHCARLGERFRFFHVAPLEGFKAGALNFALGHVAADVEVVAVIDADYCVDPDWLRHMVPHFGDPRIAVVQSPQDY
8 P. aeruginosa PAK 100.0% 99.4% WQPVEAHCARLGERFRFFHVAPLEGFKAGALNFALGHVAADVEVVAVIDADYCVDPDWLRHMVPHFGDPRIAVVQSPQDY
9 P. fluorescens SBW25 99.0% 73.7% WEPVRDYCETLGPRFKFFHVAPLAGFKGGALNYLIPHTAKDAEVIAVIDSDYCVSPNWLKHMVPHFADPKIAVVQSPQDY
10 P. fluorescens SS101 99.3% 73.6% WEPVRDYCETLGPRFKFFHVAPLAGFKGGALNYLIPHTAKDAEVIAVIDSDYCVSPNWLKHMVPHFADPKIAVVQSPQDY
11 P. fluorescens ICMP 3512 99.3% 73.3% WEPVRDYCETLGPRFKFFHVSPLAGFKGGALNYLIPHTAKDAEVIAVIDSDYCVSPNWLKHMVPHFADPKIAVVQSPQDY
12 P. fluorescens WS 5037 99.3% 73.8% WEPVRDYCETLGPRFKFFHVSPLAGFKGGALNYLIPHTAKDAEVIAVIDSDYCVSPNWLKHMVPHFADPKIAVVQSPQDY
13 P. fluorescens ICMP 11288 99.3% 73.9% WEPVRDYCETLGPRFKFFHVAPLAGFKGGALNYLIPHTAKDAEVIAVIDSDYCVSPNWLKHMVPHFADPKIAVVQSPQDY
14 P. fluorescens WH6 99.3% 73.9% WEPVRDYCETLGPRFKFFHVAPLAGFKGGALNYLIPHTAKDAEVIAVIDSDYCVSPNWLKHMVPHFADPKIAVVQSPQDY
15 P. fluorescens KF1 99.3% 74.1% WEPVRDYCATLGPRFKFFHVAPLAGFKGGALNYLIPHTAKDAEVIAVIDSDYCVSPNWLKHMVPHFADPKIAVVQSPQDY
16 P. fluorescens LMG 5329 99.3% 73.7% WEPVRDYCETLGPRFKFFHVAPLAGFKGGALNYLIPHTAKDAEVIAVIDSDYCVSPNWLKHMVPHFADPKIAVVQSPQDY
17 P. putida S610 99.5% 75.7% WEPLKAHCEKLGERFRFFHVAPLAGFKGGALNYLLPHTAKDAEVIAVIDSDYCVDRNWLKHMVPHFADPKIAVVQSPQDY
18 P. putida W619 99.3% 76.4% WEPLKAHCEKLGERFKFFHVAPLAGFKGGALNYLLPHTAKDAEVIAVIDSDYCVDRNWLKHMVPHFADPKIAVVQSPQDY
19 P. putida KT2440 99.3% 76.3% WEPLKAHCEKLGERFKFFHVAPLAGFKGGALNYLIPHTAKDAEVIAVIDSDYCVDRNWLKHMVPHFADPKIAVVQSPQDY
20 P. putida YKD221 99.3% 76.3% WEPLKAHCEKLGERFKFFHVAPLAGFKGGALNYLIPHTAKDAEVIAVIDSDYCVDRNWLKHMVPHFADPKIAVVQSPQDY
21 P. syringae ICMP 9617 95.3% 46.3% WEPVEQHCQRLGSRFKFFHVSPLAGFKAGALNYLIGQTAPDAEIVAVIDADYCVNRLWLKHMVPHFANPKIGIIQVPQDY
22 P. syringae B728a 95.3% 46.1% WKPIERYCQQLGPRFRFFHVSPLPGFKAGALNYLLRHTAEDAEVVAVIDADYCVHRQWLKHMVPHFTDPKVAVIQSPQDY
23 P. syringae UMAF0158 95.0% 45.2% WKPIERYCQQLGPRFQFFHVSPLPGFKAGALNYLLRHTAEDAEVVAVIDADYCVHRQWLKHMVPHFTDPKVAVIQSPQDY
24 P. syringae DC3000 95.3% 45.4% WEPIEQYCRQLGPRFRLFHVNPLSGFKSGALNYLLDYTAKDAEIVAAIDADYCVHRHWLKHMAPYFACPDIAVIQVPQDY
25 P. syringae NCPPB 4273 95.3% 47.3% WKPVQAYCRQLGAHFRFFHVNPLSGFKAGALNYLLEYTAEDAGIVAAIDADYCVHRHWLKHMVSHFADPDIAVIQVPQDY
26 P. syringae 41a 95.3% 46.9% WKPIQAYCRQLGARFRFLHVNRLSGFKAGALNYLLEYTAEDAGIVAAIDADYCAHRHWLKHMVSHFADPDIAVIQVPQDY
Consensus/100% WpPlptaCtpLG.+EphhHVs.L.GFKuGALNahl..sA.DstllAsIDuDYCsp..WL+HMssaFssPclullQsPQDY
Consensus/70% WpPlcsaCtpLG.RF+FFHVuPLtGFKuGALNahlsHsAtDsEVlAVIDuDYCVs.sWL+HMVPHFuDP+IAVVQSPQDY
Motifs & residues
HAKAG
DAD
QTPH
Second domain

	Species	Coverage	ID	 640
1	*P. aeruginosa* PA14	100.0%	100.0%	RDQHESAFKRLCYAEYKGFFHIGMVTRNDRDAIIEHGTMTMIRRSVLDELRWPEWCITEDAELGLRVFEKGLSAAYFERS
2	*P. aeruginosa* 19BR	100.0%	99.2%	RDQHESAFKRLCYAEYKGFFHIGMVTRNDRDAIIEHGTMTMIRRSVLDELRWPEWCITEDAELGLRVFEKGLSAAYFERS
3	*P. aeruginosa* BL14	100.0%	99.8%	RDQHESAFKRLCYAEYKGFFHIGMVTRNDRDAIIEHGTMTMIRRSVLDELRWPEWCITEDAELGLRVFEKGLSAAYFERS
4	*P. aeruginosa* 3573	100.0%	99.5%	RDQHESAFKRLCYAEYKGFFHIGMVTRNDRDAIIEHGTMTMIRRSVLDELRWPEWCITEDAELGLRVFEKGLSAAYFERS
5	*P. aeruginosa* LESB58	100.0%	99.2%	RDQHESAFKRLCYAEYKGFFHIGMVTRNDRDAIIEHGTMTMIRRSVLDELRWPEWCITEDAELGLRVFEKGLSAAYFERS
6	***P. aeruginosa* PA01**	100.0%	99.4%	RDQHESAFKRLCYAEYKGFFHIGMVTRNDRDAIIEHGTMTMIRRSVLDELRWPEWCITEDAELGLRVFEKGLSAAYFERS
7	*P. aeruginosa* AZPAE12140	100.0%	99.5%	RDQHESAFKRLCYAEYKGFFHIGMVTRNDRDAIIEHGTMTMIRRSVLDELRWPEWCITEDAELGLRVFEKGLSAAYFERS
8	*P. aeruginosa* PAK	100.0%	99.4%	RDQHESAFKRLCYAEYKGFFHIGMVTRNDRDAIIEHGTMTMIRRSVLDELRWPEWCITEDAELGLRVFEKGLSAAYFERS
9	***P. fluorescens* SBW25**	99.0%	73.7%	RDQNESTFKKLCYAEYKGFFHIGMVTRNDRDAIIQHGTMTMTRRSVLEELGWA-WCICEDAELGLRVFEKGLSAAYYHDS
10	*P. fluorescens* SS101	99.3%	73.6%	RDQNESTFKKLCYAEYKGFFHIGMVTRNDRDAIIQHGTMTMTRRSVLEELGWADWCICEDAELGLRVFEKGLSAAYYHDS
11	*P. fluorescens* ICMP 3512	99.3%	73.3%	RDQNESTFKKLCYAEYKGFFHIGMVTRNDRDAIIQHGTMTMTRRSVLEELGWADWCICEDAELGLRVFEKGLSAAYYHDS
12	*P. fluorescens* WS 5037	99.3%	73.8%	RDQNESTFKKLCYAEYKGFFHIGMVTRNDRDAIIQHGTMTMTRRSVLEELGWADWCICEDAELGLRVFEKGLSAAYYHDS
13	*P. fluorescens* ICMP 11288	99.3%	73.9%	RDQNESTFKKLCYAEYKGFFHIGMVTRNDRDAIIQHGTMTMTRRSVLEELGWADWCICEDAELGLRVFEKGLSAAYYHDS
14	*P. fluorescens* WH6	99.3%	73.9%	RDQNESTFKKLCYAEYKGFFHIGMVTRNDRDAIIQHGTMTMTRRSVLEELGWADWCICEDAELGLRVFEKGLSAAYYHDS
15	*P. fluorescens* KF1	99.3%	74.1%	RDQNESTFKKLCYAEYKGFFHIGMVTRNDRDAIIQHGTMTMTRRSVLEELGWADWCICEDAELGLRVFEKGLSAAYYHDS
16	*P. fluorescens* LMG 5329	99.3%	73.7%	RDQNESTFKKLCYAEYKGFFHIGMVTRNDRDAIIQHGTMTMTRRSVLEELGWADWCICEDAELGLRVFEKGLSAAYYHDS
17	*P. putida* S610	99.5%	75.7%	RDQHESAFKKLCYSEYKGFFHIGMVTRNDRDAIIQHGTMTMTRRSVLDELGWAEWCICEDAELGLRVFEKGLSAAYAHNS
18	*P. putida* W619	99.3%	76.4%	RDQHESAFKKLCYSEYKGFFHIGMVTRNDRDAIIQHGTMTMTRRTVLEELGWAEWCICEDAELGLRVFEKGLSAAYAHNS
19	***P. putida* KT2440**	99.3%	76.3%	RDQHESAFKKLCYSEYKGFFHIGMVTRNDRDAIIQHGTMTMTRRSVLEELGWAEWCICEDAELGLRVFEKGLSAAYAHNS
20	*P. putida* YKD221	99.3%	76.3%	RDQHESAFKKLCYSEYKGFFHIGMVTRNDRDAIIQHGTMTMTRRSVLEELGWAEWCICEDAELGLRVFEKGLSAAYAHNS
21	*P. syringae* ICMP 9617	95.3%	46.3%	SDGDKNLFKYCCHAEYKGFFEVGMVIRNDHDAIIQHGTMTLIRRSALDRLGWAQWCICEDAELGLRMLENGFSTGYTPLS
22	*P. syringae* B728a	95.3%	46.1%	RDGHESLFKCCCQAEYRGFFNIGMVIRNDHDAIIQHGTMTLIRRSALDRLGWAEWCICEDAELGLRMLESGFSTGYAAIS
23	*P. syringae* UMAF0158	95.0%	45.2%	RDGHESLFKYCCQAE-QGFFNIGMVIRNDHDAIIQHGTMTLIRRSALDRLGWAEWCICEDAELGLRMLESGFSTGYAAIS
24	***P. syringae* DC3000**	95.3%	45.4%	RDGDDSLFKRCCQAEYRVFFNIGMVIRNDHDAIIQHGTMTLIRNSVLQRLRWAEWSICEDAELGLRILENGFSTGYVAIS
25	*P. syringae* NCPPB 4273	95.3%	47.3%	RDGDESLFKRYCEAEYRVFFNIGMVIRNDHDAIIQHGTMTLIRKSVLQRLRWAEWCICEDAELGLRILEHGFSTGYAAIS
26	*P. syringae* 41a	95.3%	46.9%	RDGDESLFKRYCEAEYRVFFNIGMVIRNDHDAIIQHGTMTLIRKSVLQRLRWAEWCICEDAELGLRILEHGFSTGYAAIS
	Consensus/100%			pDtpcshFKhhC.uE.psFFplGMVhRND+DAIIpHGTMThhRposLpcLtWs.WsIsEDAELGLRhhEpGhSsuYh..S
	Consensus/70%			RDQpESsFK+LCYAEYKGFFHIGMVTRNDRDAIIpHGTMTMhRRSVL-ELtWs-WCIsEDAELGLRVFEKGLSAAYhcpS
	Motifs & residues			FFCGS TED
	Second domain			

```
   Species                       Coverage & ID     .       .       .       .       .       .       .       . 720

1  P. aeruginosa PA14            100.0% 100.0%   YGKGVMPDTFIDFKKQRFRWAYGAIQIMKRHTDALLRGRGPDGSRLTRGQRYHFVAGWLPWIADGLNIFFTLGALLWSAA
2  P. aeruginosa 19BR            100.0%  99.2%   YGKGVMPDTFIDFKKQRFRWAYGAIQIMKRHTDALLRGRGLDGSRLTRGQRYHFVAGWLPWIADGLNIFFTLGALLWSAA
3  P. aeruginosa BL14            100.0%  99.8%   YGKGVMPDTFIDFKKQRFRWAYGAIQIMKRHTDALLRGRGPDGSRLTRGQRYHFVAGWLPWIADGLNIFFTLGALLWSAA
4  P. aeruginosa 3573            100.0%  99.5%   YGKGVMPDTFIDFKKQRFRWAYGAIQIMKRHTDALLRGRSPDGSRLTRGQRYHFVAGWLPWIADGLNIFFTLGALLWSAA
5  P. aeruginosa LESB58          100.0%  99.2%   YGKGVMPDTFIDFKKQRFRWAYGAIQIMKRHTDALLRGRGPDGSRLTRGQRYHFVAGWLPWIADGLNIFFTLGALLWSAA
6  P. aeruginosa PA01            100.0%  99.4%   YGKGVMPDTFIDFKKQRFRWAYGAIQIMKRHTDALLRGRGPDGSRLTRGQRYHFVAGWLPWIADGLNIFFTLGALLWSAA
7  P. aeruginosa AZPAE12140      100.0%  99.5%   YGKGVMPDTFIDFKKQRFRWAYGAIQIMKRHTDALLRGRGPDGSRLTRGQRYHFVAGWLPWIADGLNIFFTLGALLWSAA
8  P. aeruginosa PAK             100.0%  99.4%   YGKGVMPDTFIDFKKQRFRWAYGAIQIMKRHTDALLRGRGPDGSRLTRGQRYHFVAGWLPWIADGLNIFFTLGALLWSAA

9  P. fluorescens SBW25           99.0%  73.7%   YGKGLMPDTFIDFKKQRFRWAYGAIQIIKRHTASLLRGKG---TELTRGQRYHFLAGWLPWVADGMNIFFTVGALLWSAA
10 P. fluorescens SS101           99.3%  73.6%   YGKGLMPDTFIDFKKQRFRWAYGAIQIIKRHTASLIRGKD---TELTRGQRYHFLAGWLPWVADGMNIFFTVGALLWSAA
11 P. fluorescens ICMP 3512       99.3%  73.3%   YGKGLMPDTFIDFKKQRFRWAYGAIQIIKRHTASLLRGKG---TELTRGQRYHFLAGWLPWVADGMNIFFTVGALLWSAA
12 P. fluorescens WS 5037         99.3%  73.8%   YGKGLMPDTFIDFKKQRFRWAYGAIQIIKRHTASLLRGKG---TELTRGQRYHFLAGWLPWVADGMNIFFTVGALLWSAA
13 P. fluorescens ICMP 11288      99.3%  73.9%   YGKGLMPDTFIDFKKQRFRWAYGAIQIIKRHTASLLRGKG---TELTRGQRYHFLAGWLPWVADGMNIFFTVGALLWSAA
14 P. fluorescens WH6             99.3%  73.9%   YGKGLMPDTFIDFKKQRFRWAYGAIQIIKRHTASLLRGKD---TELTRGQRYHFLAGWLPWVADGMNIFFTVGALLWSAA
15 P. fluorescens KF1             99.3%  74.1%   YGKGLMPDTFIDFKKQRFRWAYGAIQIIKRHTASLLRGKD---TELTRGQRYHFLAGWLPWVADGMNIFFTVGALLWSAA
16 P. fluorescens LMG 5329        99.3%  73.7%   YGKGLMPDTFIDFKKQRFRWAYGAIQIIKRHTASLLRGKD---TELTRGQRYHFLAGWLPWVADGMNIFFTVGALLWSAA

17 P. putida S610                99.5%  75.7%   YGKGLMPDTFIDFKKQRFRWAYGAIQIIKHHAAALLRGKG---SELTRGQRYHFLAGWLPWIADGMNIFFTVGALLWSAA
18 P. putida W619                99.3%  76.4%   YGKGLMPDTFIDFKKQRFRWAYGAIQIIKHHAAALLRGKG---SELTRGQRYHFLAGWLPWVADGMNIFFTVGALLWSAA
19 P. putida KT2440              99.3%  76.3%   YGKGLMPDTFIDFKKQRFRWAYGAIQIIKHHAGALLRGKG---SQLTRGQRYHFLAGWLPWIADGMNIFFTIGALLWSAA
20 P. putida YKD221              99.3%  76.3%   YGKGLMPDTFIDFKKQRFRWAYGAIQIIKHHAGALLRGKG---SQLTRGQRYHFLAGWLPWIADGMNIFFTIGALLWSAA

21 P. syringae ICMP 9617         95.3%  46.3%   YGKGLTPDTFNDFKKQRFRWAYGAVQIVKQHSWSLIAGRS---EALSTMQRYHFLAGWVPWAAEGVNYLLVFATLLWSAA
22 P. syringae B728a             95.3%  46.1%   YGKGLTPDTFMDFKKQRYRWAYGAMQIVKRHAGSLIAGNC---ASLSAMQRYHFIAGWMPWVAEGMNYLLTLAALAWSMA
23 P. syringae UMAF0158          95.0%  45.2%   YGKGLTPDTFMDFKKQRYRWAYGAMQIVKRHAGSLIAGNC---ASLSAMQRYHFIAGWMPWVAEGMNYLLTLAALAWSMA
24 P. syringae DC3000            95.3%  45.4%   YGKGLIPDTFMDFKKQRYRWAYGVIQILKRHTGSLIAGTC---EALTPIQRYHFIAGWMPWIAGGINYFLAIAVLLWSMA
25 P.s syringae NCPPB 4273        95.3%   47.3%   YGKGLMPDTFMDFKKQRYRWTYGAMQIFKRHAASLLAGTG---TALTPVQRYYFIAGWVPWMAGGVNYFLALAVLLWSMA
26 P. syringae 41a               95.3%  46.9%   YGKGLMPDTFMDFKKQRYRWTYGAMQIFKRHAGSLLAGTG---TALTPVQRYYFIAGWVPWMAGGVNYFLALAVLLWSMA

   Consensus/100%                                YGKGlhPDTF.DFKKQRaRWsYGshQIhKpHs.uLltGps...ttLo.hQRYaFlAGWhPWhAtGhNhhhshusLhWShA
   Consensus/70%                                 YGKGlMPDTFIDFKKQRFRWAYGAIQIhKRHssuLLRG+u...opLTRGQRYHFlAGWLPWlADGhNIFFTlGALLWSAA

   Motifs & residues                                     E        QR RW
   Second domain
```

	Species	Coverage	& ID	 800
1	*P. aeruginosa* PA14	100.0%	100.0%	MIIVPKRVDPPLLIFAILPLALFAFKVGKILFLYRRTVGVDLRDSFFAALAGLSLSHTIAKAVLYGFVTRGIPF-RTPKM
2	*P. aeruginosa* 19BR	100.0%	99.2%	MIIVPKRVDPPLLIFAILPLALFAFKVGKILFLYRRTVGVDLRDSFFAALAGLSLSHTIAKAVLYGFVTRGIPFFRTPKM
3	*P. aeruginosa* BL14	100.0%	99.8%	MIIVPKRVDPPLLIFAILPLALFAFKVGKILFLYRRTVGVDLRDSFFAALAGLSLSHTIAKAVLYGFVTRGIPFFRTPKM
4	*P. aeruginosa* 3573	100.0%	99.5%	MIIVPKRVDPPLLIFAILPLALFVFKVGKILFLYRRTVGVDLRDSFFAALAGLSLSHTIAKAVLYGFVTRGIPFFRTPKM
5	*P. aeruginosa* LESB58	100.0%	99.2%	MIIVPKRVDPPLLIFAILPLALFVFKVGKILFLYRRTVGVDLRDSFFAALAGLSLSHTIAKAVLYGFVTRGIPFFRTPKM
6	***P. aeruginosa*** **PA01**	100.0%	99.4%	MIIVPKRVDPPLLIFAILPLALFVFKVGKILFLYRRTVGVDLRDSFFAALAGLSLSHTIAKAVLYGFVTRGIPFFRTPKM
7	*P. aeruginosa* AZPAE12140	100.0%	99.5%	MIIVPKRVDPPLLIFAILPLALFVFKVGKILFLYRRTVGVDLRDSFFAALAGLSLSHTIAKAVLYGFVTRGIPFFRTPKM
8	*P. aeruginosa* PAK	100.0%	99.4%	MIIVPKRVDPPLLIFAILPLALFVFKVGKILFLYRRTVGVDLRDSFFAALAGLSLSHTIAKAVLYGFVTRGIPFFRTPKM
9	***P. fluorescens*** **SBW25**	99.0%	73.7%	MIIVPTRVDPPLLIFA-PPLALFVFKVGKIIFLYRRAVGVNLKDAFCAALAGLALSHTIAKAVLYGFFTTSIPFFRTPKN
10	*P. fluorescens* SS101	99.3%	73.6%	MIIVPTRVDPPLLIFAIPPLALFVFKVGKIIFLYRRAVGVNLKDAFCAALAGLALSHTIAKAVLYGFFTSSIPFFRTPKN
11	*P. fluorescens* ICMP 3512	99.3%	73.3%	MIIVPTRVDPPLLIFAIPPLALFVFKVGKIIFLYRRAVGVNLKDAFCAALAGLALSHTIAKAVLYGFFTTSIPFFRTPKN
12	*P. fluorescens* WS 5037	99.3%	73.8%	MIIVPTRVDPPLLIFAIPPLALFVFKVGKIIFLYRRAVGVNLKDAFCAALAGLALSHTIAKAVLYGFFTTSIPFFRTPKN
13	*P. fluorescens* ICMP 11288	99.3%	73.9%	MIIVPTRVDPPLLIFAIPPLALFVFKVGKIIFLYRRAVGVNLKDAFCAALAGLALSHTIAKAVLYGFFTTSIPFFRTPKN
14	*P. fluorescens* WH6	99.3%	73.9%	MIIVPTRVDPPLLIFAIPPLALFVFKVGKIIFLYRRAVGVNLKDAFCAALAGLALSHTIAKAVLYGFFTTSIPFFRTPKN
15	*P. fluorescens* KF1	99.3%	74.1%	MIIVPTRVDPPLLIFAIPPLALFVFKVGKIIFLYRRAVGVNLKDAFCAALAGLALSHTIAKAVLYGFFTTSIPFFRTPKN
16	*P. fluorescens* LMG 5329	99.3%	73.7%	MIIVPTRVDPPLLIFAIPPLALFVFKVGKIIFLYRRAVGVNLKDAFCAALAGLALSHTIAKAVLYGFFTTSIPFFRTPKN
17	*P. putida* S610	99.5%	75.7%	MIIVPHRVDPPLMIFAIPPLALFFFKVGKIVFLYRRAVGVNLKDALAAAVAGLALSHTIAKAVLYGFFTSSMPFFRTPKN
18	*P. putida* W619	99.3%	76.4%	MIIVPHRVDPPLMIFAIPPLALFFFKVAKIIFLYRRAVGVNLKDAFAAALAGLALSHTIAKAVLYGFFTSSMPFFRTPKN
19	***P. putida*** **KT2440**	99.3%	76.3%	MIIVPHRVDPPLMIFAIPPLALFFFKVGKIIFLYRRAVGVNLKDAFAAALAGLALSHTIAKAVLYGFFTSSMPFFRTPKN
20	*P. putida* YKD221	99.3%	76.3%	MIIVPHRVDPPLMIFAIPPLALFFFKVGKIIFLYRRAVGVNLKDAFAAALAGLALSHTIAKAVLYGFFTSSMPFFRTPKN
21	*P. syringae* ICMP 9617	95.3%	46.3%	MILRPEMLYPVPWIFSTSLLLMFTLRIVKVFFLYQQRVGSDVTEAMAAILAGMALYPTIGRAVLSGLFTSGLPFFRTPKQ
22	*P. syringae* B728a	95.3%	46.1%	MILKPETFGPLPWIFSTSLILMFALRSFKMIVLYRRLVSTHIKEALAAILAGMALYPTLGKAVLAGLFTSAMPFYRTPKH
23	*P. syringae* UMAF0158	95.0%	45.2%	MILKPETFGPLPWIFSTSLILMFALRSLKMIVLYRQLVSTHTKEALAAILAGMALYPTLGKAVLAGLFTSAMPFYRTPKH
24	***P. syringae*** **DC3000**	95.3%	45.4%	MIIQPDTLEPVPWIFSSSLLLMFVLGVCKAISLYQRLASTDIKDAFAAIIASMALYSVVGKAVLSSAFTSGLPFFRTPKQ
25	*P. syringae* NCPPB 4273	95.3%	47.3%	MIVEPDLLEPVPWLFSASLLLMFVLGIFKAFTLYQRLANTDIKDALAAMLASTALYSVIGKAVLSALFTSGLPFFRTPKQ
26	*P. syringae* 41a	95.3%	46.9%	MIVEPDLLEPVPWLFSASLLLMFVLGIFKAFTLYQRLANTDIKDALAAMLASTALYSVIGKAVLSALFTSGLPFVRTPKQ
	Consensus/100%			MIl.Pphh.P..hlFu...lhhFhht.hKhh.LYpphsssphp-uhhAhlAuhuL..slu+AVL.uhhTpuhPF.RTPK.
	Consensus/70%			MIIVPpRVDPPLhIFAI.PLALFsFKVGKIlFLYRRsVGVsL+DuFhAALAGLuLSHTIAKAVLYGFhTpulPFFRTPK.
	Motifs & residues			FxVTxK

	Species	Coverage	ID	 880
1	*P. aeruginosa* PA14	100.0%	100.0%	RSSHGLLVALAEAREEVFVMLLLWGAAAGIVAVQGVPSRDLLIWVAMLLVQSLPYLAALVMALLSSLPKPREELAGGAEQ
2	*P. aeruginosa* 19BR	100.0%	99.2%	RSSHGLLVALAEAREEVFVMLLLWGAAAGIVAVQGVPSRDLLIWVAMLLVQSLPYLAALVMALLSSLPKPREELAGGAER
3	*P. aeruginosa* BL14	100.0%	99.8%	RSSHGLLVALAEAREEVFVMLLLWGAAAGIVAVQGVPSRDLLIWVAMLLVQSLPYLAALVMALLSSLPKPREELAGGAEQ
4	*P. aeruginosa* 3573	100.0%	99.5%	RSSHGLLVALAEAREEVFVMLLLWGAAAGIVAVQGVPSRDLLIWVAMLLVQSLPYLAALVMALLSSLPKPREELAGGAER
5	*P. aeruginosa* LESB58	100.0%	99.2%	RSSHGLLVALAEAREEVFVMLLLWGAAAGIVAVQGVPSRDLLIWVAMLLVQSLPYLAALVMALLSSLPKPREELAGGAER
6	*P. aeruginosa* PA01	100.0%	99.4%	RSSHGLLVALAEAREEVFVMLLLWGAAAGIVAVQGVPSRDLLIWVAMLLVQSLPYLAALVMALLSSLPKPREELAGGAEQ
7	*P. aeruginosa* AZPAE12140	100.0%	99.5%	RSSHGLLVALAEAREEVFVMLLLWGAAAGIVAVQGVPSRDLLIWVAMLLVQSLPYLAALVMALLSSLPKPREELAGGAEQ
8	*P. aeruginosa* PAK	100.0%	99.4%	RSSHGLLVALAEAREEVFVMLLLWGAAAGIVAVQGVPSRDLLIWVAMLLVQSLPYLAALVMALLSSLPKPREELAGGAER
9	*P. fluorescens* SBW25	99.0%	73.7%	ADNHGFWVAISEAREEMFIMLLLWGAALGIYLVQGLPSNDIRFWVVMLLVQSLPYVAALVMAFLSSLPKPAPKVELATAE
10	*P. fluorescens* SS101	99.3%	73.6%	ADNHGFWVAISEAREEMFIMLLLWGAALGIYLVQGLPSNDIRFWVVMLLVQSLPYVAALIMAFLSSLPKPSAAPEAAPAA
11	*P. fluorescens* ICMP 3512	99.3%	73.3%	ADNHGFWVAISEAREEMFIMLLLWGAALGIYLVQGLPSNDIRFWVVMLLVQSLPYVAALIMAFLSSLPKPAPKGEPATAE
12	*P. fluorescens* WS 5037	99.3%	73.8%	ADNHGFWVAISEAREEMFIMLLLWGAALGIYLVQGLPSNDIRFWVVMLLVQSLPYVAALIMAFLSSLPKPAPKGEPATAE
13	*P. fluorescens* ICMP 11288	99.3%	73.9%	ADNHGFWVAISEAREEMFIMLLLWGAALGIYLVQGLPSNDIRFWVVMLLVQSLPYVAALIMAFLSSLPKPAPKVEPATAE
14	*P. fluorescens* WH6	99.3%	73.9%	ADNHGFWVAISEAREEMFIMLLLWGAALGIYLVQGLPSNDMRFWVVMLLVQSLPYVAALIMAFLSSLPKPAPAPEPAPAS
15	*P. fluorescens* KF1	99.3%	74.1%	ADNHGFWVAISEAREEMFIMLLLWGAALGIYLVQGLPSNDMRFWVVMLLVQSLPYVAALIMAFLSSLPKPAPKVEPVTVA
16	*P. fluorescens* LMG 5329	99.3%	73.7%	ADNHGFWVAISEAREEMFIMLLLWGAALGIYLVQGLPSNDIRFWVVMLLVQSLPYVAALIMAFLSSLPKPAAAPEPAPAA
17	*P. putida* S610	99.5%	75.7%	ADSHGLLVAISEAREELFIMLLLWGAAAGIYLVQGLPSSDMRFWVAMLLVQSLPYLAALVMAFLSSLPKPEEQATQKAVE
18	*P. putida* W619	99.3%	76.4%	ADSHGLLVAISEAREELFIMLLLWGAAAGIYLVQGLPSSDMRFWVAMLLVQSLPYLAALVMALLSSLPKPEHKMAEPQEA
19	*P. putida* KT2440	99.3%	76.3%	ADSHGLLVAISEAREELFIMVLLWGAALGIYLVQGLPSSDMRFWVAMLLVQSLPYVAALVMAFLSSLPKPAEKAAQAQQA
20	*P. putida* YKD221	99.3%	76.3%	ADSHGLLVAISEAREELFIMLLLWGAALGIYLVQGLPSSDMRFWVAMLLVQSLPYVAALVMAFLSSLPKPAEKAAQAQQA
21	*P. syringae* ICMP 9617	95.3%	46.3%	SSCNSFRQTIIEARQDFYVVMISLVAIVLLYIRKDTVDPDLGFWIAMLVAQSLPYLAAITMAILSARATRPALSTA----
22	*P. syringae* B728a	95.3%	46.1%	TSANRIGQNLLDVREELSTLAISWIAIVLLLTGRASIDTNSGFWITMLFAQSLPYLAAITMAILSARANRPALPTT----
23	*P. syringae* UMAF0158	95.0%	45.2%	TSANRIGQNLLDVREELSTLAISWIAIVLLLTGRASIDTNSGFWITMLFAQSLPYLAAITMAILSARATRPARSTT----
24	*P. syringae* DC3000	95.3%	45.4%	TSGSGLGKALLDVREDLYMAVVWWVMTVSLCFRKEAIGPDLGFWVAIMFAQSLPYVAAMIMAILSALANRPSRSTT----
25	*P. syringae* NCPPB 4273	95.3%	47.3%	TARTRFGQAVREATEDVCMIVLWWAAIILLCIRKETIDPDLGFWIAMLFAQSVPYLAAITMTILSARATRPAPSTA----
26	*P. syringae* 41a	95.3%	46.9%	TAHTRFGQAVREAAEDVCMILLWWAAIILLCIRKETIDPDLGFWITMLFAQSVPYLAAITMAVLSARATRPAPSTA----
	Consensus/100%			tstpthh.sl.-stp-h.hhhl.hhhhh.lhhhpt..s.s.hhWlshhhsQSlPYlAAhhMshLSuhsp.....t.....
	Consensus/70%			tssHGhhVAluEAREEhFlMLLLWGAAhGIhhVQGlPSpDhhhWVsMLLVQSLPYlAALlMAhLSSLPKPttp.tss..t
	Motifs & residues			RxxxR

	Species	Coverage	ID	
1	*P. aeruginosa* PA14	100.0%	100.0%	**IGG**
2	*P. aeruginosa* 19BR	100.0%	99.2%	**IGG**
3	*P. aeruginosa* BL14	100.0%	99.8%	**IGG**
4	*P. aeruginosa* 3573	100.0%	99.5%	**IGG**
5	*P. aeruginosa* LESB58	100.0%	99.2%	**IGG**
**6**	***P. aeruginosa* PA01**	100.0%	99.4%	**IGG**
7	*P. aeruginosa* AZPAE12140	100.0%	99.5%	**IGG**
8	*P. aeruginosa* PAK	100.0%	99.4%	**IGG**
**9**	***P. fluorescens* SBW25**	99.0%	73.7%	---
10	*P. fluorescens* SS101	99.3%	73.6%	---
11	*P. fluorescens* ICMP 3512	99.3%	73.3%	---
12	*P. fluorescens* WS 5037	99.3%	73.8%	---
13	*P. fluorescens* ICMP 11288	99.3%	73.9%	---
14	*P. fluorescens* WH6	99.3%	73.9%	---
15	*P. fluorescens* KF1	99.3%	74.1%	---
16	*P. fluorescens* LMG 5329	99.3%	73.7%	---
17	*P. putida* S610	99.5%	75.7%	**TP-**
18	*P. putida* W619	99.3%	76.4%	---
**19**	***P. putida* KT2440**	99.3%	76.3%	---
20	*P. putida* YKD221	99.3%	76.3%	---
21	*P. syringae* ICMP 9617	95.3%	46.3%	---
22	*P. syringae* B728a	95.3%	46.1%	---
23	*P. syringae* UMAF0158	95.0%	45.2%	---
**24**	***P. syringae* DC3000**	95.3%	45.4%	---
25	*P. syringae* NCPPB 4273	95.3%	47.3%	---
26	*P. syringae* 41a	95.3%	46.9%	---
	Consensus/100%			...
	Consensus/70%			...
	Motifs & residues			
