# Supplementary material for: Bioinformatics characterization of BcsA-like orphan proteins suggest they form a novel family of pseudomonad cyclic-β-glucan synthases
Source: PLoS One. 2023 Jun 2;18(6):e0286540. doi: 10.1371/journal.pone.0286540 (PMC10237404; doi:10.1371/journal.pone.0286540)
Supplement: S1 Table — This lists locus tags and UniProtKB accessions, PseudoCAP annotations, number of residues, genome coordinates, and amino acid identity and similarity to the EcBcsA reference protein. (PDF) [file pone.0286540.s008.pdf]

S1 Table. **BcsA and Orphan proteins from *Pseudomonas fluorescens* SBW25*, P. putida* KT2440 and *P. syringae* DC3000.**

	Protein	*PseudoCAP locus tag (UniProtKB accession)*	*PseudoCAP annotation*	*No. residues*	*Genome coordinates*	*% Identity (% Similarity) to Reference*
P. fluorescens SBW25	BcsA	PFLU_0301 (P58931)	Cellulose synthase catalytic subunit	739	328286 - 330505	51.7 (69.6)
	Orphan	PFLU_1260 (C3K5D6)	β-(1,3)-glucosyl transferase	863	1392128 - 1394719	24.7 (40.5)
P. putida KT2440	BcsA	PP_2635 (Q88JL4)	Cellulose synthase catalytic subunit	841	3013466 - 3016075	56.2 (71.1)
	Orphan	PP_1526 (Q88MP4)	β-(1,3)-glucosyl transferase	832	1731274 - 1733865	26.5 (42.5)
P. syringae DC3000	BcsA	PSPTO_1027 (Q888J7)	Cellulose synthase catalytic subunit	739	1121129 - 1123348	17.1 (34.0)
	Orphan	PSPTO_1524 (Q886Q3)	Glycosyl transferase family protein	842	1680211 - 1682739	26.5 (42.5)

The EMBOSS Water pairwise sequence alignment tool was used to determine the percentage amino acid identity and similarity to *Ec*BcsA (UniProtKB P37653) chosen as a reference sequence. See **S1 File** for FASTA files for all protein sequences.
