# Supplementary material for: Bioinformatics characterization of BcsA-like orphan proteins suggest they form a novel family of pseudomonad cyclic-β-glucan synthases
Source: PLoS One. 2023 Jun 2;18(6):e0286540. doi: 10.1371/journal.pone.0286540 (PMC10237404; doi:10.1371/journal.pone.0286540)
Supplement: S2 Table — This lists Pseudomonas species and strains containing Orphan genes identified in our sampling of PseudoCAP entries, by BLAST searches, and inspection of unpublished draft genome sequences. See S1 File for all protein sequences. (PDF) [file pone.0286540.s009.pdf]

S2 Table. **Orphan proteins identified in *Pseudomonas* species.**

Species	*Strain*	*Identification*
Pseudomonas aeruginosa	AZPAE12140, BL14, LESB58, PAK, PA01, PA14, 19BR, 3573 & KL28	PseudoCAP
Pseudomonas antarctica	BS2772 & PAMC 27494	BLAST
Pseudomonas azotoformans	F77 & S4	BLAST
Pseudomonas balearica	DSM6083	PseudoCAP
Pseudomonas canadensis	36C8	BLAST
Pseudomonas chlororaphis	PCL1606, YL-1 & 30–84	PseudoCAP
Pseudomonas cremoricolorata	ND07	PseudoCAP
Pseudomonas cremoris	WS 5106	BLAST
Pseudomonas deceptionensis	DSM 26521	PseudoCAP
Pseudomonas denitrificans	ATCC 13867	PseudoCAP
Pseudomonas entomophila	L48	PseudoCAP
Pseudomonas extremaustralis	PgKB38	BLAST
Pseudomonas fluorescens	SBW25, SS101 & WH6	PseudoCAP
	ICMP 3512, ICMP 11288, KF1, LMG 5329 & WS 5037	BLAST
Pseudomonas fulva	NBRC 16636	PseudoCAP
Pseudomonas helleri	DSM 29165	PseudoCAP
Pseudomonas knackmussii	B13	PseudoCAP
Pseudomonas lini	DSM 16768	PseudoCAP
Pseudomonas lundensis	DSM 6252	PseudoCAP
Pseudomonas lurida	LMG 21995 & MYb17	BLAST
Pseudomonas lutea	DSM 17257	PseudoCAP
Pseudomonas mandelii	JR-1	PseudoCAP
Pseudomonas marginalis	ICMP 3555	BLAST
Pseudomonas monteilii	SB3101	PseudoCAP
Pseudomonas moraviensis	R28-S	PseudoCAP
Pseudomonas mosselii	DSM 17497	PseudoCAP
Pseudomonas nitroreducens	Aramco J	PseudoCAP
Pseudomonas orientalis	CDVBN20, DSM 17489, F9 & 133NRW	BLAST
Pseudomonas otitidis	LNU-E-001	PseudoCAP
Pseudomonas panacis	PgKB35 & WS 4668	BLAST
Pseudomonas parafulva	YAB-1	PseudoCAP
Pseudomonas plecoglossicida	NyZ12	PseudoCAP
Pseudomonas poae	RE 1-1-14	PseudoCAP
Pseudomonas protegens	CHA0	PseudoCAP
Pseudomonas putida	KT2440, S610, W619 & YKD221	PseudoCAP
Pseudomonas reactans	IPO375 & P8021	BLAST
Pseudomonas resinovorans	CA10	PseudoCAP
Pseudomonas salomonii	ICMP 11288	BLAST
Pseudomonas simiae	WCS417	PseudoCAP
Pseudomonas synxantha	BG33R	PseudoCAP
Pseudomonas syringae	B728a, DC3000, ICMP 9617, NCPPB 4273, UMAF0158 & 41a	PseudoCAP
Pseudomonas taeanensis	MS-3	PseudoCAP
Pseudomonas taetrolens	DSM 21104	PseudoCAP
Pseudomonas taiwanensis	DSM 21245	PseudoCAP
Pseudomonas thermotolerans	J53	PseudoCAP
Pseudomonas trivialis	IHBB745	PseudoCAP
Pseudomonas tuomuerensis	JCM 14085	PseudoCAP

Pseudomonas umsongensis	20MFCvi1.1	PseudoCAP
Pseudomonas veronii	R4	PseudoCAP
Pseudomonas viridiflava	LMCA8	PseudoCAP
Pseudomonas vranovensis	DSM 16006	PseudoCAP
Pseudomonas weihenstephanensis	DSM 29166	PseudoCAP
Pseudomonas spp.	ADAK22, AP19 & C 49-2	PseudoCAP
	DBG-1, DBG-3, DBG-6, DBG-15, DBG-16, DBG-23 & NZ092	Unpublished draft genomes
	FH1, IB20, LBUM920, Leaf15, Leaf98, NS1 2017, OV546, PGPPP1, P7759, QC2, RGB, R9.37, S10E 269, WP001, WS 5532, 44 R 15, 58 R 3 & 2995	BLAST

Orphan proteins identified in PseudoCAP and from our unpublished draft genomes are confirmed to be immediately upstream of a *dapE* homolog. We note that no Orphan proteins were identified in the opportunistic human pathogen *P. stutzeri* which is well-represented in PseudoCAP. The genetic context of the Orphan genes identified by BLAST has not been confirmed.
