# Supplementary material for: Bioinformatics characterization of BcsA-like orphan proteins suggest they form a novel family of pseudomonad cyclic-β-glucan synthases
Source: PLoS One. 2023 Jun 2;18(6):e0286540. doi: 10.1371/journal.pone.0286540 (PMC10237404; doi:10.1371/journal.pone.0286540)
Supplement: S3 Table — This lists reference transmembrane helices, α-helices and β-sheets identified in the Rhizomucor miehei CAU432 and Rhodobacter sphaeroides 2.4.1 BcsA X-ray crystal structures and seen in the single-domain models and predicted structures of the Pseudomonas fluorescens SBW25 Orphan protein. The start and stop residues for each structure is recorded showing the variation from that shown in the Phyre2 homology models for the GH17 domain and the TM region and GT2 domain. (PDF) [file pone.0286540.s010.pdf]

S3 Table. **Comparison of secondary structures in *Pf* SBW25 Orphan homology models and predicted structures.**

	Secondary structure prediction		*Homology modelling*		*Predictive modelling*			
**(A) Signal peptide**	*HMMER* [a]	*Proteus2*	*Phyre*^{2} [b]	*SWISS-MODEL* [c]	*AlphaFold*	*InterFOLD6*	*RoseTTAFold*	*TrRosetta*
Signal peptide α-helix	1 – 25	8 – 27			1 – 27	Disordered	1 – 27	1 – 27
**(B) Structures associated with *Rm* Bgt17A GH17 domain**								
α-helix 1 (Bgt17A 17 – 29)	–	62 – 74	62 – 72	62 – 73	62 – 73	62 – 73	62 – 63	62 – 72
α-helix 2 (Bgt17A 41 – 57)	–	90 – 97	87 – 98	89 – 97	86 – 97	88 – 98	86 – 98	87 – 97
α-helix 3 (Bgt17A 70 – 86)	–	112 – 125	110 – 126	113 – 125	112 – 128	112 – 125	112 – 128	112 – 127
α-helix 4 (Bgt17A 107 – 126)	–	149 – 161	149 – 160	149 – 162	149 – 162	149 – 162	149 – 160	149 – 161
α-helix 5 (Bgt17A 139 – 144)	–	–	183 – 185	180 – 185	180 – 185	180 – 185	180 – 184	180 – 184
α-helix 6 (Bgt17A 161 – 179)	–	203 – 219	202 – 218	202 – 218	202 – 219	202 – 219	202 – 219	202 – 219
α-helix 7 (Bgt17A 205 – 223)	–	246 – 262	246 – 262	246 – 263	246 – 262	246 – 262	246 – 262	246 – 262
α-helix η5 (Bgt17A 239 – 245)	–	–	279 – 281	276 – 281	277 – 282	Disordered	278 – 280	Disordered
Conserved start / stops compared with the Phyre^2 *homology model*		3 & 1	(Reference)	4 & 3	4 & 2	4 & 3	4 & 3	5 & 2
**(C) Structures associated with *Rs BcsA* TM region and GT2 domain**								
TM helix 1 (BcsA 40 – 60)	312 – 332	310 – 332	315 – 333		312 – 330	316 – 332	312 – 333	310 – 333
TM helix 2 (BcsA 64 – 88)	344 – 361	344 – 361	339 – 363		339 – 364	341 – 364	338 – 365	339 – 366
TM helix 3 (BcsA 97 – 122)	367 – 391	371 – 392	372 – 394		370 – 401	375 – 401	370 – 402	370 – 402
β-sheet 1 (BcsA 143 – 149)	–	421 – 428	422 – 427	422 – 428	421 – 428	422 – 427	422 – 428	422 – 428
Amphipathic helix IF1 (BcsA 154 – 165) [d]	–	433 – 445	433 – 444	433 – 444	433 – 444	433 – 445	433 – 444	433 – 444
β-sheet 2 (BcsA 174 – 180)	–	451 – 457	451 – 456	452 – 457	451 – 458	452 – 456	451 – 458	451 – 458
β-sheet 3 (BcsA 216 – 218)	–	480 – 485	481 – 484	480 – 483	480 – 485	480 – 484	480 – 485	480 – 485
β-sheet 4 (BcsA 242 – 245)	–	508 – 513	509 – 514	509 – 512	507 – 514	509 – 513	509 – 513	509 – 512
β-sheet 5 (BcsA 270 – 275)	–	536 – 540	538 – 540	536 – 541	536 – 541	536 – 540	538 – 540	536 – 542
β-sheet 6 (BcsA 323 – 326) [e]	–	584 – 594 (H)	585 – 588	585 – 588	585 – 588	585 – 588	584 – 588	585 – 588
β-sheet 7 (BcsA 357 – 361)	–	617 – 621	618 – 620	617 – 621	617 – 621	617 – 620	619 – 620	617 – 624
Amphipathic helix IF2 (BcsA 374 – 394) [f]	–	634 – 655	634 – 654	634 – 654	634 – 654	634 – 653	634 – 661	634 – 661
TM helix 4 (BcsA 405 – 437)	681 – 703	677 – 697	669 – 701 [g]	669 – 701	669 – 704	671 – 701	668 – 704	669 – 704
TM helix 5 (BcsA 446 – 468)	715 – 737	714 – 735	710 – 732	710 – 724	713 – 737	710 – 728	713 – 737	713 – 738
TM helix 6 (BcsA 476 – 497) [g]	749 – 771	748 – 769	750 – 768 (H) [h]	747 – 768 (H) [h]	742 – 768 (H) [h]	747 – 767 (H) [h]	741 – 754 (H) [h]	742 – 768 (H) [g]
TM helix 7 (BcsA 523 – 545)	800 – 819	799 – 816	792 – 816	790 – 811	794 – 814	791 – 813	794 – 814	786 – 814

TM helix 8 (BcsA 547 – 574)	826 – 847	825 – 846	828 – 843	822 – 846	819 – 831 & 833 – 845	822 – 846	818 – 846	819 – 844
Conserved start / stops compared with the Phyre[2] homology model	1 & 2	3 & 2	(Reference)	7 & 5	6 & 5	6 & 7	6 & 5	8 & 4
**(D) C-terminal region**								
Disordered residues					846 – 860	850 – 860	847 –860	847 – 860

Secondary structures were used to qualitatively compare *Pf* SBW25 Orphan protein homology models and predicted structures. These included the α-helices involved in the GH17 TIM-barrel, the transmembrane TM helices associated with the GT2 domain, GT2 amphipathic helices, and a series of six parallel (and one anti-parallel) β-sheets in the GT2 domain that form the α/β/α sandwich. –, Not predicted. H, modelled or predicted as an α-helix rather than as a β-sheet or TM helix. Secondary structure predictions and models were scored according to whether these structures had the same starting and finishing residues compared to the Phyre[2] homology model (Reference).

[a] HMMERScan does not report α-helices other than transmembrane helices and signal peptides or β-sheets.

[b] Two Phyre[2] single-domain homology models are compared here. The GH17 homology model included residues 37 – 306 and lacks the signal peptide sequence. The TM region and GT2 domain homology model included residues 314 – 856.

[c] Two SWISS-MODEL single-domain homology models are compared here. The GH17 homology model included residues 36 – 305 and lacks the signal peptide sequence. The TM region and GT2 domain homology model included residues 416 – 855 and lacks the sequences mapping to TM1 – TM3.

[d] IF1 is located away from the cytoplasmic surface of the inner membrane and incorporates a significant bend.

[e] This β-sheet is antiparallel to β-sheets 1 – 5 & 7.

[f] IF2 is perpendicular to the main axis of the transmembrane helices and lies immediately underneath the cytoplasmic surface of the inner membrane.

[g] This helix incorporates a significant bend.

[h] This helix lies perpendicular to main axis of the transmembrane helices and is located at the base near the cytoplasmic surface of the inner membrane.
