# Supplementary material for: Bioinformatics characterization of BcsA-like orphan proteins suggest they form a novel family of pseudomonad cyclic-β-glucan synthases
Source: PLoS One. 2023 Jun 2;18(6):e0286540. doi: 10.1371/journal.pone.0286540 (PMC10237404; doi:10.1371/journal.pone.0286540)
Supplement: S4 Table — This lists the non-covalent bonds identified in the AlphaFold, InterFOLD6, RoseTTAFold and TrRosetta predicted structures of the Pseudomonas fluorescens SBW25 Orphan protein connecting residues located in the GH17 domain, linking (unstructured) sequence, and the exposed surface of the TM region. (PDF) [file pone.0286540.s011.pdf]

S4 Table. **Interactions between GH17 domain and TM region residues in *Pf* SBW25 Orphan predicted structures.**

	Links between the GH17 domain, linking sequence and TM region	Non-covalent bond
AlphaFold	GH17 domain – Linking sequence	ASN 28 | OD1 – ILE 309 | N
	GH17 domain – Linking sequence	VAL 31 | N – VAL 306 | O
	GH17 domain – TM region	ARG 237 | NH1 – PRO 706 | O
	GH17 domain – TM region	ARG 237 | NH2 – GLN 814 | OE1
	Linking structure – TM region	SER 284 | OG – ASP 710 | OD2
	Linking structure – TM region	TYR 288 | OH – TYR 367 | N
InterFOLD	GH17 domain – TM region	ARG 29 | O – LYS 726 | NZ
	GH17 domain – TM region	VAL 31 | N – PHE 725 | O
	GH17 domain – TM region	VAL 31 | O – ILE 731 | N
	GH17 domain – TM region	ASN 35 | ND2 – TRP 402 | O
	GH17 domain – TM region	TRP 36 | NE1 – GLN 399 | O
	GH17 domain – Linking sequence	GLN 39 | NE2 – GLY 304 | O
	GH17 domain – Linking sequence	ARG 254 | O – VAL 307 | N
	Linking structure – TM region	GLN 298 | OE1 – TYR 673 | OH
RoseTTAFold	GH17 domain – TM region	ASN 9 | OD1 – ARG 340 | NH1
	GH17 domain – Linking sequence	ARG 29 | NE – ILE 309 | O
	GH17 domain – Linking sequence	THR 31 | OG1 – VAL 306 | O
	GH17 domain – Linking sequence	ALA 33 | O – VAL 306 | N
	GH17 domain – Linking sequence	ARG 254 | NH1 – GLY 304 | O
TrRosetta	GH17 domain – Linking sequence	VAL 31 | N – LYS 299 | O
	GH17 domain – Linking sequence	GLN 55 | NE2 – ALA 308 | O
	GH17 domain – TM region	ARG 237 | NH1 & 2 – ASP 710 | OD2
	GH17 domain – Linking sequence	GLN 275 | OE1 – ALA 308 | N
	GH17 domain – TM region	ASP 281 | OD1 – TRP 358 | NE1

Non-covalent bonds were identified using Mol* 3D View. Bonds between residues less than 30 residues apart were ignored. The boundaries between the GH17 domain (residues ~26 – 282), linking (unstructured) sequence (~283 – 312), and the periplasm-exposed TM region (residues ~310 – 400 and ~668 – 846) are arbitrary and differ slightly between models.
