# Supplementary material for: Bioinformatics characterization of BcsA-like orphan proteins suggest they form a novel family of pseudomonad cyclic-β-glucan synthases
Source: PLoS One. 2023 Jun 2;18(6):e0286540. doi: 10.1371/journal.pone.0286540 (PMC10237404; doi:10.1371/journal.pone.0286540)
Supplement: S5 Table — This lists MdoB homologs identified in Pseudomonas aeruginosa PA01, P. fluorescens SBW25, P. putida KT2440, and P. syringae DC3000. (PDF) [file pone.0286540.s012.pdf]

S5 Table. **Homologs of the *Escherichia coli* Phosphoglycerol transferase MdoB.**

	Locus tag / UniProtKB	*UniProtKB annotation / localisation*	*Alignment length*	*% Identity*
P. aeruginosa PA01	PA1115 / Q9I4M0	Sulfatase domain-containing protein / cytoplasmic membrane	150	26.7
	PA1689 / Q9I338 *	Sulfatase domain-containing protein / cytoplasmic membrane	261	24.5
P. fluorescens SBW25	PFLU4327 / C3K075	Putative sulfatase / cytoplasmic membrane	273	22.7
	PFLU4985 / C3K198	Putative sulfatase / cytoplasmic membrane	242	26.8
P. putida KT2440	PP1838 / Q88LT9 *	Sulfatase domain-containing protein / cytoplasmic membrane	276	23.1
	PP2852 / Q88IZ7	Putative Sulfatase domain protein / cytoplasmic membrane	254	27.1
	PP2974 / Q88IM5	Putative sulfatase / cytoplasmic membrane	270	24.4
P. syringae DC3000	PSPTO2056 / Q884N2 *	Membrane protein / cytoplasmic membrane	347	23.9

PFLU4327 was first identified as a homolog of the *E. coli* K12 MdoB protein (UniProtKB P39401) using PseudoCAP BLASTP [60]. PseudoCAP was then used to identify PFLU4327 homologs in other *Pseudomonas*, while further BLASTP analyses identified additional *Ec*MdoB homologs in *P. aeruginosa* PA01, *P. fluorescens* SBW25, and *P. putida* KT2440.

[a] *P. fluorescens* SBW25 PFLU4327 homologs.
